# Deep learning driven de novo drug design based on gastric proton pump structures

**DOI:** 10.1038/s42003-023-05334-8

**Published:** 2023-09-19

**Authors:** Kazuhiro Abe, Mami Ozako, Miki Inukai, Yoe Matsuyuki, Shinnosuke Kitayama, Chisato Kanai, Chiaki Nagai, Chai C. Gopalasingam, Christoph Gerle, Hideki Shigematsu, Nariyoshi Umekubo, Satoshi Yokoshima, Atsushi Yoshimori

**Affiliations:** 1 Cellular and Structural Physiology Institute, Nagoya University, Nagoya, Aichi 464-8601 Japan; 2 Graduate School of Pharmaceutical Sciences, Nagoya University, Nagoya, Aichi 464-8601 Japan; 3 Center for One Medicine Innovative Translational Research (COMIT), Nagoya University, Nagoya, Aichi 464-8601 Japan; 4 INTAGE Healthcare, Inc., 3-5-7, Kawaramachi Chuo-ku, Osaka, 541-0048 Japan; 5 RIKEN SPring-8 Center, Kouto, Sayo-gun, Hyogo, 679-5148 Japan; 6 Japan Synchrotron Radiation Research Institute (JASRI), SPring-8, 1-1-1 Kouto, Sayo, Hyogo, 679-5148 Japan; 7 Institute for Theoretical Medicine, Inc., 26-1, Muraoka-Higashi 2-chome, Fujisawa, Kanagawa 251-0012 Japan

**Keywords:** Membrane proteins, Drug discovery and development, Cryoelectron microscopy

## Abstract

Existing drugs often suffer in their effectiveness due to detrimental side effects, low binding affinity or pharmacokinetic problems. This may be overcome by the development of distinct compounds. Here, we exploit the rich structural basis of drug-bound gastric proton pump to develop compounds with strong inhibitory potency, employing a combinatorial approach utilizing deep generative models for de novo drug design with organic synthesis and cryo-EM structural analysis. Candidate compounds that satisfy pharmacophores defined in the drug-bound proton pump structures, were designed in silico utilizing our deep generative models, a workflow termed Deep Quartet. Several candidates were synthesized and screened according to their inhibition potencies in vitro, and their binding poses were in turn identified by cryo-EM. Structures reaching up to 2.10 Å resolution allowed us to evaluate and re-design compound structures, heralding the most potent compound in this study, DQ-18 (*N*-methyl-4-((2-(benzyloxy)-5-chlorobenzyl)oxy)benzylamine), which shows a *K*_i_ value of 47.6 nM. Further high-resolution cryo-EM analysis at 2.08 Å resolution unambiguously determined the DQ-18 binding pose. Our integrated approach offers a framework for structure-based de novo drug development based on the desired pharmacophores within the protein structure.

## Abstract (web summary)

Deep generative models are used to develop gastric proton pump blockers with novel chemical skeletons and strong inhibitory potency.

## Introduction

To better meet medical needs, improvement of existing drugs’ efficacy is highly desired due to problems caused by harmful side effects, poor pharmacokinetics or low binding affinity. Designing new compounds by derivation, sometimes prevented by patent restriction, and phenotype screening often leads to unsatisfactory results. Recent advances in structural biology eases access to drug bound protein structures. However, even when available, structural data on the drug-target interaction remains hard to exploit for the design of novel compounds. To this end, establishing a framework that enables the production of a compound with a radically different chemical skeleton and effectiveness is required, which we attempt to address here by exploiting the accumulation of structural and functional information of drug-bound gastric proton pump^1,2^.

Painful symptoms of acid-related gastric diseases such as peptic ulcers or gastroesophageal reflux disease are associated with disorders of the gastrointestinal tract^3^. Gastric mucosal injury due to continuous use of nonsteroidal anti-inflammatory drugs (NSAIDs)^4^ or gastrin-producing tumors may also cause peptic ulcers^5^. Current therapies to treat these conditions either prevent the stimulation of parietal cells by antagonizing histamine H2 receptors^6^ or inhibit the final step in acid production by targeting the gastric proton pump, H^+^,K^+^-ATPase. Eradication of *Helicobacter pylori*, the main cause of gastric ulcers and gastric cancer, has been accomplished by the suppression of gastric acid secretion in combination with antibiotic treatment^7^. The molecular targets of acid suppressants, H^+^,K^+^-ATPase, is a gastric proton pump that mediates H^+^ export in exchange for luminal K^+^ across the parietal cell membrane accompanied by ATP hydrolysis^8,9^. Like other P-type ATPases^10,11^, vectorial cation transport is accomplished by the cyclical conversion of the pump consisting of four cornerstone states; E1 − E1P − E2P − E2, according to the so-called Post-Albers scheme^12^, which describes alternating access and affinity for the H^+^ and its counterion K^+^. Cytoplasmic-facing E1 and luminal-facing E2P states show high affinity for H^+^ and K^+^, respectively^13^. H^+^,K^+^-ATPase consists of two subunits. The catalytic α-subunit (100 kDa) comprises 10 transmembrane (TM) helices in which the cation and inhibitor binding sites are located and three cytoplasmic domains (the nucleotide-binding (N), phosphorylation (P), and actuator (A) domains) executing ATP hydrolysis and autophosphorylation. An accessory β-subunit (35 kDa) has a single TM helix with a short N-terminal tail and an ectodomain with six *N*-linked glycosylation sites, and is required for the folding of the complex and membrane trafficking^14^.

Proton pump inhibitors (PPIs) such as omeprazole have been utilized for acid suppression. The PPI drug itself is a prodrug, requiring acid activation to irreversibly inhibit H^+^,K^+^-ATPase by forming a covalent bond with its Cys813 residue^15^. However, given its relatively short plasma half-life and requirement for an acidic pH to convert the prodrug to the active compound, considerable effort has been expended to develop different types of H^+^,K^+^-ATPase inhibitors. The K^+^-competitive acid blockers (P-CABs) differ from the PPIs in that they are not dependent on acid activation, are rather stable in the acidic canaliculus, and bind directly to the proton pump, thereby providing a more rapid onset and better inhibition of acid secretion^3^. The binding of omeprazole and P-CABs is mutually exclusive, indicating these drugs share an overlapping binding site^16^. They are currently in clinical use in some Asian countries. Although the prototypic P-CAB, the imidazo[1,2-*a*]pyridine derivative SCH28080^17^, is unsuitable for clinical use due to its hepatotoxicity, its benzimidazole derivative tegoprazan^18^ was approved in 2018 in South Korea. Besides SCH28080-related compounds, chemically distinct pyrimidine-based revaprazan^19^ (approved for clinical use in 2007 in South Korea) and pyrrole derivative vonoprazan^20^ (approved for clinical use in 2015 in Japan), have been developed (Supplementary Fig. 1). These P-CABs are proving to be successful, providing rapid and reliable cures for acid-related gastrointestinal diseases. On the other hand, the relationship between long-term administration of PPI or P-CAB and their effects on intestinal microflora^21^, interstitial nephritis^22^ and gastric cancer^23^ have also received attention. Therefore, further pharmacokinetic improvement and increased binding affinity is desired to reduce the required drug dosage. Furthermore, the availability of alternative compounds is expected to broaden the treatment options and expand its clinical use. Because all the above drugs have been developed based on phenotypic screening, there is room for further improvement, specifically, the development of novel drugs using structure-based rational design.

**Fig. 1:**
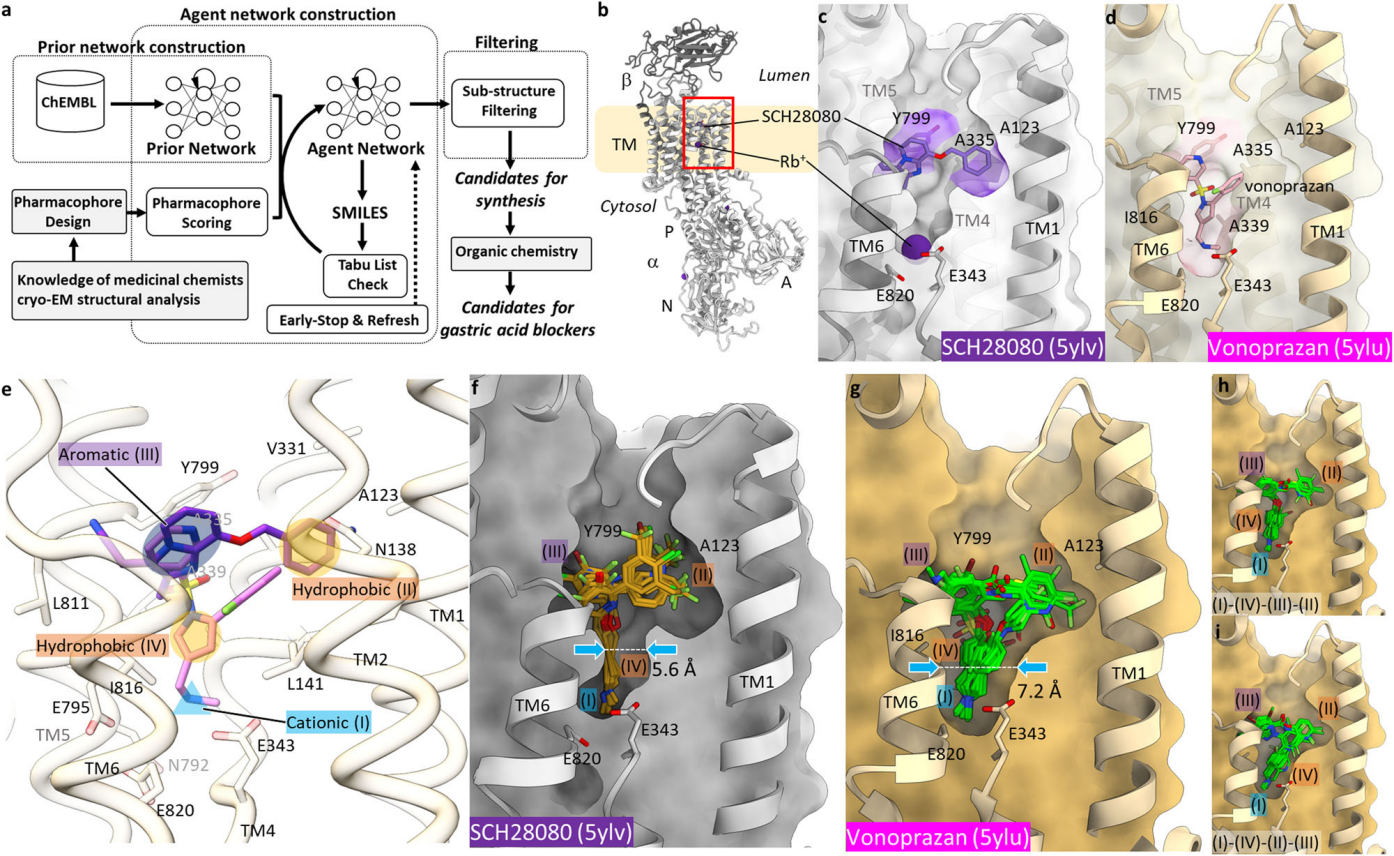
De novo drug design based on the desired pharmacophores. **a** Workflow of “Deep Quartet”. See main text and “Methods” for details. **b** Crystal structure of the gastric H^+^,K^+^-ATPase complexed with SCH28080 (PDB ID: 5YLV, gray) in ribbon representation, viewed parallel to the membrane plane with its luminal-side facing upwards. **c**, **d** Close-up view of the P-CAB-binding site (indicated as a red box in **b**). Clipped cross section of the luminal-facing conduit (surface) of SCH28080- (gray) and vonoprazan- (PDB ID: 5YLU, wheat) bound forms from the viewpoint similar to (**b**). Positions of P-CAB binding are indicated as purple or pink surface for SCH28080 or vonoprazan, respectively. **e** Defined pharmacophore features (I–IV, see text for details) on the luminal-facing cavity of the H^+^,K^+^-ATPase structure. Navy and yellow ovals, and blue triangle indicates pharmacophore features with aromatic, hydrophobic or cationic properties, respectively. **f** Binding poses of candidate compounds in the template structure (5YLV) calculated by Deep Quartet. **g** Same as (**f**) but with vonoprazan-bound form used as a template (5YLU). The most constrained positions of the lumina-facing conduit in each structure are indicated as dotted lines and blue arrows, with accompanying distance in Å. Two distinct binding modes of candidates from vonoprazan-bound form are displayed separately in (**h**) and (**i**). In (**c**, **d**, **f**–**i**), TM2 is omitted for clarity.

So far we have reported X-ray crystal and cryo-EM derived structures of H^+^,K^+^-ATPase complexed with seven different P-CABs; SCH28080^2^, vonoprazan^2^, BYK99^24^, tegoprazan^1^, PF03716556^1^, soraprazan^1^ and revaprazan^1^ (Supplementary Fig. 1). All P-CABs bind to the luminal-facing cavity leading to the cation-binding site in the luminal-open E2P state, and physically block K^+^-entry to the cation-binding site, hence preventing luminal gate closure which is induced by K^+^-binding to the cation-binding site. The binding mode of some of P-CABs are, however, significantly distinct; while SCH28080 (Fig. 1b, c) and its related compounds bind around the entrance of the luminal cavity, vonoprazan (Fig. 1d) and to some extent revaprazan binds more deeply toward the cation-binding site, indicating there are multiple pharmacophores in the P-CAB binding site. To date, however, not a single P-CAB satisfies all these pharmacophores. Therefore, if a compound that satisfies the greatest common denominator of these pharmacophores can be created, it is expected to be highly potent in gastric acid suppression.

Deep generative models (DGMs) have been successfully applied in various fields, including image processing^25^, language translation^26^ and the generation of chemical structures for drug development^27–30^. Despite its remarkable advances, most of the previous studies utilizing DGMs for compound generation focused on optimizing molecular properties such as logP (lipophilicity), QED (Quantitative Estimate Drug-likeness score)^31^ and SA (Synthetic Accessibility) score calculated on a 2D representation of the molecule^32^, and the application of DGMs to the actual drug discovery for the specific target is surprisingly limited so far^28,30^. Given the potential of DGMs to optimize compound chemical structures based on a biological target derived pharmacophore 3D coordinates, the appropriate application of DGMs is expected to significantly accelerate drug discovery.

Here, we demonstrate the approach by focusing on improving gastric proton pump blockers, where our deep learning-driven drug design based on the desired pharmacophores in the target protein structure (Fig. 1a) was capable of suggesting useful compounds with distinct chemical skeletons. Subsequent rounds of candidate selection, synthesis, in vitro drug screening and high-resolution cryo-EM structure analysis allows for successful de novo drug design resulting in a greatly facilitated process of drug discovery.

## Results and discussion

### De novo design of P-CABs based on the desired pharmacophores

Based on the reported structures of the H^+^,K^+^-ATPase complexed with different P-CABs, we tried to define the vital pharmacophores for P-CAB binding. SCH28080, known as an ancestor compound of P-CABs, and its related compounds (BYK99, PF-03716556 and soraprazan) or a drug (tegoprazan) (Supplementary Fig. 1), share a similar binding site at the entrance of the luminal cavity leading to the cation-binding site where Rb^+^ is bound as a congener of K^+^ in the SCH28080-bound form (Fig. 1b, c, e). The imidazo[1,2-*a*]pyridine ring of SCH28080 is bound to Tyr799 via π-π interaction^33^, and its benzyl group makes hydrophobic contacts with residues in TM1 (Ala123), TM2 (Asn138) and TM4 (Val331, Ala335)^34^. On the other hand, vonoprazan has a unique chemical structure and harbors thus far, to the best of our knowledge, the most potent in vitro inhibition activity amongst P-CAB. Its methylamino group reaches near the cation-binding site located deep in the conduit thus preventing K^+^-binding, while its pyridine ring keeps Tyr799 at the luminal entrance (Fig. 1d, e)^2^. A recently reported revaprazan bound structure shows the intermediate binding pose between SCH28080-type compounds and vonoprazan; while the pyrimidine and fluorophenyl rings of revaprazan overlap the binding pose of SCH28080, its tetrahydroisoquinoline moiety is accommodated in the middle of conduit and faces toward the cation-binding site^1^.

In order to design unique P-CABs by “Deep Quartet” (DQ), a de novo design workflow for generating molecules with a desired pharmacophore (Fig. 1a), we defined multiple features for pharmacophores, based on the above-described structural information (Fig. 1e and Supplementary Fig. 2).

(I) A cationic and hydrogen bond donor pharmacophore feature near the cation-binding site: the amino group of vonoprazan binds close to the K^+^-binding site where three glutamates (Glu343, Glu795 and Glu820) are located.
(II) A hydrophobic pharmacophore feature between TM1 and TM4, near the residue Ala123: the terminal hydrophobic group of SCH28080-related P-CABs binds at this position; the Ala123Val mutant shows significantly reduced affinity for SCH28080 and related compounds, but almost no effect for vonoprazan^2^.
(III) An aromatic pharmacophore feature close to Tyr799: the aromatic group of all P-CABs interacts with the side chain of Tyr799, and mutation of this residue (Tyr799Ala) severely reduces the apparent affinity of all P-CABs^1,2,34^.
(IV) A hydrophobic pharmacophore feature connecting I and III: the conduit connecting cation-binding site and luminal solution is mostly hydrophobic; the aromatic five-membered ring of vonoprazan, and the hydrophobic tetrahydroisoquinoline moiety of revaprazan occupy this position.

These four pharmacophore features (I–IV) were set alongside the crystal structure of SCH28080- (PDB ID: 5YLV) or vonoprazan-bound (PDB ID: 5YLU) forms, and we subsequently generated candidate compound structures by DQ. After iterative processing, compounds having a methylamino group were extracted. Although the two crystal structures used as templates are almost identical (RMSD for Cα atoms = 0.755 Å), at least same conformation, to our surprise, generated candidate compounds from each template structures are distinct. Characteristic compound structures with an alkyne moiety were generated when SCH28080-bound structure is used as a template (Fig. 1f and Supplementary Fig. 2 Type A). Likewise, when we set two aromatic rings at pharmacophore feature III position (akin to the heterocyclic ring structure of the imidazo[1,2-*a*]pyridine ring of SCH28080 is in mind) of the SCH28080-bound form, similarly alkyne-connecting, but incorporating heteroaromatic ring structures were generated (Fig. 1f and Supplementary Fig. 2 Type B). In contrast, aromatic-rich compounds were generated from the vonoprazan-bound structure (Fig. 1g–i and Supplementary Fig. 2 Type C). DQ generated diverse compounds for each pharmacophore type, among which 71, 10 and 181 compounds were selected as candidates with pharmacophore scores (see “Methods”) above 0.90 (Supplementary Fig. 3). Synthetic accessibility scores^32^ suggest that most of the DQ-generated candidates show mean values of around 3, which is similar to the score for most drug-like compounds available for clinical use, thus capable to synthesize (Supplementary Fig. 3). From the suggested candidates by DQ, several compounds were selected and synthesized (Supplementary Fig. 4), and their inhibition potencies and binding poses are characterized in the following sections.

### Alkyne-backbone of DQ-02 and related compounds

**Fig. 2:**
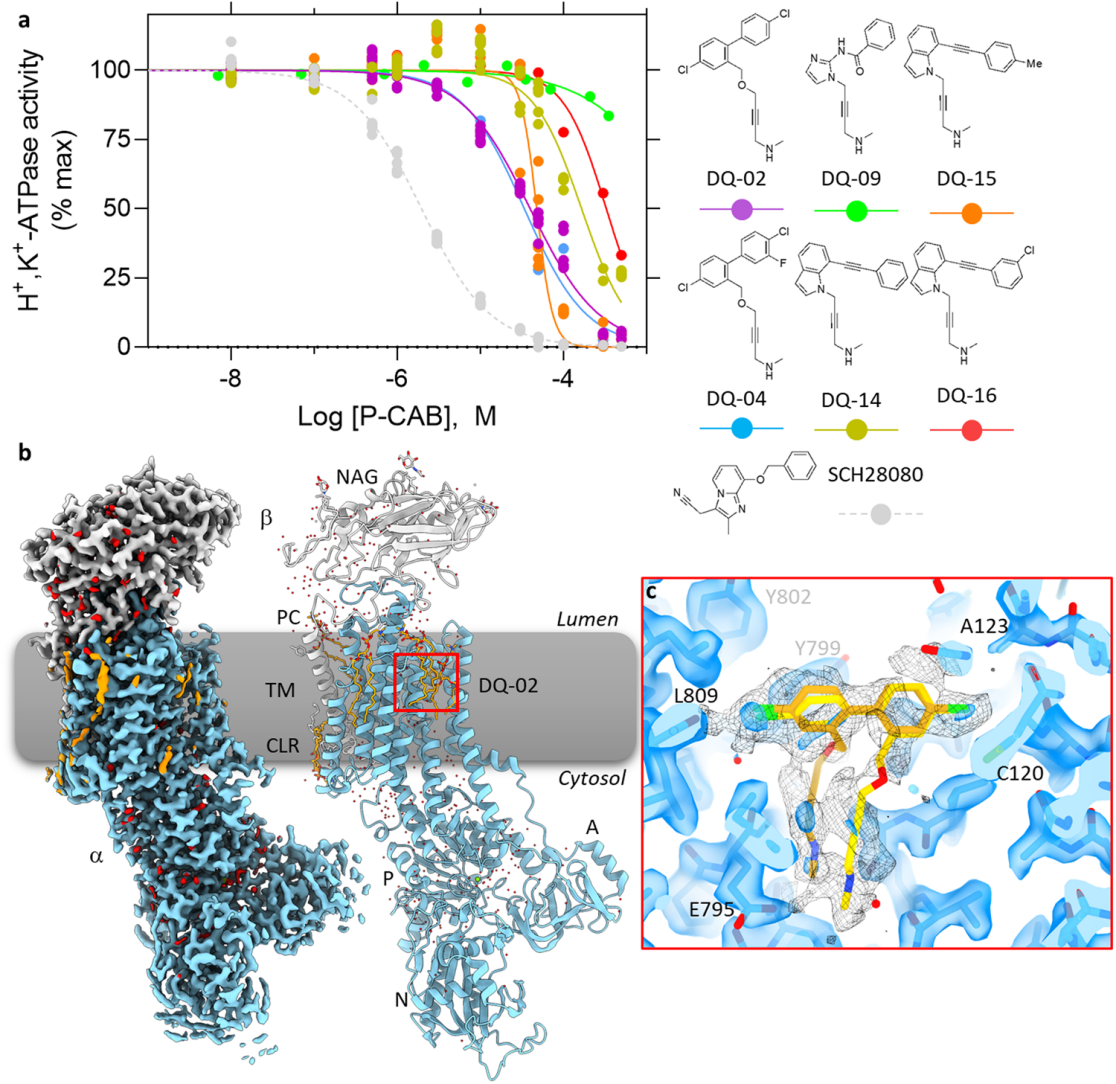
Inhibition potency and the binding pose of DQ-02. **a** Dose-dependent inhibition of H^+^,K^+^-ATPase activity by indicated synthesized compounds (SCH28080 as a control, gray circles and a dashed line). Data plotted represent each data point from triplicate of three independent measurement at 12 different concentrations of P-CABs, using membrane fractions purified from pig stomach. **b** EM potential map (colored surface) and cartoon model of the gastric H^+^,K^+^-ATPase complexed with DQ-02 (α-subunit, skyblue; β-subunit, gray; lipids, orange; waters, red). **c** Close-up view of the DQ-02 binding site indicated by the red box in (**b**). Transparent blue surface and black mesh represent EM potential maps in high and low-contour levels, respectively. Only the EM density around DQ-02 is displayed for low-contour map (mesh). Two possible conformations of DQ-02 (gold and yellow sticks) are shown.

From the candidate compound structures with an alkyne moiety (Fig. 1f and Supplementary Fig. 2) as a reference, we chemically synthesized several compounds taking into account their synthetic feasibility. Because most of the suggested compounds have various modifications, and different functional groups were employed even for similar chemical skeletons (Supplementary Fig. 2), we synthesized simple and representative compounds after examining these candidates (Supplementary Figs. 3 and 4, see also Supplementary Data 2 for details), and then evaluated their potency by measuring dose-dependence of ATPase activity inhibition using H^+^,K^+^-ATPase-enriched membrane fractions (Fig. 2a). Among them, although exhibiting lower apparent affinity than SCH28080 (*IC*_50,SCH_ = 1.97 ± 0.12 μM), DQ-02 and DQ-04 inhibit H^+^,K^+^-ATPase activity in a dose-dependent manner with similar apparent affinities (*IC*_50,DQ02_ = 39.9 ± 3.3 μM, *IC*_50,DQ04_ = 33.9 ± 3.8 μM), suggesting that halogen modification to a benzene ring does not make a large difference for the apparent inhibition affinity. Others showed lower apparent affinity than DQ-02 (*IC*_50_s for DQ-09: >2000 μM, DQ-14: >120 μM, DQ-15: 49.5 ± 4.9 μM, DQ-16: 89.4 ± 13.5 μM), and some compounds gave scattered values due to low water solubility. Regardless of the low apparent affinity of DQ-02, its double reciprocal plot analysis (Supplementary Fig. 5) shows a typical competitive inhibition pattern (estimated inhibition constant (*K*_i,DQ02_) is 4.65 μM), suggesting that DQ-02 binds to the luminal-facing cavity as other P-CABs do.

To determine the DQ-02 binding pose, we performed cryo-EM analysis of H^+^,K^+^-ATPase, bound with DQ-02 and obtained a structure at 2.10 Å overall resolution (Fig. 2b, Supplementary Fig. 6 and Table 1). Counterintuitively, however, the EM density map corresponding to DQ-02 was poorly defined, and does not account for a single binding pose of the compound (Fig. 2c and Supplementary Movie 1). We therefore modeled two possible conformations of DQ-02 in the binding cavity. In both binding poses, the terminal secondary amine moiety reaches close to the cation-binding site, as seen in the vonoprazan binding mode. Two benzene rings are likely bound to the Tyr799 and Ala123 similar to SCH28080 and related P-CABs. The structure of the alkyne backbone may be too thin to stably bind in a single conformation to the hydrophobic conduit, and the nearly symmetrical structure of two benzene rings also allows for multiple binding poses of DQ-02. Because alkyne backbone structures were generated using the SCH28080-bound structure as a template, we compared the dimension of its P-CAB binding pocket with another template, the vonoprazan-bound form (Fig. 1f, g). The comparison revealed that the width of the most constricted portion of the luminal conduit in SCH28080-bound form (5.6 Å) is narrower than that in the vonoprazan-bound form (7.2 Å), which may be one reason for the production of the alkyne-connecting candidates by DQ. Given the relatively low affinity and instable binding mode of DQ-02, we halted further improvement of DQ-02 and other alkyne-backbone compounds.

**Table 1:** Cryo-EM data collection, refinement and validation statistics.

	DQ-02 (EMDB-35500) (PDB 8IJV)	DQ-06 (EMDB-35501) (PDB 8IJW)	DQ-18 (EMDB-35502) (PDB 8IJX)	DQ-21 (EMDB-36424) (PDB 8JMN)
Data collection and processing				
Magnification	60,000	60,000	60,000	60,000
Voltage (kV)	300	300	300	300
Electron exposure (e^–^/Å^2^)	60	60	60	60
Defocus range (μm)	0.8–1.8	0.8–1.8	0.8–1.8	0.8–1.8
Pixel size (Å)	0.752	0.580	0.752	0.752
Symmetry imposed	*C1*	*C1*	*C1*	*C1*
Initial particle images (no.)	2,832,257	1,564,257	3,387,269	2,230,909
Final particle images (no.)	354,147	168,758	288,130	599,919
Map resolution (Å)	2.10	2.19	2.08	2.26
FSC threshold	0.143	0.143	0.143	0.143
Refinement				
Initial model used (PDB)	5ylu	5ylu	5ylu	8IJX
Model resolution (Å)	2.16	2.27	2.15	2.30
FSC threshold	0.5	0.5	0.5	0.5
Map sharpening *B* factor (Å^2^)	−49.6	−46.5	−47.1	−79.4
Model composition				
Non-hydrogen atoms	10,533	10,543	10,545	10,491
Protein residues	1251	1251	1251	1251
Waters	370	379	373	373
Ligands	DQ02,Mg^2+^,5PCW,2CLR,3NAG	DQ06,Mg^2+^,5PCW,2CLR,3NAG	DQ18,Mg^2+^,6PCW,CLR,3NAG	DQ21,Mg^2+^,5PCW,CLR,3NAG
*B* factors (Å^2^)				
Protein	49.75	52.98	48.68	94.43
Ligand	54.70	58.78	53.09	84.62
Waters	46.13	44.69	44.86	66.10
R.m.s. deviations				
Bond lengths (Å)	0.003	0.003	0.003	0.003
Bond angles (°)	0.945	0.796	0.778	0.779
Validation				
MolProbity score	1.69	1.78	1.35	1.76
Clashscore	5.79	7.00	4.84	5.76
Poor rotamers (%)	2.92	2.44	1.32	3.29
Ramachandran plot				
Favored (%)	97.9	97.5	98.3	97.8
Allowed (%)	2.1	2.5	1.7	2.2
Disallowed (%)	0.0	0.0	0.0	0.0

### DQ-06: satisfying the pharmacophore of H^+^,K^+^-ATPase Deep Quartet assisted drug design

**Fig. 3:**
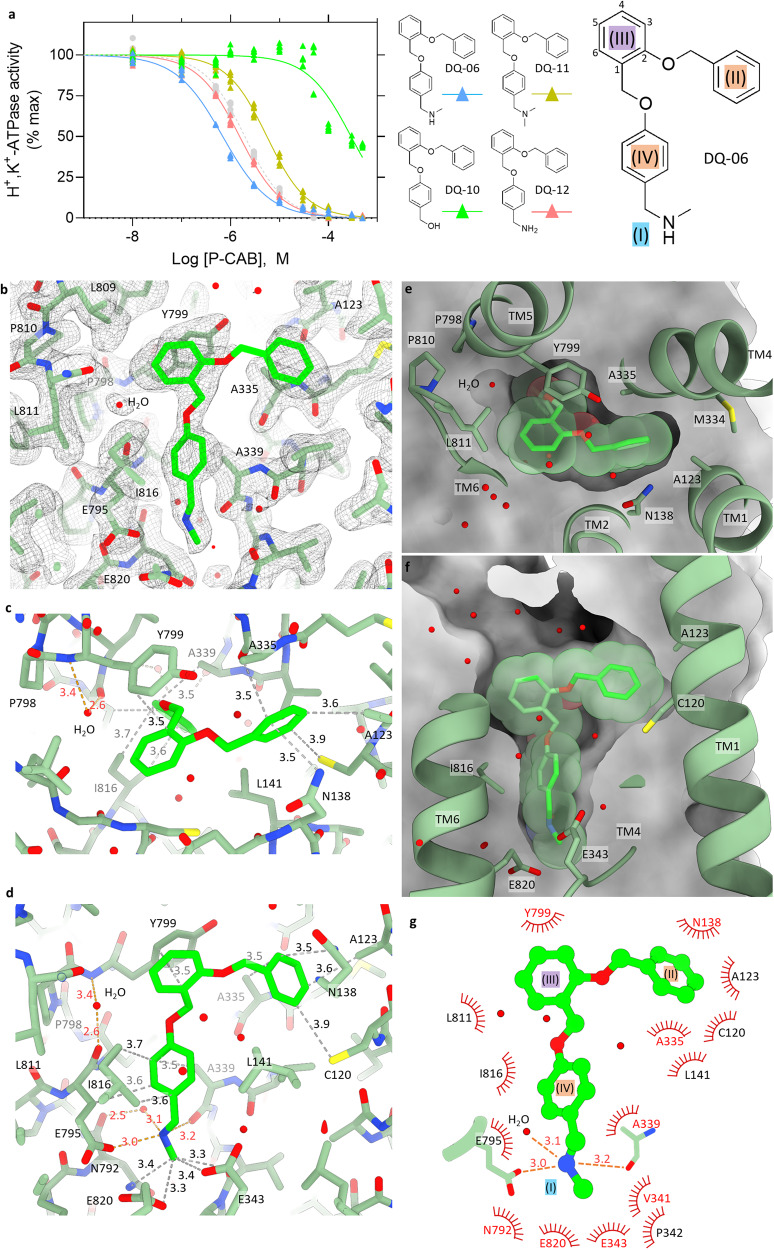
Inhibition potency and the binding pose of DQ-06. **a** Dose-dependent inhibition of H^+^,K^+^-ATPase activity by indicated compounds as shown in Fig. 2a. Data plotted represent each data point from triplicate of three independent measurement at 12 different concentrations of P-CABs. Three benzene rings in DQ-06 are attributed as illustrated in the figure, which corresponds to the defined pharmacophore features (II)–(IV). **b** EM potential map (mesh) and the atomic model of the gastric H^+^,K^+^-ATPase complexed with DQ-06 (green sticks). Shown is a close-up view of the DQ-06 binding site as in Fig. 2c. **c**, **d** Molecular interactions between H^+^,K^+^-ATPase and DQ-06 in stick representation. Hydrophilic and hydrophobic atoms of DQ-06 within 3.5 and 4.0 Å distance from amino acids of H^+^,K^+^-ATPase are connected by orange and gray dotted lines, respectively. Panels are viewed from luminal side (**c**), or parallel to the membrane plane with luminal-side up (**d**). **e**, **f** Clipped cross sections of the DQ-06 binding site from the viewpoints approximately similar to (**c**) and (**d**). Molecular surface (gray) of H^+^,K^+^-ATPase shows the dimension of the binding site in which DQ-06 is accommodated (green stick with transparent van der Waals spheres). Except for TM2, which is removed from the figure for clarity, TM helices and some of the key amino acids are shown in ribbon and stick representations. **g** A schematic 2D representation of DQ-06 binding pose. Hydrophobic residues that are located within 3.9 Å from DQ-06 are shown, and those within 3.5 Å are highlighted as red. Expected polar interactions within 3.5 Å are indicated as orange dotted lines.

In contrast to DQ-02 and other alkyne-backbone compounds, another variety of candidates were suggested by DQ, when using the vonoprazan-bound template structure which has a much wider hydrophobic conduit (Fig. 1g and Supplementary Fig. 2). With reference to these candidates, we designed and synthesized a simple compound that has three benzene rings connected via ether linkers and has a terminal secondary amine moiety (DQ-06, Supplementary Data 2). To our surprise, DQ-06 shows an apparent affinity (*IC*_50,DQ06_ = 0.70 ± 0.04 μM) significantly higher than SCH28080 (Fig. 3a), and its inhibition mode is purely competitive with K^+^ (Supplementary Fig. 5, *K*_i,DQ06_ = 93.8 nM). To evaluate the importance of this terminal secondary amine, systematic modifications were made on the secondary amine moiety of DQ-06, generating DQ-10 ~ 12. Comparison of the inhibition potencies of DQ-06-related compounds, which have a hydroxy (*IC*_50,DQ10_ = 292 ± 67 μM), dimethylamino (*IC*_50,DQ11_ = 5.57 ± 0.45 μM), or amino (*IC*_50,DQ12_ = 1.54 ± 0.07 μM) group, shows clear structure-activity relationships (SAR), indicating that the compounds with a secondary amine moiety showed the best inhibition potency (Fig. 3a).

The binding pose of DQ-06 to the H^+^,K^+^-ATPase was analyzed by using a 2.19 Å resolution cryo-EM structure (Fig. 3b, Supplementary Fig. 6, Supplementary Movie 2 and Table 1). In contrast to the blurred EM density observed for the DQ-02 bound to H^+^,K^+^-ATPase (Fig. 2c), the EM density clearly defines the binding pose of DQ-06, with several bound water molecules visualized. The initial DQ calculation suggests roughly two different binding poses for DQ-06 related candidates (Fig. 1h, i and Supplementary Fig. 2); starting from the cationic secondary amine (pharmacophore feature I), ~60% of DQ-06 related candidates took a binding pose that connected the pharmacophore features in the order (I)→(IV)→(III)→(II) (Fig. 1h), and others took a different binding pose (I)→(IV)→(II)→(III) (Fig. 1i). The binding pose of DQ-06 in the cryo-EM structure is unambiguously determined as a single conformation (Fig. 3b), which agrees with the former binding pose [(I)→(IV)→(III)→(II)] (Fig. 3c, d, g for simplified schematics). The cationic secondary amine reaches the cation-binding site, and is located within hydrogen bond distance to Glu795 side chain oxygen (3.0 Å), main chain carbonyl from the Ala339 located on the unwound portion of TM4 (3.2 Å) and an accompanied water molecule (3.1 Å) (Fig. 3c, d), help fulfill the requirement of “pharmacophore feature I” (Fig. 1e). The SAR of DQ-06-related compounds shows the importance of the methylamino group (Fig. 3a), which is now explained from the structural viewpoint. The terminal methyl group is in van der Waals contact with the surrounding side chains including Glu343 (3.4 Å), Asn792 (3.4 Å) and Glu820 (3.3 Å). The benzene ring III (see Fig. 3a for the nomenclature of the benzene rings in DQ-06) is located close to Tyr799 (3.5 Å) with a nearly parallel relationship, supposedly interacting via their π electron systems (“pharmacophore feature III”). These two portions (pharmacophore features I and III) are connected by the benzene ring IV, which now occupies the space at the hydrophobic conduit (pharmacophore feature IV) surrounded by side chains of Leu141 (4.2 and 4.3 Å), Ala339 (3.5 Å) and Ile816 (3.6 and 3.7 Å) and C_β_ of Glu795 (3.6 Å). The terminal benzene ring II is located near Ala123 (3.6 Å) and making further hydrophobic contacts with Cys120 (3.9 Å), Asn138 (3.4 Å) and Ala335 (3.5 Å), thus fixing its azimuthal position is fixed in a thin, restricted pocket formed between TM1 and TM4 (pharmacophore feature III) (Fig. 3e, f). In contrast to above-described portions that match the defined pharmacophore features, EM density corresponding to the ether linker connecting benzene rings II and III is relatively weak (Fig. 3b), indicating its flexibility, also seen in the SCH28080 binding mode^1,35^.

Because of the simple chemical structure of two benzene rings II and III in DQ-06, which are bound to the luminal entrance of the binding cavity, there is some vacant space in the binding site (Fig. 3e, f). This observation let us consider the possibility that a simple modification in this portion (ring II or III) of the compound may improve the apparent affinity, given that the much tighter binding mode may enhance van der Waals interaction.

### Chloro derivatives of DQ-06

**Fig. 4:**
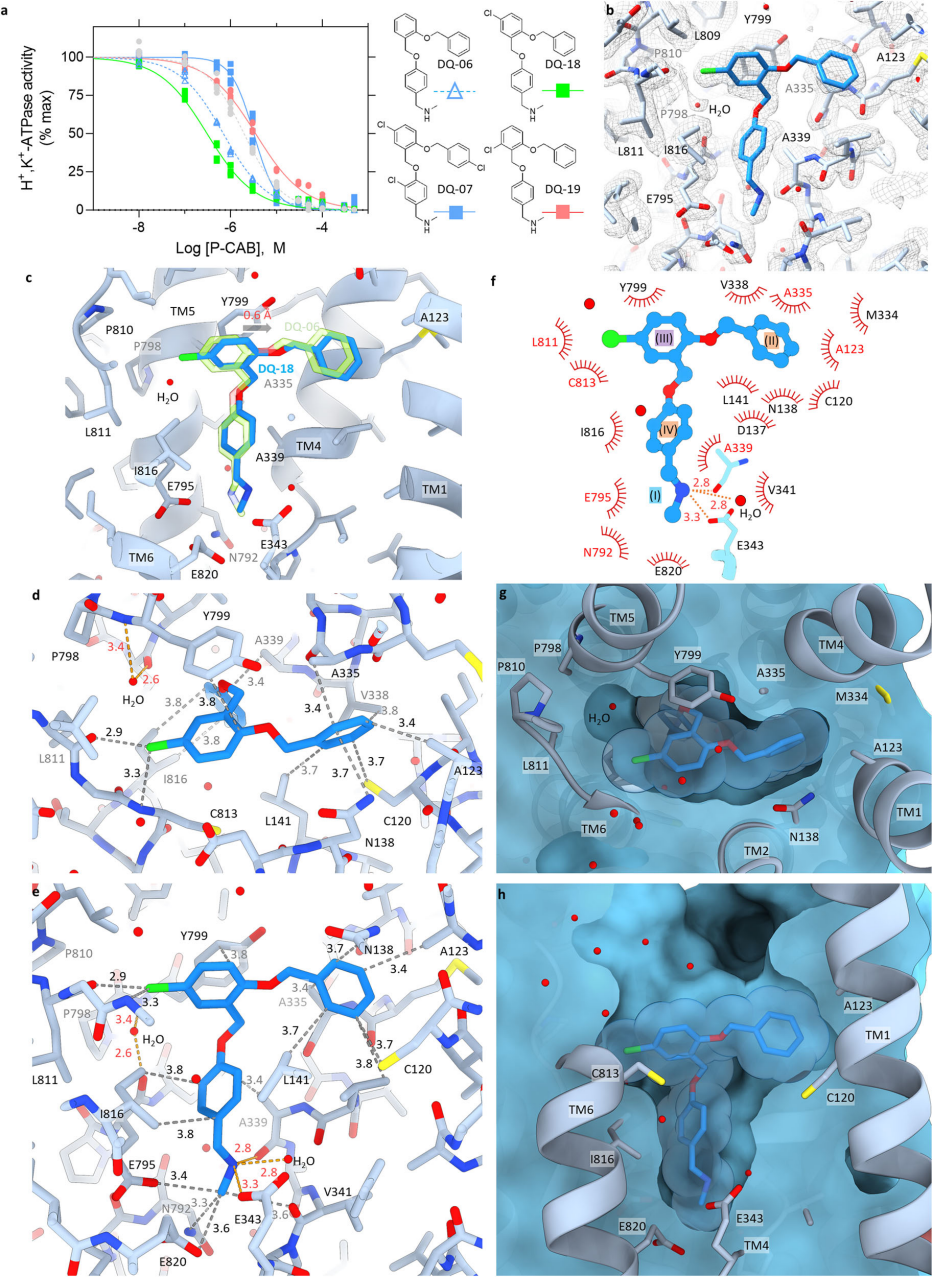
Inhibition potency and the binding pose of DQ-18. **a** Dose-dependent inhibition of H^+^,K^+^-ATPase activity by indicated P-CABs (DQ-06, 07, 18, 19 and SCH28080) as shown in Fig. 2a. Data plotted represent each data point from triplicate of three independent measurement at 12 different concentrations of P-CABs. **b** EM potential map (mesh) and atomic model of the gastric H^+^,K^+^-ATPase complexed with DQ-18 (blue sticks). **c** Comparison of the binding poses between DQ-06 (green) and DQ-18 (blue). Arrow indicates the displacement of the binding position from DQ-06 to DQ-18 (0.6 Å). **d**, **e** Molecular interactions between H^+^,K^+^-ATPase and DQ-18 in stick representation as shown in Fig. 3c, d. **f** A schematic 2D representation of DQ-18 binding pose as shown in Fig. 3g. **g**, **h** Clipped cross sections of the DQ-18 binding site as in Fig. 3e, f.

For further improvement of the binding affinity, we synthesized a series of DQ-06 chloro derivatives (Fig. 4a and Supplementary Data 2). Compared to the original DQ-06, introduction of three chloro groups (DQ-07) significantly reduced its inhibition potency (*IC*_50,DQ07_ = 3.20 ± 0.20 μM). As for compounds with a single modification on ring C, the apparent affinity of 5-chloro derivative (DQ-18) is improved by a factor of two (*IC*_50,DQ18_ = 0.31 ± 0.02 μM) relative to DQ-06, while that of 6-chloro derivative (DQ-19) is significantly reduced (*IC*_50,DQ19_ = 3.52 ± 0.22 μM), indicating that the effect is position-specific (Fig. 4a). We also confirmed the K^+^-competitive inhibition mode of DQ-18 (Supplementary Fig. 5, *K*_i,DQ18_ = 47.6 nM).

The binding pose of DQ-18 was determined by a cryo-EM reconstruction at 2.08 Å resolution (Supplementary Fig. 6 and Table 1), in which the chloro group is clearly seen as a protruded density at the C5 position of benzene ring III (Fig. 4a, b). Due to the modification by the chloro group, the binding position of the terminal (II) and central (III) benzene rings are offset by 0.6 Å compared to DQ-06 (Fig. 4c and Supplementary Movie 3), which gives DQ-18 a much closer contact to the surface of the binding pocket (Fig. 4d–h). The high-resolution EM map unambiguously determined the 5-chloro moiety (Fig. 4b, d, e), which is now in close contact with the main chain oxygen of Leu811 (2.9 Å) presumably interacting via a halogen bond, and the main chain nitrogen of Cys813 (3.3 Å), thus enhanced van der Waals interactions can be expected. Due to the 0.6 Å offset of the binding position of DQ-18 relative to that of DQ-06, the benzene ring II is in much closer contact to amino acids surrounding pharmacophore feature II position near Ala123, and thus the number of amino acids located within 3.9 Å is increased at this position (Fig. 4f, five residues for DQ-06 → eight for DQ-18). In contrast to the relatively large difference in the binding pose between DQ-06 and DQ-18 at the luminal side of the cavity, the effect for cationic secondary amine (I) and connecting benzene ring (IV) is limited (Fig. 4c). Similar to the case for DQ-06, the secondary amine moiety of DQ-18 also reaches the cation-binding site, and is surrounded by three oxygen atoms within 3.5 Å distance. Likewise, the terminal methyl group on the amino group is accommodated with amino acids located at the cation-binding site, including main chain oxygen of Val341 (3.6 Å) and side chains of Asn792 (3.3 Å), Glu795 (3.4 Å) and Glu820 (3.6 Å), within van der Waals distance (Fig. 4e, f). Therefore, based on the high-resolution cryo-EM structure, we conclude the rationale for the improved affinity of DQ-18 to be most likely due to tighter packing of the terminal and central benzene rings (ring II and III) into the binding pocket, enhancing hydrophobic interaction between DQ-18 and H^+^,K^+^-ATPase.

**Fig. 5:**
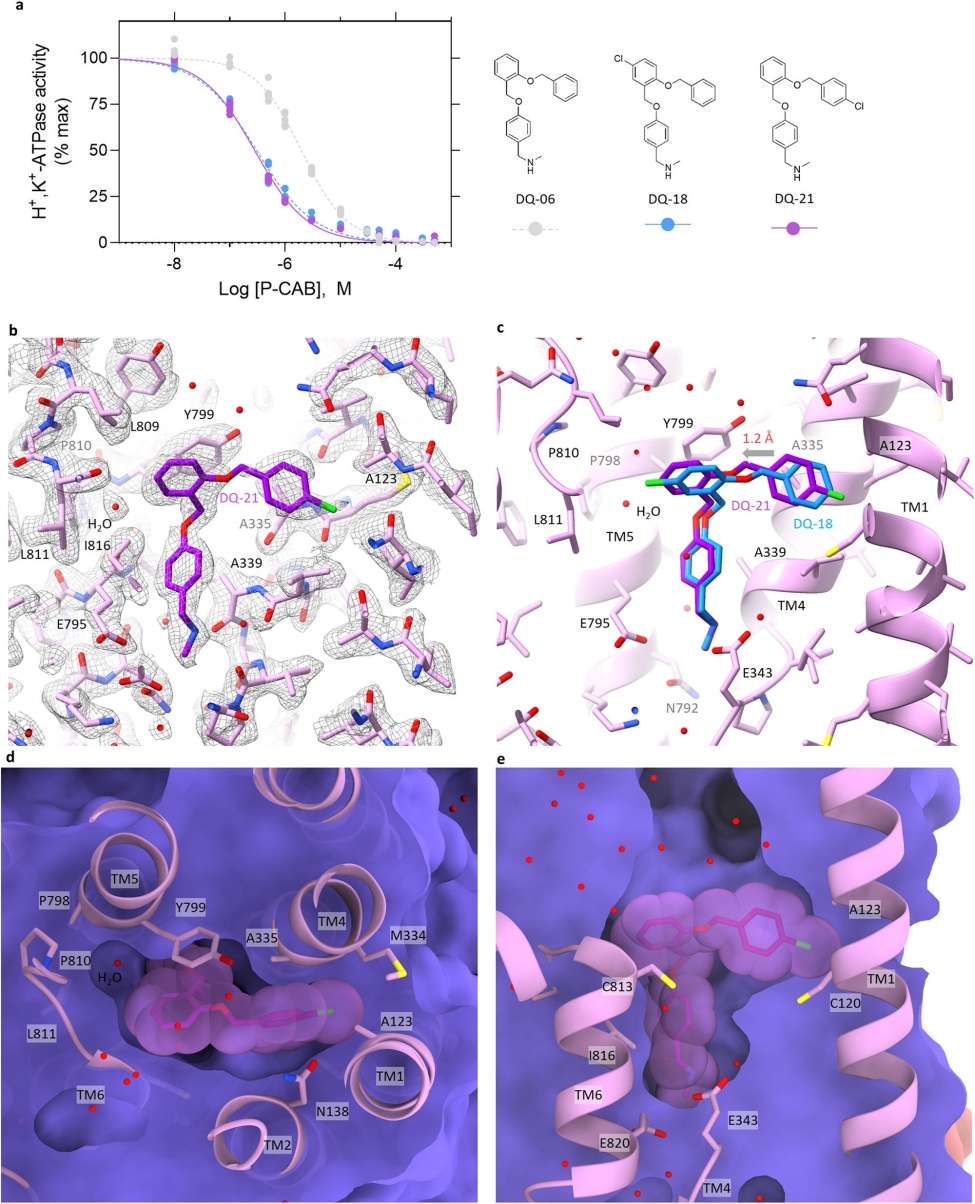
Inhibition potency and the binding pose of DQ-21. **a** Dose-dependent inhibition of H^+^,K^+^-ATPase activity by indicated P-CABs (DQ-06, 18 and 21) as shown in Fig. 2a. Data plotted represent each data point from triplicate of three independent measurement at 12 different concentrations of P-CABs. **b** EM potential map (mesh) and atomic model of the gastric H^+^,K^+^-ATPase complexed with DQ-21 (purple sticks). **c** Comparison of the binding poses between DQ-18 (blue) and DQ-21 (purple). Arrow indicates the displacement of the binding position from DQ-18 to DQ-21 (1.2 Å). **d**, **e** Clipped cross sections of the DQ-21 binding site as in Fig. 3e, f.

This hypothesis is confirmed by structural and functional analysis of DQ-21, a chloro-modified DQ-06 at *para*-position of ring II (Fig. 5 and Supplementary Data 2). DQ-18 and DQ-21 have a chloro group on just opposite sides of ring II and ring III, respectively, and the spatial volume in this part of the compound is the same. Therefore, if the improvement of apparent affinity of DQ-18 would be due to the tighter packing of DQ-18 compared to DQ-06, similar improvement would be observed for DQ-21. As shown in Fig. 5a, DQ-21 shows an apparent affinity (*IC*_50,DQ21_ = 0.28 ± 0.02 μM) similar to that of DQ-18 (*IC*_50,DQ18_ = 0.31 ± 0.02 μM). As we expected, cryo-EM analysis at 2.26 Å reveals that the binding pose of DQ-21 is 1.2 Å offset toward the TM6 side (Fig. 5b, c), and the chloro group at ring II is embedded in the cleft formed between TM1 and TM4, close to Ala123 (Fig. 5d, e). We have thus succeeded in further increasing the affinity of the DQ-suggested compound by its tailored modification based on the high-resolution structural information.

### Potential further improvements of compound binding

High-resolution cryo-EM structures of DQ-06, DQ-18 and DQ-21 bound forms allowed us to visualize several water molecules in their direct vicinity. This includes a water located in the pocket surrounded by Pro798, Pro810 and Leu811, stabilized by the hydrogen-bond network connecting main chain carbonyl of Glu795 and main chain amide of Tyr799 in DQ-06, DQ-18 and DQ-21 bound forms (Figs. 3c–e, 4d, e, g and 5b–d). In the SCH28080-bound structure, this water-filled pocket is occupied with its cyanomethyl group and thus excluded from the binding pocket (c.f., Fig. 1d, see ref. ^2^), which would give a favorable increase in the entropy of the whole system. In fact, an SCH28080 derivative without a cyanomethyl group shows more than 20-times reduced affinity^34^. Given the C6 position of the benzene ring III of DQ-06 (Fig. 3a, c, e) is the closest to the aforementioned water molecule in the pocket, it was expected that the 6-chlorobenzyl derivative (DQ-19) may exclude it, and resulting in an improvement of its apparent affinity. Thus, a question arises as to why DQ-19 does not improve, rather reduces, the binding affinity compared to DQ-06 (Fig. 4a). We speculate that a bulky chlorine modification at C6 position of the ring III may interfere with the oxygen atom of the ether linker connecting two benzene rings III and IV, and prevents the adoption of this particular binding pose. Modification of C6 position of ring III with a smaller atom, or a much longer functional group at C5 position leading to the water-filled pocket, may instead exclude this water molecule. Alternatively, it would be possible to try different compound skeletons; e.g., replacement of the benzene ring III to a 5-membered aromatic ring or a heterocyclic ring like imidazo[1,2-*a*]pyridine of SCH28080 (Supplementary Fig. 1) that could change the direction of the side chain, whilst preserving the π electron donation system bound to Tyr799.

In contrast to the clear EM densities for the most of the functional groups of DQ-06 and DQ-18, the EM densities corresponding to their ether linkers that connecting ring II and III are weak, indicating this portion is mutually flexible for both compounds. This is in good agreement with the conclusion from a previously reported systematic SAR of SCH28080 derivatives with fixed ring analogs (BYK99 and soraprazan, Supplementary Fig. 1)^35^, which show more than 25 and 7 times higher apparent affinity than SCH28080, respectively^1,34^. We also reported that the dihedral angle at the connecting oxygen atom of SCH28080 in solution distributed three metastable positions in the molecular dynamics simulation, indicating its conformational freedom^1^. Such flexibility in the relative orientation of two benzene rings II and III connected by an ether linker is also expected for the DQ-06-related compounds. By fixing the linker structure and thereby restricting the conformation that DQ-06-related compounds can adopt, a dramatic improvement in their apparent affinities could be anticipated. However, we cannot exclude the possibility that the flexibility of the linker region helps DQ-06 and DQ-18 binding, as chemical backbone of these compounds is different from that of SCH28080.

In this manuscript, all the ATPase measurements for the determination of apparent affinities of the compounds were performed at neutral (pH 7) condition. In the case of SCH28080, because of its p*K*_a_ value of 5.6 for imidazo[1,2-a]pyridine amine, its apparent affinity differs around neutral (*K*_i_ = 66 nM at pH 7.35) and weakly acidic (*K*_i_ = 20 nM at pH 6.11) conditions^36^. However, expected p*K*_a_ value of secondary amine groups of DQ-related compounds are in the range of 9-11, indicating they are mostly protonated at neutral pH, and also at highly acidic condition in the stomach. According to Henderson-Hasselbalch equation, ratio of protonated : free amine is expected to be 1:25 at pH 7 and 1:10 at pH 6.6 for SCH28080 with p*K*_a_ of 5.6. In contrast, it is expected to be 100:1 ~ 10,000:1 for the secondary amine of DQ-related compounds with p*K*_a_ of 9–11, and this ratio does not largely change around neutral (pH 7), weakly acidic (for example, pH 6.0) and even strongly acidic condition in the stomach, thus the apparent affinities of DQ-related compounds unlikely affected by the in vitro solution pH.

### A unique framework for de novo drug generation

Here, a combination of AI-driven compound design, chemical synthesis and cryo-EM analysis of drug bound structures underpinned the development of P-CAB candidates that have de novo chemical structures (Fig. 1a). Recent advances in structural analysis by single particle cryo-EM^37,38^ renders drug-bound protein structures significantly more accessible and reliable than previously. This enables the determination of pharmacophores, relevant structure-based modifications, and iterative repetition of these cycles to improve affinity, potencies and other pharmacokinetic parameters. To address unforeseen side effects and expanding medical needs, the development of alternative drugs with distinct chemical skeletons is highly desired. However, when creating new compounds based on the original drug-bound structure, it is often difficult to generate a truly different chemical skeleton derived far from the original drug structures. As highlighted in our case, even if several pharmacophore features have been determined on the protein structure, it is not easy to manually create the optimal chemical structure to link them with. Deep Quartet, a deep learning based de novo design workflow, generates chemical structures that satisfy the desired pharmacophores on the protein structure^29,30^. Since the software “learns” chemical structures from the database ChEMBL^39^ (https://www.ebi.ac.uk/chembl/) in which more than 2-million bioactive molecules are stored, generated candidates have mostly “drug-like” chemical structures (Fig. 1a and Supplementary Fig. 2). However, in some cases DQ generates too many candidate compounds, and some of them are not trivial to synthesize, or, compounds themselves are instable in aqueous solution. We therefore selected candidates “heuristically”, based on the knowledge of organic chemistry and previous functional analysis of H^+^,K^+^-ATPase with P-CABs, and started with simple compounds, like DQ-02 (Fig. 2) or DQ-06 (Fig. 3), for streamlined structure-activity studies. This strategy may be particularly useful to determine the direction of which compounds are promising in the very early stages of drug development.

Pharmacophore-based de novo design of the chemical structure may also be applied for the replacement of large molecular weight drugs such as polypeptides, nucleic acids and antibodies by small molecule compounds. Recent technological innovations enabled the identification of high-affinity cyclic peptides through DNA-encoded library screening^40^. Many antibody-based medicines are also available for the clinical treatment. These large molecules often suffer from pharmacokinetic issues including their low permeability to the cell membrane or expensive production cost. When the target structures complexed with these large molecules are available, our strategy may offer a promising option to develop small compounds that satisfy the greatest common denominator of pharmacophores on the protein structure.

“Deep Quartet” (Fig. 1a) consists of a series of flows including (1) deep reinforcement learning, (2) LigandScout, a software using pharmacophore models, and (3) Sub-structure filtering to select candidates that match the desired target. In addition to (1)–(3), (4) knowledge of medicinal or organic chemistry, is also included in the flow, to create an AI-platform for the drug discovery achieved by the “quartet” (Fig. 1a). Now Deep Quartet has been added a fifth flow (5) high-resolution cryo-EM structural analysis. Thus, this platform now is “Deep Quintet”, providing a distinct and more powerful framework for the drug discovery.

## Methods

### De novo drug design by Deep Quartet

Deep Quartet (DQ) is a workflow for generating chemical structures with desired pharmacophore (Fig. 1a)^29,30^. REINVENT^41^, which is an open-source Python application, is used as DGMs in DQ. The DGMs consist of two recurrent neural network models named as Prior and Agent networks. The Prior network is trained using SMILES representation of molecules obtained from ChEMBL^39^. The Agent network is initialized by Prior network and then trained using reinforcement learning^41^. During the training, the probability distribution of the Agent network shifts toward a distribution modulated by a scoring function. After the training, Agent network generates SMILES with a desired property obtained from a scoring function. In this study, to generate diverse chemical structure, two approaches, early stop & refresh and tabu list, were implemented in REINVENT. In early stop & refresh, when averaged scores exceeded a pre-defined score (set to 0.8) threshold or training steps exceed pre-defined steps (set to 1000) during the training, the training was stopped. Then, the Agent network was initialized and the training was re-started. This process was done up to a pre-defined number of total training steps. The top 50 scoring structures generated from each Agent network were stored. In tabu list approach, scaffolds of the structures generated from each Agent network are appended to the tabu list. The scaffolds were calculated using MurckoScaffold function implemented in RDKit (https://www.rdkit.org)^42^. When the Agent network generated a chemical structure with the same scaffold as the scaffolds included in the tabu list, the score of the compound was set to zero. The tabu list was updated each time the Agent network was initialized. In REINVENT, training of the Agent network was done with sigma (a parameter related to learning rate) of 120, set to experience_replay and total training steps of 10,000. All other parameters were set to default values and pre-trained Prior network provided from REINVENT is used. To generate chemical structure with desired pharmacophore, the Agent networks were trained using the scoring function (Relative Pharmacophore-Fit) of LigandScout 4.4^43^. The Relative Pharmacophore-Fit outputs a pharmacophore score that is normalized to [0, 1] range based on the number of matching pharmacophore features and the RMSD of the pharmacophore alignment. Based on the gastric proton pump complexed with its blockers derived from X-ray crystallography (PDB ID: 5ylv and 5ylu), three different types of pharmacophore models (Type A, B, and C) were defined as shown in Supplementary Fig. 2. A DQ run using Type A pharmacophore generated 550 chemical structures. Among them, compounds with methylamino group and pharmacophore score of 0.9 or higher were selected, and the resulting 71 compounds were considered as candidates for synthesis. In the same manner, a DQ run using Type B pharmacophore generated 600 chemical structures from which 10 compounds were considered as candidates for synthesis. A DQ run using Type C pharmacophore generated 650 chemical structures where 181 compounds were considered as candidates for synthesis.

### Selection and synthesis of candidate compounds

DQ-02, DQ-04 (Type A) and DQ-07 (Type C) were selected from the list of the above candidates. The structures of DQ-06 (Type C) and DQ-09 (Type A) were generated by removing halogen substituents of the compounds in the list. The structure of DQ-10, DQ-11 or DQ-12 were systematically generated by replacing the methylamino group of DQ-06 with a hydroxy, dimethylamino or amino group, respectively. The structures of DQ-18, DQ-19 and DQ-21 were designed by adding a chloro group onto DQ-06. The list generated by using Type B pharmacophore included bicyclic heteroaromatic compounds having two alkyne units. Based on these structural features, DQ-14, DQ-15 and DQ-16 were newly designed from the viewpoint of synthetic accessibility, and were evaluated by the pharmacophore scores before the synthesis. All the compounds were synthesized according to the synthetic procedures provided in the Supplementary Data 2.

### ATPase activity measurement

H^+^,K^+^-ATPase-enriched membrane fractions were purified from pig stomach according to the previously reported procedures^44^. These membrane fractions (~0.05 μg protein/40 μl solution in each well) were suspended in buffer containing 40 mM PIPES/Tris (pH 7.0), 2 mM MgCl_2_, 2 mM ATP di-tris salt and 10 mM KCl in the presence of different concentrations of SCH28080 or synthesized compounds in 96-well microtubes^34^. Reactions were initiated by incubating the fractions at 37 °C using a thermal cycler and maintained for 1 h. Reactions were terminated by adding 2 M HCl, and the amount of released inorganic phosphate was determined colorimetrically^45^ using a microplate reader (TECAN). The specific H^+^,K^+^-ATPase activity was calculated by subtracting the activities in the presence of 0.5 mM SCH28080. The *IC*_50_ value was estimated by the sigmoidal curve fitting using software PRISM 9.

Data measured from 96-well plate contained triplicates of four different sets of K^+^-dependent ATPase assays in the absence or presence of three different concentrations of synthesized compounds. Data were corrected for background values in the absence of K^+^ at each compound concentration, and plot them against the double reciprocal axes in Supplementary Fig. 5. Data were also fitted by using simultaneous nonlinear regression as described previously to estimate their *K*_i_ values^34^.

Raw data for the ATPase measurement can be found in Supplementary Data 1.

### Expression and purification of recombinant H^+^,K^+^-ATPase

Procedures for protein expression are essentially the same as those reported previously^2,46^. Briefly, Flag epitope tag (DYKDDDDK), a hexa-histidine tag and the enhanced green fluorescence protein (EGFP) were inserted in the amino terminal side of Met48 of the pig gastric H^+^,K^+^-ATPase α-subunit, followed by a tobacco etch virus (TEV) protease recognition sequence and subcloned into a hand-made vector^2^. The wild type pig gastric H^+^,K^+^-ATPase β-subunit was also cloned. The αβ-complex of H^+^,K^+^-ATPase were expressed in the plasma membrane using baculovirus-mediated transduction of mammalian HEK293S GnT1^-^ cells (BacMam)^47^ purchased from ATCC.

For cryo-EM analysis, cells were directly solubilized with 1% lauryl maltose neopentyl glycol (LMNG)^48^ in the presence of 40 mM MES/Tris (pH 6.5), 10% glycerol, 5 mM dithiothreitol, 1 mM MgCl_2_, in the presence of 1 mM BeSO_4_, 3 mM NaF and protease inhibitor cocktail (Roche) on ice for 20 min. After removing insoluble material by ultracentrifugation, the supernatant was mixed with anti-GFP nanobody resin^49^ at 4 °C for 2 h, which was followed by washing with buffer containing 40 mM MES/Tris (pH 6.5), 5% glycerol, 1 mM MgCl_2_, 1 mM BeSO_4_, 3 mM NaF, 50 mM NaCl and 0.06% glyco-diosgenin (GDN)^50^. After addition of TEV protease and endoglycosidase, anti-GFP nanobody was incubated at 4 °C overnight. Digested peptide fragments containing EGFP and endoglycosidase were removed by passing the fractions through Ni-NTA resin (Qiagen). Flow-through fractions were concentrated and subjected to size-exclusion column chromatography using a Superose6 Increase column (Cytiva) equilibrated in buffer comprising 20 mM MES/Tris (pH 6.5), 1 mM MgCl_2_, 1 mM BeSO_4_, 3 mM NaF, 50 mM NaCl and 0.06% GDN. Peak fractions were collected and concentrated to 8 mg/ml. A final concentration of 0.1 mM synthesized compound (DQ-02, DQ-06 or DQ-18) was added to the protein sample.

### Cryo-EM structural analysis

Preparation of sample and cryo-EM grids were done according to a previous report^1,51^. The purified protein samples (at 8 mg/ml) containing 0.1 mM synthesized compound were applied to a freshly glow-discharged Quantifoil holey carbon grids (R1.2/1.3, Cu/Rh, 200 mesh), using a Vitrobot Mark IV (Thermo Fisher) at 4 °C with a blotting time of 4 s under 99% humidity, and then plunge-frozen in liquid ethane. The prepared grids were transferred to a CRYO ARM 300 microscope (JEOL), operated at 300 kV, with a cold-field emission gun as the electron source, an in-column Ω filter and equipped with a Gatan K3 direct electron detector in the electron counting mode. Imaging was performed at a nominal magnification of ×60,000 for DQ-02 and DQ-18 bound forms, and ×80,000 for DQ-06 bound one, corresponding to a calibrated pixel size of 0.753 and 0.580 Å/pix, respectively (EM01CT at SPring-8). Each movie was recorded in correlated-double sampling (CDS) mode for 2.6 s and subdivided into 60 frames. The electron flux was set to 8.46 e^−^/pix/s at the detector, resulting in an accumulated exposure of 60 e^−^/Å^2^ at the specimen. The data were automatically acquired by the image shift method using SerialEM software^52^, with a defocus range of −0.8 to −1.8 μm. The dose-fractionated movies were subjected to beam-induced motion correction, using RELION 3.1^53^, and the contrast transfer function (CTF) parameters were estimated using patch CTF estimation in cryoSPARC (v4, Structura Biotechnology)^54^.

For each dataset, particles were initially picked by blob picker using cryoSPARC (v4), and extracted with down-sampling to a pixel size of 3.24 Å/pix. These particles were subjected to several rounds of 2D classifications. Good-looking classes were then subjected to ab initio reconstruction in three models, and refined by non-uniform refinement^55^. The particles from the best class was then re-extracted to the full pixel size and subjected to non-uniform refinement with per-particle defocus refinement, beam-tilt refinement in cryoSPARC (v4). The particle stack was then transferred to RELION 3.1, and subjected to Bayesian polishing^56^. Polished particles were re-imported to cryoSPARC (v4), and subjected to non-uniform refinement. The resolution of the analyzed map was defined according to the FSC = 0.143 criterion^57^ (Supplementary Fig. 6). The local resolution and angular distributions for each structure were estimated by cryoSPARC (v4). All the models were manually built in Coot^58^ using the crystal structure of SCH28080-bound H^+^,K^+^-ATPase (5ylu) as a starting template^2^. Phenix^59^ (version 20) was used for refinement.

### Reporting summary

Further information on research design is available in the Nature Portfolio Reporting Summary linked to this article.

### Supplementary information

Peer Review File

Supplementary Figures

Description of Additional Supplementary Files

Supplementary Data 1

Supplementary Data 2

Supplementary Movie 1

Supplementary Movie 2

Supplementary Movie 3

Supplementary Software

Reporting Summary

## Data Availability

The structural data generated in this study have been deposited in the Protein Data Bank and EM Data Bank under accession codes 8IJV and EMD-35500; Cryo-EM structure of the gastric proton pump with bound DQ-02, 8IJW and EMD-35501; Cryo-EM structure of the gastric proton pump with bound DQ-06, 8IJX and EMD-35502; Cryo-EM structure of the gastric proton pump with bound DQ-18; Cryo-EM structure of the gastric proton pump with bound DQ-21, 8JMN and EMD-36424. Source data are provided as Supplementary Data.

## Code availability

Deep Quartet is a software developed by Intage Healthcare Inc., is provided as Supplementary_Software.zip.

## References

[1] Tanaka S (2022). Structural basis for binding of potassium-competitive acid blockers to the gastric proton pump. J. Med. Chem..

[2] Abe K, Irie K, Nakanishi H, Suzuki H, Fujiyoshi Y (2018). Crystal structures of the gastric proton pump. Nature.

[3] Shin JM, Sachs G (2008). Pharmacology of proton pump inhibitors. Curr. Gastroenterol. Rep..

[4] Wallace JL (2000). How do NSAIDs cause ulcer disease?. Best Pract. Res. Clin. Gastroenterol..

[5] Hain E, Coriat R, Dousset B, Gaujoux S (2016). Prise en charge du gastrinome. Presse Med..

[6] Black JW, Duncan WAM, Durant CJ, Ganellin CR, Parsons EM (1972). Definition and antagonism of histamine H2-receptors. Nature.

[7] Wroblewski LE, Peek RM, Wilson KT (2010). Helicobacter pylori and gastric cancer: factors that modulate disease risk. Clin. Microbiol. Rev..

[8] Ganser AL, Forte JG (1973). K+-stimulated ATPase in purified microsomes of bullfrog oxyntic cells. BBA Biomembr..

[9] Chang H, Saccomani G, Rabon E, Schackmann R, Sachs G (1977). Proton transport by gastric membrane vesicles. BBA Biomembr..

[10] Palmgren MG, Axelsen KB (1998). Evolution of P-type ATPases. Biochim. Biophys. Acta Bioenerg..

[11] Toyoshima C, Nakasako M, Nomura H, Ogawa H (2000). Crystal structure of the calcium pump of sarcoplasmic reticulum Ê resolution. Nature.

[12] Post RL, Kume S, Tobin T, Orcutt B, Sen AK (1969). Flexibility of an active center in sodium-plus-potassium adenosine triphosphatase. J. Gen. Physiol..

[13] Rabon EC, Reuben MA (1990). The mechanism and structure of the gastric H,K-ATPase. Annu. Rev. Physiol..

[14] Vagin O, Turdikulova S, Tokhtaeva E (2007). Polarized membrane distribution of potassium-dependent ion pumps in epithelial cells: different roles of the N-glycans of their beta subunits. Cell Biochem. Biophys..

[15] Shin JM, Cho YM, Sachs G (2004). Chemistry of covalent inhibition of the gastric (H+, K +)-ATPase by proton pump inhibitors. J. Am. Chem. Soc..

[16] Hersey SJ, Steiner L, Mendlein J, Rabon E, Sachs G (1988). SCH28080 prevents omeprazole inhibition of the gastric H + / K +-ATPase. Biochim. Biophys. Acta.

[17] Wallmark B (1987). Inhibition of gastric H+,K+-ATPase and acid secretion by SCH 28080, a substituted pyridyl(1,2a)imidazole. J. Biol. Chem..

[18] Kim DK (2019). Effects of tegoprazan, a novel potassium-competitive acid blocker, on rat models of gastric acid-related disease. J. Pharmacol. Exp. Ther..

[19] Han KS, Kim YG, Yoo JK, Lee JW, Lee MG (1998). Pharmacokinetics of a new reversible proton pump inhibitor, YH1885, after intravenous and oral administrations to rats and dogs: hepatic first-pass effect in rats. Biopharm. Drug Dispos..

[20] Otake K (2016). Characteristics of the novel potassium-competitive acid blocker vonoprazan fumarate (TAK-438). Adv. Ther..

[21] Nagata N (2022). Population-level metagenomics uncovers distinct effects of multiple medications on the human gut microbiome. Gastroenterology.

[22] Nochaiwong S (2018). The association between proton pump inhibitor use and the risk of adverse kidney outcomes: a systematic review and meta-analysis. Nephrol. Dial. Transplant..

[23] Hagiwara T, Mukaisho K-I, Nakayama T, Sugihara H, Hattori T (2011). Long-term proton pump inhibitor administration worsens atrophic corpus gastritis and promotes adenocarcinoma development in Mongolian gerbils infected with Helicobacter pylori. Gut.

[24] Abe K, Yamamoto K, Irie K, Nishizawa T, Oshima A (2021). Gastric proton pump with two occluded K+ engineered with sodium pump-mimetic mutations. Nat. Commun..

[25] Huang, H., Yu, P. S. & Wang, C. An introduction to image synthesis with generative adversarial nets. 10.48550/arxiv.1803.04469 (2018).

[26] Johnson M (2017). Google’s multilingual neural machine translation system: enabling zero-shot translation. Trans. Assoc. Comput. Linguist..

[27] Bilodeau C, Jin W, Jaakkola T, Barzilay R, Jensen KF (2022). Generative models for molecular discovery: recent advances and challenges. WIREs Comput. Mol. Sci..

[28] Zhavoronkov A (2019). Deep learning enables rapid identification of potent DDR1 kinase inhibitors. Nat. Biotechnol..

[29] Yoshimori A, Kawasaki E, Kanai C, Tasaka T (2020). Strategies for design of molecular structures with a desired pharmacophore using deep reinforcement learning. Chem. Pharm. Bull..

[30] Yoshimori A (2021). Design and synthesis of DDR1 inhibitors with a desired pharmacophore using deep generative models. ChemMedChem.

[31] Bickerton GR, Paolini GV, Besnard J, Muresan S, Hopkins AL (2012). Quantifying the chemical beauty of drugs. Nat. Chem..

[32] Ertl P, Schuffenhauer A (2009). Estimation of synthetic accessibility score of drug-like molecules based on molecular complexity and fragment contributions. J. Cheminform..

[33] Asano S, Io T, Kimura T, Sakamoto S, Takeguchi N (2001). Alanine-scanning mutagenesis of the sixth transmembrane segment of gastric H+,K+-ATPase α-subunit. J. Biol. Chem..

[34] Abe K (2017). The cryo-EM structure of gastric H^+^,K^+^-ATPase with bound BYK99, a high-affinity member of K^+^-competitive, imidazo[1,2-a]pyridine inhibitors. Sci. Rep..

[35] Kaminski JJ, Wallmark B, Briving C, Andersson BM (1991). Antiulcer agents. 5. Inhibition of gastric H+/K(+)-ATPase by substituted imidazo[1,2-a]pyridines and related analogues and its implication in modeling the high affinity potassium ion binding site of the gastric proton pump enzyme. J. Med. Chem..

[36] Keeling DJ, Laing SM, Senn-Bilfinger J (1988). SCH 28080 is a lumenally acting, K+-site inhibitor of the gastric (H+ + K+)-ATPase. Biochem. Pharmacol..

[37] Maki-Yonekura S, Hamaguchi T, Naitow H, Takaba K, Yonekura K (2021). Advances in cryo-EM and ED with a cold-field emission beam and energy filtration—refinements of the CRYO ARM 300 system in RIKEN SPring-8 center—. Microscopy.

[38] Nakane T (2020). Single-particle cryo-EM at atomic resolution. Nature.

[39] Gaulton A (2012). ChEMBL: a large-scale bioactivity database for drug discovery. Nucleic Acids Res..

[40] Yoshida S (2022). Peptide-to-small molecule: a pharmacophore-guided small molecule lead generation strategy from high-affinity macrocyclic peptides. J. Med. Chem..

[41] Olivecrona M, Blaschke T, Engkvist O, Chen H (2017). Molecular de-novo design through deep reinforcement learning. J. Cheminform..

[42] Bemis GW, Murcko MA (1996). The properties of known drugs. 1. Molecular frameworks. J. Med. Chem..

[43] Wolber G, Dornhofer AA, Langer T (2006). Efficient overlay of small organic molecules using 3D pharmacophores. J. Comput. Aided Mol. Des..

[44] Abe K, Olesen C (2016). Isolation of H^+^,K^+^-ATPase-enriched membrane fraction from pig stomachs. Methods Mol. Biol..

[45] Chifflet S, Torriglia A, Chiesa R, Tolosa S (1988). A method for the determination of inorganic phosphate in the presence of labile organic phosphate and high concentrations of protein: application to lens ATPases. Anal. Biochem..

[46] Young VC (2022). Structure and function of H+/K+ pump mutants reveal Na+/K+ pump mechanisms. Nat. Commun..

[47] Dukkipati A, Park HH, Waghray D, Fischer S, Garcia KC (2008). BacMam system for high-level expression of recombinant soluble and membrane glycoproteins for structural studies. Protein Expr. Purif..

[48] Chae PS (2010). Maltose–neopentyl glycol (MNG) amphiphiles for solubilization, stabilization and crystallization of membrane proteins. Nat. Methods.

[49] Kubala MH, Kovtun O, Alexandrov K, Collins BM (2010). Structural and thermodynamic analysis of the GFP:GFP-nanobody complex. Protein Sci..

[50] Chae PS (2012). A new class of amphiphiles bearing rigid hydrophobic groups for solubilization and stabilization of membrane proteins. Chem. Eur. J..

[51] Nakanishi H (2020). Transport cycle of plasma membrane flippase ATP11C by Cryo-EM. Cell Rep..

[52] Mastronarde DN (2005). Automated electron microscope tomography using robust prediction of specimen movements. J. Struct. Biol..

[53] Zivanov J (2018). New tools for automated high-resolution cryo-EM structure determination in RELION-3. Elife.

[54] Punjani A, Rubinstein JL, Fleet DJ, Brubaker MA (2017). cryoSPARC: algorithms for rapid unsupervised cryo-EM structure determination. Nat. Methods.

[55] Punjani A, Zhang H, Fleet DJ (2020). Non-uniform refinement: adaptive regularization improves single-particle cryo-EM reconstruction. Nat. Methods.

[56] Zivanov J, Nakane T, Scheres SHW (2019). A Bayesian approach to beam-induced motion correction in cryo-EM single-particle analysis. IUCrJ.

[57] Rosenthal PB, Henderson R (2003). Optimal determination of particle orientation, absolute hand, and contrast loss in single-particle electron cryomicroscopy. J. Mol. Biol..

[58] Emsley P (2004). *Coot*: model-building tools for molecular graphics. Acta Crystallogr. Sect. D Biol. Crystallogr..

[59] Adams PD (2010). PHENIX: a comprehensive Python-based system for macromolecular structure solution. Acta Crystallogr. Sect. D Biol. Crystallogr..

