# Supplementary material for: Deep learning driven de novo drug design based on gastric proton pump structures
Source: Commun Biol. 2023 Sep 19;6:956. doi: 10.1038/s42003-023-05334-8 (PMC10509173; doi:10.1038/s42003-023-05334-8)
Supplement: Supplementary file 2 — Supplementary Figures [file 42003_2023_5334_MOESM2_ESM.pdf]

Supplementary Information for

Deep learning driven *de novo* drug design based on gastric proton pump structures

Kazuhiro Abe[1,2,3]*, Mami Ozako[2], Miki Inukai[2], Youe Matsuyuki[2], Shinnosuke Kitayama[2], Chisato Kanai[4], Chiaki Nagai[2], Chai C. Gopalasingam[5], Christoph Gerle[5], Hideki Shigematsu[6], Nariyoshi Umekubo[2], Satoshi Yokoshima[2]* & Atsushi Yoshimori[7]*

[1]Cellular and Structural Physiology Institute, Nagoya University, Nagoya, Aichi, Japan

[2]Graduate School of Pharmaceutical Sciences, Nagoya University, Nagoya, Aichi, Japan

[3]Center for One Medicine Innovative Translational Research, Gifu University Institute for Advanced Study, Gifu, Japan

[4]INTAGE Healthcare, Inc., 3-5-7, Kawaramachi Chuo-ku, Osaka 541-0048, Japan

[5]RIKEN Spring-8 Center, Kouto, Sayo-gun, Hyogo 679-5148, Japan

[6]Japan Synchrotron Radiation Research Institute (JASRI), SPring-8, 1-1-1 Kouto, Sayo, Hyogo 679-5148, Japan

[7]Institute for Theoretical Medicine, Inc., 26-1, Muraoka-Higashi 2-chome, Fujisawa, Kanagawa, 251-0012, Japan

*Correspondence to:

kabe@cespi.nagoya-u.ac.jp (K.A.)

yokosima@ps.nagoya-u.ac.jp (S.Y.)

yoshimori@itmol.com (A.Y.)

Contents

Supplementary Figures 1-6

SCH28080-related P-CABs

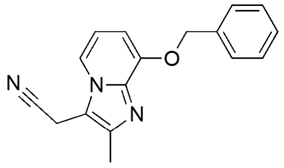

SCH28080 (1.97 ± 0.12 μM)

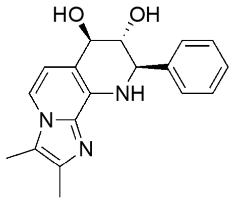

BYK99 (0.077 μM)[26]

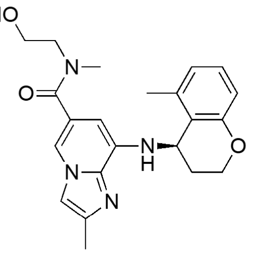

PF-03716556 (6.5 μM)[1]

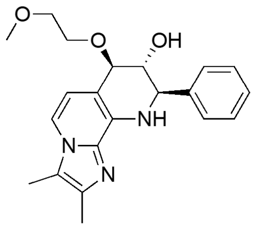

Soraprazan (0.30 μM)[1]

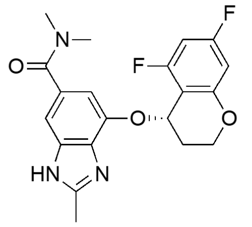

Tegoprazan (2.3 μM)[1]

P-CABs with unique skeleton

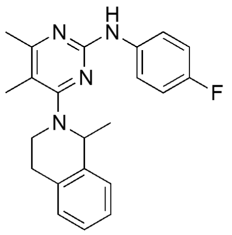

Revaprazan (2.3 μM)[1]

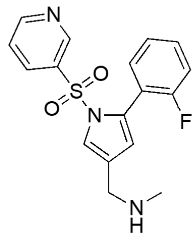

Vonoprazan (0.0015 μM)[1]

Supplementary Figure 1| Chemical structures of P-CABs and related compounds
Chemical structures of SCH28080-related P-CABs (upper panel) and those with unique skeleton. Their IC_{50} values (in the presence of 10 mM KCl) described in references[1,26] are indicated in the parenthesis.

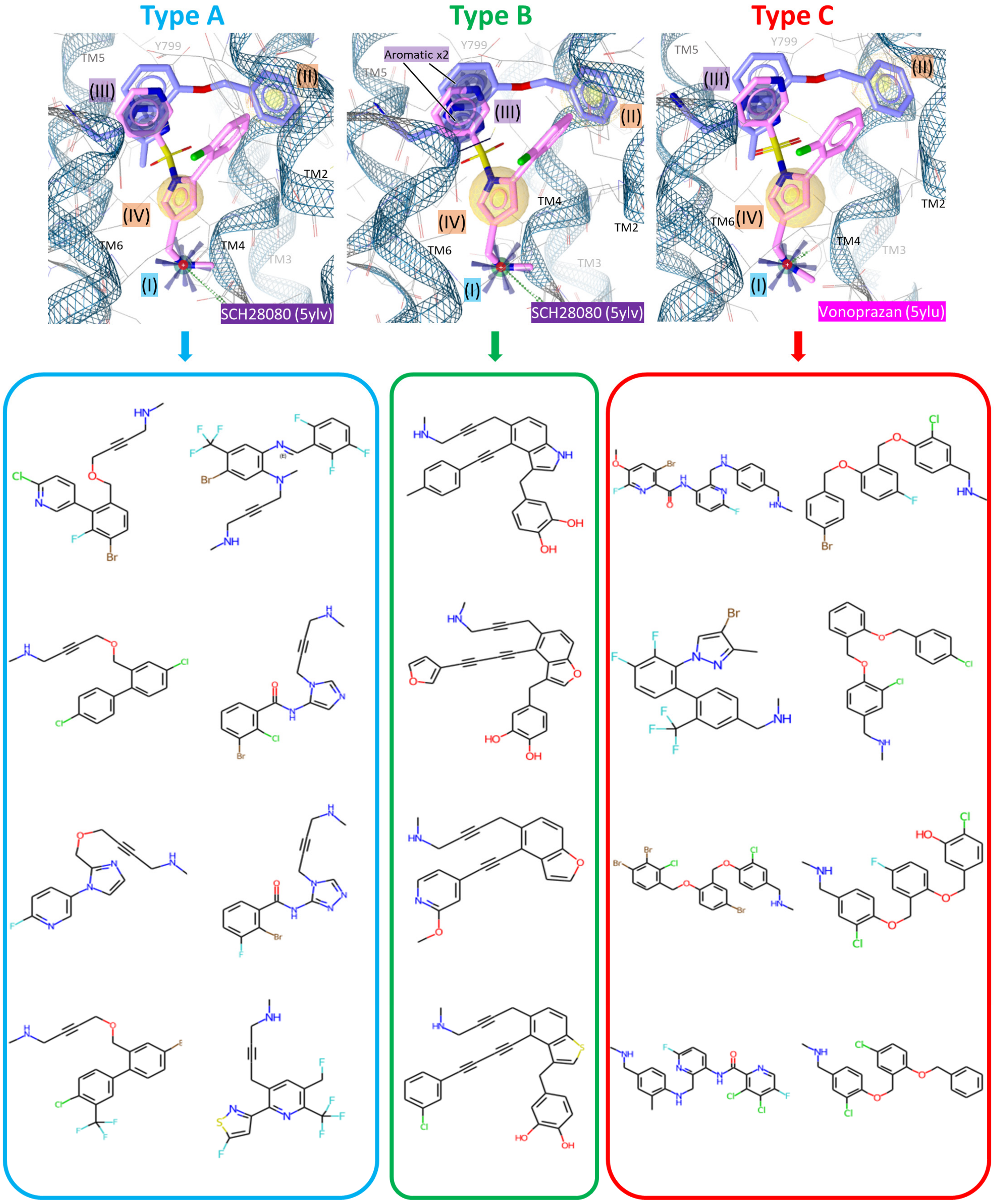

Supplementary Figure 2| Pharmacophore settings and candidate compounds
Defined pharmacophores for three different models (Type A – C) were shown (upper row). Note two adjacent aromatic pharmacophore features are defined in the "pharmacophore feature III" (refer to Figure 3) position where imidazo[1,2-*a*]pyridine ring of SCH28080 is located in Type B setting, while others set a single aromatic pharmacophore feature at this position. Chemical structures of representative compounds generated from corresponding pharmacophore settings (lower row). Only part of candidates related to the compounds evaluated in this study were shown for clarity.

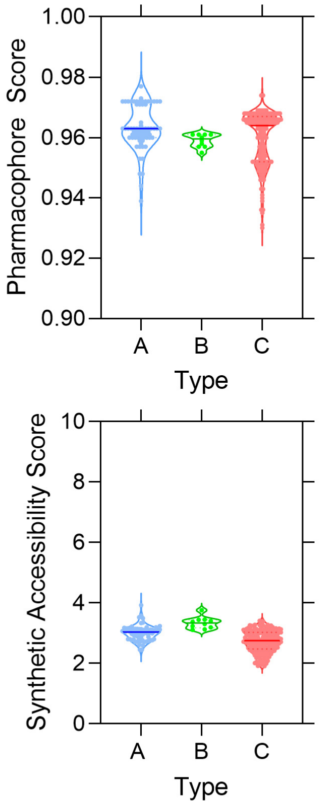

Synthetic compound				Similarity	Generated compound by DQ			
Comp ID	Structure	PH score	SA score		Comp ID	Structure	PH score	SA score
DQ-02	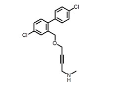	0.963	2.451	1.00	TypeA-035	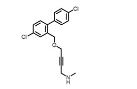	0.963	2.451
DQ-04	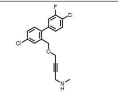	0.963	2.615	1.00	TypeA-036	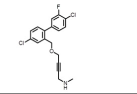	0.963	2.615
DQ-06	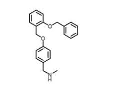	0.963	1.702	0.69	TypeC-116	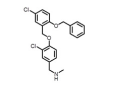	0.958	1.920
DQ-07	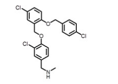	0.949	1.994	1.00	TypeC-163	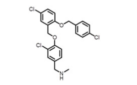	0.949	1.994
DQ-09	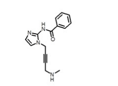	0.801	2.633	0.84	TypeA-055	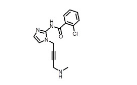	0.960	2.716
DQ-10	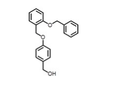	0.000	1.638	0.58	TypeC-116	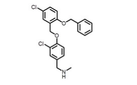	0.958	1.920
DQ-11	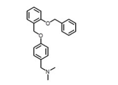	0.771	1.73	0.68	TypeC-116	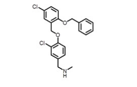	0.958	1.920
DQ-12	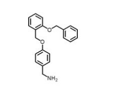	0.965	1.665	0.65	TypeC-116	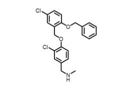	0.958	1.920
DQ-14	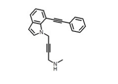	0.834	2.852	0.39	TypeB-007	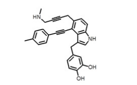	0.958	3.114
DQ-15	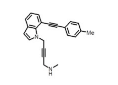	0.822	2.871	0.40	TypeB-007	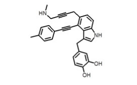	0.958	3.114
DQ-16	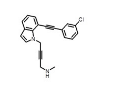	0.941	2.947	0.39	TypeB-007	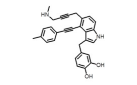	0.958	3.114
DQ-18	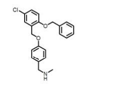	0.965	1.793	0.85	TypeC-116	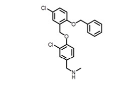	0.958	1.920
DQ-19	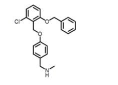	0.965	1.868	0.67	TypeC-116	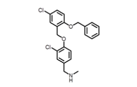	0.958	1.920
DQ-21	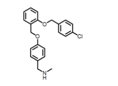	0.964	1.782	0.789	TypeC_032	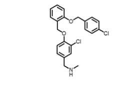	0.967	1.91

Supplementary Figure 3| Statistic analysis for the candidate compounds generated by DQ
Distribution of the pharmacophore (PH) score (**a**, see Methods, 0.0, does not fit ~ 1.0 fit) and synthetic accessibility (SA) scores (**b**, 1, easy to make ~ 10, very difficult to make) for the candidate compounds in each type of pharmacophores are plotted. Mean values are indicated as lines for each type of pharmacophore. **c**, Comparison of the synthesized compounds (left) and their original compounds generated by DQ (right). Their PH and SA scores are indicated. The similarity of the chemical structure between synthesized and original compounds is evaluated by RDKFingerprint (https://www.rdkit.org).

a. Synthesis of DQ-02

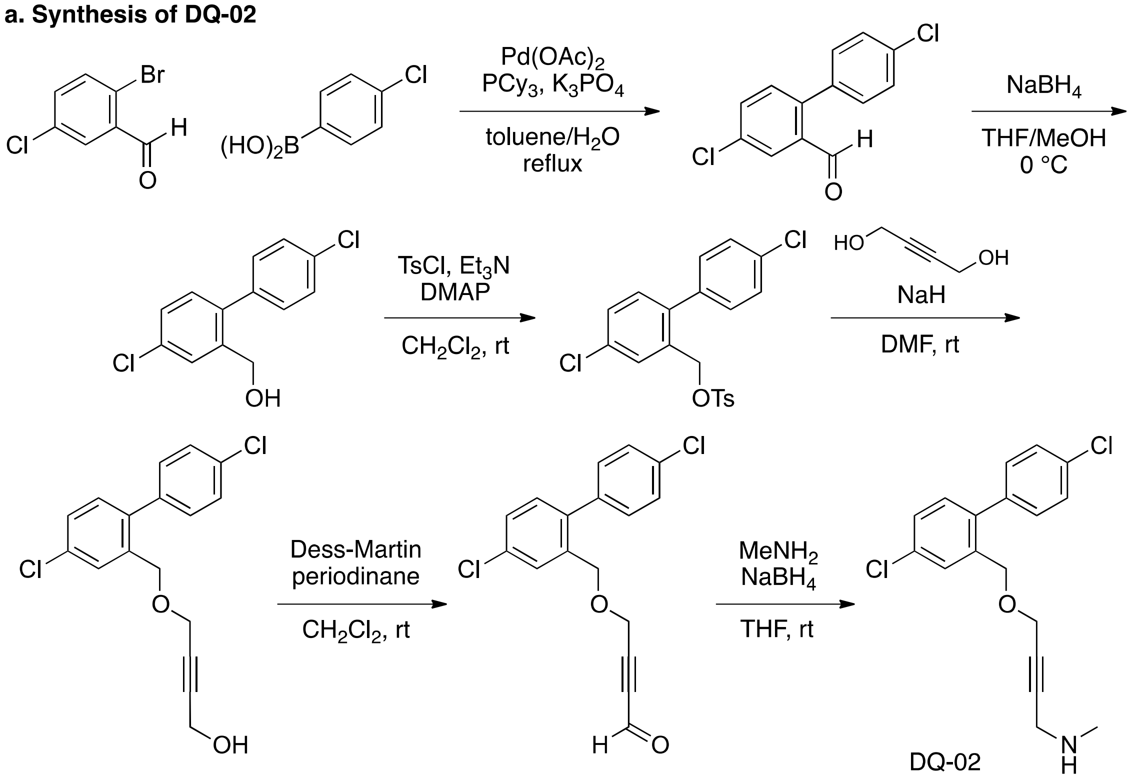

b. Synthesis of DQ-06, DQ-18 and DQ-21

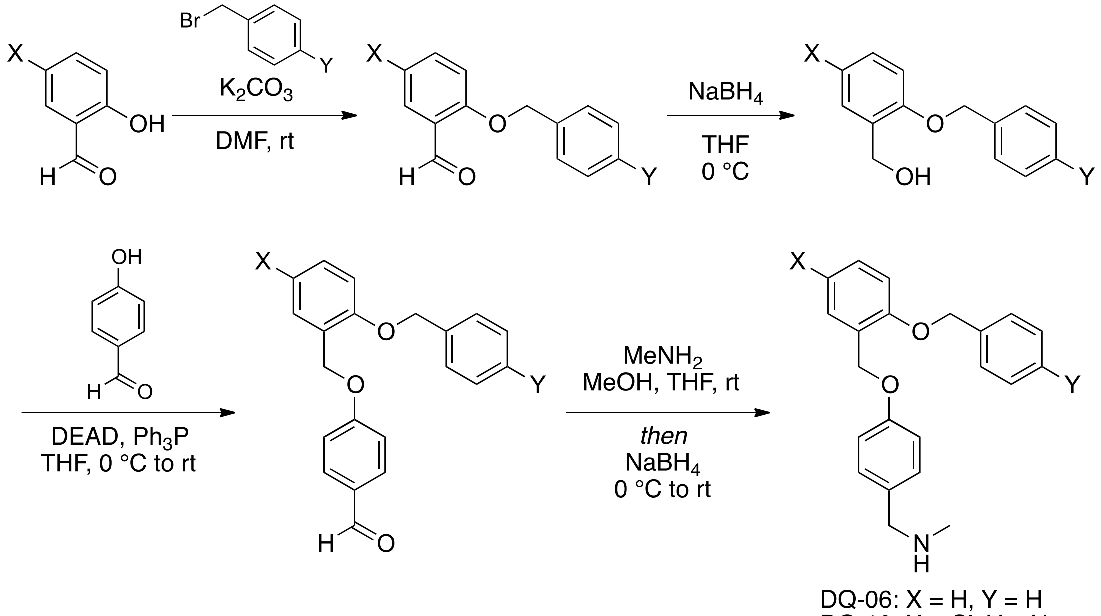

DQ-06: X = H, Y = H
DQ-18: X = Cl, Y = H
DQ-21: X = H, Y = Cl

Supplementary Figure 4| Synthesis of candidate compounds

a, Synthesis of DQ-02. Ac = acetyl; Cy = cyclohexyl; DMAP = 4-(dimethylamino)pyridine; DMF = *N*,*N*-dimethylformamide; THF = tetrahydrofuran; Ts = *p*-toluenesulfonyl. **b**, Synthesis of DQ-06, DQ-18 and DQ-21. DEAD = diethyl azodicarboxylate; Ph = phenyl.

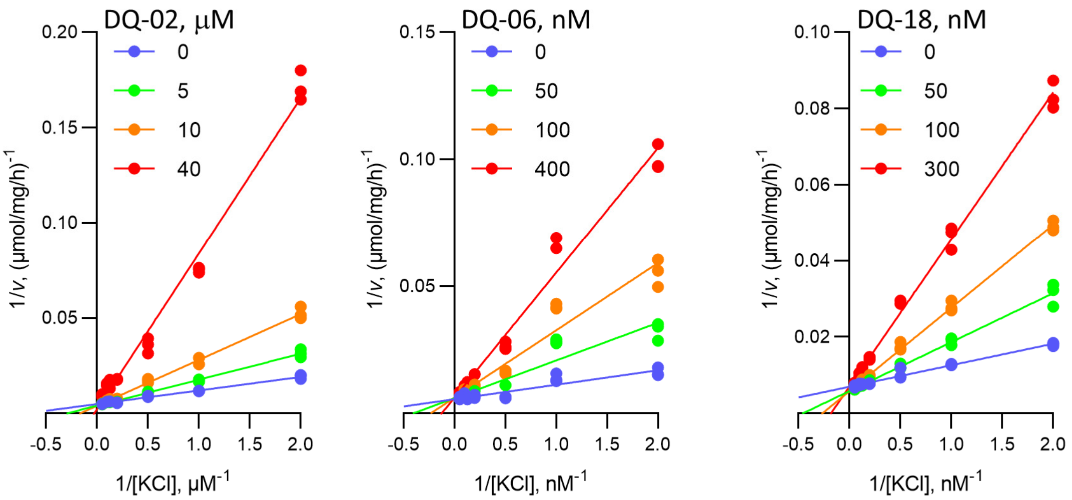

Supplementary Figure 5| K⁺-competitive inhibition of synthesized compounds
K⁺-stimulated ATPase activity of H⁺,K⁺-ATPase-enriched membrane fraction. Lineweaver-Burk inverse plot of each assay in the absence or presence of indicated concentrations of three different compounds used for the structural analysis.

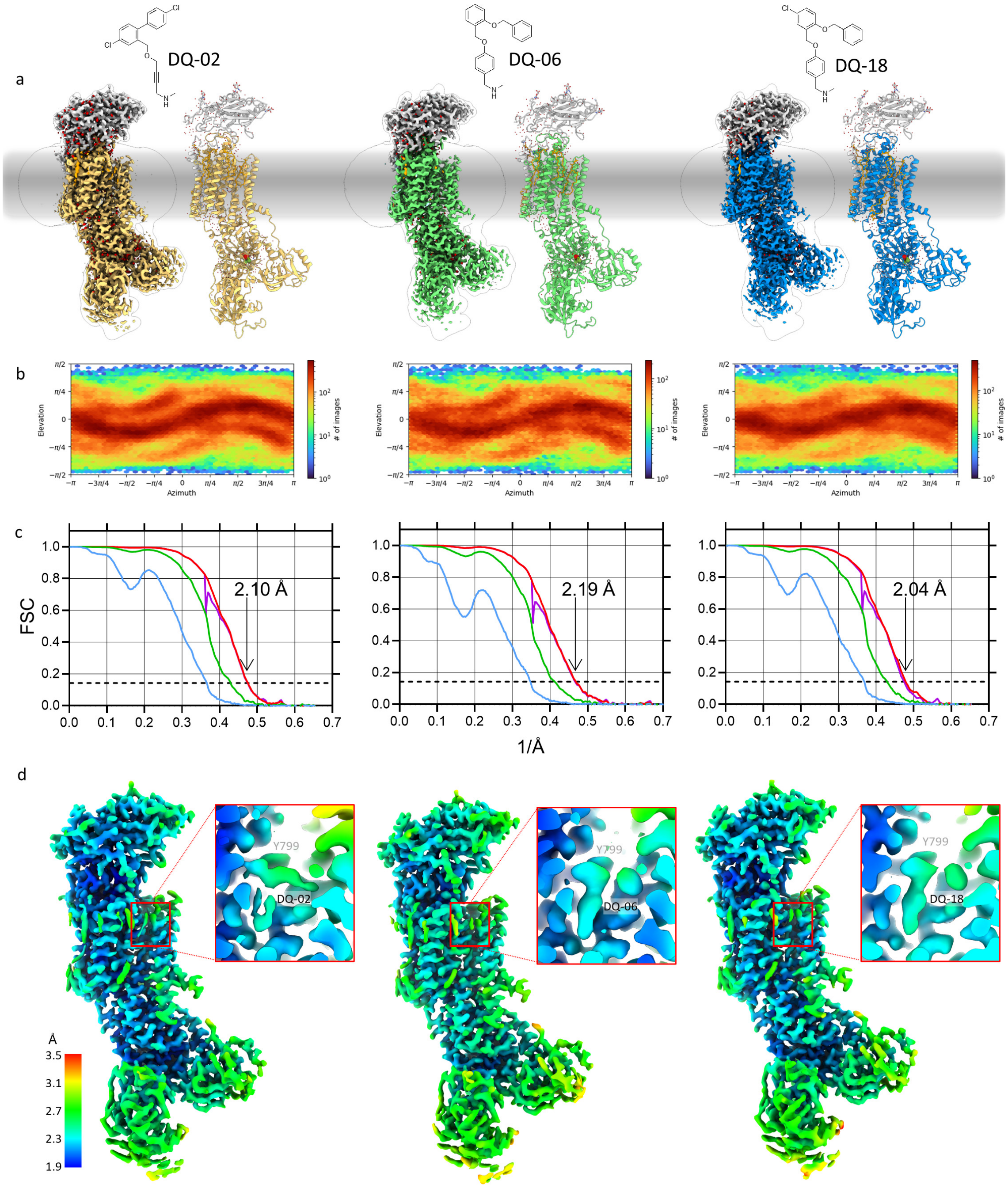

Supplementary Figure 6| Cryo-EM analysis

a, Overall structure of H^+,K^+-ATPase complexed with DQ-02 (yellow), DQ-06 (green) and DQ-18 (blue). **b**, Angular distribution plot of particles included in the final 3D reconstruction. The number of views at each angular orientation is represented by the color (blue to red). **c**, FSC plot used for resolution estimation (blue: no mask, green: loose, red: tight, purple: corrected). Dotted lines indicate FSC value of 0.143. **d**, Unsharpened maps colored by local resolution as calculated by cryoSPARC (scale is indicated in the figure). Inset images correspond to the close-up view of the compound binding site. In this figure, left, middle and right panels correspond to the data obtained form DQ-02, DQ-06 and DQ-18 bound forms, respectively.
