# Supplementary material for: Deep learning driven de novo drug design based on gastric proton pump structures
Source: Commun Biol. 2023 Sep 19;6:956. doi: 10.1038/s42003-023-05334-8 (PMC10509173; doi:10.1038/s42003-023-05334-8)
Supplement: Supplementary file 3 — Description of Additional Supplementary Files [file 42003_2023_5334_MOESM3_ESM.pdf]

Description of Additional Supplementary Files

File name: Supplementary Data 1
Description: Raw data for the ATPase measurement.

File name: Supplementary Data 2
Description: Procedures and results for the chemical synthesis of compounds use d in this study.

File name: Supplementary Software
Description: ZIP file for Deep Quartet Software.

File name: Supplementary Movie 1
Description: Close-up view of DQ-02 binding site as shown in Figure 2.

File name: Supplementary Movie 2
Description: Close-up view of DQ-06 binding site as shown in Figure 3.

File name: Supplementary Movie 3
Description: Close-up view of DQ-18 binding site as shown in Figure 4.
