# Supplementary material for: Deep learning driven de novo drug design based on gastric proton pump structures
Source: Commun Biol. 2023 Sep 19;6:956. doi: 10.1038/s42003-023-05334-8 (PMC10509173; doi:10.1038/s42003-023-05334-8)

Supplementary Data 2 for

Deep learning driven *de novo* drug design based on gastric proton pump structures

Kazuhiro Abe[1,2,3]*, Mami Ozako[2], Miki Inukai[2], Youe Matsuyuki[2], Shinnosuke Kitayama[2], Chisato Kanai[4], Chiaki Nagai[2], Chai C. Gopalasingam[5], Christoph Gerle[5], Hideki Shigematsu[6], Nariyoshi Umekubo[2], Satoshi Yokoshima[2]* & Atsushi Yoshimori[7]*

[1]Cellular and Structural Physiology Institute, Nagoya University, Nagoya, Aichi, Japan

[2]Graduate School of Pharmaceutical Sciences, Nagoya University, Nagoya, Aichi, Japan

[3]Center for One Medicine Innovative Translational Research, Gifu University Institute for Advanced Study, Gifu, Japan

[4]INTAGE Healthcare, Inc., 3-5-7, Kawaramachi Chuo-ku, Osaka 541-0048, Japan

[5]RIKEN Spring-8 Center, Kouto, Sayo-gun, Hyogo 679-5148, Japan

[6]Japan Synchrotron Radiation Research Institute (JASRI), SPring-8, 1-1-1 Kouto, Sayo, Hyogo 679-5148, Japan

[7]Institute for Theoretical Medicine, Inc., 26-1, Muraoka-Higashi 2-chome, Fujisawa, Kanagawa, 251-0012, Japan

*Correspondence to:

kabe@cespi.nagoya-u.ac.jp (K.A.)

yokosima@ps.nagoya-u.ac.jp (S.Y.)

yoshimori@itmol.com (A.Y.)

General Remarks

Nuclear magnetic resonance (NMR) spectra were determined on a JEOL-ECS400 or a JEOL-ECZ400 instrument unless otherwise noted. Chemical shifts for ^{1}H NMR are reported in parts per million (ppm) relative to internal standard tetramethylsilane (0.00 ppm) or residual solvent peaks (deuteriochloroform = 7.26 ppm; dimethyl sulfoxide-d_6 = 2.50 ppm; methanol-d_4 = 3.31 ppm) and coupling constants are in hertz (Hz). The following abbreviations are used for spin multiplicity: s = singlet, d = doublet, t = triplet, q = quartet, m = multiplet, br = broad. Chemical shifts for ^{13}C NMR were reported in ppm relative to solvent peaks (deuteriochloroform = 77.0 ppm; dimethyl sulfoxide-d_6 = 39.5 ppm; methanol-d_4 = 49.0 ppm). Infrared (IR) spectra were recorded on a JASCO FT/IR-410 Fourier Transform Infrared Spectrophotometer and were reported in wavenumbers (cm^{-1}). High resolution mass spectra (HRMS) were obtained on a Bruker Daltonics compact in electrospray ionization (ESI) method, using ESI tuning mix as the internal standard. Analytical thin layer chromatography (TLC) was performed on Merck precoated analytical plates, 0.25 mm thick, silica gel 60 F254. Preparative TLC separations were performed on Merck analytical plates (0.25 or 0.50 mm thick) precoated with silica gel 60 F254 or NH_2 F254s. Flash chromatography separations were performed on KANTO CHEMICAL Silica Gel 60 (spherical, 40-100 mesh) unless otherwise noted. Reagents were commercial grades and were used without any purification. Dehydrated tetrahydrofuran (THF) and dichloromethane were purchased from FUJIFILM Wako Pure Chemical Co. Dehydrated diethyl ether, benzene and *N,N*-dimethylformamide (DMF) were purchased from Wako Pure Chemical Co. and stored over activated MS4A. Dehydrated methanol, ethanol and acetonitrile were also purchased from FUJIFILM Wako Pure Chemical Co. and stored over activated MS3A. All reactions sensitive to oxygen or moisture were conducted under an argon atmosphere. *tert*-Butyl *N*-(4-hydroxybut-2-yn-1-yl)-*N*-methylcarbamate (**12**) was prepared according to the literature.[1]

Synthesis of DQ-02

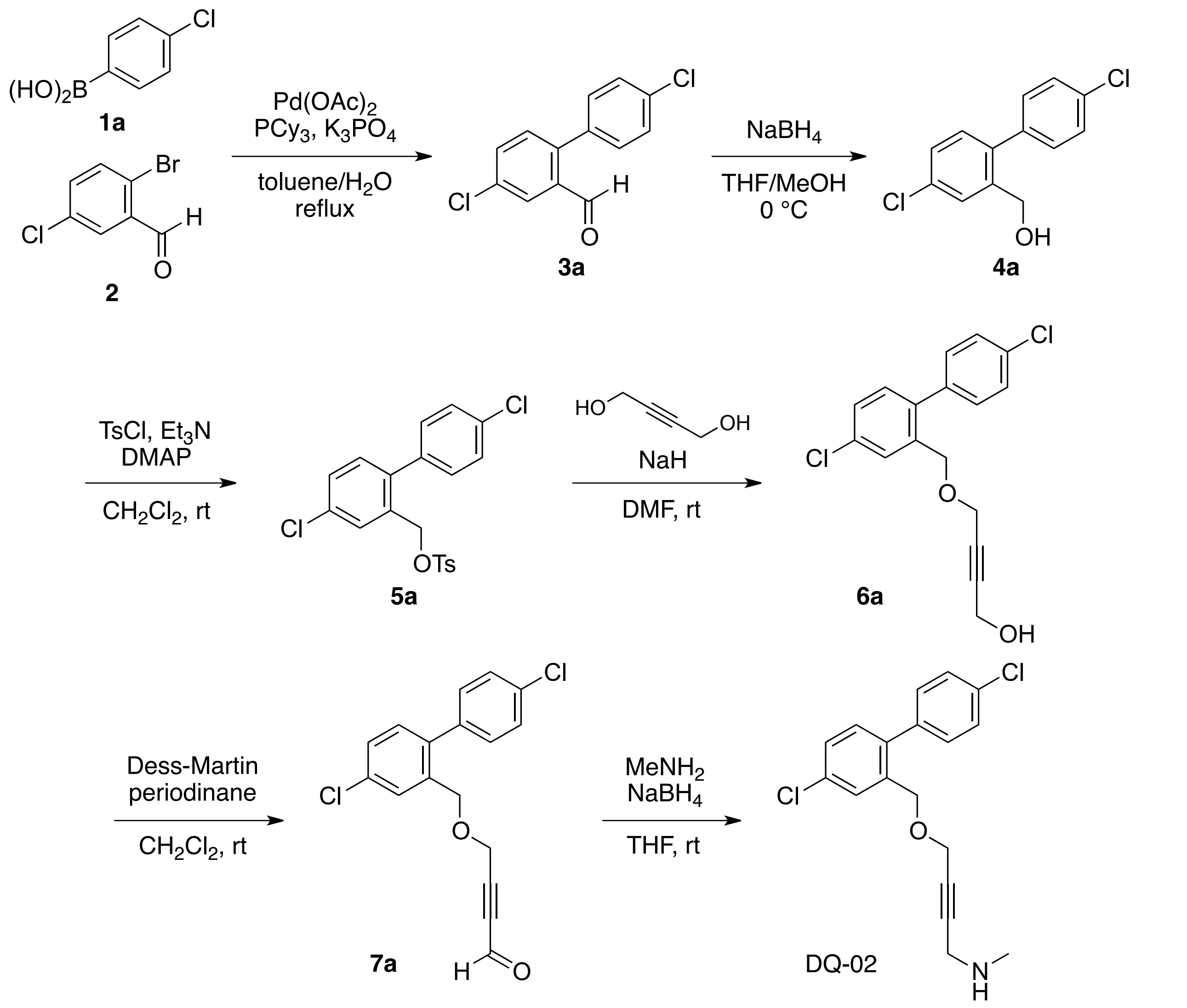

Step 1: To a stirred solution of **1a** (934 mg, 5.98 mmol) and **2** (890 mg, 4.06 mmol) in toluene (20 mL) and water (5 mL) were added palladium(II) acetate (88.5 mg, 0.394 mmol), tricyclohexylphosphine (228 mg, 0.811 mmol) and tripotassium phosphate (2.54 g, 11.8 mmol) at room temperature. After the resulting mixture was heated at reflux for 25 h, the reaction mixture was diluted with water at room temperature, and extracted three times with EtOAc. The combined organic phases were washed with brine, and dried over Na_2SO_4. After filtration, the filtrate was concentrated *in vacuo*, and the residue was purified by flash silica gel column chromatography (*n*-hexane: EtOAc = 150:1~100:1) to afford **3a** (781 mg, 3.11 mmol, 78% yield, pale yellow solid).

^{1}H NMR ($CDCl_3$, 400 MHz) δ 9.90 (s, 1H), 7.98 (d, J = 2.1 Hz, 1H), 7.60 (dd, J = 8.2, 2.1 Hz, 1H), 7.46 (d, J = 8.2 Hz, 2H), 7.37 (d, J = 8.2 Hz, 1H), 7.29 (d, J = 8.2 Hz, 2H)

^{13}C NMR ($CDCl_3$, 100 MHz) δ 190.5 (CH), 142.7 (C), 135.0 (C), 134.9 (C), 134.7 (C), 134.6 (C), 133.6 (CH), 132.1 (CH), 131.2 (CH), 128.9 (CH), 127.7 (CH)

IR (film, cm^{-1}) 2850, 1692, 1591, 1466, 1389, 1240, 1179, 1091, 820, 702

HRMS (ESI-QTOF) m/z: $[M + Na]^+$ Calcd for $C_{13}H_8Cl_2NaO$ 272.9844; Found 272.9844

Step 2: To a stirred solution of **3a** (674 mg, 2.68 mmol) in THF (27 mL) and methanol (15 mL) was added sodium borohydride (125 mg, 3.29 mmol) at 0 °C. After stirring for 40 min at the same temperature, the reaction mixture was quenched with hydrochloric acid and extracted three times with EtOAc. The combined organic phases were washed with brine and dried over Na_2SO_4. After filtration, the filtrate was concentrated *in vacuo*, and the residue was purified by flash silica gel column chromatography (*n*-hexane:EtOAc = 15:1) to afford **4a** (423 mg, 1.67 mmol, 62% yield, pale yellow solid).

^{1}H NMR ($CDCl_3$, 400 MHz) δ 7.57 (d, *J* = 2.0 Hz, 1H), 7.40 (m, 2H), 7.32 (dd, *J* = 7.9, 2.0 Hz, 1H), 7.26 (m3, 2H), 7.17 (d, *J* = 7.9 Hz, 1H), 4.56 (s, 1H)

^{13}C NMR ($CDCl_3$, 100 MHz) δ 139.7 (C), 138.2 (C), 137.8 (C), 134.0 (C), 133.7 (C), 131.2 (CH), 130.3 (CH), 128.6 (CH), 128.3 (CH), 127.7 (CH), 62.5 (CH_2)

IR (film, cm^{-1}) 3255, 1471, 1186, 1096, 1039, 1016, 1005, 820, 689, 556

HRMS (ESI-QTOF) m/z: $[M + Na]^+$ Calcd for $C_{13}H_{10}Cl_2NaO$ 275.0001; Found 275.0000

Step 3: To a stirred solution of **4a** (10.8 mg, 42.7 μmol) and 4-dimethylaminopyridine (1.7 mg, 13.9 μmol) were added dropwise a solution of *p*-toluenesulfonyl chloride (10.3 mg, 50.5 μmol) in dichloromethane (0.6 mL), and then added triethylamine (40 μl, 5.42 μmol) at 0 °C. The resulting mixture was allowed to warm up to room temperature. After stirring for 4.5 h, the reaction mixture was quenched with hydrochloric acid and extracted four times with dichloromethane. The combined organic phases were washed with brine, and dried over Na_2SO_4. After filtration, the filtrate was concentrated *in vacuo*, and the residue was purified by preparative TLC (*n*-hexane:EtOAc = 20:1) to afford **5a** (8.1 mg, 19.9 μmol, 47% yield, white solid).

^{1}H NMR ($CDCl_3$, 400 MHz) δ 7.69 (d, *J* = 7.5 Hz, 1H), 7.36-7.34 (m, 2H), 7.32 (d, *J* = 7.5 Hz, 1H), 7.27 (d, *J* = 7.6 Hz, 1H), 7.16 (d, *J* = 8.7 Hz, 1H), 7.09 (d, *J* = 7.6 Hz, 1H), 4.87 (s, 2H), 2.48 (s, 3H)

^{13}C NMR ($CDCl_3$, 100 MHz) δ 145.1 (C), 139.8 (C), 136.8 (C), 134.0 (C), 133.9 (C), 132.6 (C), 132.1 (C), 131.4 (CH), 130.5 (CH), 130.2 (CH), 129.9 (CH), 129.4 (CH), 128.5 (CH), 127.9 (CH), 69.0 (CH_2), 21.7 (CH_3)

IR (film, cm^{-1}) 1470, 1365, 1179, 1102, 1007, 932, 820, 754, 670, 553

HRMS (ESI-QTOF) m/z: $[M + Na]^+$ Calcd for $C_{20}H_{16}Cl_2NaO_3S$ 429.0089; Found 429.0089

Step 4: To a stirred solution of 2-butyne-1,4-diol (87.5 mg, 1.02 mmol) in DMF (0.6 mL) was slowly added sodium hydride (60% in oil, 9.6 mg, 0.667 mmol) at room temperature. After stirring for 30 min, to the mixture was added dropwise a solution of **5a** (43.3 mg, 0.106 mmol) in DMF (0.4 mL). After stirring for 1 h, the reaction mixture was quenched with hydrochloric acid and extracted three times with EtOAc. The combined organic phases were washed with brine and dried over Na_2SO_4. After filtration, the filtrate was concentrated *in vacuo*, and the residue was purified by preparative TLC (*n*-hexane:EtOAc = 5:1) to afford **6a** (20.2 mg, 62.9 μmol, 59% yield, white solid).

^{1}H NMR ($CDCl_3$, 400 MHz) δ 7.54 (d, *J* = 2.3 Hz, 1H), 7.40 (m, 2H), 7.33 (dd, *J* = 8.2, 2.3 Hz, 1H),

7.28 (m, 2H), 7.19 (d, J = 8.2 Hz, 1H), 4.38 (s, 2H), 4.29 (m, 2H) 4.18 (dd, J = 1.8, 1.8 Hz, 2H)
^{13}C NMR ($CDCl_3$, 100 MHz) δ 139.1 (C), 137.9 (C), 136.4 (C), 133.7 (C), 133.6 (C), 131.1 (CH), 130.5 (CH), 129.6 (CH), 128.4 (CH), 128.1 (CH), 85.0 (C), 81.3 (C), 69.0 (CH_2), 57.9 (CH_2), 51.1 (CH_2)
IR (film, cm^{-1}) 3341, 2922, 2867, 1473, 1352, 1125, 1095, 1073, 1011, 818
HRMS (ESI-QTOF) m/z: $[M + Na]^+$ Calcd for $C_{17}H_{14}Cl_2NaO_2$ 343.0263; Found 343.0263

Step 5: To a stirred solution of **6a** (9.5 mg, 29.6 μmol) in dichloromethane (1.0 mL) was added Dess-Martin periodinane (40.7 mg, 96.0 μmol) at room temperature. After stirring for 30 min, the reaction mixture was quenched with aqueous $NaHCO_3$ and $Na_2S_2O_3$, and extracted three times with EtOAc. The combined organic phases were washed with aqueous $NaHCO_3$ and dried over Na_2SO_4. After filtration, the filtrate was concentrated *in vacuo*, and the residue was purified by preparative TLC (*n*-hexane:EtOAc = 5:1) to afford **7a** (2.9 mg, 9.08 μmol, 31% yield, white solid).
^{1}H NMR ($CDCl_3$, 400 MHz) δ 9.17 (s, 1H), 7.51 (d, J = 2.3 Hz, 1H), 7.39 (dd, J = 6.5, 1.9 Hz, 2H), 7.34 (dd, J = 8.2, 2.3 Hz, 1H), 7.25 (dd, J = 6.5, 1.9 Hz, 2H), 7.19 (d, J = 8.2 Hz, 1H), 4.42 (s, 2H), 4.31 (s, 2H)
^{13}C NMR ($CDCl_3$, 100 MHz) δ 176.0 (C), 139.3 (C), 137.7 (C), 135.8, (C), 133.8 (C), 133.8 (C), 131.3 (CH), 130.5 (CH), 129.6 (CH), 128.5 (CH), 128.4 (CH), 91.3 (C), 85.8 (C), 69.6 (CH_2), 57.5 (CH_2)
IR (film, cm^{-1}) 2869, 1673, 1593, 1474, 1387, 1349, 1193, 1006, 819, 696
HRMS (ESI-QTOF) m/z: $[M + Na]^+$ Calcd for $C_{17}H_{12}Cl_2NaO_2$ 341.0107; Found 341.0108

Step 6: To a stirred solution of **7a** (50.2 mg, 0.157 mmol) in methanol (2.0 mL) was added methylamine (40% in methanol, 20 μL, 0.258 mmol) at room temperature. After stirring for 1 h, to the mixture was added sodium borohydride (11.0 mg, 0.291 mmol). After stirring for 2 h, the reaction mixture was concentrated *in vacuo*, and extracted three times with EtOAc. The combined organic phases were washed with brine and dried over Na_2SO_4. After filtration, the filtrate was concentrated *in vacuo*, and the residue was purified by preparative TLC (dichloromethane:methanol = 20:1) to afford DQ-02 (24.4 mg, 73.0 μmol, 46% yield, white solid).
^{1}H NMR ($CDCl_3$, 400 MHz) δ 7.55 (d, J = 2.4 Hz, 1H), 7.40 (dd, J = 6.6, 2.2 Hz, 2H), 7.33 (dd, J = 8.0, 2.4 Hz, 1H), 7.28 (dd, J = 6.6, 2.2 Hz, 2H), 7.18 (d, J = 8.0 Hz, 1H), 4.40 (s, 2H), 4.17 (d, J = 2.0 Hz, 2H) 3.40 (d, J = 2.0 Hz, 2H), 2.43 (s, 1H)
^{13}C NMR ($CDCl_3$, 100 MHz) δ 139.1 (C), 137.9 (C), 136.7 (C), 133.7 (C), 133.6 (C), 131.1 (CH), 130.5 (CH), 129.5 (CH), 128.4 (CH), 128.0 (CH), 85.0 (C), 78.7 (C), 68.8 (CH_2), 58.0 (CH_2), 40.2 (CH_2), 35.3 (CH_3)
IR (film, cm^{-1}) 3341, 2929, 2852, 1593, 1478, 1349, 1193, 1094, 1070, 820
HRMS (ESI-QTOF) m/z: $[M + H]^+$ Calcd for $C_{18}H_{18}Cl_2NO$ 334.0760; Found 334.0760

Synthesis of DQ-04

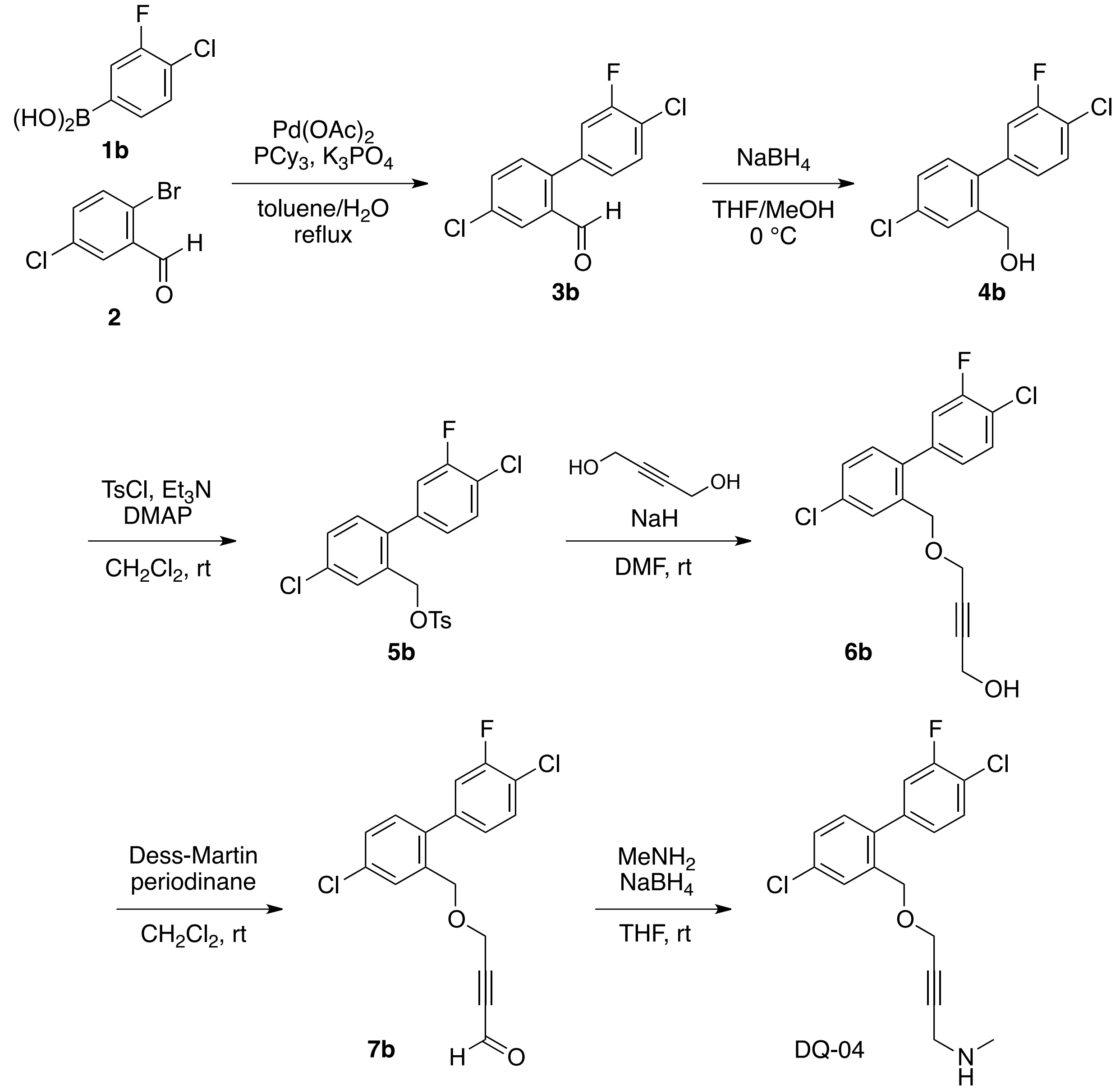

Step 1: To a stirred solution of **1b** (1.08 g, 6.18 mmol) and **2** (896 mg, 4.08 mmol) in toluene (20 mL) and H_2O (5.0 mL) were added palladium(II) acetate (94.6 mg, 0.421 mmol), tricyclohexylphosphine (225 mg, 0.803 mmol) and tripotassium phosphate (2.54 g, 11.8 mmol) at room temperature. After the resulting mixture was heated at reflux for 25 h, the reaction mixture was diluted with H_2O at room temperature, and extracted three times with EtOAc. The combined organic phases were washed with brine, and dried over Na_2SO_4. After filtration, the filtrate was concentrated *in vacuo*, and the residue was purified by flash silica gel column chromatography (*n*-hexane:EtOAc = 150:1~100:1) to afford **3b** (1.12 g, yellow solid) containing a small amount of impurities. Out of 1.12 g, 901 mg of the material was used for the next step.

^{1}H NMR ($CDCl_3$, 400 MHz) δ 9.92 (s, 1H), 7.99 (d, *J* = 2.3 Hz, 1H), 7.62 (dd, *J* = 8.2, 2.3 Hz, 1H), 7.50 (dd, *J* = 8.0, 8.0 Hz, 1H), 7.36 (d, *J* = 8.2 Hz, 1H), 7.18 (dd, *J* = 8.0, 1.6 Hz, 1H), 7.08 (dd, *J* = , 8.2, 1.6 Hz, 1H)

^{13}C NMR ($CDCl_3$, 100 MHz) δ 190.1 (CH), 157.9 (C, d, *J* = 249.8 Hz), 141.4 (C), 137.1 (C, d, *J* =

6.9 Hz), 135.2 (C), 134.6 (C), 133.7 (CH), 132.0 (CH), 130.8 (CH, d, J = 9.5 Hz), 128.0 (CH), 126.5 (CH, d, J = 3.8 Hz), 121.7 (C, d, J = 17.2 Hz), 118.0 (C, d, J = 21.9 Hz), 117.9

IR (film, cm^{-1}) 2930, 2852, 1696, 1571, 1474, 1417, 1389, 1120, 908, 823

HRMS (ESI-QTOF) m/z: $[M + Na]^+$ Calcd for $C_{13}H_7Cl_2FNaO$ 290.9750; Found 290.9749

Step 2: To a stirred solution of the above material containing **3b** (901 mg) in THF (34 mL) and methanol (17 mL) was added sodium borohydride (156 mg, 4.12 mmol) at 0 °C. After stirring for 30 min at the same temperature, the reaction mixture was quenched with hydrochloric acid and extracted three times with EtOAc. The combined organic phases were washed with brine and dried over Na_2SO_4. After filtration, the filtrate was concentrated *in vacuo*, and the residue was purified by flash silica gel column chromatography (*n*-hexane:EtOAc = 15:1) to afford **4b** (751 mg, 2.77 mmol, 83% yield in 2 steps, yellow solid).

^{1}H NMR ($CDCl_3$, 400 MHz) δ 7.57 (d, J = 2.1 Hz, 1H), 7.44 (dd, J = 8.3, 8.3 Hz, 1H), 7.33 (dd, J = 8.0, 2.1 Hz, 1H), 7.18 (d, J = 8.0 Hz, 1H), 7.16 (dd, J = 8.4, 1.8, 1H), 7.09 (dd, J = 8.3, 1.8 Hz, 1H), 4.56 (s, 2H)

^{13}C NMR ($CDCl_3$, 100 MHz) δ 157.7 (C, d, J = 248.9 Hz), 139.9 (C, d, J = 5.9 Hz), 139.4 (C), 137.3 (C), 134.3 (C), 131.1 (CH), 130.5 (CH), 128.5 (CH), 127.9 (CH), 125.6 (CH, d, J = 3.8 Hz), 120.4 (C, d, J = 15.4 Hz), 117.3 (CH, d, J = 21.0 Hz), 62.4 (CH_2)

IR (film, cm^{-1}) 3317, 1567, 1470, 1417, 1203, 1098, 1058, 901, 815, 686

HRMS (ESI-QTOF) m/z: $[M + Na]^+$ Calcd for $C_{13}H_9Cl_2FNaO$ 292.9907; Found 292.9906

Step 3: To a stirred solution of **4b** (698 mg, 2.57 mmol) and 4-dimethylaminopyridine (65.5 mg, 0.536 mmol) in dichloromethane (20 mL) were added dropwise a solution of *p*-Toluenesulfonyl chloride (599 mg, 2.93 mmol) in dichloromethane (10 mL), and then added triethylamine (0.75 mL, 5.41 mmol) at 0 °C. The resulting mixture was allowed to warm up to room temperature. After stirring for 1.5 h, the reaction mixture was quenched with hydrochloric acid and extracted three times with dichloromethane. The combined organic phases were washed with brine, and dried over Na_2SO_4. After filtration, the filtrate was concentrated *in vacuo*, and the residue was purified by flash silica gel column chromatography (*n*-hexane:EtOAc = 33:1) to afford **5b** (591 mg, 1.39 mmol, 54% yield, yellow solid).

^{1}H NMR ($CDCl_3$, 400 MHz) δ 7.68 (d, J = 8.2 Hz, 2H), 7.38-7.30 (m, 4H), 7.15 (d, J = 8.2 Hz, 1H), 6.95 (dt, J = 8.9, 1.6 Hz, 1H), 6.90 (dd, J = 8.9, 1.6 Hz, 1H), 4.86 (s, 1H), 2.47 (s, 1H)

^{13}C NMR ($CDCl_3$, 100 MHz) δ 157.6 (C, d, J = 249.8 Hz), 145.3 (C), 138.9 (C, d, J = 6.7 Hz), 138.8 (C), 134.4 (C), 132.5 (C), 132.1 (C), 131.2 (CH), 130.7 (CH), 130.5 (CH), 129.9 (CH), 129.6 (CH), 127.8 (CH), 125.5 (CH, d, J = 2.9 Hz), 120.8 (C, d, J =17.2 Hz), 117.2 (CH, d, J = 21.0 Hz), 68.7 (CH_2), 21.9 (CH_3)

IR (film, cm^{-1}) 3059, 1482, 1357, 1175, 1102, 1062, 936, 811, 661, 549

HRMS (ESI-QTOF) m/z: $[M + Na]^+$ Calcd for $C_{20}H_{15}Cl_2FNaO_3S$ 446.9995; Found 446.9994

Step 4: To a stirred solution of 2-butyne-1,4-diol (1.08 g, 12.5 mmol) in DMF (6.0 mL) was slowly added sodium hydride (60% in oil, 117 mg, 2.94 mmol) at room temperature. After stirring for 30 min, to the mixture was added dropwise a solution of **5b** (491 mg, 1.15 mmol) in DMF (4.0 mL). After stirring for 1 h, the reaction mixture was quenched with hydrochloric acid and extracted three times with EtOAc. The combined organic phases were washed with brine and dried over Na_2SO_4. After filtration, the filtrate was concentrated *in vacuo*, and the residue was purified by flash silica gel column chromatography (*n*-hexane:EtOAc = 5:1) to afford **6b** (321 mg, 0.945 mmol, 82% yield, white solid).

^{1}H NMR ($CDCl_3$, 400 MHz) δ 7.54 (d, *J* = 2.2 Hz, 1H), 7.45 (dd, *J* = 8.0, 8.0 Hz, 1H), 7.35 (dd, *J* = 8.2, 2.2 Hz, 1H), 7.21 (dd, *J* = 8.9, 1.8 Hz, 1H), 7.19 (d, *J* = 8.2 Hz, 1H), 7.09 (dd, *J* = 8.0, 1.8 Hz, 1H), 4.39 (s, 2H), 4.28 (d, *J* = 2.9 Hz,2H), 4.20 (t, *J* = 2.9, 2.9 Hz, 2H)

^{13}C NMR ($CDCl_3$, 100 MHz) δ 157.6 (C, d, *J* = 247.9 Hz), 139.9 (C, d, *J* = 7.6 Hz), 138.2 (C), 136.3 (C), 134.2 (C), 131.0 (CH), 130.4 (CH), 130.0 (CH), 128.3 (CH), 125.8 (CH, d, *J* = 3.8 Hz), 120.3 (C, d, *J* = 18.1 Hz), 117.5 (CH, d, *J* = 21.0 Hz), 85.2 (C), 81.1 (C), 68.9 (CH_2), 57.9 (CH_2), 51.0 (CH_2)

IR (film, cm^{-1}) 3309, 1475, 1353, 1207, 1123, 1071, 1021, 913, 882, 810

HRMS (ESI-QTOF) m/z: $[M + Na]^+$ Calcd for $C_{17}H_{13}Cl_2FNaO_2$ 362.0169; Found 362.0168

Step 5: To a stirred solution of **6b** (19.6 mg, 057.8 μmol) in dichloromethane (1.0 mL) was added Dess-Martin periodinane (53.3 mg, 0.126 mmol) at room temperature. After stirring for 3 h, the reaction mixture was quenched with aqueous $NaHCO_3$ and $Na_2S_2O_3$, and extracted three times with EtOAc. The combined organic phases were washed with aqueous $NaHCO_3$ and dried over Na_2SO_4. After filtration, the filtrate was concentrated *in vacuo*, and the residue was purified by preparative TLC (*n*-hexane:EtOAc = 3:1) to afford **7b** (15.9 mg, 47.2 μmol, 82% yield, white solid).

^{1}H NMR ($CDCl_3$, 400 MHz) δ 9.20 (s, 1H), 7.52 (d, *J* = 2.1 Hz, 1H), 7.45 (dd, *J* = 8.4, 8.4 Hz, 1H), 7.37 (dd, *J* = 8.1, 2.1 Hz, 1H), 7.21 (d, *J* = 8.1 Hz, 1H), 7.18 (dd, *J* = 8.4, 1.7 Hz, 1H), 4.43 (s, 2H), 4.36 (s, 2H)

^{13}C NMR ($CDCl_3$, 100 MHz) δ 176.0 (CH), 157.7 (C, d, *J* = 248.9 Hz), 139.7 (C, d, *J* = 6.7 Hz), 138.3 (C), 135.6 (C), 134.2 (C), 131.1 (CH), 130.5 (CH), 129.9 (CH), 128.6 (CH), 125.7 (CH, d, *J* = 3.8 Hz), 120.6 (C, d, *J* =28.6 Hz), 117.5 (CH, d, *J* = 21.0 Hz), 91.0 (C), 85.8 (C), 69.6 (CH_2), 57.5 (CH_2)

IR (film, cm^{-1}) 2888, 1668, 1475, 1351, 1207, 1111, 1070, 884, 813, 690

HRMS (ESI-QTOF) m/z: $[M + Na]^+$ Calcd for $C_{17}H_{11}Cl_2FNaO_2$ 359.0012; Found 359.0011

Step 6: To a stirred solution of **7b** (50.0 mg, 0.148 mmol) in methanol (2.0 mL) was added methylamine (40% in methanol, 18 μL, 0.232 mmol) at room temperature. After stirring for 1 h, to the mixture was added sodium borohydride (10.7 mg, 0.283 mmol). After stirring for 3 h, the reaction mixture was concentrated *in vacuo*, and extracted three times with EtOAc. The combined organic phases were washed with brine and dried over Na_2SO_4. After filtration, the filtrate was concentrated *in vacuo*, and the residue was purified by preparative TLC (dichloromethane:methanol

= 20:1) to afford DQ-04 (16.0 mg, 43.7 μmol, 29% yield, pale yellow solid).

^{1}H NMR ($CDCl_3$, 400 MHz) δ 7.54 (d, *J* = 2.3 Hz, 1H), 7.44 (dd, *J* = 8.0, 8.0 Hz, 1H), 7.34 (dd, *J* = 8.1, 2.3 Hz, 1H), 7.19 (dd, *J* = 12.0, 2.0 Hz, 1H), 7.18 (d, *J* = 8.1 Hz, 1H), 7.08 (ddd, *J* = 8.2, 2.0, 0.8 Hz, 1H), 4.39 (s, 2H), 4.19 (t, *J* = 1.98, 1.8 Hz,2H), 3.40 (t, *J* = 1.8, 1.8 Hz, 2H), 2.43 (s, 3H)

^{13}C NMR ($CDCl_3$, 100 MHz) δ157.6 (C, d, *J* = 247.9 Hz), , 140.0 (C, d, *J* = 6.7 Hz), 138.1 (C), 136.6 (C), 134.2 (C), 131.0 (CH), 130.3 (CH), 129.9 (CH), 128.2 (CH), 125.7 (CH, d, *J* = 3.8 Hz), 120.3 (C, d, *J* = 17.1 Hz), 117.5 (CH, d, *J* = 21.9 Hz), 85.2 (C), 78.6 (C), 68.7 (CH_2), 58.1 (CH_2), 40.2 (CH_2), 35.2 (CH_3)

IR (film, cm^{-1}) 2853, 1567, 1473, 1414, 1202, 1098, 1068, 908, 819, 690

HRMS (ESI-QTOF) m/z: $[M + H]^+$ Calcd for $C_{18}H_{17}Cl_2FNO$ 352.0666; Found 352.0666

Synthesis of DQ-09

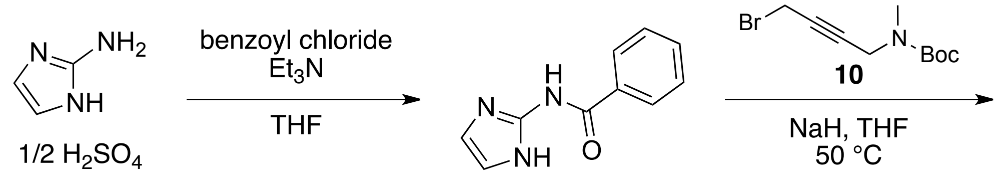

8 **9**

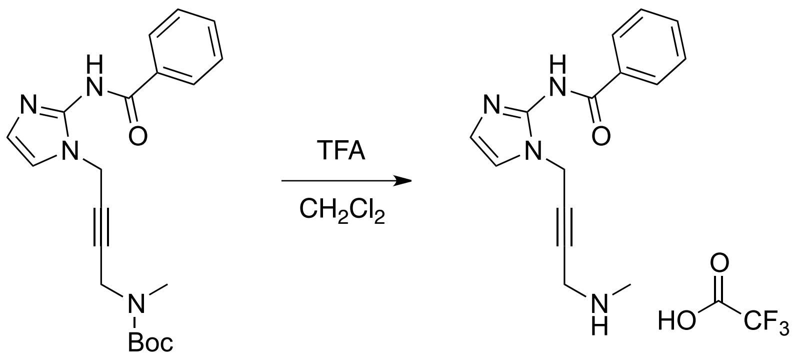

11 DQ-09

Step 1: To a solution of **8** (102 mg, 0.78 mmol) in THF (4.0 mL) were added Et_3N (1.0 mL, 7.11 mmol) and benzoyl chloride (300 μL, 2.58 mmol) at 0 °C. The resulting mixture was stirred for 14.5 h at room temperature. The resulting mixture was concentrated under reduced pressure and was quenched with 1 N aqueous NaOH. The resulting mixture was extracted three times with EtOAc. The combined organic layer was dried over Na_2SO_4 and the filtrate was concentrated under reduced pressure. The crude product was purified by silica gel column chromatography (EtOAc/hexane 1:1 to EtOAc/methanol 10:1) to give compound **9** (111 mg, 0.59 mmol, 76%) as a pale yellow solid.

^{1}H NMR (400 MHz, DMSO-d_6) δ 6.81 (s, 2H), 7.49 (dd, J = 7.4, 7.8 Hz, 2H), 7.57 (dd, J = 6.9, 7.8 Hz, 1H), 8.05 (d, J = 7.4 Hz, 2H), 11.8 (NH, brs, 1H).

^{13}C NMR (100 MHz, DMSO-d_6) δ 166.2 (C), 142.3 (C), 134.0 (C), 131.8 (CH), 128.4 (2CH), 128.0 (2CH), 117.9 (brs, 2CH).

IR (neat, cm^{-1}) 3319, 2831, 2360, 1655, 1601, 1533, 1449, 1340, 1295, 706.

HRMS (ESI-QTOF) *m*/*z* $[M+H]^+$ Calcd for $C_{10}H_{10}N_3O$ 188.0818; Found 188.0825

Step 2: To a solution of **9** (161 mg, 0.86 mmol) in THF (4.5 mL) were added NaH (72.2 mg, 1.66 mmol) at 0 °C. After stirring for 30 min at 0 °C, a solution of propargyl bromide **10** (273 mg, 1.04 mmol; preparation of **10** was describes below) in THF (3.5 mL) was added at 0 °C. The resulting mixture was stirred for 21 h at 50 °C, before the reaction was quenched with water. The resulting mixture was extracted three times with EtOAc. The combined organic layer was dried over Na_2SO_4. After filtration, the filtrate was concentrated under reduced pressure. The residue containing compound **11** was used for the next step without further purification.

Step 3: The above residue containing compound **11** was dissolved in CH_2Cl_2 (10 mL), and to the solution was added trifluoroacetic acid (1.0 mL, 13.1 mmol) at 0 °C. The resulting mixture was

stirred for 16 h at room temperature. After concentration of the resulting mixture under reduced pressure, the crude product was purified by silica gel column chromatography (EtOAc/methanol 1:10 to 0:1) to give compound DQ-09 (trifluoroacetic acid salt, 31.0 mg, 81.1 µmol, 9.4%) as a pale yellow oil.

^{1}H NMR (400 MHz, CD_3OD) δ 8.13 (m, 2H), 7.51-7.44 (m, 3H), 7.12 (s, 1H), 6.94 (s, 1H), 4.88-4.85 (m, 2H), 3.38-3.32 (m, 2H), 2.36 (s, 3H).

^{13}C NMR (100 MHz, CD_3OD) δ 171.3 (C), 163.1 (TFA-CO, q, J = 33.4 Hz), 143.3 (C), 135.1 (C), 133.6 (CH), 129.6 (2CH), 129.5 (2CH), 118.7 (CH), 117.8 (CH), 118.1 (TFA-CF_3, q, J = 291 Hz), 82.3 (C), 77.4 (CH), 38.7 (CH_2), 36.8 (CH_2), 32.6 (CH_3).

IR (neat, cm^{-1}) 3146, 2925, 2361, 1677, 1583, 1549, 1348, 1277, 1022, 883, 716.

HRMS (ESI-QTOF) *m/z* $[M+Na]^+$ Calcd for $C_{15}H_{16}N_4NaO$ 291.1216; Found 291.1217

Preparation of propargyl bromide 10

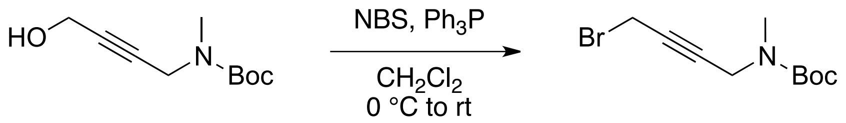

12 **10**

Compound **12** was synthesized according to the known procedure.[1] To a stirred solution of **12** (701 mg, 3.52 mmol) and *N*-bromosuccinimide (1.03 g, 5.79 mmol) in dichloromethane (7.2 mL) was added a solution of triphenylphosphine (1.40 g, 5.33 mmol) in dichloromethane (2.8 mL) dropwise at 0 °C. The resulting mixture was allowed to warm up to room temperature. After stirring for 2.5 h, the reaction mixture was extracted three times with dichloromethane. The combined organic phases were washed with brine and dried over Na_2SO_4. After filtration, the filtrate was concentrated *in vacuo*, and the residue was purified by flash silica gel column chromatography (*n*-hexane:EtOAc = 25:1) to afford **10** (482 mg, 1.84 mmol, 52% yield, pale yellow oil).

^{1}H NMR ($CDCl_3$, 400 MHz) δ 4.10 (m, 2H), 3.91 (dd, J = 2.1, 2.1, 2H), 2.89 (s, 3H), 1.46 (m, 2H)

^{13}C NMR ($CDCl_3$, 100 MHz) δ 155.0 (C, confirmed by HMBC), 82.7 (C), 80.4 (C), 78.6 (C), 38.7 (CH_2, confirmed by HMQC), 33.7 (CH_3), 28.5 (CH_3), 14.9 (CH_2)

IR (film, cm^{-1}) 2976, 2930, 1696, 1486, 1458, 1389, 1146, 874, 770, 612

HRMS (ESI-QTOF) m/z: $[M + Na]^+$ Calcd for $C_{10}H_{16}BrNNaO_2$ 284.0257; Found 284.0256

Synthesis of DQ-14

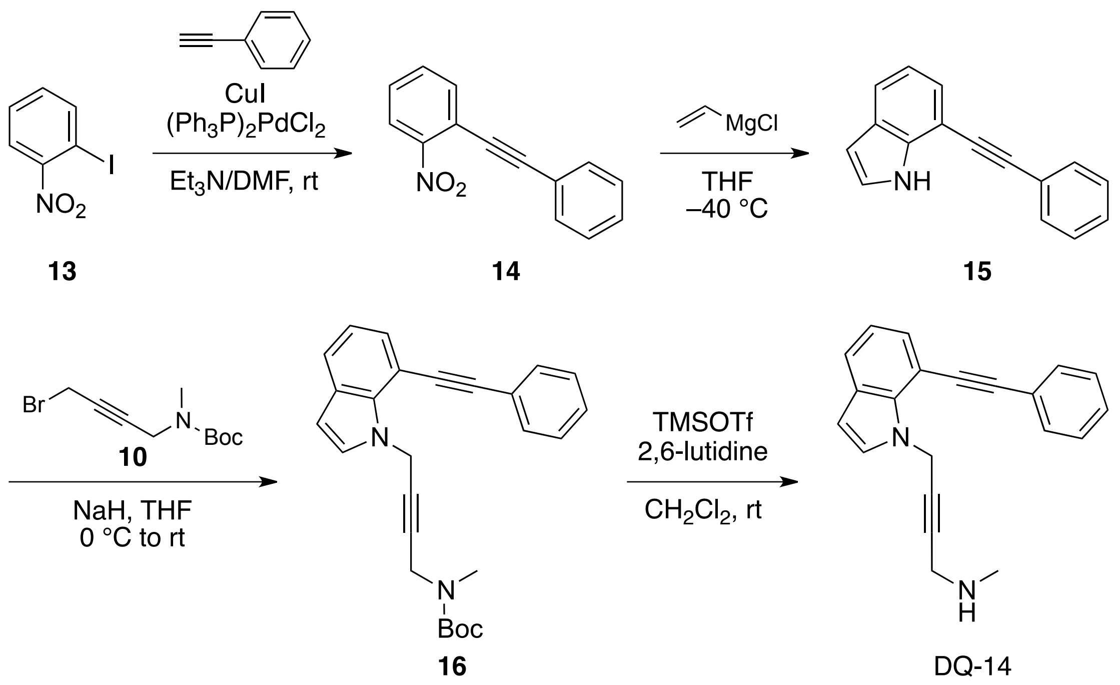

Step 1: To a stirred solution of **13** (1.02 g, 4.08 mmol) and ethynylbenzene (0.90 mL, 8.20 mmol) in triethylamine (25 mL) were added copper(I) iodide (96.5 mg, 0.507 mmol) and bis(triphenylphosphine)palladium(II) dichloride (119 mg, 0.170 mmol) at room temperature. After stirring for 1 h, the mixture was filtered through a pad of Celite and the filtrate was extracted three times with EtOAc. The combined organic phases were washed with brine and dried over Na_2SO_4. After filtration, the filtrate was concentrated *in vacuo*, and the residue was purified by flash silica gel column chromatography (*n*-hexane:EtOAc = 50:1) to afford **14** (890 mg, 3.99 mmol, 98% yield, yellow oil).

^{1}H NMR ($CDCl_3$, 400 MHz) δ 8.08 (d, *J* = 8.1 Hz, 1H), 7.72 (dd, *J* = 7.9,1.4 Hz, 1H), 7.60 (m, 3H), 7.45 (dd, J = 8.1, 7.9 Hz, 1H), 7.39 (m, 3H)

^{13}C NMR ($CDCl_3$, 100 MHz) δ 149.5 (C), 134.6 (CH), 132.8 (CH), 132.0 (CH), 129.2 (CH), 128.5 (CH), 128.4 (CH), 124.7 (CH), 122.3 (C), 118.7 (C), 97.1 (C), 84.7 (C)

IR (film, cm^{-1}) 3059, 2222, 1611, 1530, 1490, 1345, 1292, 787, 742, 690

HRMS (ESI-QTOF) m/z: $[M + Na]^+$ Calcd for $C_{14}H_9NNaO_2$ 246.0525; Found 246.0525

Step 2: To a stirred solution of **14** (106 mg, 0.474 mmol, azeotroped once with toluene prior to use) in THF (5.0 mL) was added dropwise vinylmagnesium chloride (1.39 M in THF, 1.30 mL, 1.81 mmol) at –40 °C. After stirring for 30 min, the reaction mixture was quenched with aqueous ammonium chloride and extracted three times with EtOAc. The combined organic phases were washed with brine and dried over Na_2SO_4. After filtration, the filtrate was concentrated *in vacuo*, and the residue was purified by flash silica gel column chromatography (*n*-hexane:EtOAc = 20:1) to afford **15** (44.6 mg, 0.205 mmol, 43% yield, brown solid).

^{1}H NMR ($CDCl_3$, 400 MHz) δ 8.54 (brs, 1H), 7.67 (d, *J* = 7.6 Hz, 1H), 7.60 (m, 2H), 7.41 (m, *J* = 4H), 7.28 (dd, *J* = 2.9, 2.9 Hz, 1H), 7.13 (dd, *J* = 7.6, 7.6 Hz, 1H), 6.62 (dd, J = 2.9, 1.9 Hz, 1H)
^{13}C NMR ($CDCl_3$, 100 MHz) δ 136.3 (C), 131.6 (CH), 128.4 (CH), 128.4 (CH), 127.4 (C), 125.3 (CH), 124.3 (CH), 123.2 (C), 121.6 (CH), 120.0 (CH), 106.0 (C), 103.3 (CH), 93.3 (C), 85.5 (C)
IR (film, cm^{-1}) 3435, 1490, 1434, 1336, 1272, 1058, 787, 755, 726, 694
HRMS (ESI-QTOF) m/z: $[M + H]^+$ Calcd for $C_{16}H_{12}N$ 218.0964; Found 218.0965

Step 3: To a stirred solution of **15** (23.7 mg, 0.109 mmol) in THF (0.5 mL) was added sodium hydride (60% in oil, 9.9 mg, 0.248 mmol) at 0 °C. After stirring for 1 h, the reaction mixture was added dropwise a solution of propargyl bromide **10** (39.0 mg, 0.149 mmol) in THF (0.5 mL). After stirring for 4 h, the reaction mixture was quenched with aqueous ammonium chloride and extracted three times with EtOAc. The combined organic phases were washed with brine and dried over Na_2SO_4. After filtration, the filtrate was concentrated *in vacuo*, and the residue was purified by flash silica gel column chromatography (*n*-hexane:EtOAc = 5:1) to afford **16** (31.1 mg, 78.0 µmol, 72% yield, yellow oil).
^{1}H NMR ($CDCl_3$, 400 MHz) δ 7.62 (d, *J* = 7.4 Hz, 1H), 7.59 (dd, *J* = 7.2, 1.2 Hz, 2H), 7.43 (d, *J* = 7.4 Hz, 1H), 7.41-7.36 (m, 1H), 7.38 (d, *J* = 7.2 Hz, 2H), 7.25 (s, 1H), 7.10 (dd, *J* = 7.4, 7.4 Hz, 1H), 6.56 (d, *J* = 3.2 Hz, 1H), 5.52 (s, *J* = 2H), 4.09-4.01 (m, 2H), 2.82 (s, 3H), 1.42 (s, 9H)
^{13}C NMR ($CDCl_3$, 100 MHz) δ 155.4 (C), 134.1 (C), 131.2 (CH), 129.7 (C), 128.8 (CH), 128.4 (CH), 128.2 (CH), 127.7 (CH), 123.4 (C), 122.1 (CH), 119.7 (CH), 105.9 (C), 102.4 (CH), 92.8 (C), 87.4 (C), 81.0 (C), 80.0 (C), 37.7 (CH_2), 37.5 (CH_2), 33.4 (CH_3), 28.3 (CH_3)
IR (film, cm^{-1}) 2978, 1700, 1490, 1442, 1390, 1365, 1248, 1150, 868, 756
HRMS (ESI-QTOF) m/z: $[M + Na]^+$ Calcd for $C_{26}H_{26}N_2NaO_2$ 421.1886; Found 421.1886

Step 4: To a stirred solution of **16** (29.6 mg, 74.3 µmol) in dichloromethane (0.5 mL) were added 2,6-lutidine (16.0 µl, 0.138 mmol) and trimethylsilyl trifluoromethanesulfonate (22.0 µl, 0.120 mmol) at room temperature. After stirring for 1 h, the reaction mixture was concentrated *in vacuo*. The residue was purified two times by preparative TLC (dichloromethane:methanol = 10:1) to afford DQ-14 (5.28 mg, 17.7 µmol, 24% yield, yellow solid).
^{1}H NMR ($CDCl_3$, 400 MHz) δ 7.62 (d, *J* = 7.4 Hz, 1H), 7.59 (d, *J* = 7.8 Hz, 2H), 7.42 (d, *J* = 7.8 Hz, 1H), 7.38 (d, *J* = 7.4 Hz, 1H), 7.38 (d, *J* = 7.8 Hz, 2H), 7.27-7.26 (m, 1H), 7.09 (dd, *J* = 7.4, 7.4 Hz, 1H), 6.56 (dd, *J* = 3.2, 1.4 Hz, 1H), 5.53 (d, *J* = 1.4 Hz, 2H), 3.39 (d, *J* = 1.4 Hz, 2H), 2.39 (d, *J* = 0.9 Hz, 3H)
^{13}C NMR ($CDCl_3$, 100 MHz) δ134.1 (C), 131.2 (CH), 129.6 (C), 128.7 (CH), 128.3 (CH), 128.1 (CH), 127.6 (CH), 123.4 (C), 122.0 (CH), 119.6 (CH), 105.8 (C), 102.3 (CH), 92.7 (C), 87.5 (C), 83.4 (C), 78.6 (C), 40.1 (CH_2), 37.5 (CH_2), 35.1 (CH_3)
IR (film, cm^{-1}) 3049, 2925, 1495, 1595, 1442, 1308, 1179, 783, 714, 686
HRMS (ESI-QTOF) m/z: $[M + Na]^+$ Calcd for $C_{21}H_{18}N_2Na$ 321.1362; Found 321.1361

Synthesis of DQ-15

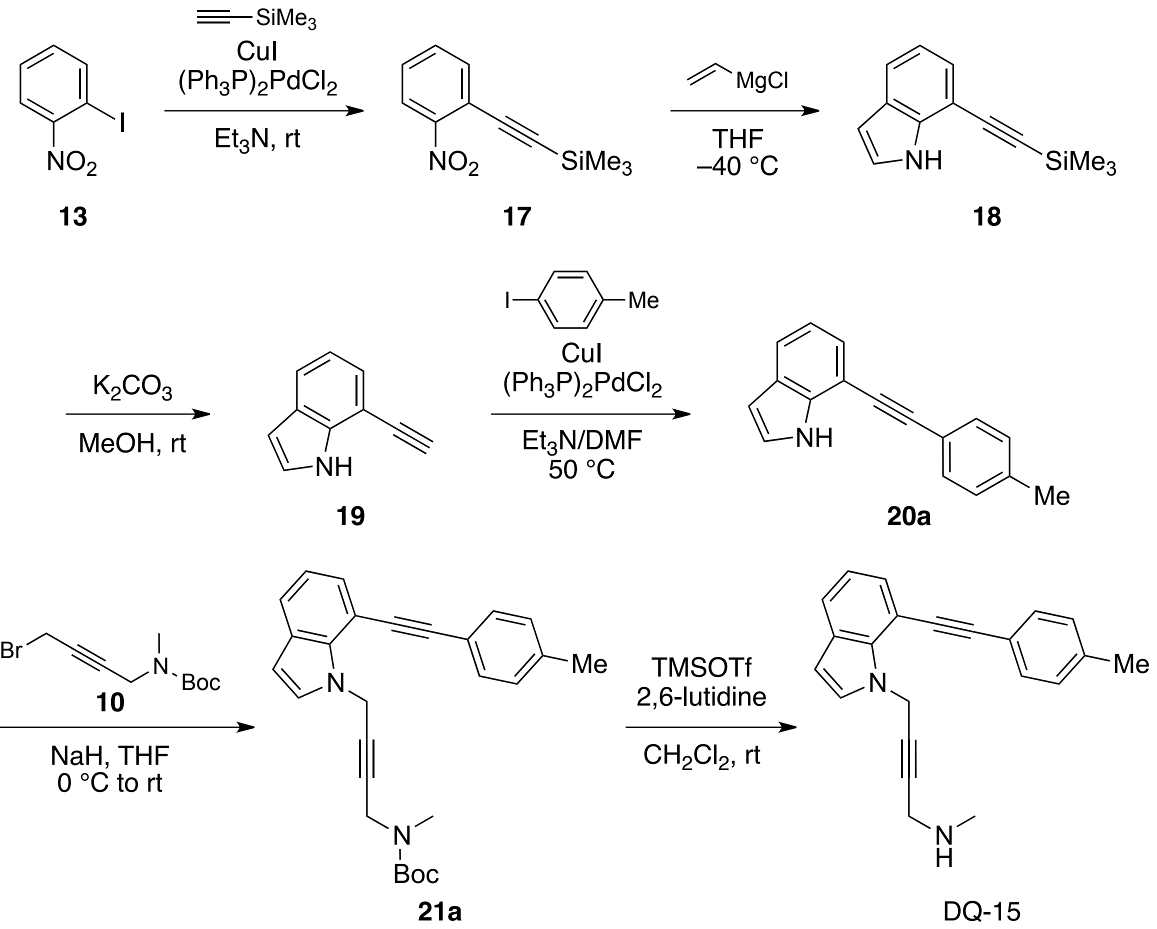

Step 1: To a stirred solution of **13** (1.97 g, 7.93 mmol) and ethynyltrimethylsilane (2.3 mL, 16.1 mmol) in triethylamine (4.0 mL) and DMF (15 ml) were added copper(I) iodide (135 mg, 0.707 mmol) and bis(triphenylphosphine)palladium(II) dichloride (230 mg, 0.327 mmol) at room temperature. After stirring for 30 min, the mixture was filtered through a pad of Celite and the filtrate was extracted three times with EtOAc. The combined organic phases were washed with brine and dried over Na_2SO_4. After filtration, the filtrate was concentrated *in vacuo*, and the residue was purified by flash silica gel column chromatography (*n*-hexane:EtOAc = 50:1) to afford **17** (1.20 g, 5.46 mmol, 69% yield, brown oil).

^{1}H NMR ($CDCl_3$, 400 MHz) δ 8.00 (dd, *J* = 7.9, 1.5 Hz, 1H), 7.65 (dd, *J* = 7.7, 1.4 Hz, 1H), 7.55 (ddd, *J* = 7.7, 7.7, 1.4 Hz, 1H), 7.44 (ddd, *J* = 7.9, 7.9, 1.5 Hz, 1H), 0.28 (s, 9H)

^{13}C NMR ($CDCl_3$, 100 MHz) δ 150.1 (C), 135.1 (CH), 132.6 (CH), 128.8 (CH), 124.4 (CH), 118.4 (C), 103.7 (C), 99.3 (C), −0.40 (CH_3)

IR (film, cm^{-1}) 2961, 2165, 1606, 1569, 1535, 1350, 1252, 872, 848, 742

HRMS (ESI-QTOF) m/z: $[M + Na]^+$ Calcd for $C_{11}H_{13}NNaO_2Si$ 242.0608; Found 242.0611

Step 2: To a stirred solution of **17** (817 mg, 3.72 mmol, azeotroped once with toluene prior to use) in THF (15 mL) was added dropwise vinylmagnesium chloride (1.38 M in THF, 11.0 mL, 15.2 mmol) at −40 °C. After stirring for 1 h, the reaction mixture was quenched with aqueous ammonium chloride and extracted three times with EtOAc. The combined organic phases were

washed with brine and dried over Na_2SO_4. After filtration, the filtrate was concentrated *in vacuo*, and the residue was purified by flash silica gel column chromatography (*n*-hexane:EtOAc = 50:1) to afford **18** (248 mg, 1.16 mmol, 31% yield, brown solid).

^{1}H NMR ($CDCl_3$, 400 MHz) δ 8.44 (brs, 1H), 7.64 (d, *J* = 7.2 Hz, 1H), 7.34 (d, *J* = 7.2 Hz, 1H), 7.26 (d, *J* = 4.2 Hz, 1H), 7.06 (dd, *J* = 7.2, 7.2 Hz, 1H), 6.58 (dd, *J* = 4.2, 2.0 Hz, 1H), 0.31 (s, 9H)

^{13}C NMR ($CDCl_3$, 100 MHz) δ 136.7 (C), 127.3 (C), 125.6 (CH), 124.2 (CH), 121.8 (CH), 119.6 (CH), 105.9 (C), 103.3 (CH), 101.3 (C), 98.4 (C), 0.16 (CH_3)

IR (film, cm^{-1}) 3430, 2961, 2144, 1434, 1325, 1252, 835, 791, 759, 726

HRMS (ESI-QTOF) m/z: $[M + H]^+$ Calcd for $C_{13}H_{16}NSi$ 214.1047; Found 214.1046

Step 3: To a stirred solution of **18** (39.5 mg, 0.185 mmol) in methanol (0.5 mL) was added potassium carbonate (37.6 mg, 0.268 mmol) at room temperature. After stirring for 30 min, the reaction mixture was quenched with aqueous ammonium chloride and extracted three times with EtOAc. The combined organic phases were washed with brine and dried over Na_2SO_4. After filtration, the filtrate was concentrated *in vacuo*, and the residue was purified by preparative TLC (*n*-hexane:EtOAc = 5:1) to afford **19** (21.1 mg, 0.149 mmol, 81% yield, brown solid).

^{1}H NMR ($CDCl_3$, 400 MHz) δ 8.48 (brs, 1H), 7.68 (d, *J* = 7.8 Hz, 1H), 7.39 (d, *J* = 7.8 Hz, 1H), 7.26 (d, *J* = 4.3 Hz, 1H), 7.10 (dd, *J* = 7.8, 7.8 Hz, 1H), 6.60 (dd, *J* = 4.3, 1.8 Hz, 1H), 3.40 (s, 1H)

^{13}C NMR ($CDCl_3$, 100 MHz) δ 136.9 (C), 127.4 (C), 125.9 (CH), 124.4 (CH), 122.0 (CH), 119.7 (CH), 104.8 (C), 103.3 (CH), 81.1 (CH), 80. 2 (C)

IR (film, cm^{-1}) 3426, 3289, 2100, 1478, 1434, 1333,1281, 1102, 795, 735

Step 4: To a stirred solution of **19** (29.7 mg, 0.210 mmol) and 4-iodotoluene (97.6 mg, 0.448 mmol) in triethylamine (0.20 mL) and *DMF* (0.50 ml) were added copper(I) iodide (4.8 g, 25.2 μmol) and bis(triphenylphosphine)palladium(II) dichloride (7.4 g, 10.5 μmol) at 50 °C. After stirring for 4 h, the mixture was filtered through a pad of Celite and the filtrate was extracted three times with EtOAc. The combined organic phases were washed with brine and dried over Na_2SO_4. After filtration, the filtrate was concentrated *in vacuo*, and the residue was purified by preparative TLC (*n*-hexane:EtOAc = 20:1) to afford **20a** (24.7 mg, 0.106 mmol, 51% yield, yellow solid).

^{1}H NMR ($CDCl_3$, 400 MHz) δ 8.54 (brs, 1H), 7.64 (d, *J* = 7.7 Hz, 1H), 7.48 (d, *J* = 6.4 Hz, 2H), 7.39 (d, *J* = 7.7 Hz, 1H), 7.28-7.26 (m, 2H), 7.11 (ddd, *J* = 7.7, 7.7, 1.7 Hz, 1H), 6.60 (ddd, *J* = 4.0, 1.7, 1.7 Hz, 1H), 2.39 (s, 3H)

^{13}C NMR ($CDCl_3$, 100 MHz) δ 138.5 (C), 136.4 (C), 131.5 (CH), 129.2 (CH), 127.4 (C), 125.2 (CH), 124.2 (CH), 121.4 (CH), 120.1 (C), 119.8 (CH), 106.3 (C), 103.3 (CH), 93.5 (C), 84.8 (C), 21.5 (CH_3)

IR (film, cm^{-1}) 3754, 3430, 1648, 1515, 1434, 1333, 1272, 815, 791, 730

HRMS (ESI-QTOF) m/z: $[M + H]^+$ Calcd for $C_{17}H_{14}N$ 232.1121; Found 232.1121

Step 5: To a stirred solution of **20a** (7.8 mg, 33.7 μmol) in THF (0.3 mL) was added sodium hydride (60% in oil, 5.5 mg, 138 mmol) at 0 °C. After stirring for 2 h, the reaction mixture was

added dropwise a solution of propargyl bromide **10** (27.4 mg, 1.045 mmol) in THF (0.3 mL). After stirring for 2 h, the reaction mixture was quenched with aqueous ammonium chloride and extracted three times with EtOAc. The combined organic phases were washed with brine and dried over Na_2SO_4. After filtration, the filtrate was concentrated *in vacuo*, and the residue was purified by preparative TLC (*n*-hexane:EtOAc = 5:1) to afford **21a** (7.1 mg, 17.2 μmol, 51% yield, yellow oil).

^{1}H NMR ($CDCl_3$, 400 MHz) δ 7.60 (d, *J* = 7.6 Hz, 1H), 7.47 (d, *J* = 7.8 Hz, 2H), 7.40 (d, *J* = 7.6 Hz, 1H), 7.25 (d, *J* = 2.5 Hz, 1H), 7.19 (d, *J* = 7.8 Hz, 2H), 6.07 (dd, *J* = 7.6, 7.6 Hz, 1H), 6.54 (d, *J* = 2.5 Hz, 1H), 5.51 (d, *J* = 0.9 Hz, 2H), 4.08 (m, 2H), 2.82 (s, 3H), 2.38 (s, 3H), 1.41 (s, 9H)

^{13}C NMR ($CDCl_3$, 100 MHz) δ 155.3 (C, confirmed by HMBC), 138.4 (C), 134.1 (C), 131.1 (CH), 129.7 (C), 129.2 (CH), 128.7(CH), 127.6 (CH), 121.9 (CH), 120.4 (C), 119.7 (CH), 106.1 (C), 102.4 (CH), 93.0 (C), 86.7 (C), 80.0 (C), 78.9 (C), 38.0 (CH_2, confirmed by HMQC), 37.5 (CH_2), 33.4 (CH_3), 28.3, (CH_3), 21.5 (CH_3)

IR (film, cm^{-1}) 2974, 1692, 1482, 1445, 1393, 1240, 1146, 820, 795, 714

HRMS (ESI-QTOF) m/z: $[M + Na]^+$ Calcd for $C_{27}H_{28}N_2NaO_2$ 435.2043; Found 435.2043

Step 6: To a stirred solution of **21a** (42.0 mg, 0.102 mmol) in dichloromethane (0.5 mL) were added 2,6-lutidine (15 μL, 0.129 mmol) and trimethylsilyl trifluoromethanesulfonate (26 μL, 0.142 mmol) at room temperature. After stirring for 1 h, the reaction mixture was concentrated *in vacuo*. The residue was purified by preparative TLC (dichloromethane:Methanol = 10:1) to afford DQ-15 (17.9 mg, 57.3 μmol, 56% yield, yellow oil).

^{1}H NMR ($CDCl_3$, 400 MHz) δ 7.60 (d, *J* = 7.6 Hz, 1H), 7.48 (d, *J* = 7.8 Hz, 2H), 7.40 (d, *J* = 7.8 Hz, 1H), 7.26 (d, *J* = 3.2 Hz, 1H), 7.19 (d, *J* = 7.8 Hz, 2H), 7.07 (dd, *J* = 7.6, 7.6 Hz, 1H), 6.54 (d, *J* = 3.2 Hz, 1H), 5.52 (s, 2H), 3.38 (s, 2H), 2.39 (s, 6H)

^{13}C NMR ($CDCl_3$, 100 MHz) δ 138.4 (C), 134.2 (C), 131.1 (CH), 129.7 (C), 129.2 (CH), 128.7 (CH), 127.6 (CH), 121.9 (CH), 120.4 (C), 119.7 (CH), 106.1 (C), 102.3 (CH), 93.0 (C), 86.8 (C), 83.6 (C), 78.7 (C), 40.2 (CH_3), 37.5 (CH_2), 35.2 (CH_2), 21.5 (CH_3)

IR (film, cm^{-1}) 3333, 2925, 2791, 1507, 1445, 1308, 1183, 816, 793, 721

HRMS (ESI-QTOF) m/z: $[M + Na]^+$ Calcd for $C_{22}H_{21}N_2$ 313.1699; Found 313.1703

Synthesis of DQ-16

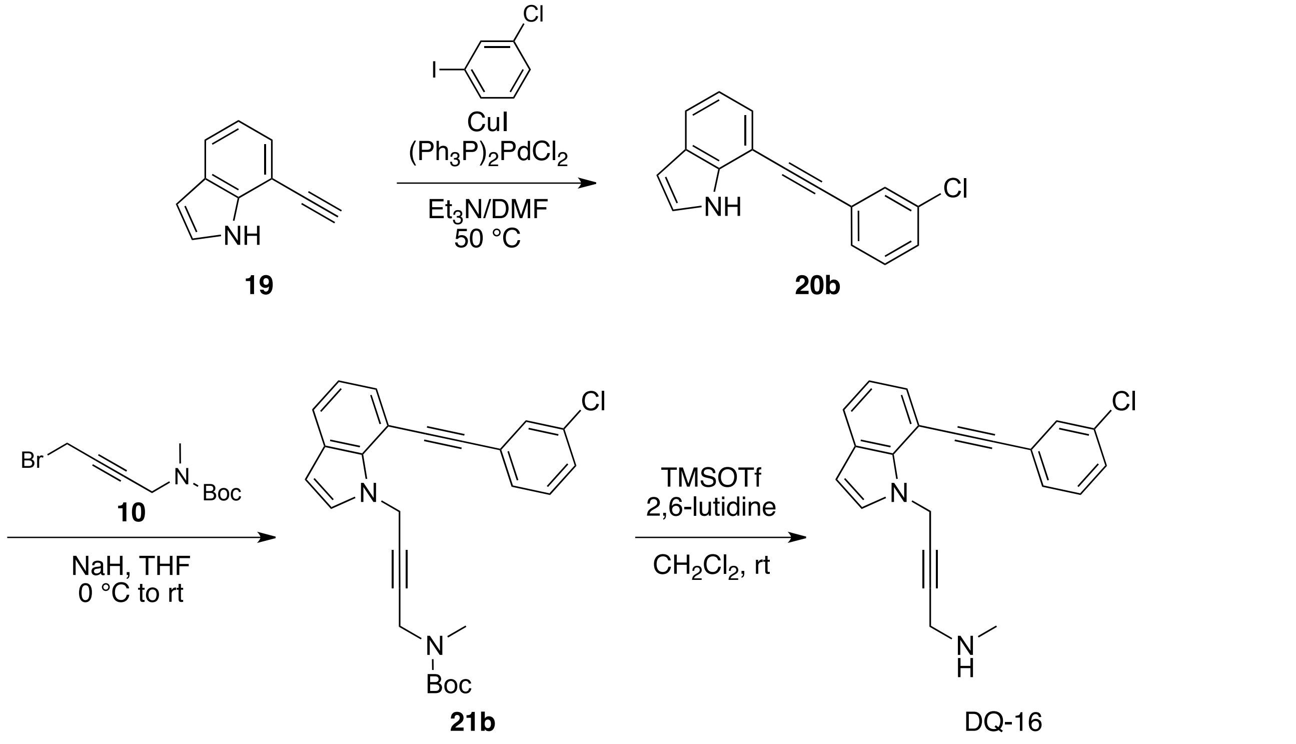

Step 1: To a stirred solution of **19** (50.0 mg, 0.354 mmol) and 1-chloro-3-iodobenzene (0.10 mL, 0.807 mmol) in triethylamine (0.20 mL) and DMF (1.0 ml) were added copper(I) iodide (6.3 mg, 33.1 μmol) and bis(triphenylphosphine)palladium(II) (10.8 mg, 15.4 μmol) dichloride at room temperature. After stirring for 30 min, the mixture was filtered through a pad of Celite and the filtrate was extracted three times with EtOAc. The combined organic phases were washed with brine and dried over Na_2SO_4. After filtration, the filtrate was concentrated *in vacuo*, and the residue was purified by flash silica gel column chromatography (*n*-hexane:EtOAc = 30:1) to afford **20b** (67.7 mg, 0.269 mmol, 76% yield, brown solid).

^{1}H NMR ($CDCl_3$, 400 MHz) δ 8.51 (brs, 1H), 7.68 (d, *J* = 7.5 Hz, 1H), 7.58 (d, *J* = 1.6 Hz, 1H), 7.47 (ddd, *J* = 6.8, 1.6, 1.6 Hz, 1H), 7.40 (d, *J* = 7.5 Hz, 1H) 7.34-7.28 (m, 3H), 7.13 (dd, *J* = 7.5, 7.5 Hz, 1H), 6.62 (dd, *J* = 3.2, 2.5 Hz, 1H)

^{13}C NMR ($CDCl_3$, 100 MHz) δ 136.3 (C), 134.2 (C), 131.4 (CH), 129.7 (CH), 129.7 (CH), 128.6 (CH), 127.5 (C), 125.5 (CH), 124.9 (C), 124.4 (CH), 122.0 (CH), 119.8 (CH), 105.5 (C), 103.4 (CH), 91.8 (C), 86.8 (C)

IR (film, cm^{-1}) 3450, 3062, 1595, 1470, 1434, 1329, 1272, 787, 722, 678

HRMS (ESI-QTOF) m/z: $[M + H]^+$ Calcd for $C_{16}H_{11}ClN$ 252.0575; Found 252.0575

Step 2: To a stirred solution of **20b** (47.4 mg, 0.188 mmol) in THF (1.0 mL) was added sodium hydride (60% in oil, 12.1 mg, 0.303 mmol) at 0 °C. After stirring for 30 min, the reaction mixture was added dropwise a solution of propargyl bromide **10** (80.0 mg, 0.305 mmol) in THF (0.50 mL). After stirring for 3 h, the reaction mixture was quenched with aqueous ammonium chloride and extracted three times with EtOAc. The combined organic phases were washed with brine and dried over Na_2SO_4. After filtration, the filtrate was concentrated *in vacuo*, and the residue was purified

second times by preparative TLC (*n*-hexane:EtOAc = 20:1, eluted twice with the same solvent system) to afford **21b** (63.2 mg, 0.146 mmol, 77%, yellow oil).

^{1}H NMR ($CDCl_3$, 400 MHz) δ 7.64 (d, *J* = 7.7 Hz, 1H), 7.56 (s, 1H), 7.46 (d, *J* = 5.9 Hz, 1H), 7.41 (d, *J* = 7.7 Hz, 1H), 7.33-7.31 (m, 2H), 7.25 (s, 1H), 7.09 (dd, *J* = 7.7, 7.7 Hz, 1H), 6.56 (d, *J* = 3.2 Hz, 1H), 5.47 (s, 2H), 4.05 (s, 2H), 2.82 (s, 3H), 1.41 (s, 9H)

^{13}C NMR ($CDCl_3$, 100 MHz) δ 154.4 (C, confirmed by HMBC), 134.2 (C), 134.1 (C), 131.0 (CH), 129.7 (CH), 129.7 (CH), 129.3 (CH), 128.9 (C), 128.4 (CH), 127.9 (CH), 125.1 (C), 122.5 (C), 119.7 (C), 105.3 (C), 102.5 (C), 91.3 (C), 88.7 (C), 81.2 (C), 80.1 (C), 78.6 (C), 38.4 (CH_2, confirmed by HMQC), 37.5 (CH_2), 33.4 (CH_3), 28.3 (CH_3)

IR (film, cm^{-1}) 2976, 1696, 1594, 1482, 1385, 1248, 1150, 876, 795, 681

HRMS (ESI-QTOF) m/z: $[M + Na]^+$ Calcd for $C_{26}H_{25}ClN_2NaO_2$ 455.1497; Found 455.1497

Step 3: To a stirred solution of **21b** (50.0 mg, 0.115 mmol) in dichloromethane (1.0 mL) were added 2,6-lutidine (20 μL, 0.172 mmol) and trimethylsilyl trifluoromethanesulfonate (30 μL, 0.163 mmol) at room temperature. After stirring for 2 h, the reaction mixture was concentrated *in vacuo*. The residue was purified by preparative TLC (dichloromethane:methanol = 10:1) to afford DQ-16 (15.8 mg, 47.5 μmol, 41% yield, white solid).

^{1}H NMR ($CDCl_3$, 400 MHz) δ 7.6 (dd, *J* = 7.6, 0.9 Hz, 1H), 7.60 (s, 1H), 7.47 (ddd, *J* = 7.8, 2.0, 2.0 Hz, 1H), 7.42 (d, *J* = 7.6 Hz, 1H), 7.33-7.31 (m, 2H), 7.26 (d, *J* = 3.7 Hz, 1H), 7.09 (dd, *J* = 7.6, 7.6 Hz, 1H), 6.56 (d, *J* = 3.7 Hz, 1H), 5.48 (t, *J* = 1.8, 1.8 Hz, 2H), 3.40 (t, *J* = 1.8, 1.8 Hz, 2H), 2.40 (s, 1H)

^{13}C NMR ($CDCl_3$, 100 MHz) δ 134.5 (C), 134.4 (C), 131.3 (CH), 130.0 (CH), 129.5 (CH), 129.1 (CH), 128.6 (CH), 128.1 (CH), 125.5 (C), 122.7 (CH), 119.9 (CH), 105.5 (C), 102.7 (CH), 91.5 (C), 89.1 (C), 84.0 (C), 78.8 (C), 40.4 (CH_2), 37.8 (CH_2), 35.5 (CH_3)

IR (film, cm^{-1}) 3337, 2925, 1593, 1442, 1308, 1182, 868, 787, 721, 686

HRMS (ESI-QTOF) m/z: $[M + Na]^+$ Calcd for $C_{21}H_{17}ClN_2Na$ 355.0972; Found 355.0971

Synthesis of DQ-06

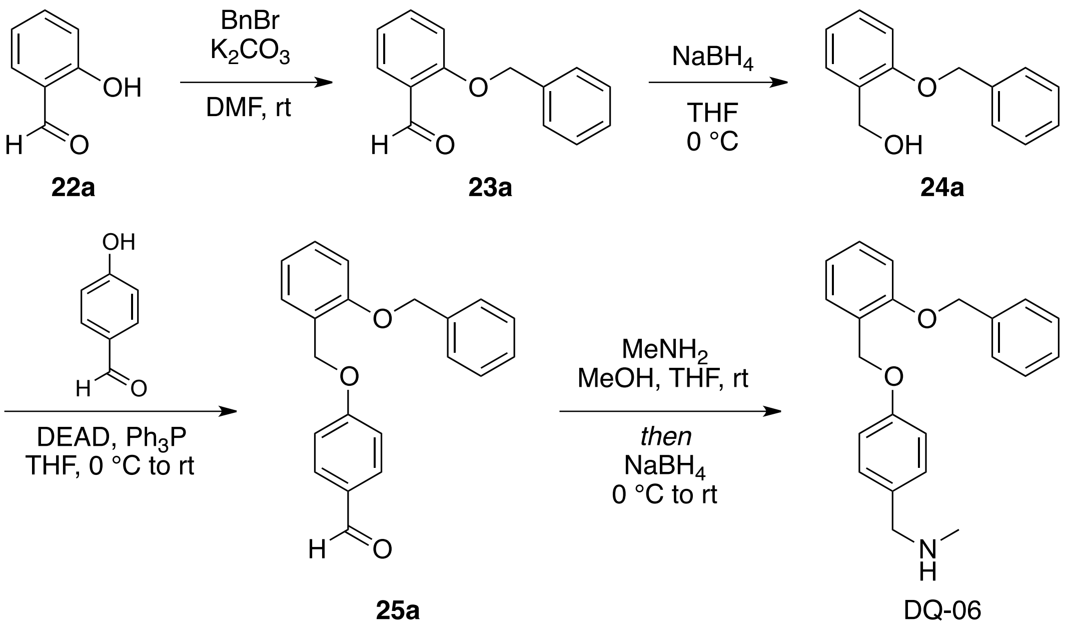

Step 1: To a solution of salicylaldehyde (**22a**, 1.00 mL, 9.58 mmol) in DMF (6 mL) were added benzyl bromide (1.25 mL, 10.5 mmol) and K_2CO_3 (2.65 g, 19.2 mmol). The mixture was stirred at room temperature for 3 days. The mixture was quenched with water and extracted three times with EtOAc. The combined organic phases were washed with brine. The organic layer was dried over anhydrous Na_2SO_4, filtered, and concentrated. The crude product was purified by column chromatography (EtOAc:*n*-hexane = 1:10) to afford **23a** (2.03 g, 9.58 mmol, quant) as a colorless oil.

^{1}H NMR (400 MHz, $CDCl_3$) δ 10.57 (s, 1H), 7.86 (dd, *J* = 8.2, 1.8 Hz, 1H), 7.56-7.52 (m, 1H), 7.46-7.34 (m, 5H), 7.07-7.03 (m, 2H), 5.20 (s, 2H).

^{13}C NMR (100 MHz, $CDCl_3$) δ 189.7 (CH), 161.0 (C), 136.0 (C), 135.9 (CH), 128.7 (CH), 128.4 (CH), 128.2 (CH), 127.2 (CH), 125.1 (C), 121.0 (CH), 113.0 (CH), 70.4 (CH_2).

IR (film, cm^{-1}): 3036, 2866, 1686, 1596, 1457, 1292, 1238, 1191, 1011, 753.

HRMS (ESI-QTOF): *m/z* [M+Na]$^+$ Calcd for $C_{14}H_{12}NaO_2$ 235.0730; Found 235.0737.

Step 2: To a solution of 2-(benzyloxy)benzaldehyde (**23a**, 2.03 g, 9.58 mmol) in THF (15 mL) was added $NaBH_4$ (779 mg, 20.6 mmol) at 0 °C. After stirring for 1 h at 0 °C, the reaction mixture was quenched with water and extracted three times with EtOAc. The combined organic phases were washed with brine. The organic layer was dried over anhydrous Na_2SO_4, filtered, and concentrated. The crude product was purified by column chromatography (EtOAc:*n*-hexane = 1:9) to afford **24a** (2.04 g, 9.51 mmol, 99%) as a colorless oil.

^{1}H NMR (400 MHz, $CDCl_3$) δ 7.44-7.25 (m, 7H), 6.99-6.95 (m, 2H), 5.13 (s, 2H), 4.74 (d, *J* = 6.4 Hz, 2H), 2.29 (t, *J* = 6.6 Hz, 1H).

^{13}C NMR (100 MHz, $CDCl_3$) δ 156.4 (C), 136.7, (C) 129.4 (C), 128.8 (CH), 128.7 (CH), 128.6 (CH), 128.0 (CH), 127.2 (CH), 120.9 (CH), 111.5 (CH), 69.9 (CH_2), 61.9 (CH_2).

IR (film, cm^{-1}): 3370, 3034, 2871, 1600, 1494, 1453, 1237, 1042, 752.
HRMS (ESI-QTOF): *m/z* $[M+Na]^+$ Calcd for $C_{14}H_{14}NaO_2$ 237.0886; Found 237.0892.

Step 3: To a solution of triphenylphosphine (268 mg, 1.02 mmol) in THF (3 mL) were added **24a** (200 mg, 0.933 mmol) and 4-hydroxybenzaldehyde (104 mg, 0.852 mmol) at 0 °C. Then diethyl azodicarboxylate (40% in toluene, 0.464 mL, 1.02 mmol) was added slowly at 0 °C. The mixture was stirred at room temperature for 20 h. The mixture was concentrated under reduced pressure and then brine was added. The aqueous phase was extracted three times with EtOAc. The combined organic phases were washed with brine. The organic layer was dried over anhydrous Na_2SO_4, filtered, and concentrated. The crude product was purified by column chromatography (EtOAc:*n*-hexane = 1:4) to afford **25a** (132 mg, 0.415 mmol, 49%) as a white solid.
^{1}H NMR (400 MHz, $CDCl_3$) δ 9.88 (s, 1H), 7.83-7.81 (m, 2H), 7.45-7.29 (m, 7H), 7.10-7.07 (m, 2H), 7.02-6.98 (m, 2H), 5.26 (s, 2H), 5.15 (s, 2H).
^{13}C NMR (100 MHz, $CDCl_3$) δ 190.9 (CH), 164.0 (C), 156.1 (C), 136.8 (C), 132.0 (CH), 129.9 (C), 129.4 (CH), 128.8 (CH), 128.6 (CH), 128.0 (CH), 127.2 (CH), 124.7 (C), 121.0 (CH), 115.2 (CH), 111.9 (CH), 70.1 (CH_2), 65.5 (CH_2).
IR (film, cm^{-1}): 3035, 2737, 1690, 1600, 1504, 1254, 1159, 1023, 753.
HRMS (ESI-QTOF): *m/z* $[M+Na]^+$ Calcd for $C_{21}H_{18}NaO_3$ 341.1148; Found 341.1151.

Step 4: To a solution of **25a** (132 mg, 0.415 mmol) in methanol (1 mL) and THF (1 mL) was added 40% methanol solution of methylamine (63.5 μL, 0.623 mmol) at room temperature and the mixture was stirred for 18 h. Then the mixture was cooled to 0 °C and sodium borohydride (15.7 mg, 0.415 mmol) was added portion wise and the mixture was stirred at 0 °C for 1 h. The reaction mixture was quenched with 1 M hydrochloric acid and partially concentrated under reduced pressure. The resulting mixture was basified with 1 M aqueous NaOH and extracted three times with dichloromethane. The combined organic phases were washed with brine. The organic layer was dried over anhydrous Na_2SO_4, filtered, and concentrated. The crude product was purified by column chromatography (dichloromethane:methanol = 4:1) to afford DQ-06 (105 mg, 0.316 mmol, 77%) as a white solid.
^{1}H NMR (400 MHz, $CDCl_3$) δ 7.52-7.31 (m, 7H), 7.25 (m, 2H), 7.00-6.95 (m, 4H), 5.16 (s, 2H), 5.14 (s, 2H), 3.74 (s, 2H), 2.44 (s, 3H).
^{13}C NMR (100 MHz, $CDCl_3$) δ 158.4 (C), 155.9 (C), 137.0 (C), 130.3 (C), 129.7 (CH), 128.8 (CH), 128.6 (CH), 128.5 (CH), 127.8 (CH), 127.1 (CH), 125.8 (C), 120.9 (CH), 114.9 (CH), 111.8 (CH), 70.0 (CH_2), 65.2 (CH_2), 54.7 (CH_2), 34.9 (CH_3).
IR (film, cm^{-1}): 3034, 2932, 1606, 1509, 1454, 1241, 1012, 752, 696.
HRMS (ESI-QTOF): *m/z* $[M+H]^+$ Calcd for $C_{22}H_{24}NO_2$ 334.1802; Found 334.1816

Synthesis of DQ-18

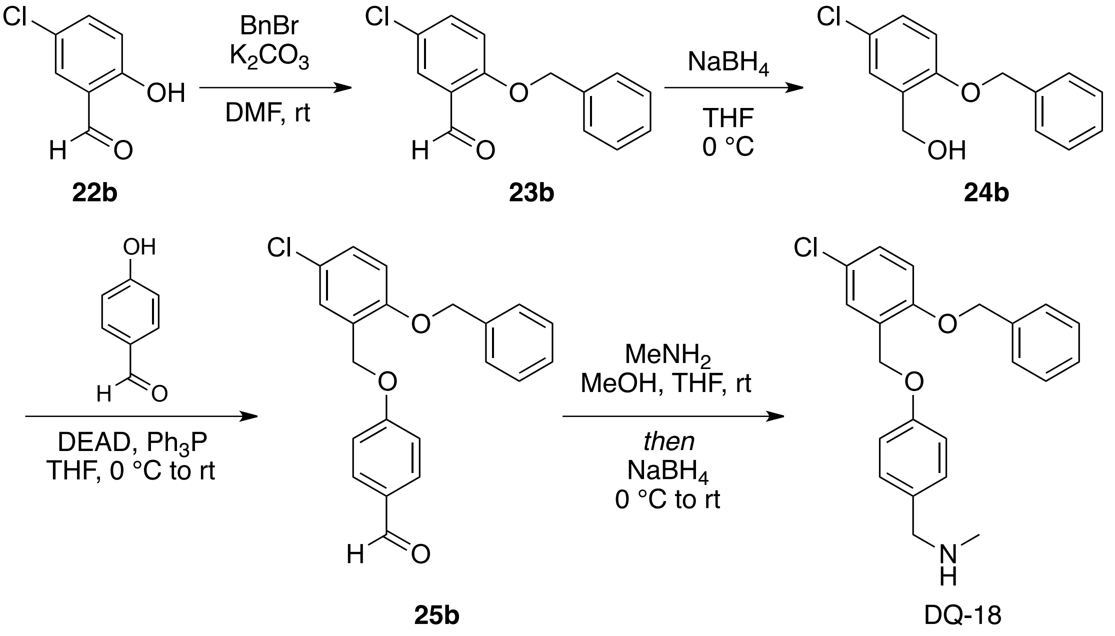

Step 1: To a solution of 5-chlorosalicylaldehyde **22b** (100 mg, 0.639 mmol) in DMF (1 mL) were added benzyl bromide (75.9 μL, 0.639 mmol) and K_2CO_3 (177 mg, 1.28 mmol). The mixture was stirred at room temperature for 19 h. The mixture was quenched with water and extracted three times with EtOAc. The combined organic phases were washed with brine. The organic layer was dried over anhydrous Na_2SO_4, filtered, and concentrated. The residue containing **23b** was subjected to the next reaction without further purification.

Step 2: To a solution of the above residue containing **23b** in THF (1 mL) was added $NaBH_4$ (48.4 mg, 1.28 mmol) at 0 °C. After stirring for 1 h at 0 °C, the reaction mixture was quenched with water and extracted three times with EtOAc. The combined organic phases were washed with brine. The organic layer was dried over anhydrous Na_2SO_4, filtered, and concentrated. The crude product was purified by column chromatography (EtOAc:*n*-hexane = 1:10) to afford **24b** (136 mg, 0.546 mmol, 86%) as a colorless oil.

^{1}H NMR (400 MHz, $CDCl_3$) δ 7.42-7.39 (m, 4H), 7.38-7.33 (m, 1H), 7.32 (d, J = 2.7 Hz, 1H), 7.20 (dd, J = 8.7, 2.7 Hz, 1H), 6.86 (d, J = 8.7 Hz, 1H), 5.10 (s, 2H), 4.70 (d, J = 3.2 Hz, 2H), 2.19 (brs, 1H).

^{13}C NMR (100 MHz, $CDCl_3$) δ 154.9 (C), 136.3 (C), 131.3 (C), 128.7 (CH), 128.4 (CH), 128.3 (2CH), 127.3 (CH), 125.9 (C), 112.8 (CH), 70.4 (CH_2), 61.4 (CH_2).

IR (film, cm^{-1}): 3368, 3034, 2873, 1597, 1489, 1456, 1240, 1185, 1017, 738.

HRMS (ESI-QTOF): *m/z* $[M+Na]^+$ Calcd for $C_{14}H_{13}ClNaO_2$ 271.0496; Found 271.0505.

Step 3: To a solution of triphenylphosphine (81.8 mg, 0.312 mmol) in THF (1 mL) were added **24b** (70.0 mg, 0.281 mmol) and 4-hydroxybenzaldehyde (31.8 mg, 0.260 mmol) at 0 °C. Then diethyl azodicarboxylate (40% in toluene, 0.142 mL, 0.312 mmol) was added slowly at 0 °C. The mixture was stirred at room temperature for 18 h. The mixture was concentrated under reduced pressure and

then brine was added. The aqueous phase was extracted three times with EtOAc. The combined organic phases were washed with brine. The organic layer was dried over anhydrous Na_2SO_4, filtered, and concentrated. The crude product was purified by column chromatography (EtOAc:*n*-hexane = 1:10) to afford **25b** (67.6 mg, 0.192 mmol, 74%) as a pale yellow solid.

^{1}H NMR (400 MHz, $CDCl_3$) δ 9.89 (s, 1H), 7.85-7.82 (m, 2H), 7.44 (d, *J* = 2.3 Hz, 1H), 7.38-7.32 (m, 5H), 7.26-7.23 (m, 1H), 7.09-7.07 (m, 2H), 6.91 (d, *J* = 8.7 Hz, 1H), 5.20 (s, 2H), 5.12 (s, 2H).

^{13}C NMR (100 MHz, $CDCl_3$) δ 190.8 (CH), 163.6 (C), 154.3 (C), 136.3 (C), 132.0 (CH), 130.2 (C), 128.8 (CH), 128.7 (CH), 128.4 (CH), 128.2 (CH), 127.2 (CH), 126.6 (C), 126.1 (C), 115.1 (CH), 113.1 (CH), 70.5 (CH_2), 64.8 (CH_2).

IR (film, cm^{-1}): 2922, 1691, 1600, 1499, 1458, 1254, 1160, 1009, 831, 740.

HRMS (ESI-QTOF): *m/z* $[M+Na]^+$ Calcd for $C_{21}H_{17}ClNaO_3$ 375.0758; Found 375.0759.

Step 4: To a solution of **25b** (67.0 mg, 0.190 mmol) in methanol (1 mL) and THF (1 mL) was added 40% methanol solution of methylamine (29.1 μL, 0.285 mmol) at room temperature and the mixture was stirred for 18 h. Then the mixture was cooled to 0 °C and sodium borohydride (7.19 mg, 0.190 mmol) was added portion wise and the mixture was stirred at 0 °C for 1 h. The reaction mixture was quenched with 1 M hydrochloric acid and partially concentrated under reduced pressure. The resulting mixture was basified with 1 M aqueous NaOH and extracted three times with dichloromethane. The combined organic phases were washed with brine. The organic layer was dried over anhydrous Na_2SO_4, filtered, and concentrated. The crude product was purified by column chromatography (dichlorimethane:methanol = 9:1) to afford DQ-18 (19.0 mg, 51.6 μmol, 27%) as a white solid.

^{1}H NMR (400 MHz, $CDCl_3$) δ 7.47 (d, *J* = 2.3 Hz, 1H), 7.40-7.33 (m, 5H), 7.26-7.24 (m, 2H), 7.20 (dd, *J* = 8.7, 2.3 Hz, 1H), 6.94 (d, *J* = 8.2 Hz, 2H), 6.87 (d, *J* = 8.7 Hz, 1H), 5.11 (s, 4H), 3.71 (s, 2H), 2.44 (s, 3H).

^{13}C NMR (100 MHz, $CDCl_3$) δ 158.0 (C), 154.2 (C), 136.5 (C), 131.1 (C), 129.8 (CH), 128.6 (CH), 128.3 (CH), 128.2 (CH), 128.1 (CH), 127.8 (C), 127.2 (CH), 126.1 (C), 114.9 (CH), 113.0 (CH), 70.4 (CH_2), 64.6 (CH_2), 54.9 (CH_2), 35.1 (CH_3).

IR (film, cm^{-1}): 2926, 1609, 1509, 1492, 1457, 1244, 1017, 806, 739.

HRMS (ESI-QTOF): *m/z* $[M+H]^+$ Calcd for $C_{22}H_{23}ClNO_2$ 368.1412; Found 368.1395.

Synthesis of DQ-07

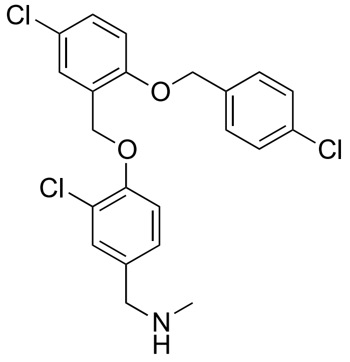

DQ-07

DQ-07 was synthesized according to the same procedure for the synthesis of DQ-06, by using 4-chlorosalicylaldehyde, 4-chlorobenzyl bromide and 3-chloro-4-hydroxybenzaldehyde instead of salicylaldehyde, benzyl bromide and 4-hydroxybenzaldehyde, respectively.

^{1}H NMR (400 MHz, $CDCl_3$) δ 7.55 (d, *J* = 2.3 Hz, 1H), 7.37-7.31 (m, 5H), 7.22 (dd, *J* = 8.7, 2.7 Hz, 1H), 7.12 (dd, *J* = 8.2, 1.8 Hz, 1H), 6.89 (d, *J* = 8.2 Hz, 1H), 6.84 (d, *J* = 9.2 Hz, 1H), 5.14 (s, 2H), 5.07 (s, 2H), 3.67 (s, 2H), 2.44 (s, 3H).

^{13}C NMR (100 MHz, $CDCl_3$) δ 154.0 (C), 153.0 (C), 134.9 (C), 134.1 (C), 134.0 (C), 130.1 (CH), 128.8 (CH), 128.6 (CH), 128.5 (CH), 128.4 (CH), 127.4 (C), 127.3 (CH), 126.4 (C), 123.1 (C), 113.9 (CH), 112.9 (CH), 69.7 (CH_2), 65.7 (CH_2), 55.0 (CH_2), 35.9 (CH_3).

IR (film, cm^{-1}): 2932, 1602, 1496, 1459, 1285, 1252, 1011, 885, 807.

HRMS (ESI-QTOF): *m/z* $[M+H]^+$ Calcd for $C_{22}H_{21}Cl_3NO_2$ 436.0632; Found 436.0628.

Synthesis of DQ-19

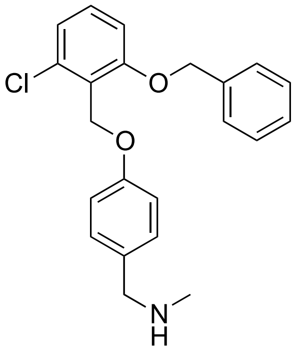

DQ-19

DQ-19 was synthesized according to the same procedure for the synthesis of DQ-06, by using 2-chloro-6-hydroxybenzaldehyde instead of salicylaldehyde.

^{1}H NMR (400 MHz, $CDCl_3$) δ 7.35-7.19 (m, 8H), 7.04 (d, *J* = 8.2 Hz, 1H), 7.00 (d, *J* = 8.2 Hz, 2H), 6.87 (d, *J* = 8.2 Hz, 1H), 5.23 (s, 2H), 5.11 (s, 2H), 3.76 (s, 2H), 2.44 (s, 3H).

^{13}C NMR (100 MHz, $CDCl_3$) δ 158.7 (C), 158.4 (C), 136.8 (C), 136.4 (C), 130.3 (CH), 129.9 (CH), 129.8 (C), 128.6 (CH), 128.0 (CH), 127.0 (CH), 123.4 (C), 122.2 (CH), 115.1 (CH), 111.0 (CH), 70.7 (CH_2), 62.2 (CH_2), 54.4 (CH_2), 34.6 (CH_3).

IR (film, cm^{-1}): 3033, 2940, 1589, 1510, 1452, 1267, 1236, 1021, 737, 699.
HRMS (ESI-QTOF): *m/z* [M+H]$^+$ Calcd for $C_{22}H_{23}ClNO_2$ 368.1412; Found 368.1413.

Synthesis of DQ-21

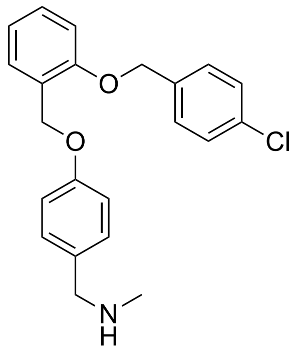

DQ-21

DQ-21 was synthesized according to the same procedure for the synthesis of DQ-06, by using 4-chlorobenzyl bromide instead of benzyl bromide.
^{1}H NMR (400 MHz, $CDCl_3$) δ 7.49-7.47 (m, 1H), 7.35-7.27 (m, 5H), 7.22 (d, J = 8.2 Hz, 2H), 7.01-6.98 (m, 1H), 6.95-6.92 (m, 3H), 5.14 (s, 2H), 5.09 (s, 2H), 3.69 (s, 2H), 2.45 (s, 3H).
^{13}C NMR (100 MHz, $CDCl_3$) δ 158.7, (C) 155.8 (C), 135.4 (C), 133.7 (C), 130.3 (CH), 129.0 (CH), 128.9 (CH), 128.8 (CH), 128.5 (CH), 128.2 (C), 125.7(C), 121.1 (CH), 115.0 (CH), 111.8 (CH), 69.3 (CH_2), 65.2 (CH_2), 53.9 (CH_2), 33.9 (CH_3).
IR (film, cm^{-1}) 3039, 2927, 1606, 1510, 1456, 1244, 1011, 810, 754
HRMS (ESI-QTOF): *m/z* [M+H]$^+$ Calcd for $C_{22}H_{23}ClNO_2$ 368.1412; Found 368.1408.

Synthesis of DQ-10

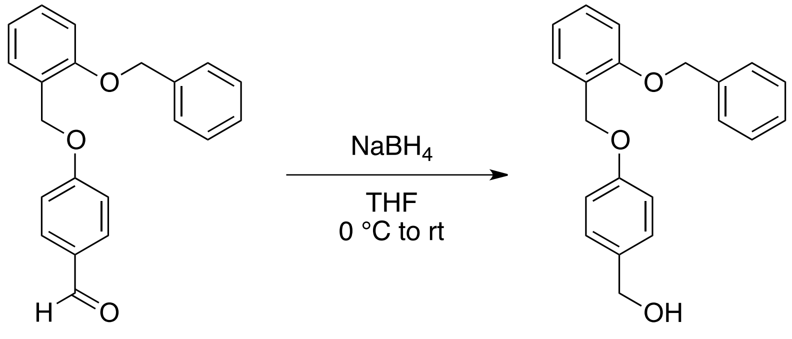

25a DQ-10

To a solution of **25a** (100 mg, 0.314 mmol) in THF (3 mL) was added $NaBH_4$ (23.8 mg, 0.628 mmol) at 0 °C. After stirring for 19 h at room temperature, the reaction mixture was quenched with water and extracted three times with EtOAc. The combined organic phases were washed with brine. The organic layer was dried over anhydrous Na_2SO_4, filtered, and concentrated. The crude product was purified by column chromatography (EtOAc:*n*-hexane = 1:4) to afford DQ-10 (81.8 mg, 0.255 mmol, 83%) as a colorless oil.
^{1}H NMR (400 MHz, $CDCl_3$) δ 7.48-7.29 (m, 9H), 7.01-6.96 (m, 4H), 5.19 (s, 2H), 5.14 (s, 2H), 4.62 (d, *J* = 3.7 Hz, 2H).

^{13}**C NMR** (100 MHz, $CDCl_3$) δ 158.6 (C), 155.9 (C), 137.0 (C), 133.2 (C), 128.9 (CH), 128.7 (CH), 128.6 (CH), 128.6 (CH), 127.9 (CH), 127.1 (CH), 125.8 (C), 120.9 (CH), 115.0 (CH), 111.8 (CH), 70.0 (CH_2), 65.2 (CH_2), 65.1 (CH_2).
IR (film, cm^{-1}): 3353, 3034, 2872, 1607, 1509, 1454, 1242, 1010, 825, 752.
HRMS (ESI-QTOF): *m/z* $[M+Na]^+$ Calcd for $C_{21}H_{20}NaO_3$ 343.1305; Found 343.1316.

Synthesis of DQ-11

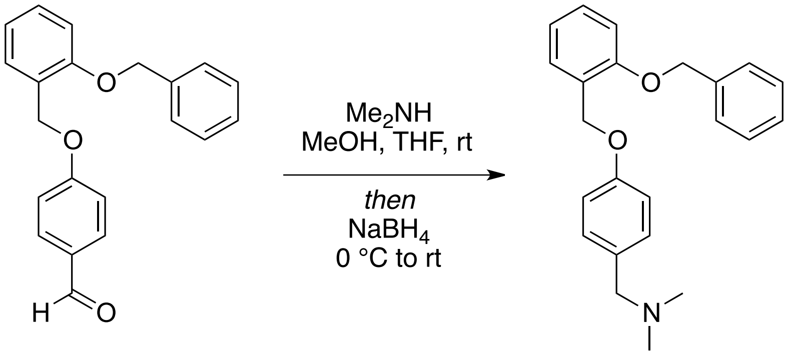

25a DQ-11

To a solution of **25** (100 mg, 0.314 mmol) in methanol (1 mL) and THF (1 mL) was added 2 M methanol solution of dimethylamine (0.236 mL, 0.471 mmol) at room temperature and the mixture was stirred for 18 h. Then the mixture was cooled to 0 °C and sodium borohydride (11.9 mg, 0.314 mmol) was added portion wise and the mixture was stirred at 0 °C for 1 h. The reaction mixture was quenched with 1 M HCl and partially concentrated under reduced pressure. The resulting mixture was basified with 1 M NaOH and extracted three times with dichloromethane. The combined organic phases were washed with brine. The organic layer was dried over anhydrous Na_2SO_4, filtered, and concentrated. The crude product was purified by column chromatography (dichloromethane:methanol = 10:1) to afford DQ-11 (24.0 mg, 0.0691 mmol, 22%) as a colorless oil.
^{1}**H NMR** (400 MHz, $CDCl_3$) δ 7.48 (d, J = 7.3 Hz, 1H), 7.42-7.40 (m, 2H), 7.37-7.25 (m, 4H), 7.21-7.19 (m, 2H), 7.00-6.93 (m, 4H), 5.17 (s, 2H), 5.13 (s, 2H), 3.37 (s, 2H), 2.23 (s, 6H).
^{13}**C NMR** (100 MHz, $CDCl_3$) δ 158.2 (C), 155.9 (C), 137.0 (C), 130.7 (C), 130.3 (CH), 128.8 (CH), 128.7 (CH), 128.5 (CH), 127.9 (CH), 127.1 (CH), 126.0 (C), 120.9 (CH), 114.7 (CH), 111.8 (CH), 70.0 (CH_2), 65.2 (CH_2), 63.6 (CH_2), 45.1 (CH_3).
IR (film, cm^{-1}): 3034, 2936, 2767, 1607, 1507, 1454, 1240, 1173, 1021, 751.
HRMS (ESI-QTOF): *m/z* $[M+H]^+$ Calcd for $C_{23}H_{26}NO_2$ 348.1958; Found 348.1967.

Synthesis of DQ-12

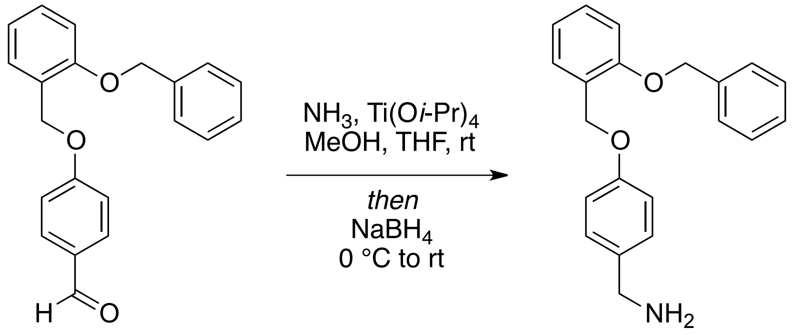

25a DQ-12

To a solution of **25a** (200 mg, 0.628 mmol) in THF (2 mL) were added 2 M methanol solution of ammonia (11.0 mL, 22.0 mmol) and tetraisopropyl orthotitanate (0.375 mL, 1.26 mmol at room temperature and the mixture was stirred for 3 h. Then the mixture was cooled to 0 °C and sodium borohydride (35.9 mg, 0.942 mmol) was added portion wise and the mixture was stirred at room temperature for 18 h. The reaction mixture was quenched with 2 M NH_4OH, filtered and concentrated. The crude product was purified by column chromatography (dichloromethane:methanol = 10:1) to afford DQ-12 (48.6 mg, 0.152 mmol, 24%) as a pale yellow oil.

^{1}H NMR (400 MHz, $CDCl_3$) δ 7.47 (d, J = 7.3 Hz, 1H), 7.42-7.40 (m, 2H), 7.38-7.24 (m, 4H), 7.21-7.19 (m, 2H), 7.00-6.94 (m, 4H), 5.17 (s, 2H), 5.13 (s, 2H), 3.79 (s, 2H).

^{13}C NMR (100 MHz, $CDCl_3$) δ 157.9 (C), 155.9 (C), 137.0 (C), 135.5 (C), 128.8 (CH), 128.7 (CH), 128.5 (CH), 128.2 (CH), 127.9 (CH), 127.1 (CH), 125.9 (C), 120.9 (CH), 115.0 (CH), 111.8 (CH), 70.0 (CH_2), 65.2 (CH_2), 45.9 (CH_2).

IR (film, cm^{-1}): 3035, 2923, 1603, 1505, 1455, 1241, 1017, 827, 733, 701.

HRMS (ESI-QTOF): m/z $[M+Na]^+$ Calcd for $C_{21}H_{21}NNaO_2$ 342.1464; Found 342.1457.

Reference:

(1) Tayama, E.; Toma, Y. *Tetrahedron* **2015**, *71*, 554-559.

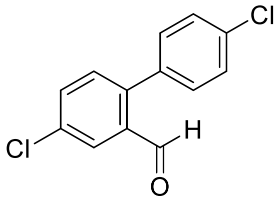

3a

^{1}H NMR (400 MHz, $CDCl_3$)

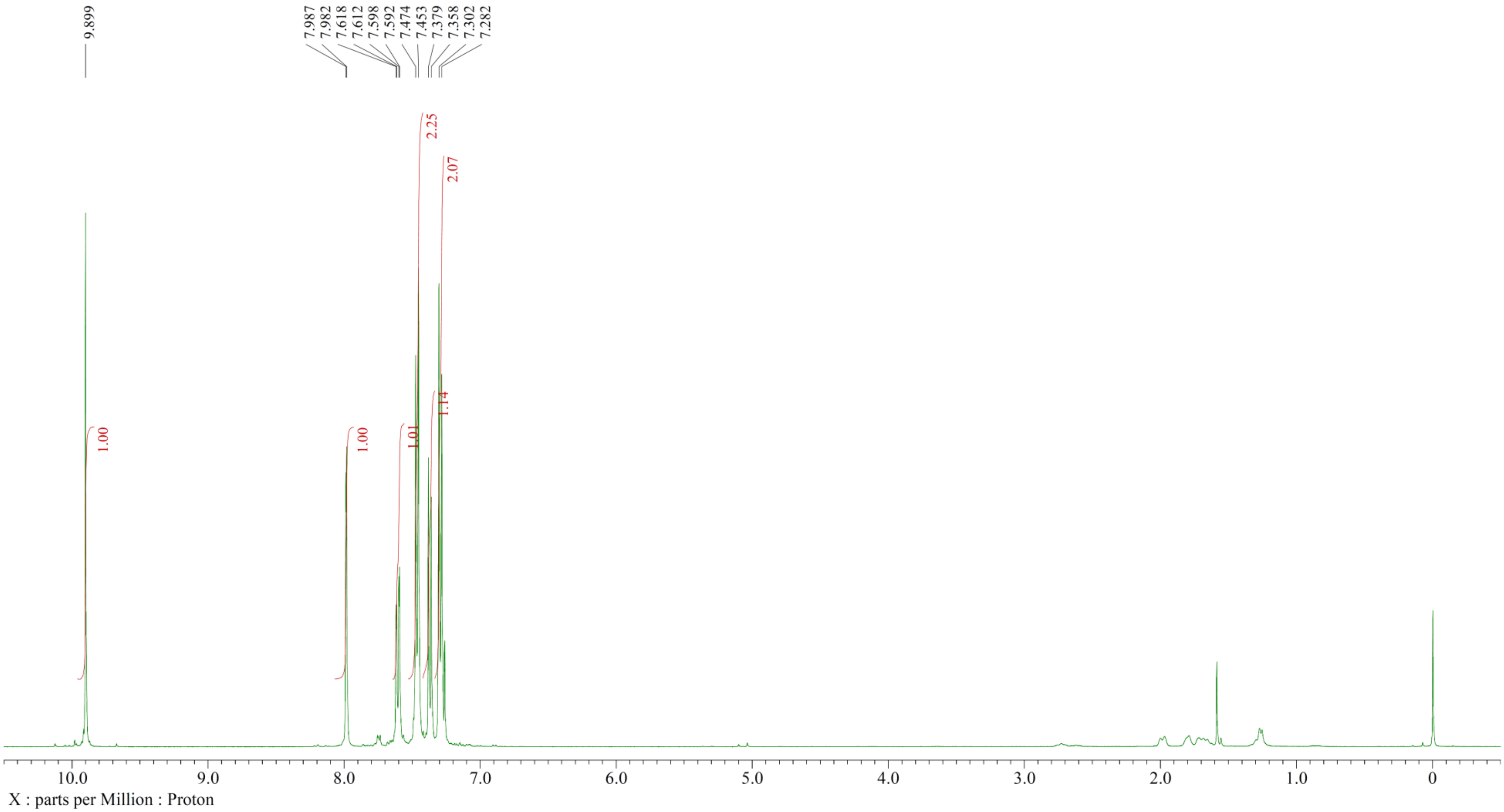

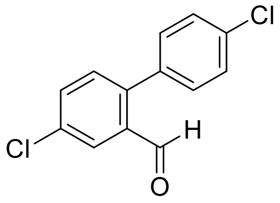
3a

^{13}C NMR (100 MHz, $CDCl_3$)

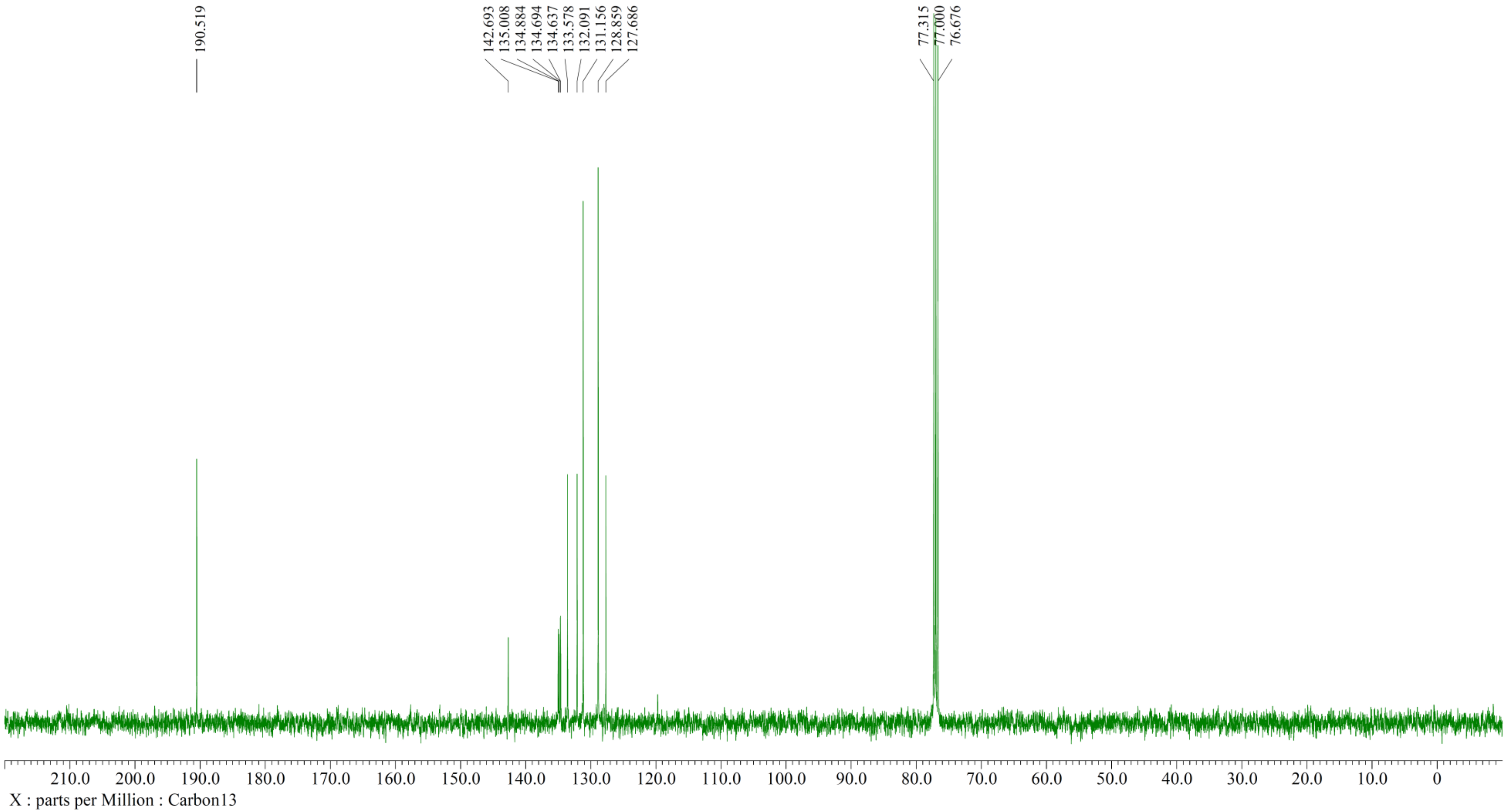

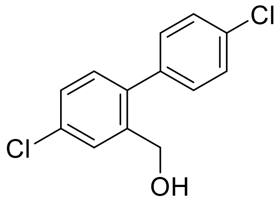

4a

^{1}H NMR (400 MHz, $CDCl_3$)

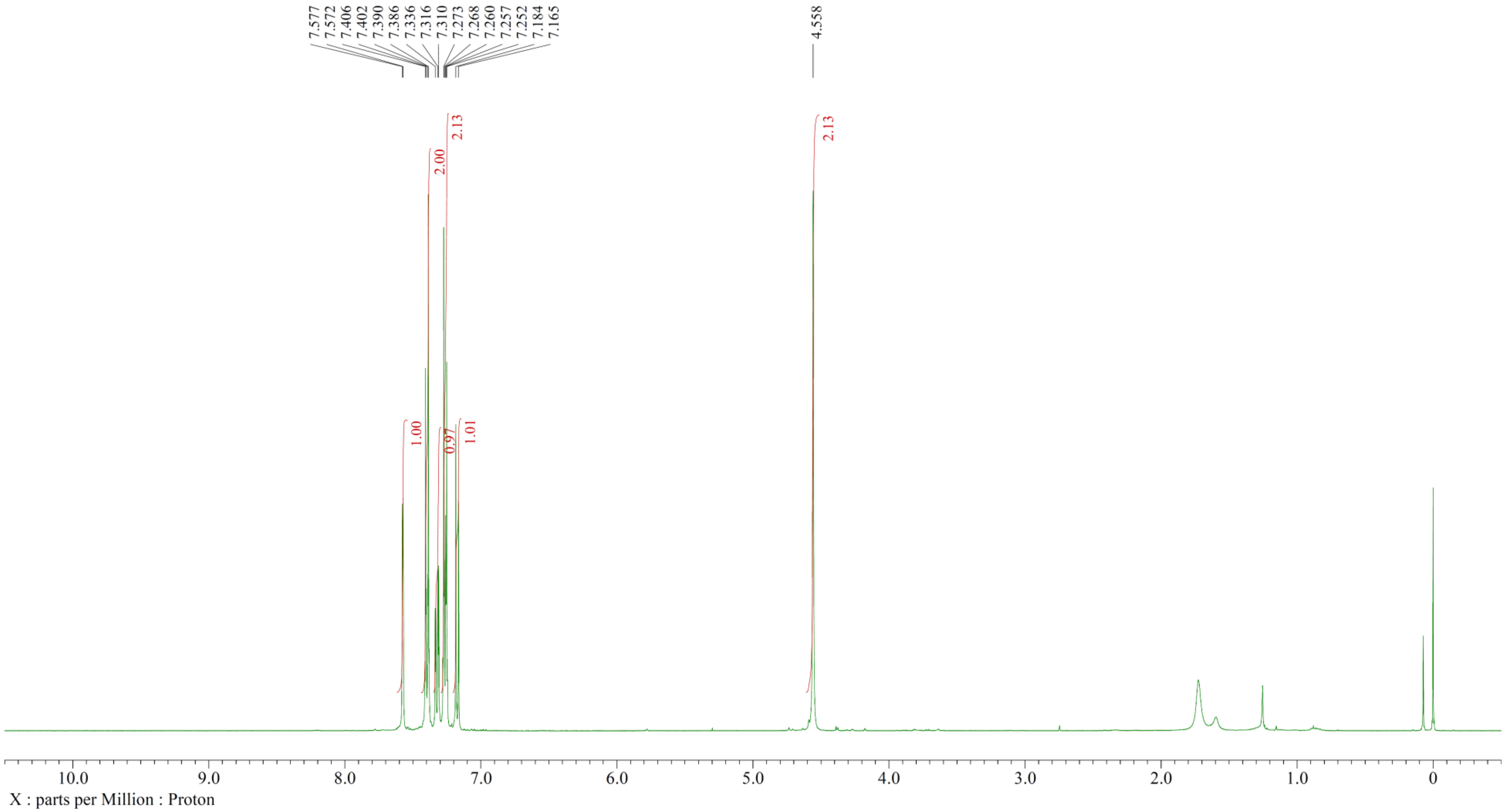

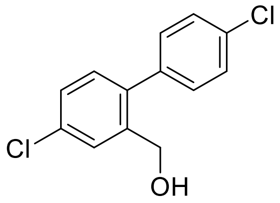

4a

^{13}C NMR (100 MHz, $CDCl_3$)

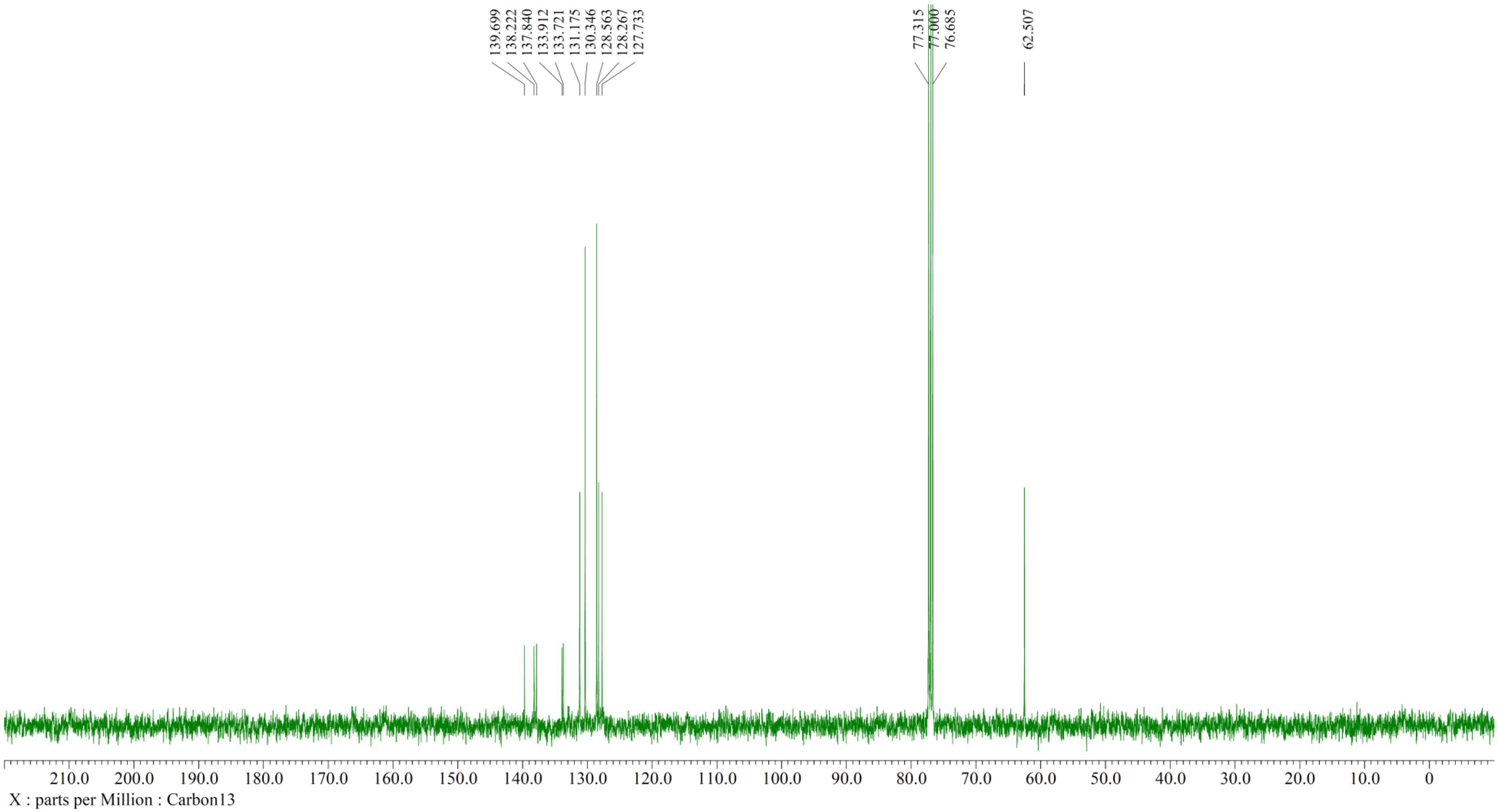

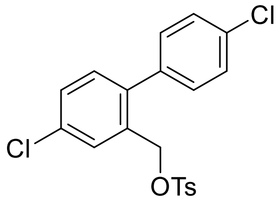

5a

^{1}H NMR (400 MHz, $CDCl_3$)

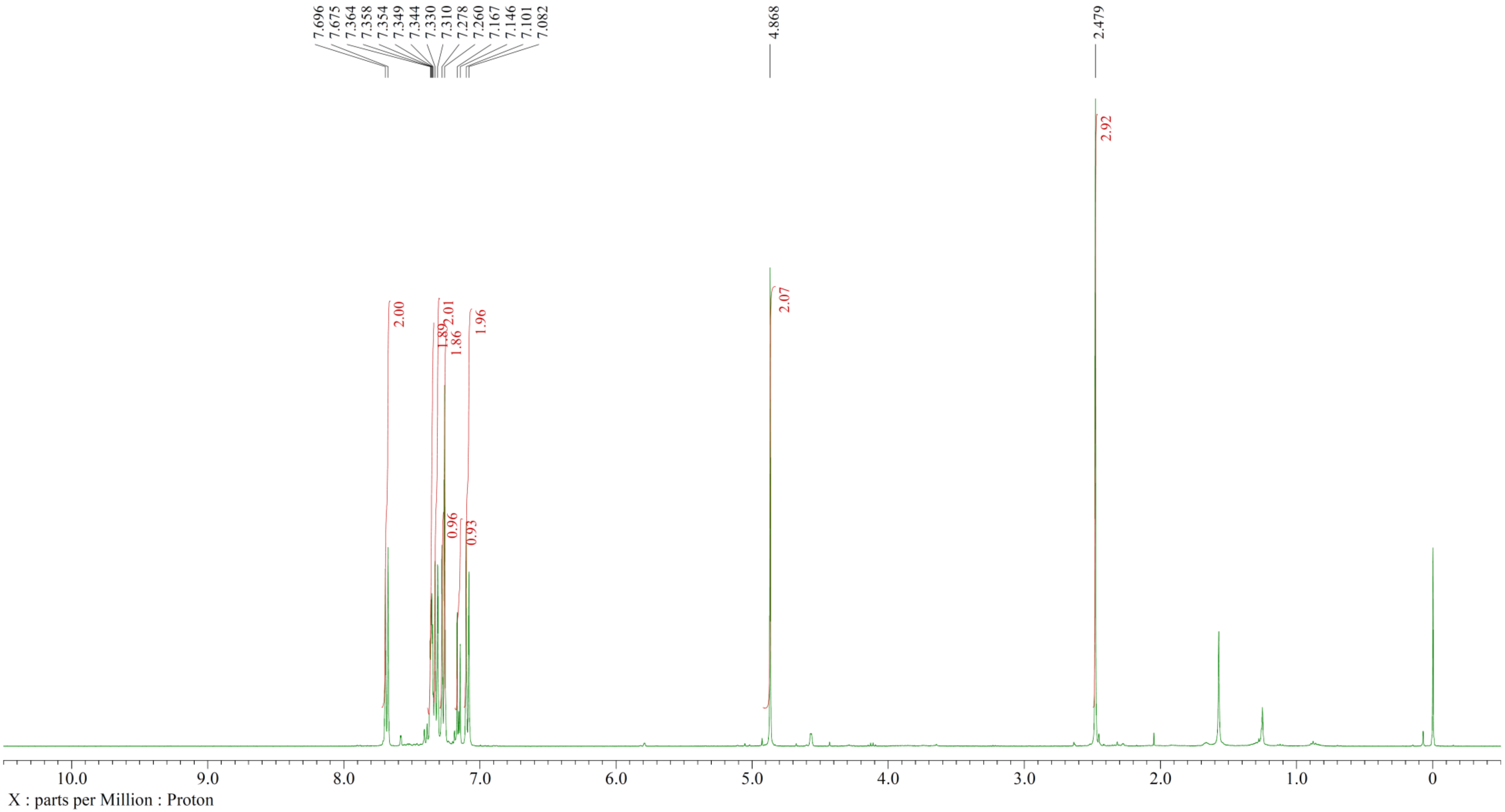

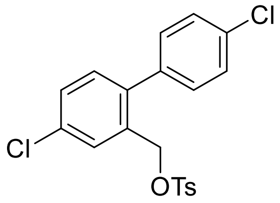

5a

^{13}C NMR (100 MHz, $CDCl_3$)

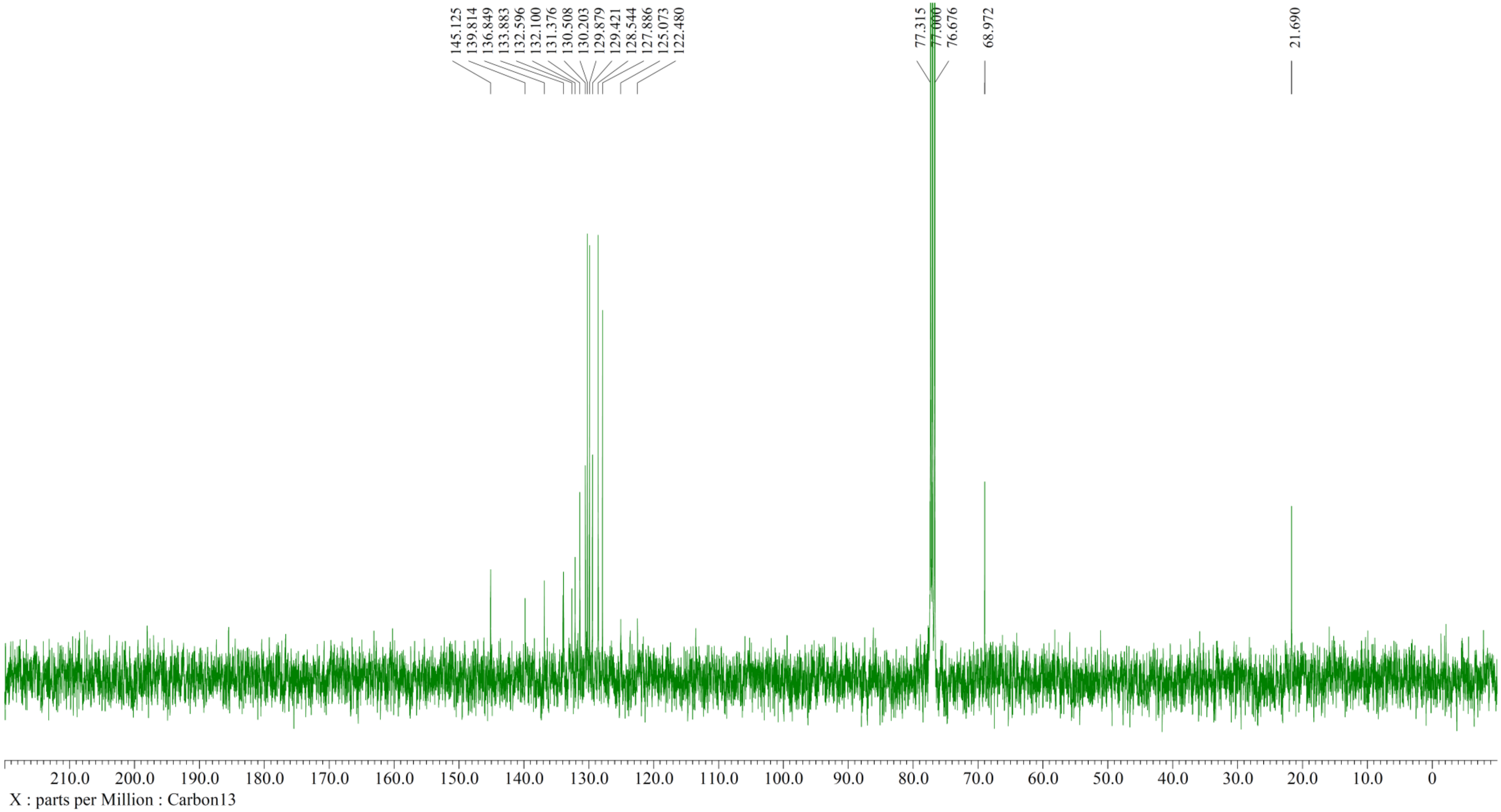

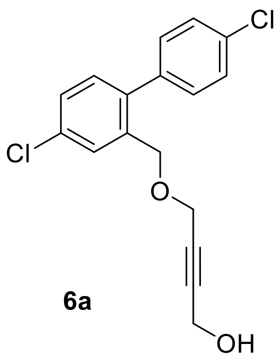

6a

^{1}H NMR (400 MHz, $CDCl_3$)

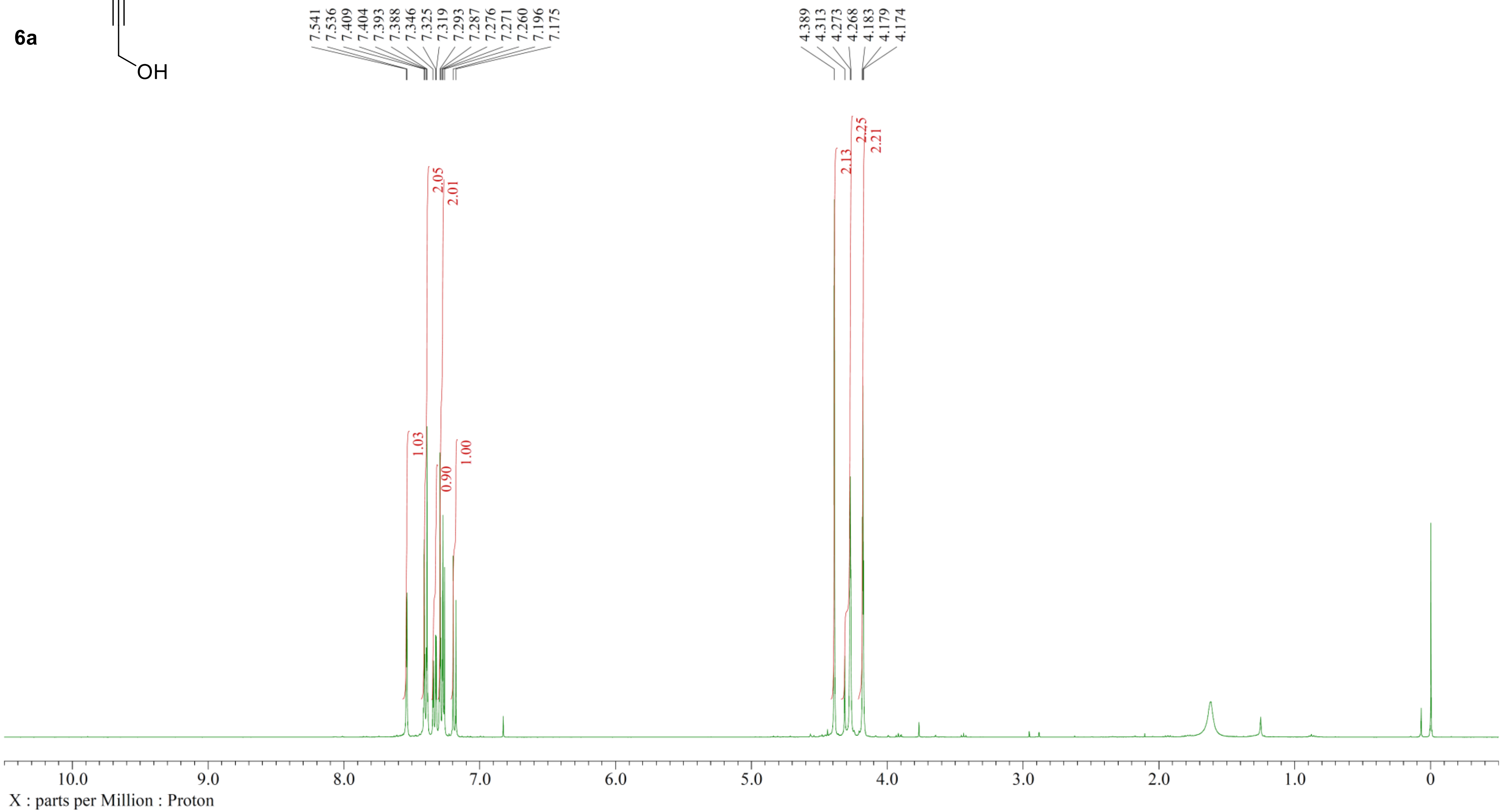

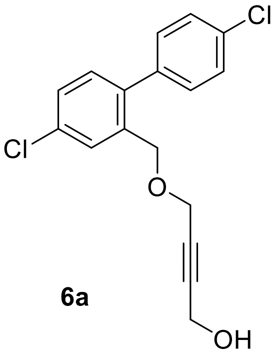

6a

^{13}C NMR (100 MHz, $CDCl_3$)

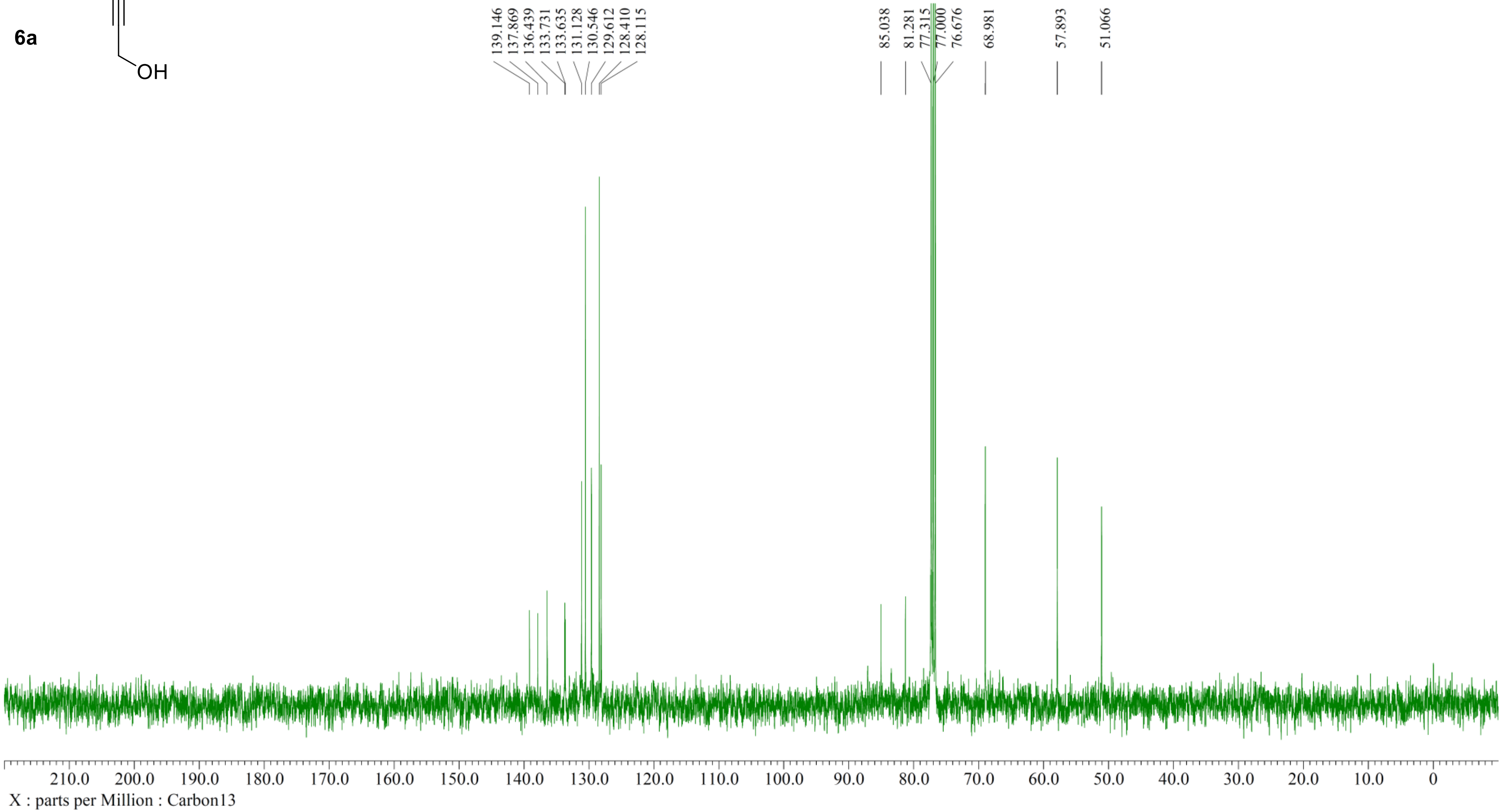

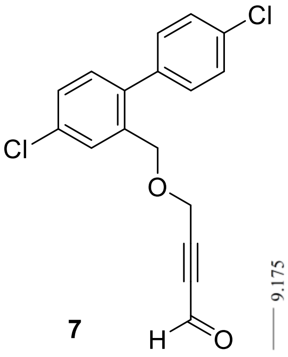

7

^{1}H NMR (400 MHz, $CDCl_3$)

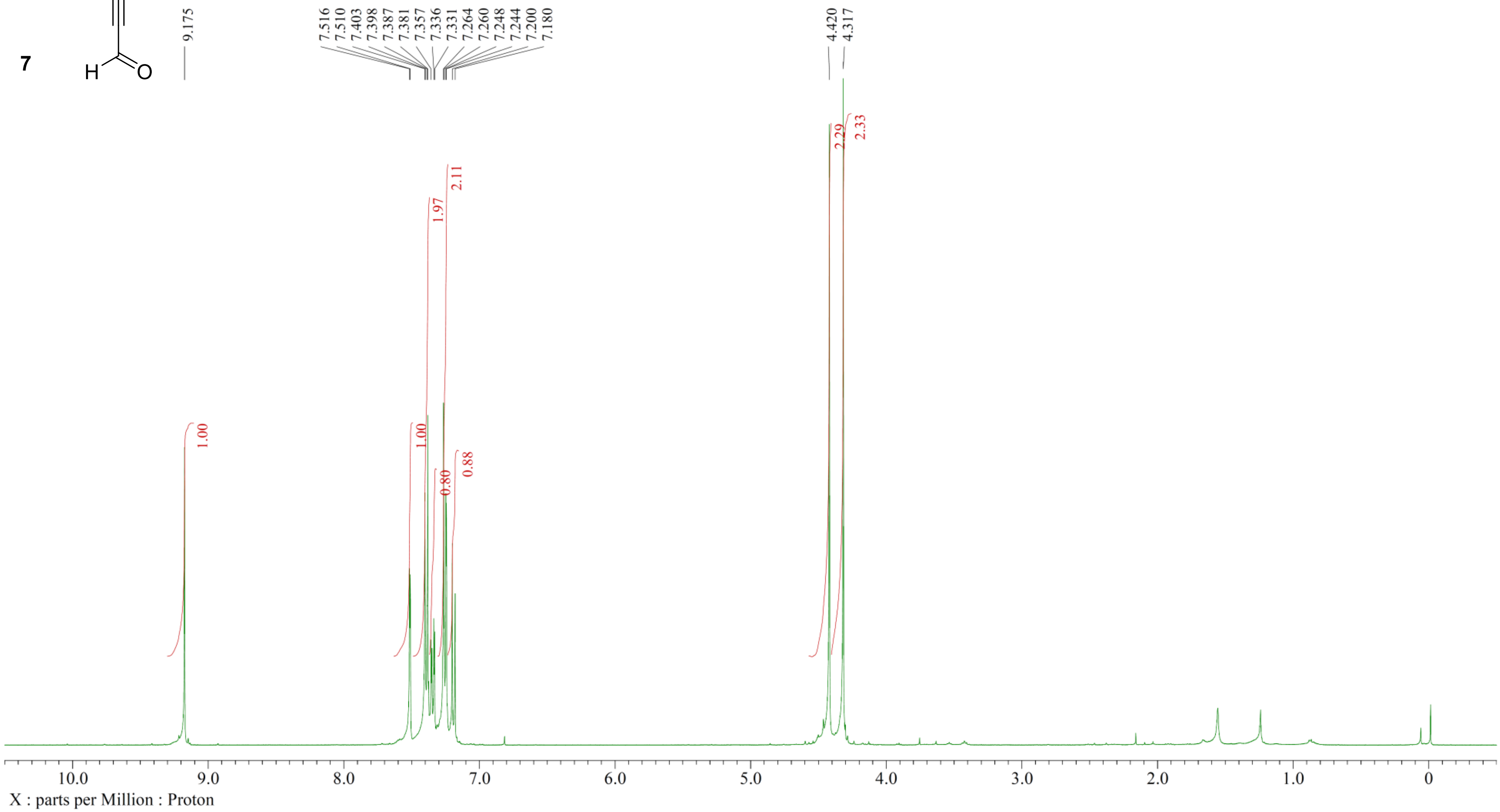

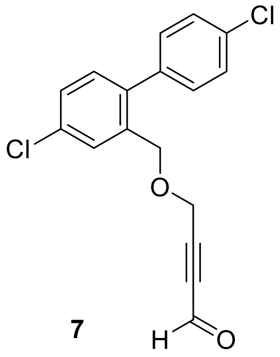

7

^{13}C NMR (100 MHz, $CDCl_3$)

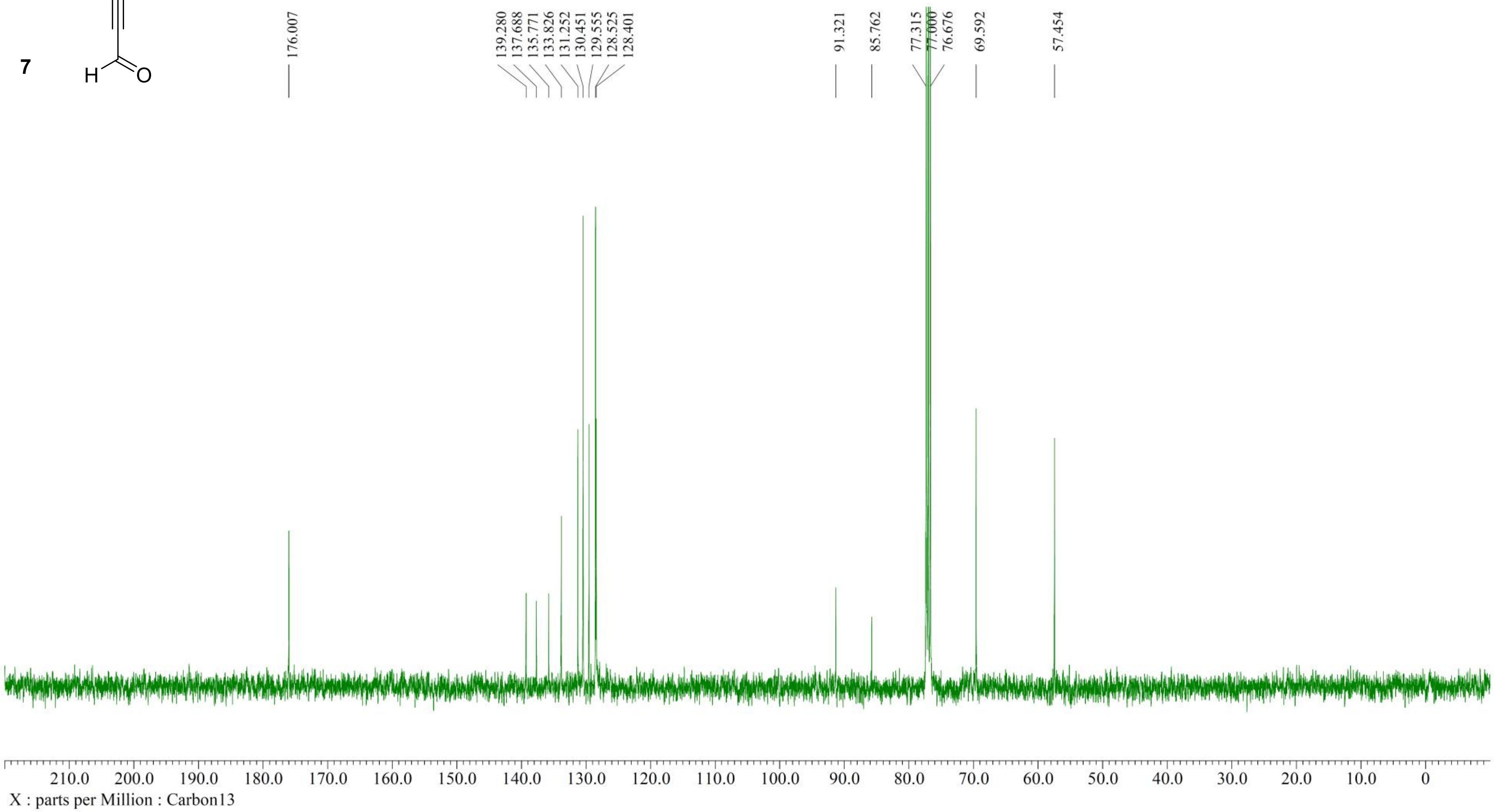

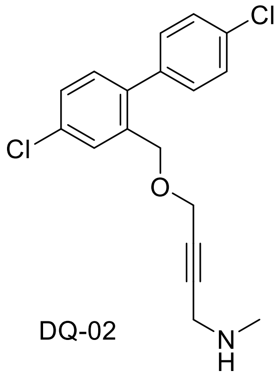

DQ-02

^{1}H NMR (400 MHz, $CDCl_3$)

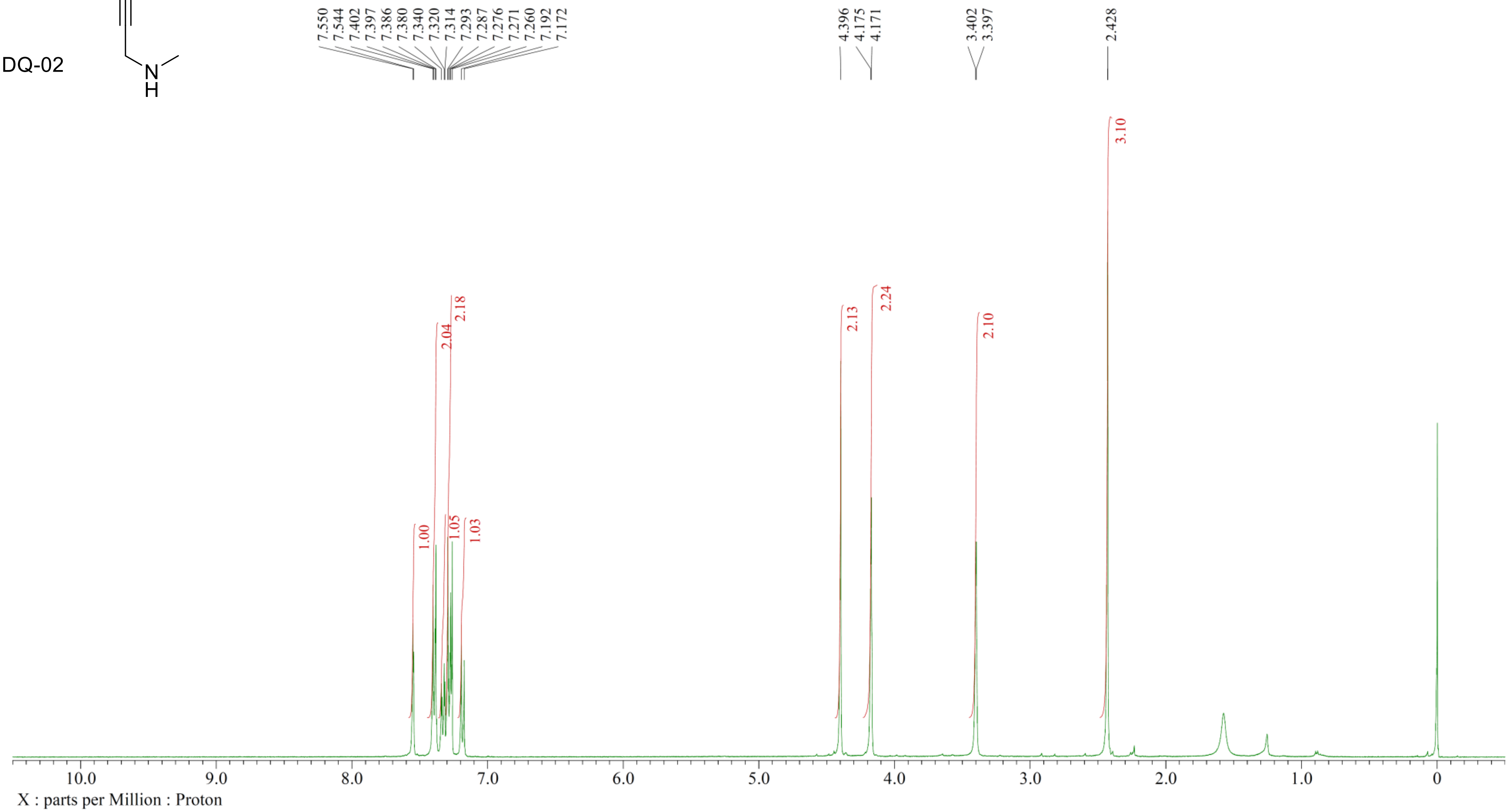

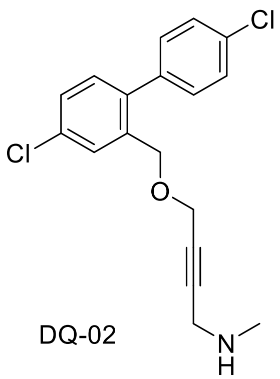

DQ-02

^{13}C NMR (100 MHz, $CDCl_3$)

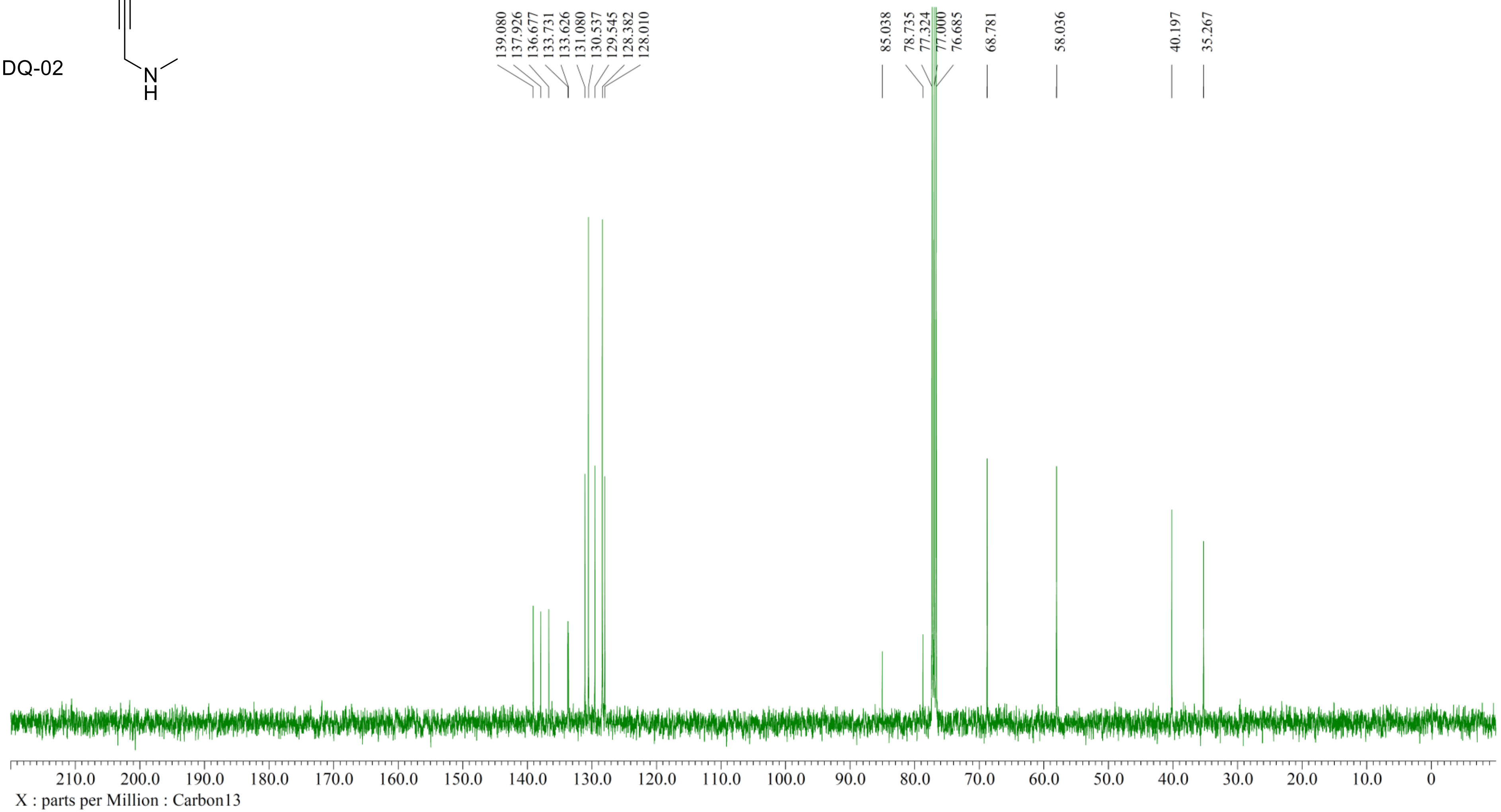

^{1}H NMR (400 MHz, $CDCl_3$)

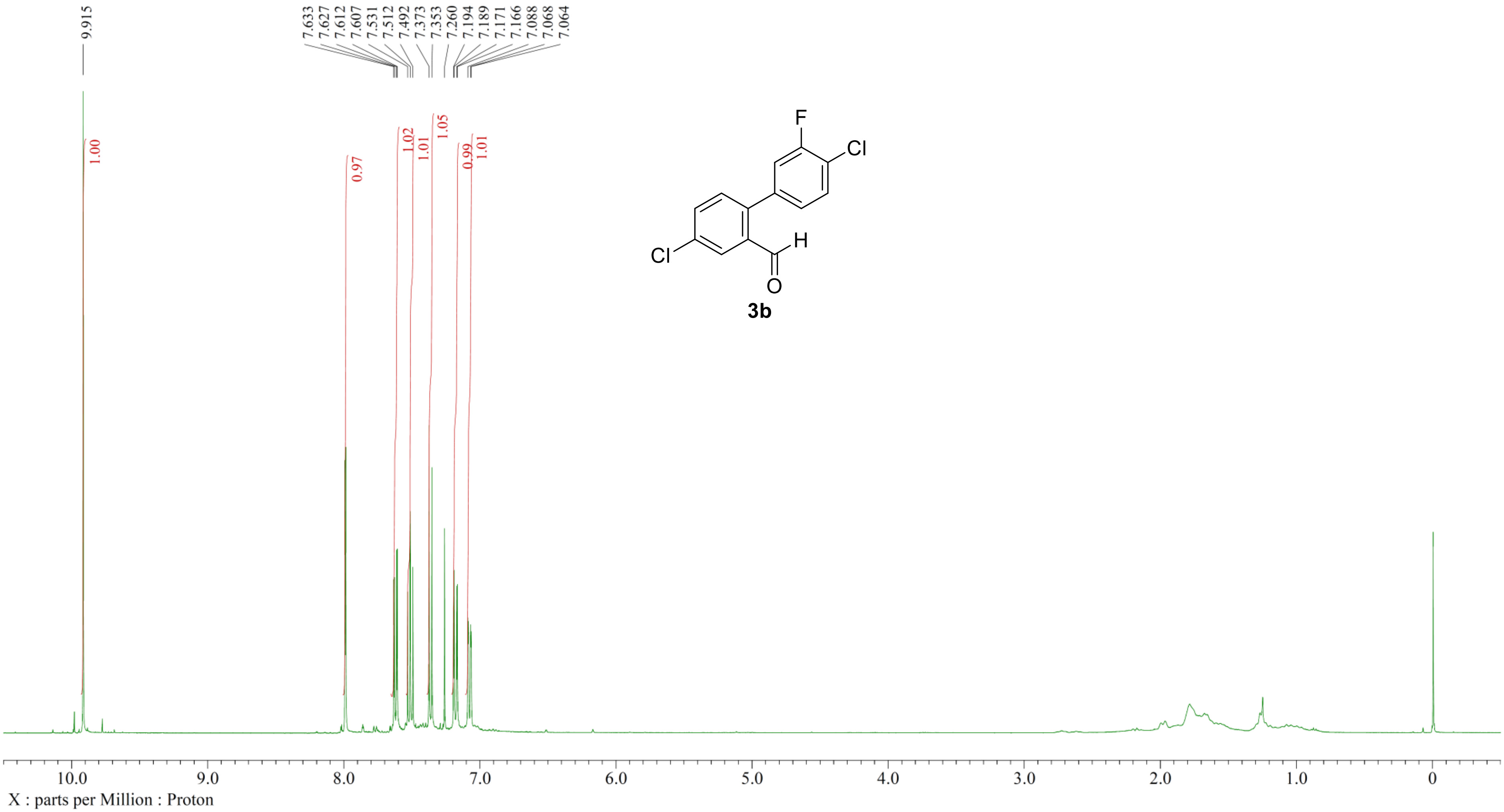

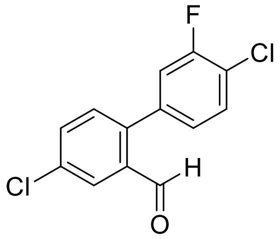

3b

^{13}C NMR (100 MHz, $CDCl_3$)

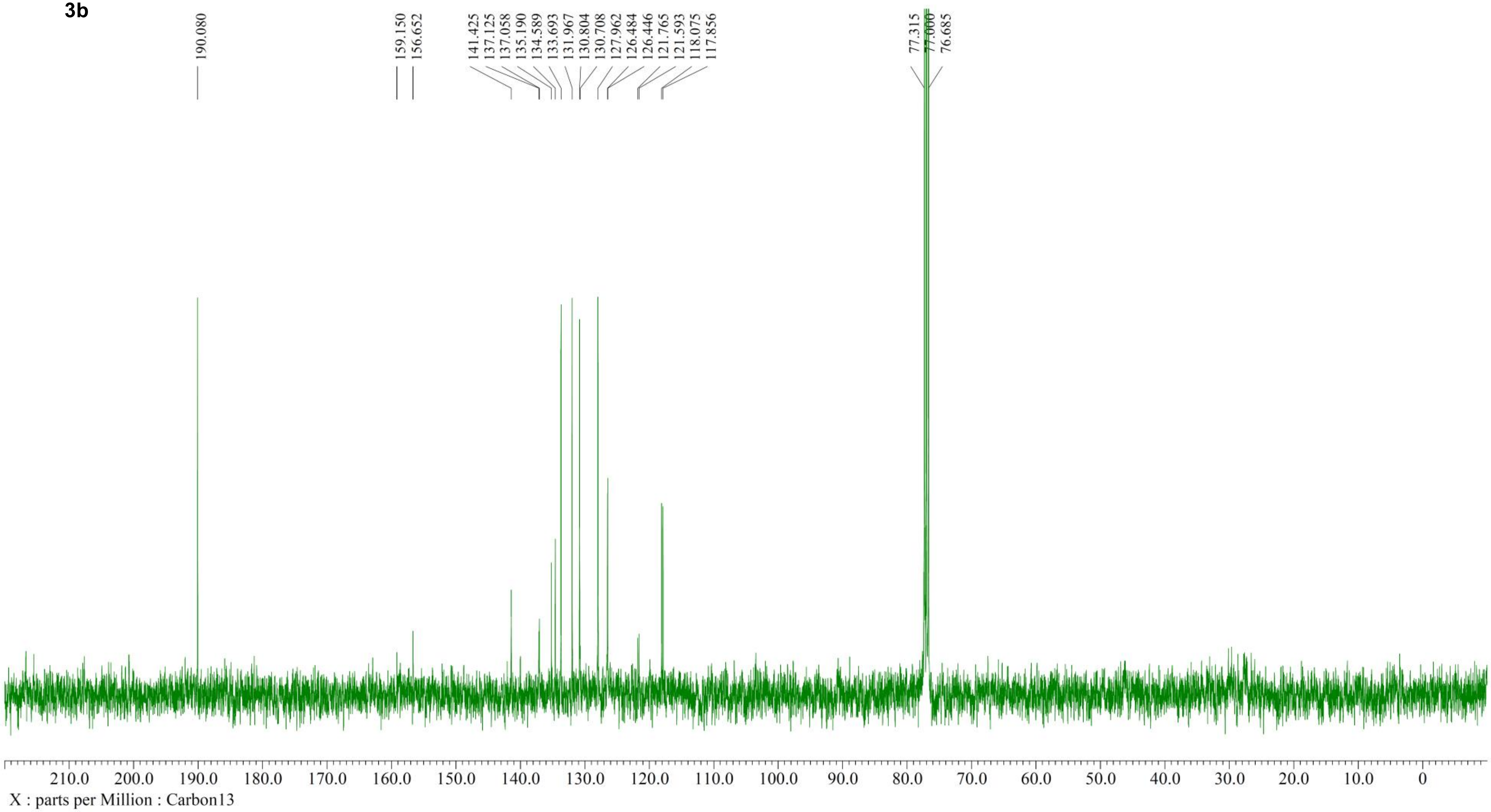

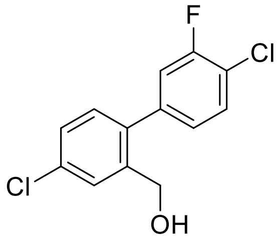

4b

¹H NMR (400 MHz, CDCl₃)

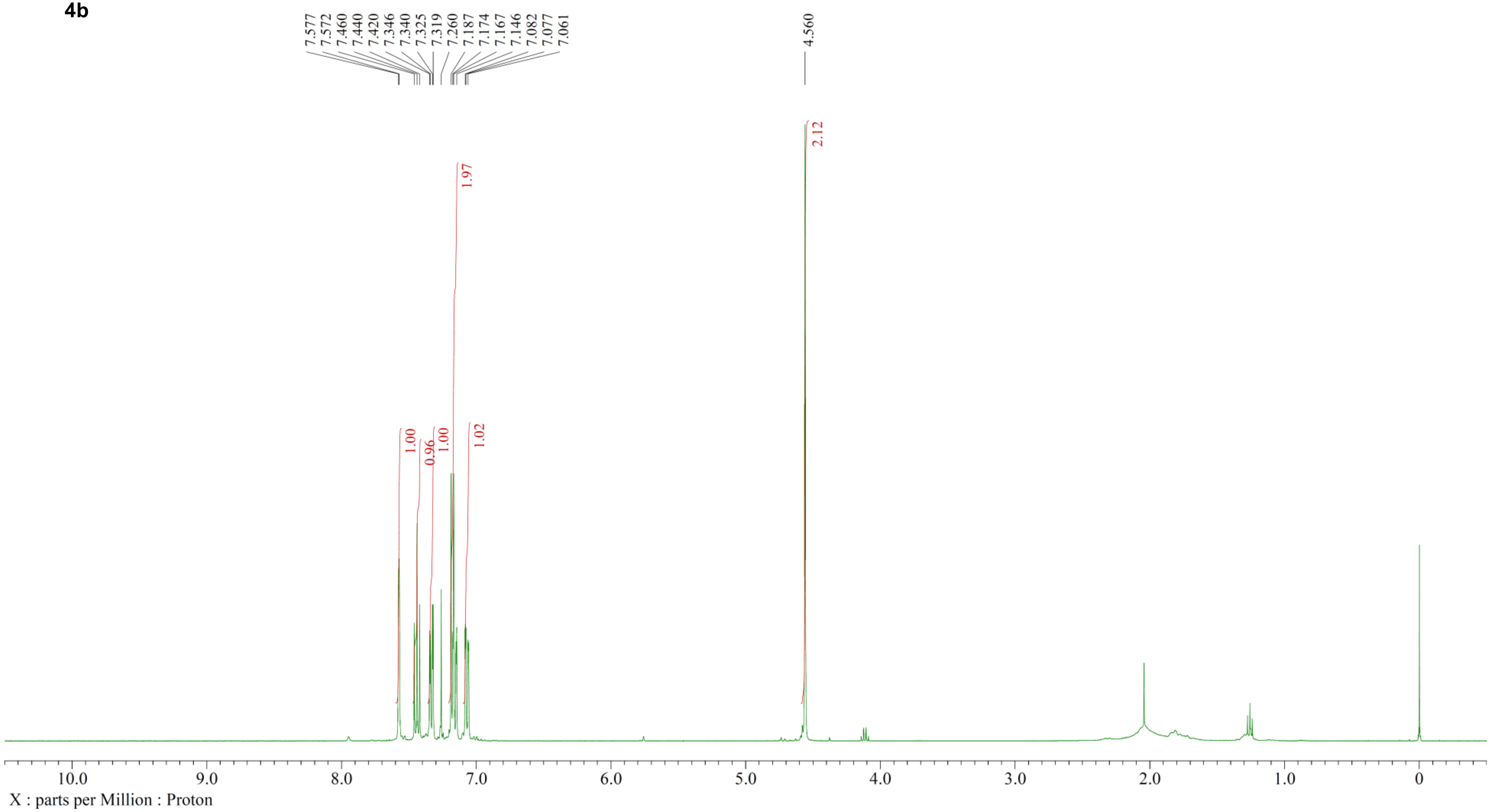

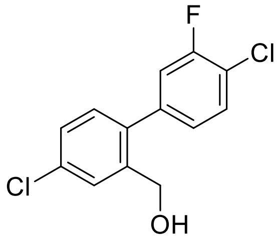

4b

^{13}C NMR (100 MHz, $CDCl_3$)

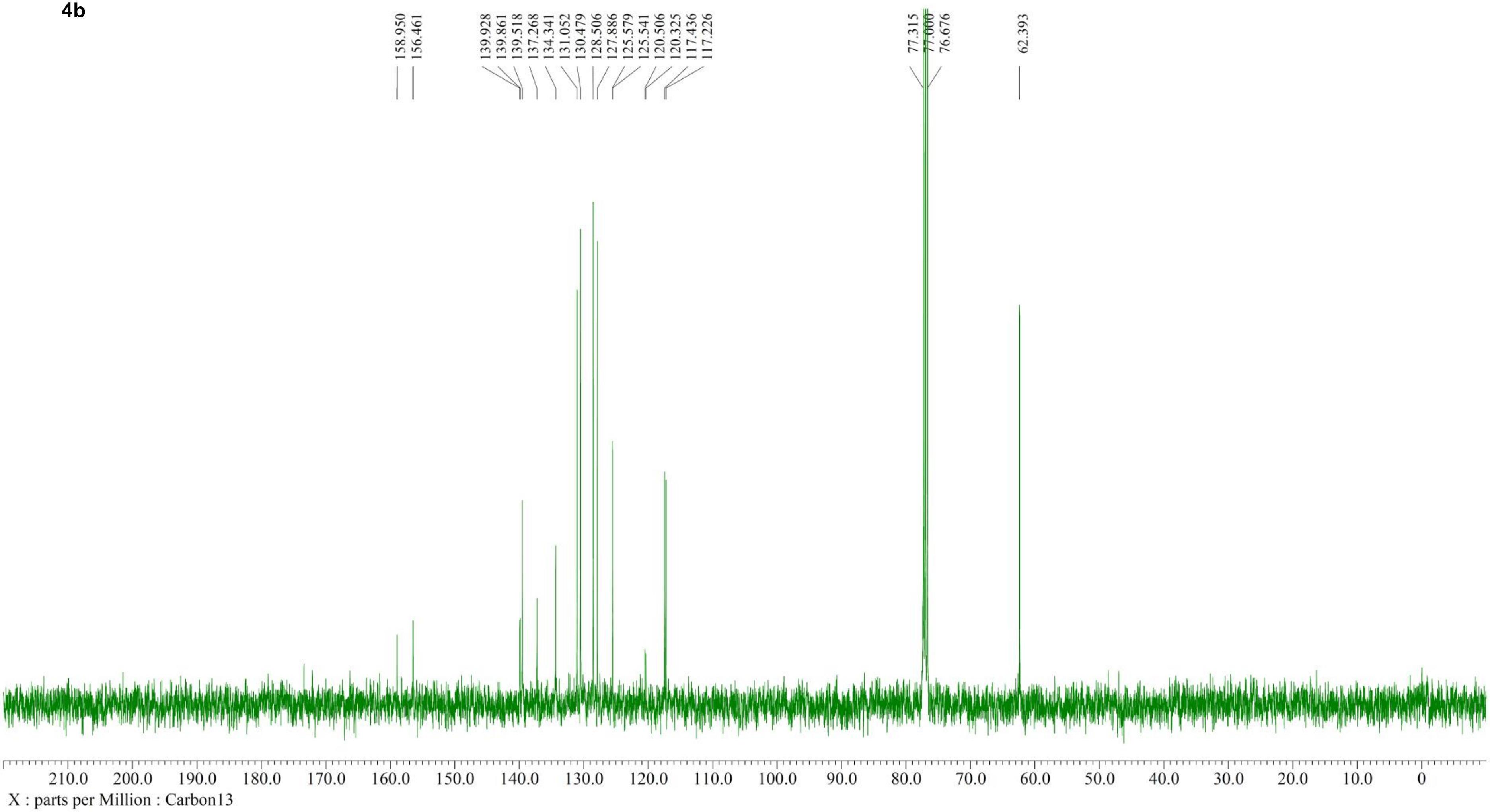

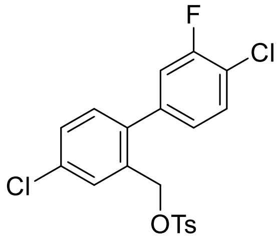

5b

^{1}H NMR (400 MHz, $CDCl_3$)

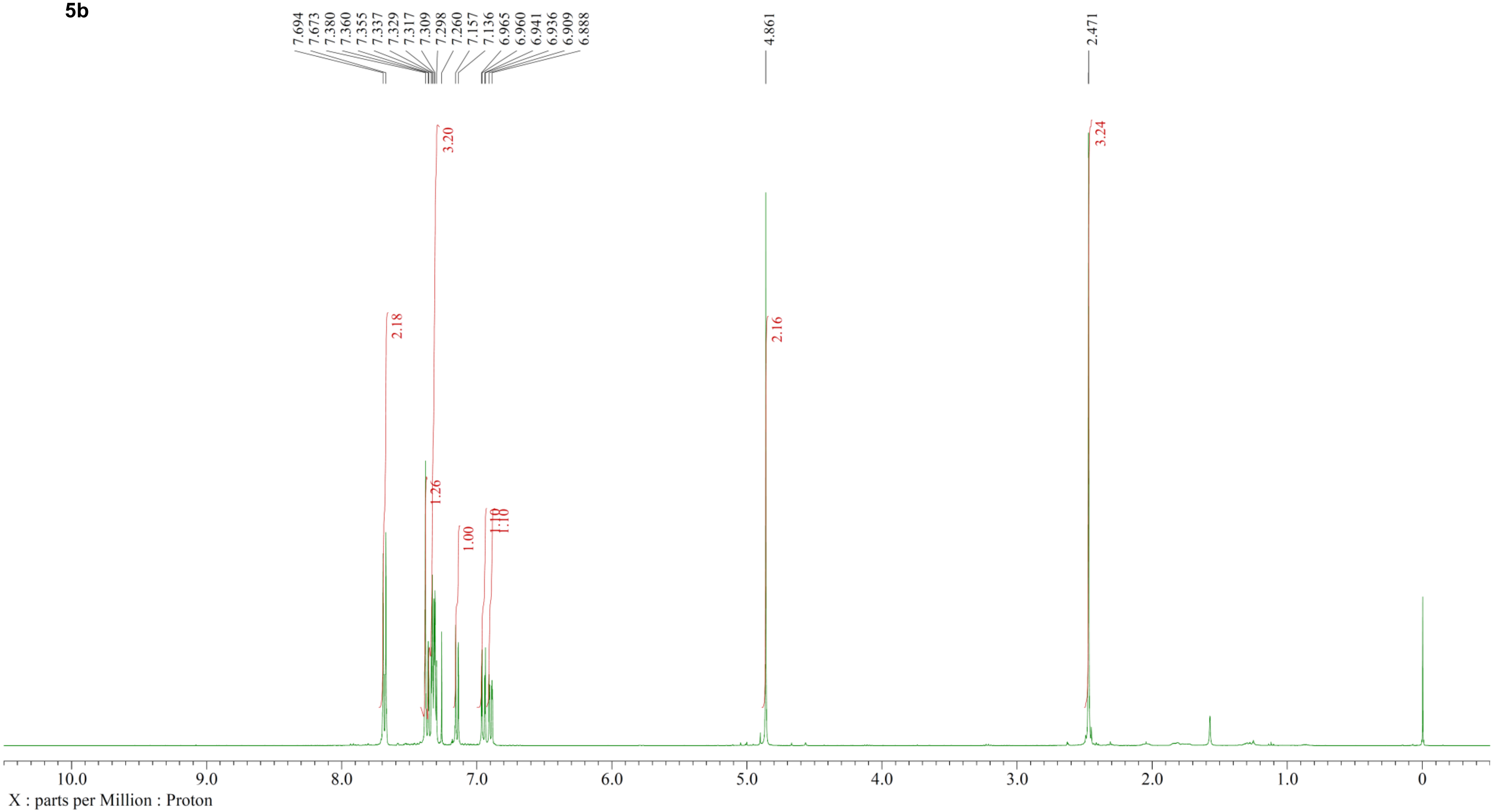

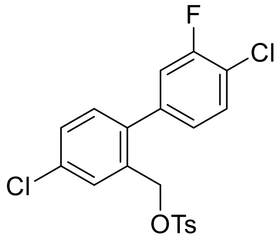

5b

^{13}C NMR (100 MHz, $CDCl_3$)

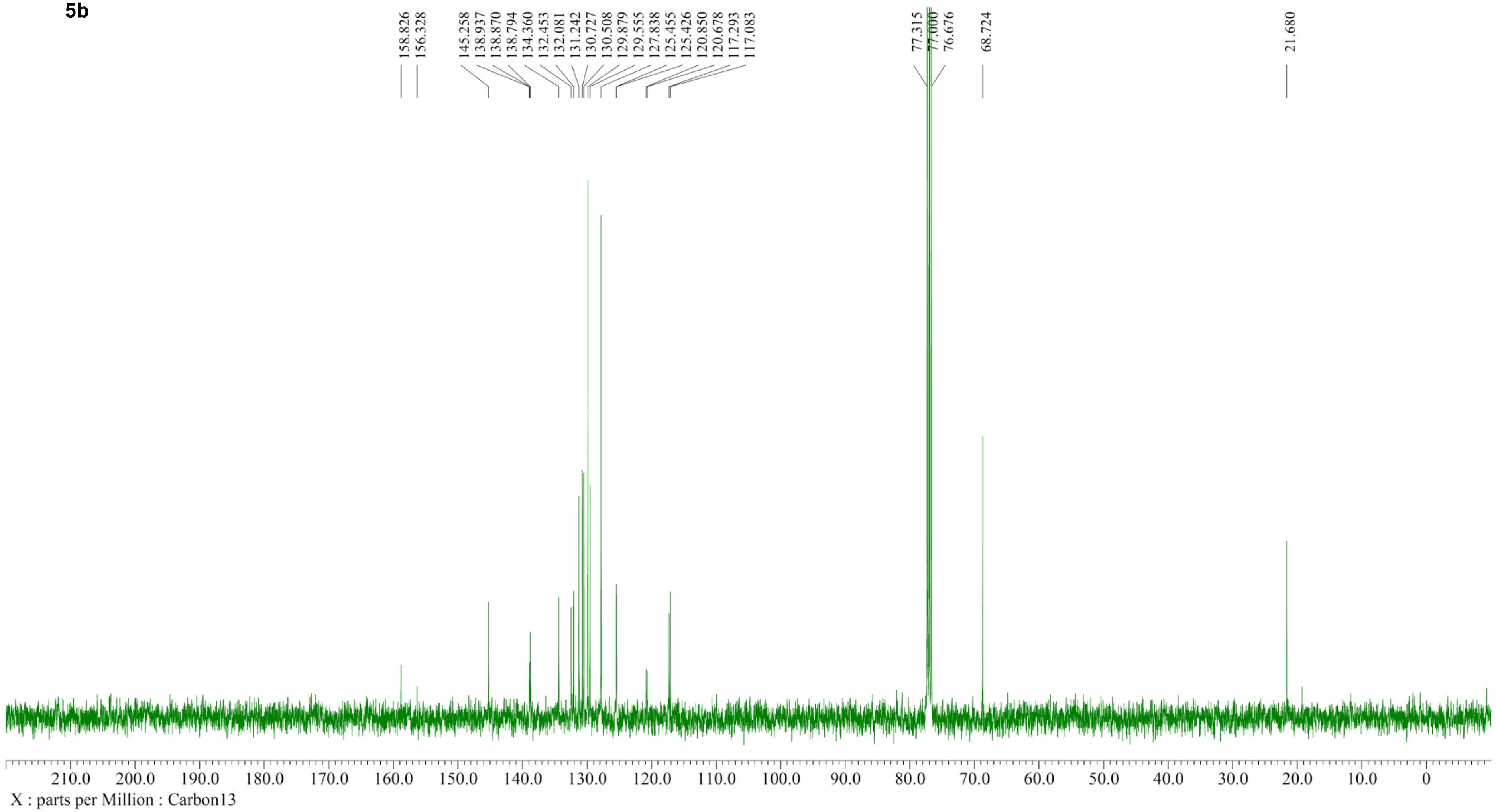

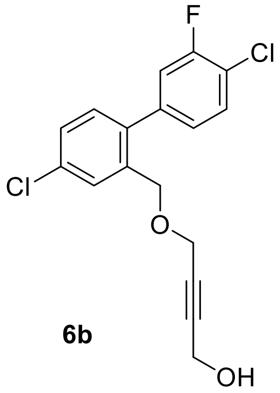

6b

^1H NMR (400 MHz, $CDCl_3$)

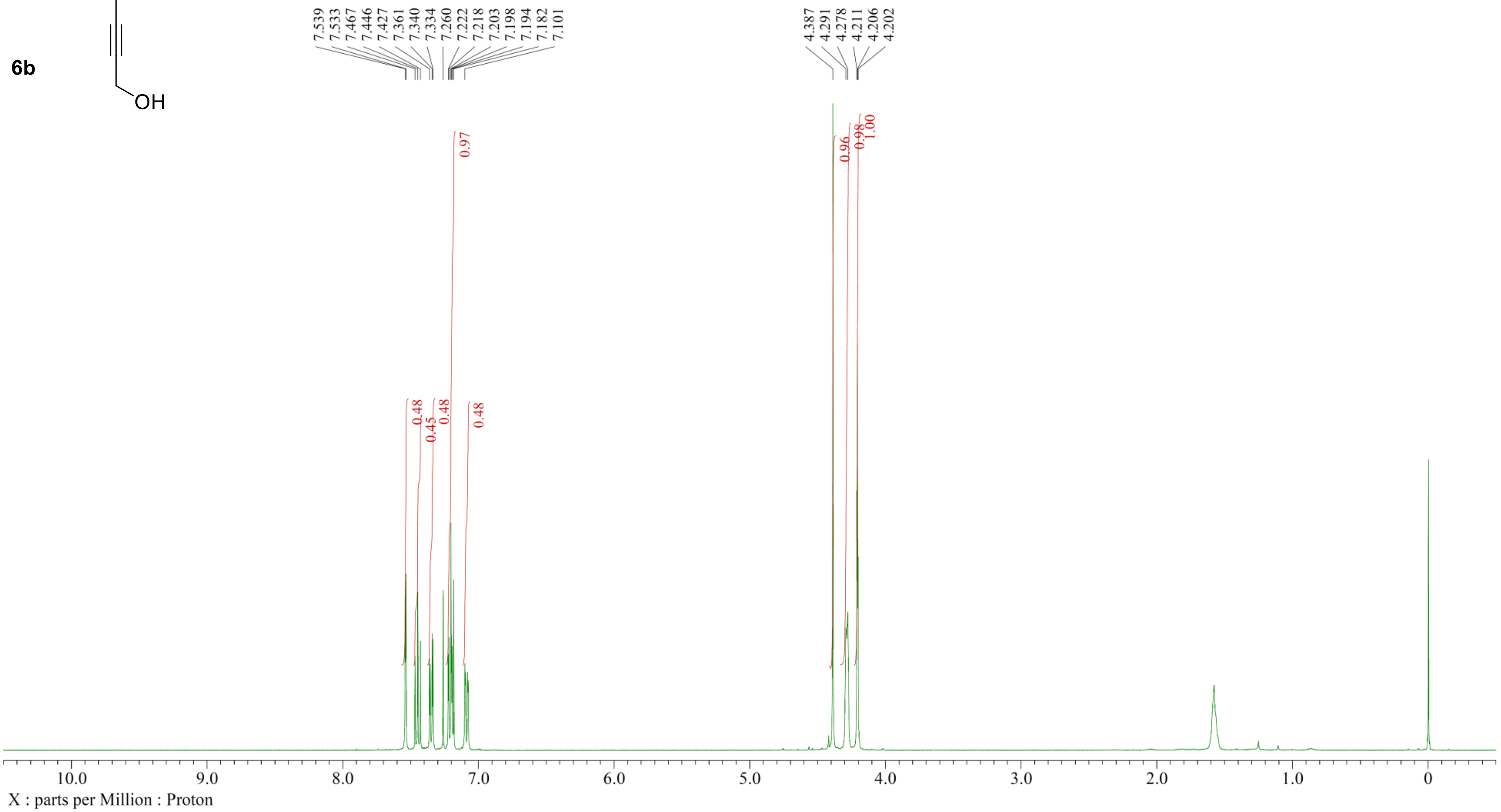

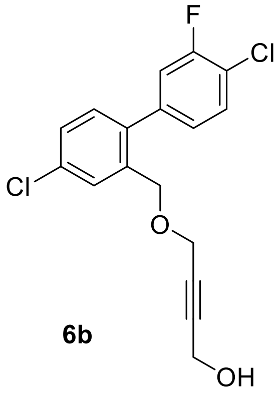

6b

^{13}C NMR (100 MHz, $CDCl_3$)

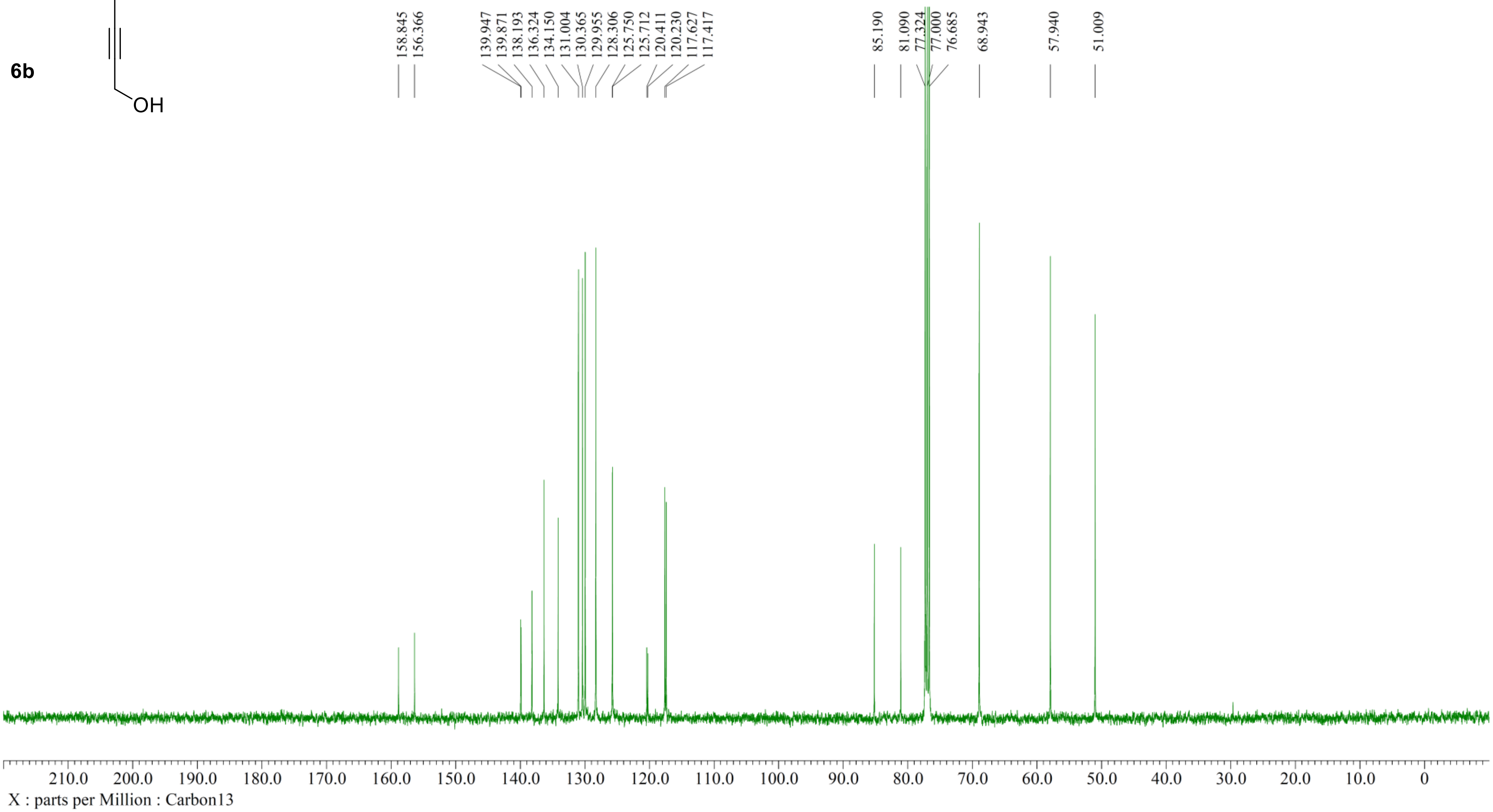

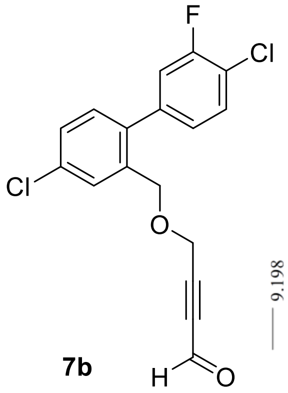

7b

^{1}H NMR (400 MHz, $CDCl_3$)

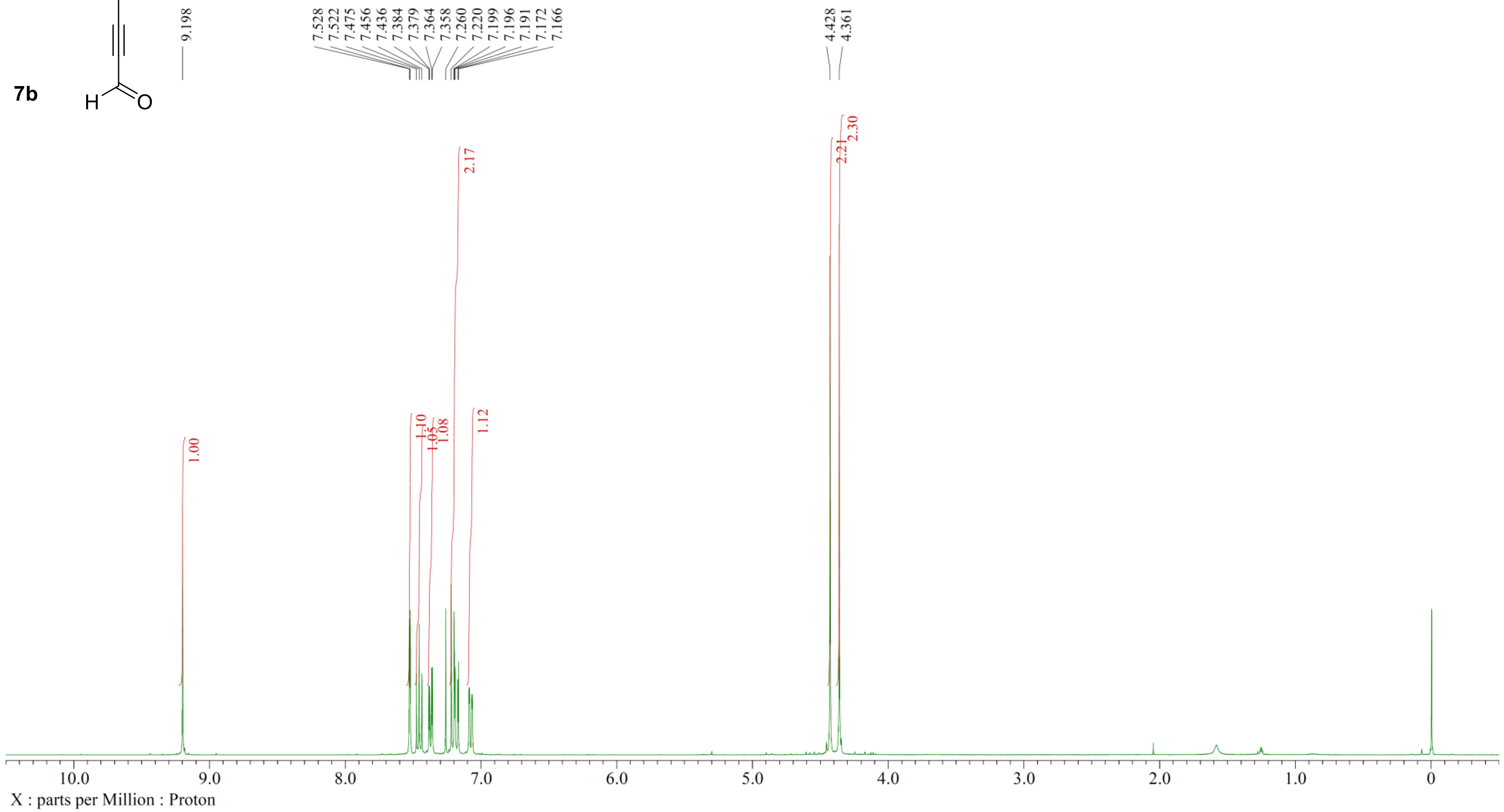

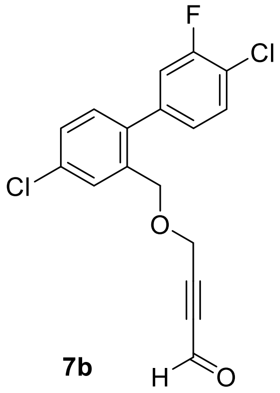

7b

^{13}C NMR (100 MHz, $CDCl_3$)

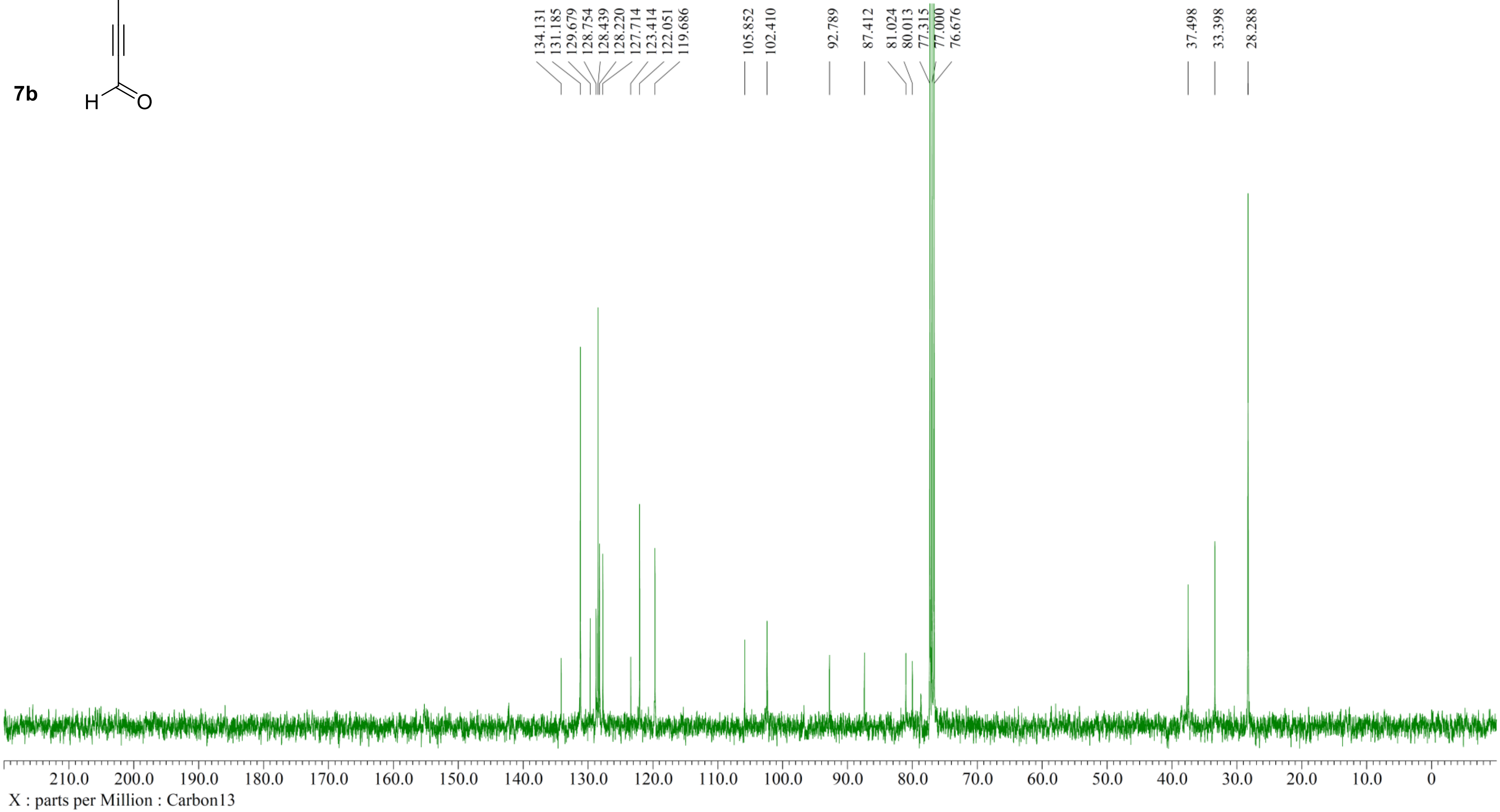

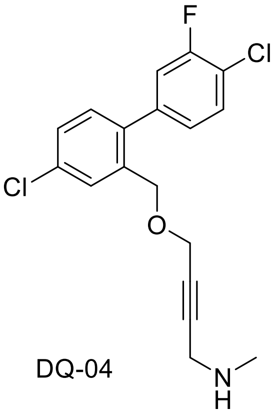

DQ-04

^{1}H NMR (400 MHz, $CDCl_3$)

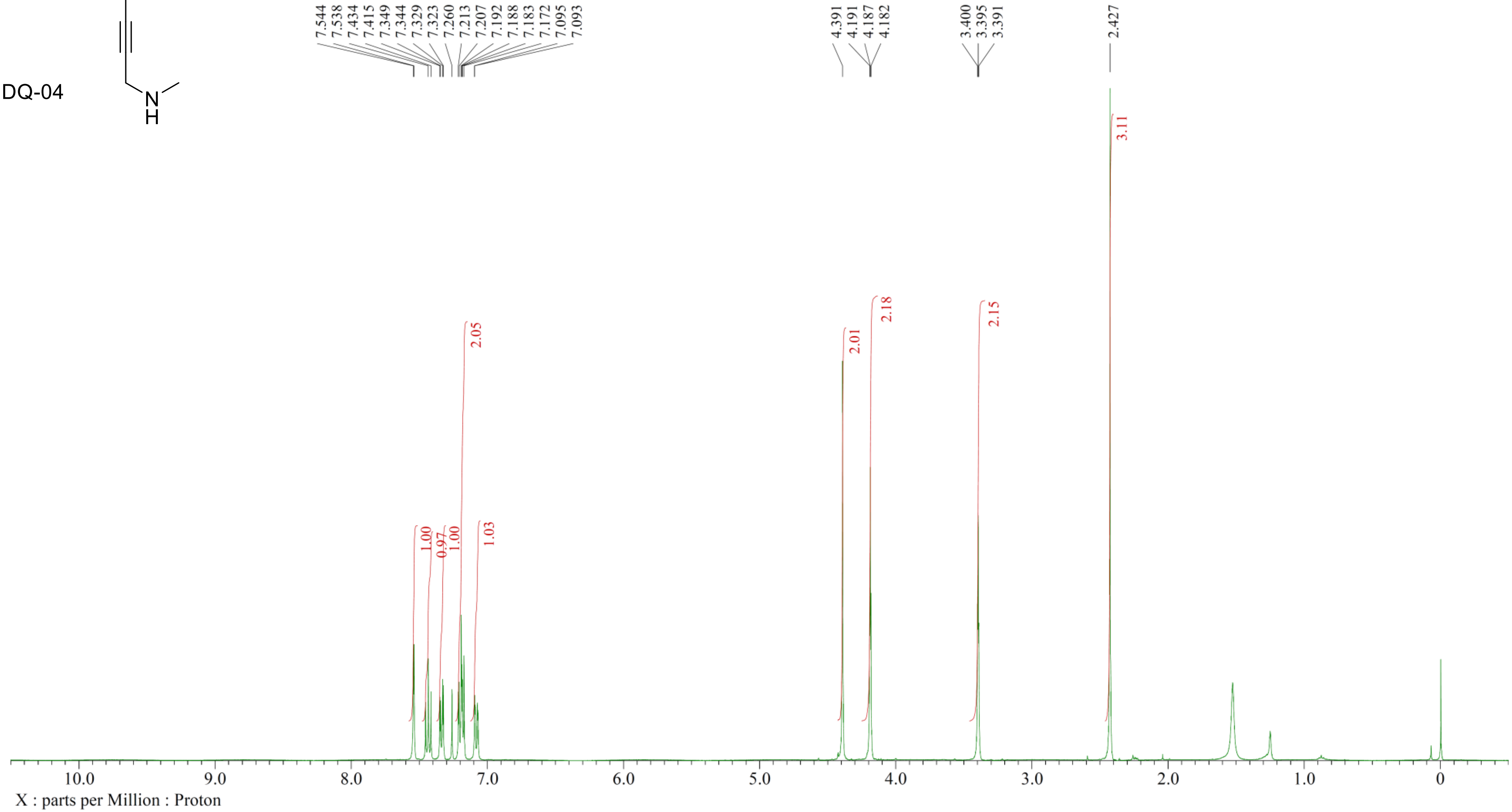

^{13}C NMR (100 MHz, $CDCl_3$)

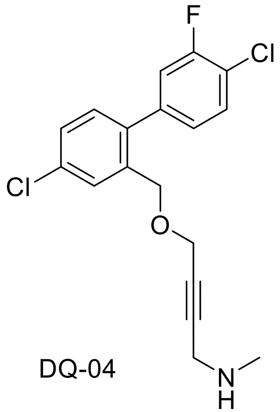

DQ-04

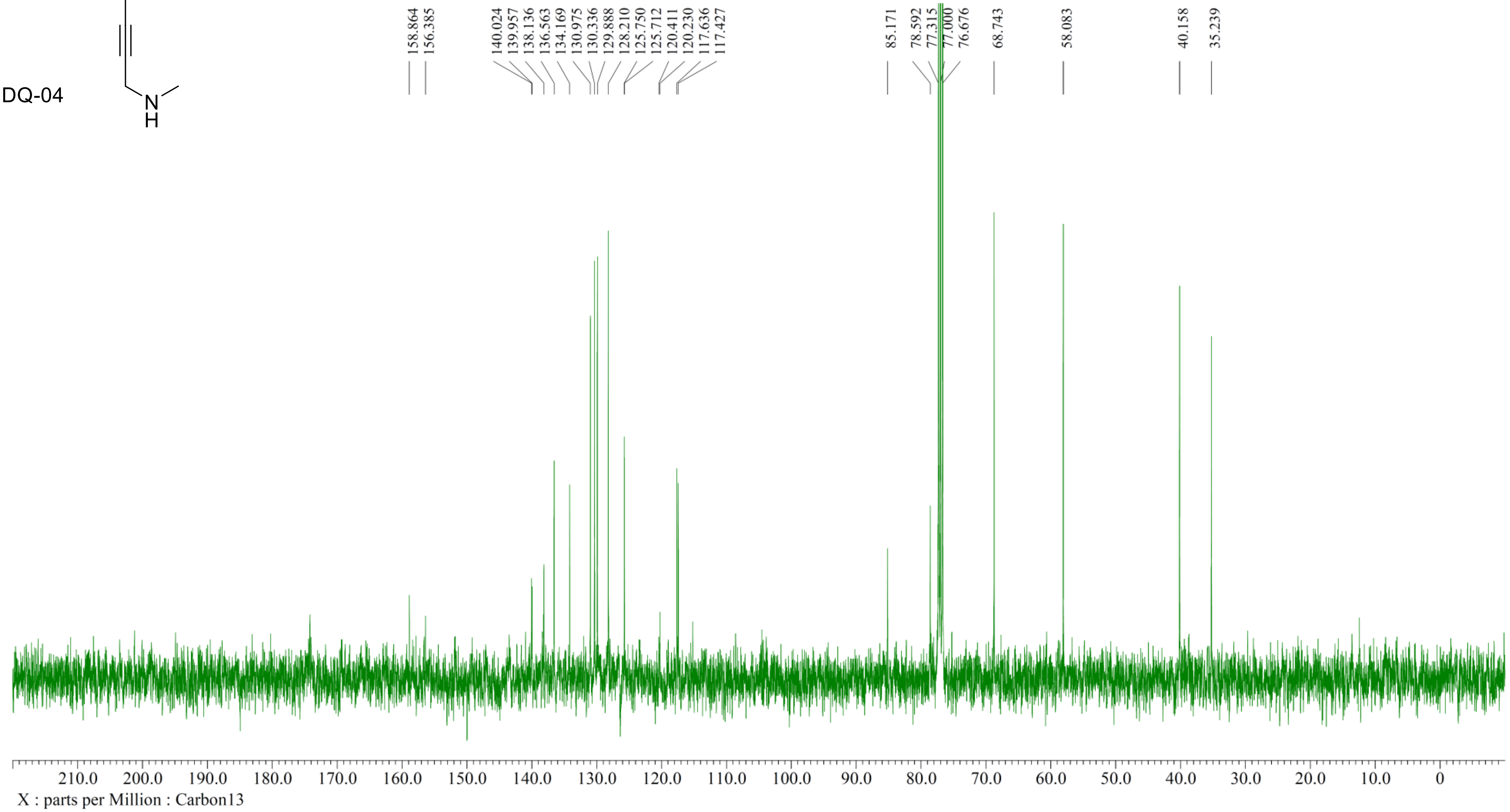

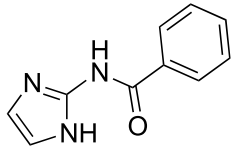

9

^{1}H NMR (400 MHz, DMSO-d_6)

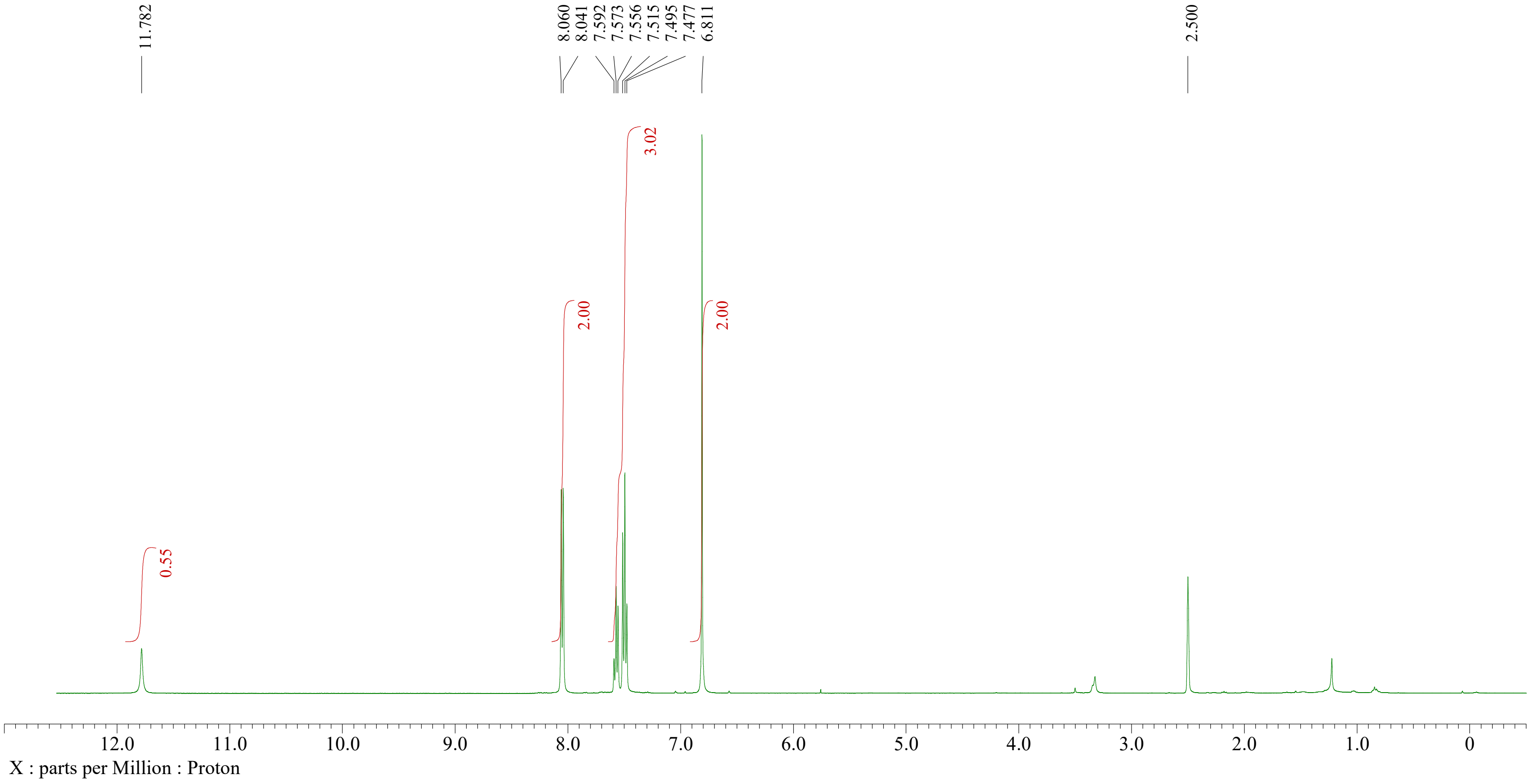

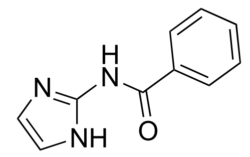

9

^{13}C NMR (100 MHz, DMSO-d_6)

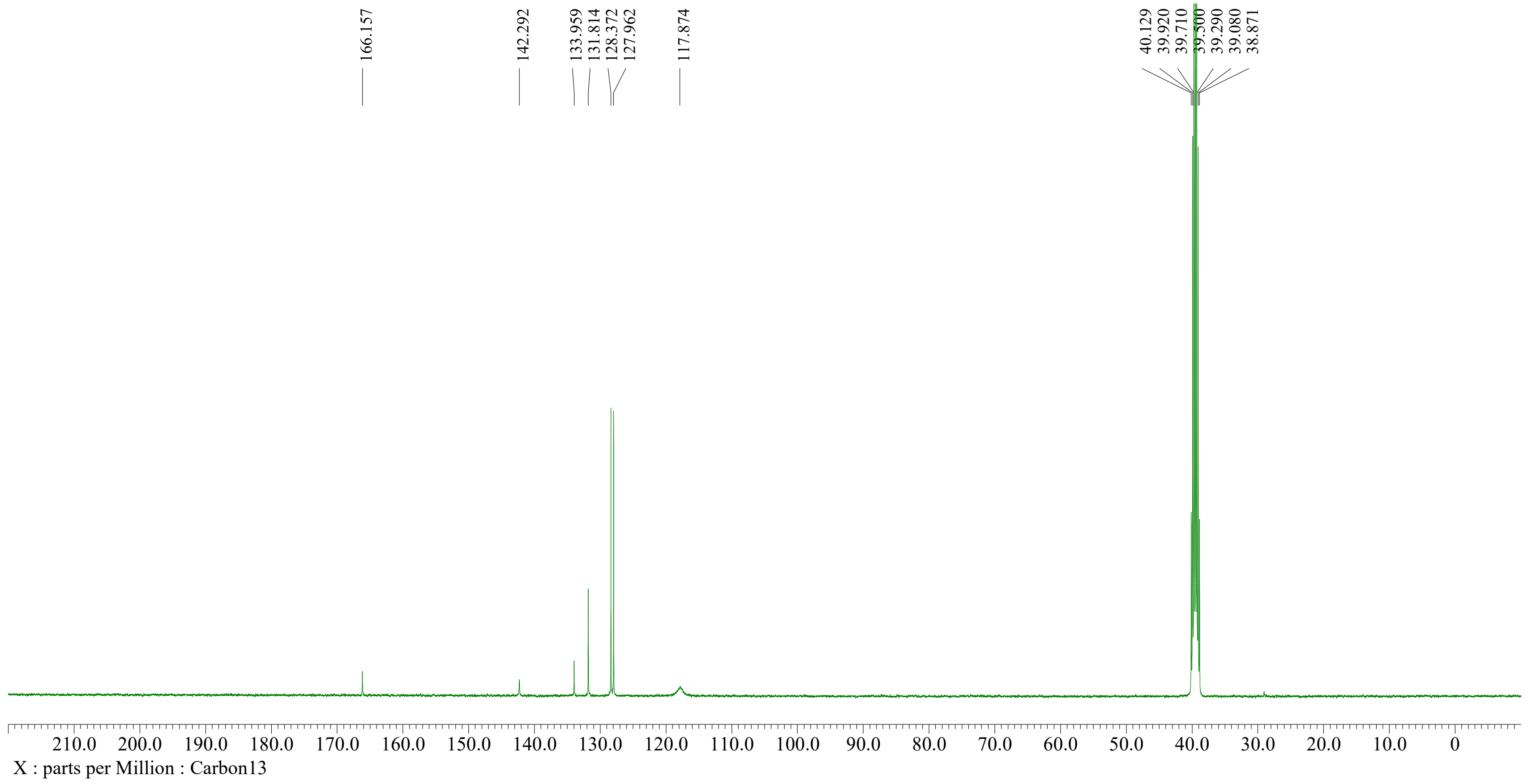

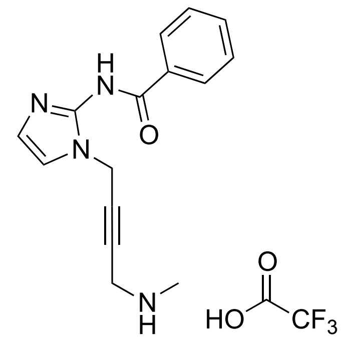

^{1}H NMR (400 MHz, CD_3OD)

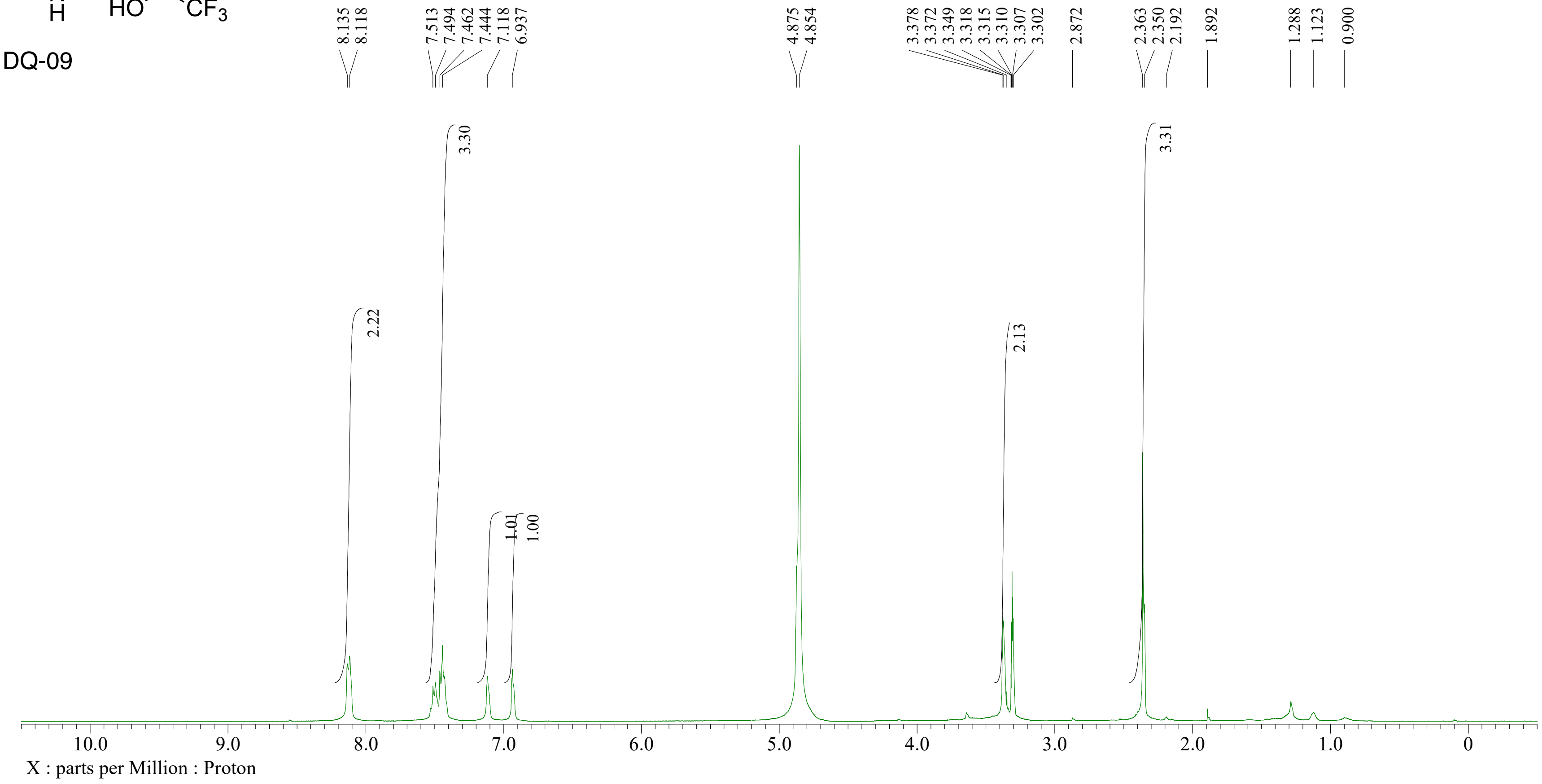

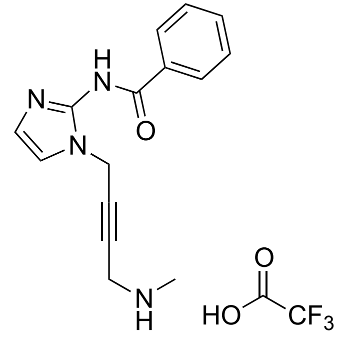

DQ-09

^{13}C NMR (100 MHz, CD_3OD)

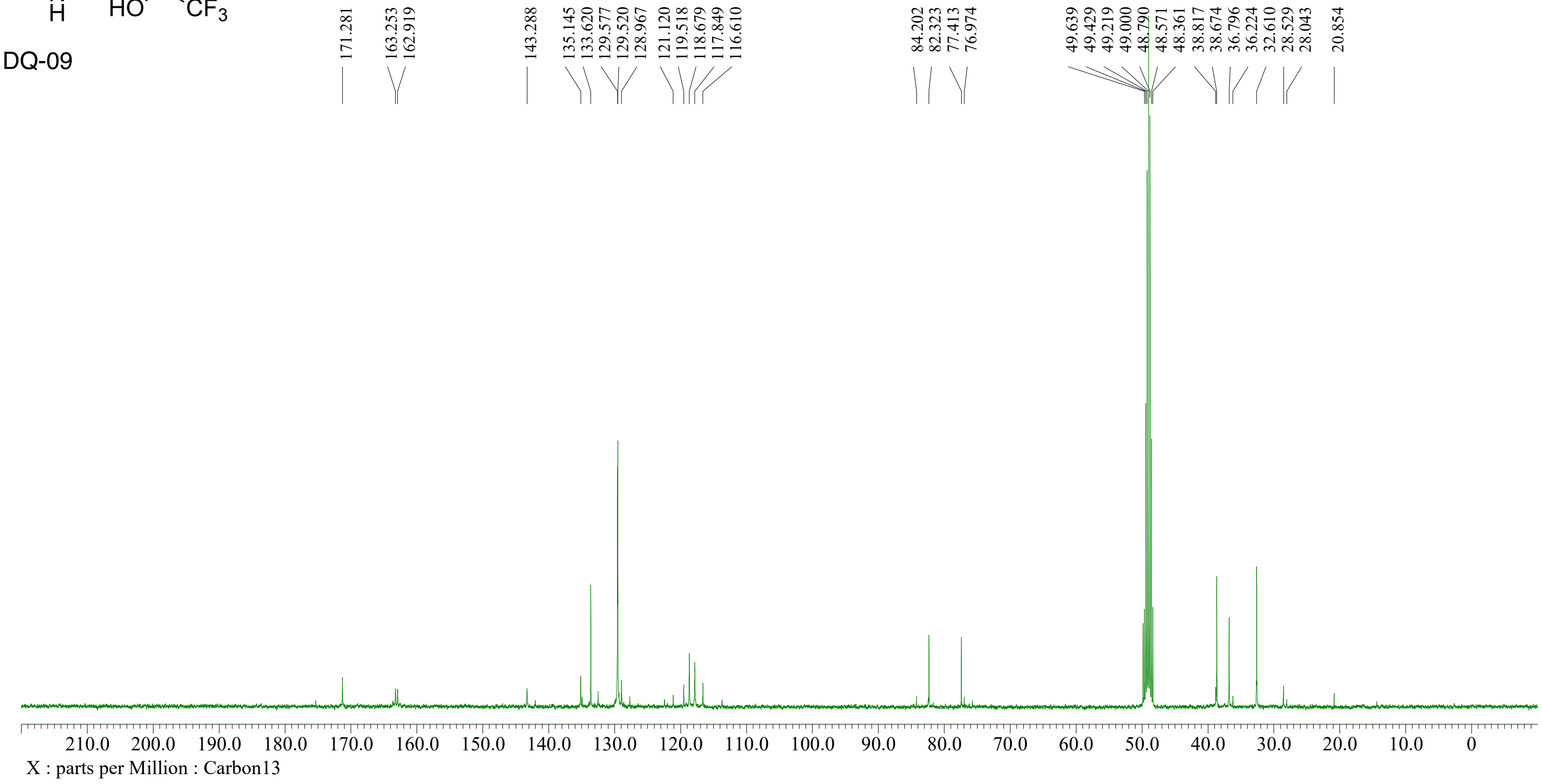

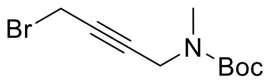

10

¹H NMR (400 MHz, $CDCl_3$)

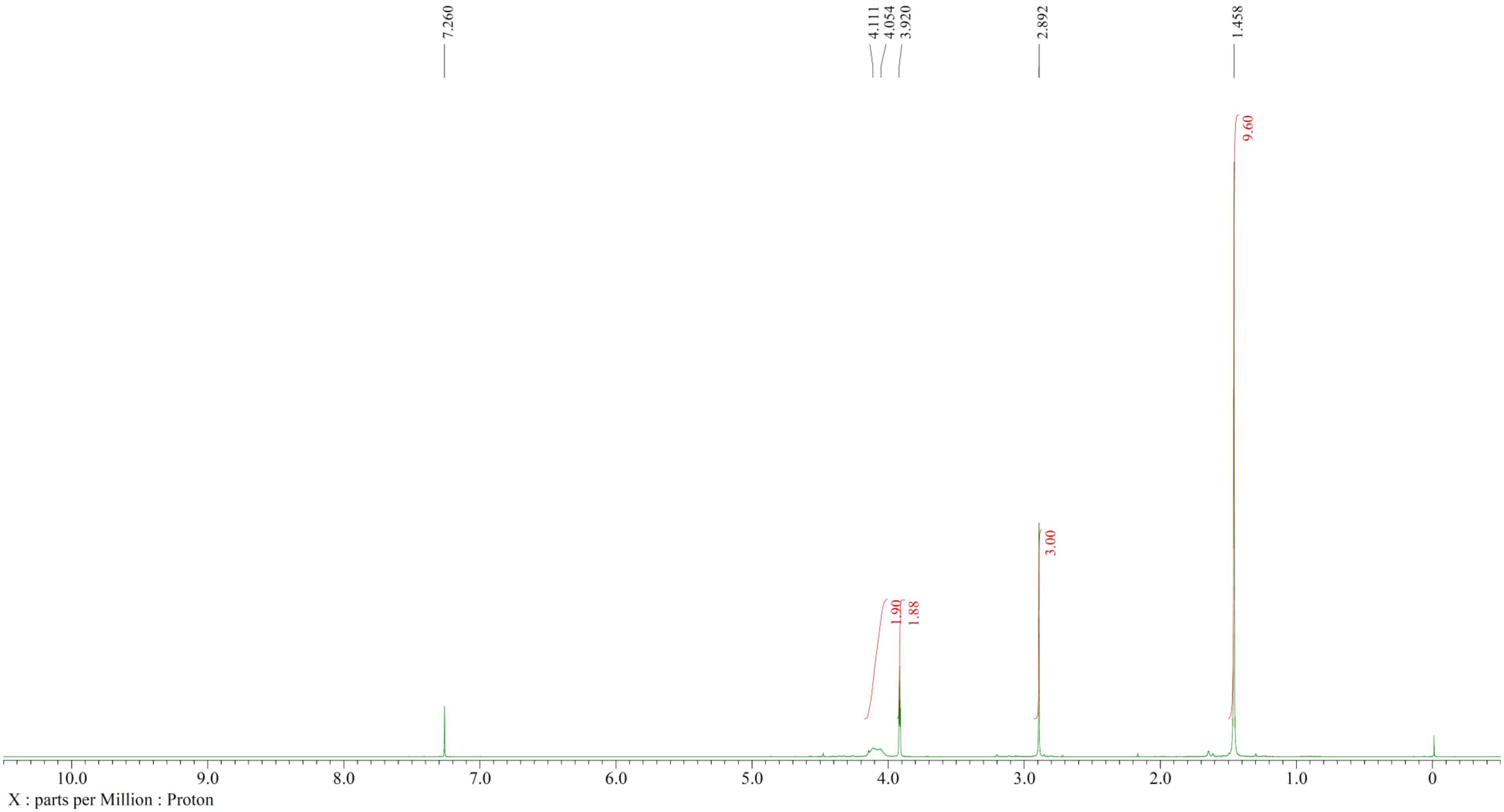

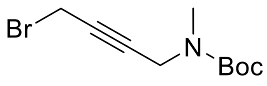

10

^{13}C NMR (100 MHz, $CDCl_3$)

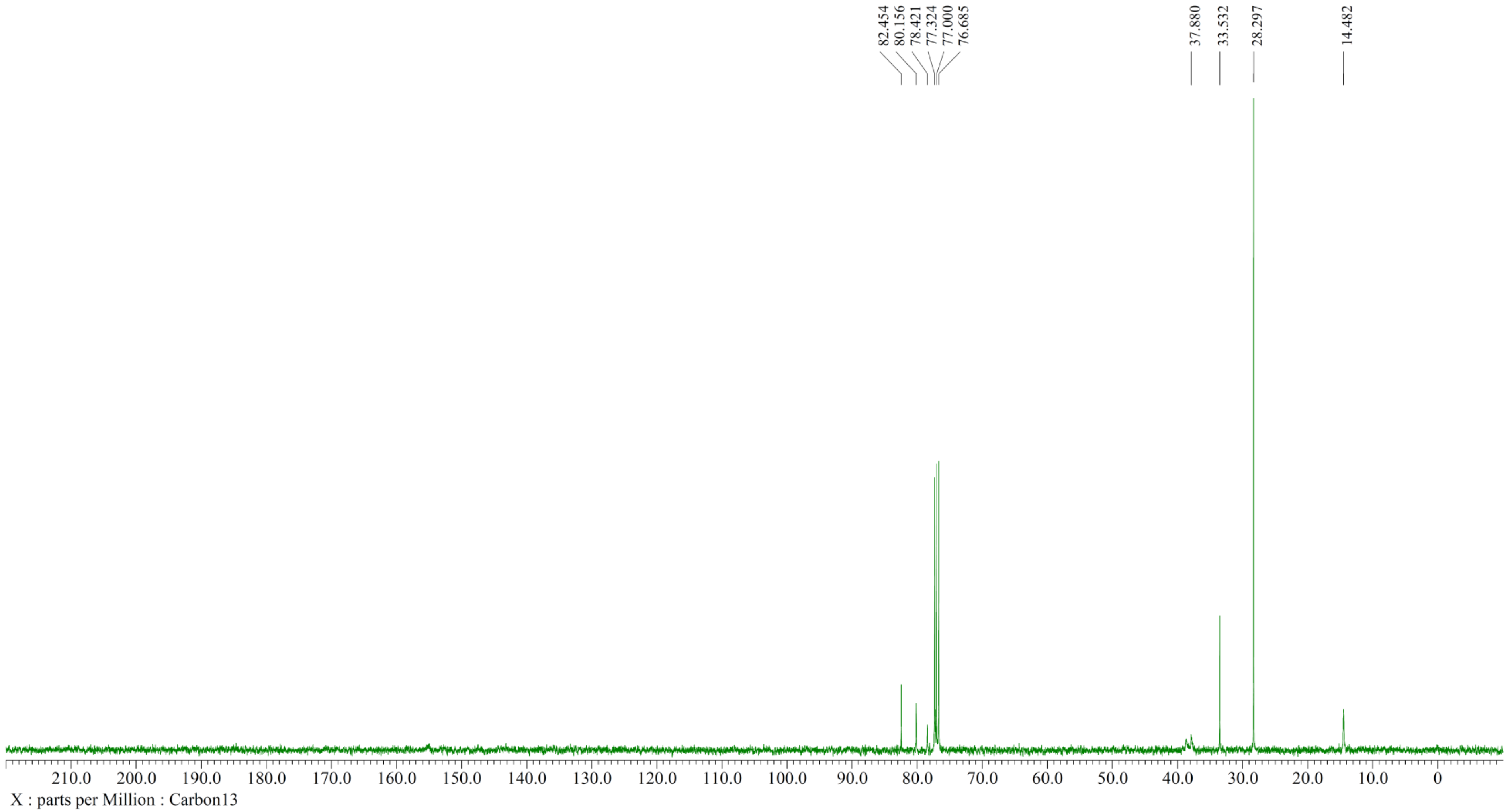

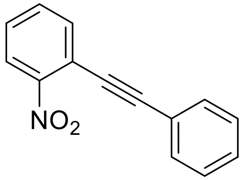

14

^1H NMR (400 MHz, $CDCl_3$)

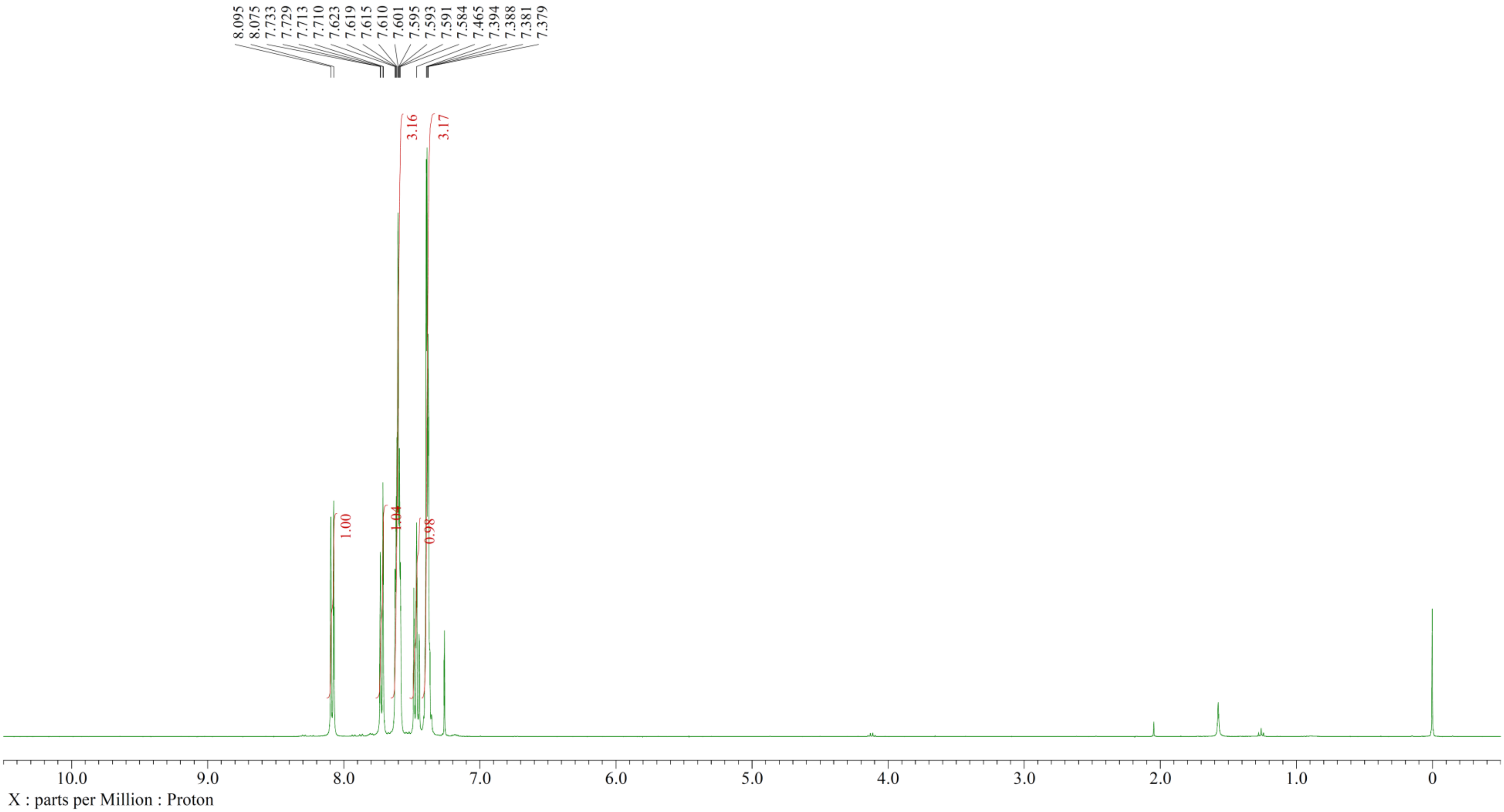

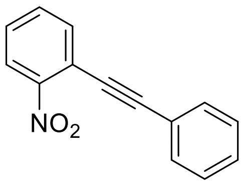

14

^{13}C NMR (100 MHz, $CDCl_3$)

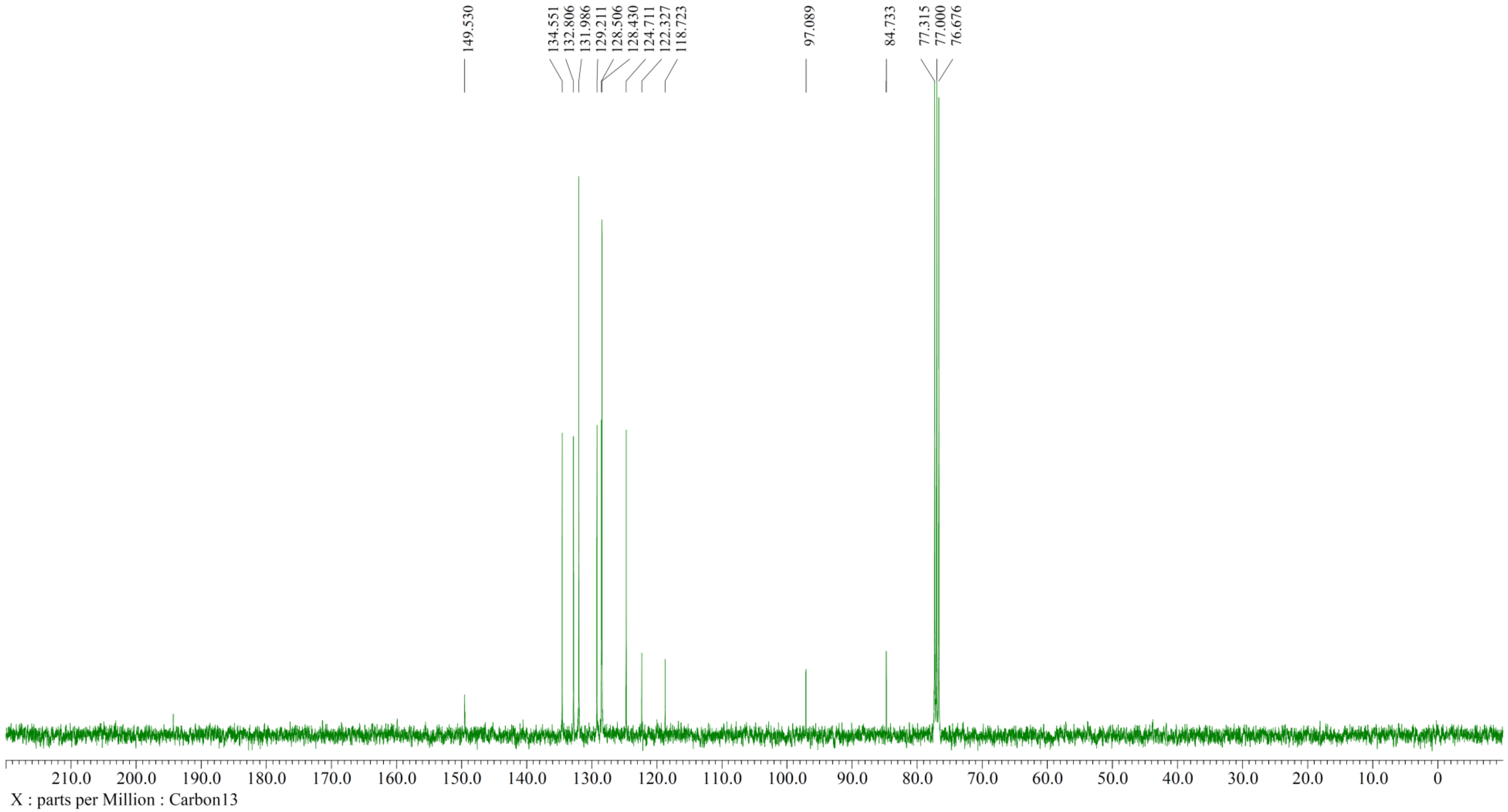

^{1}H NMR (400 MHz, $CDCl_3$)

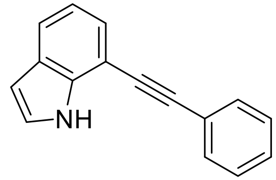

15

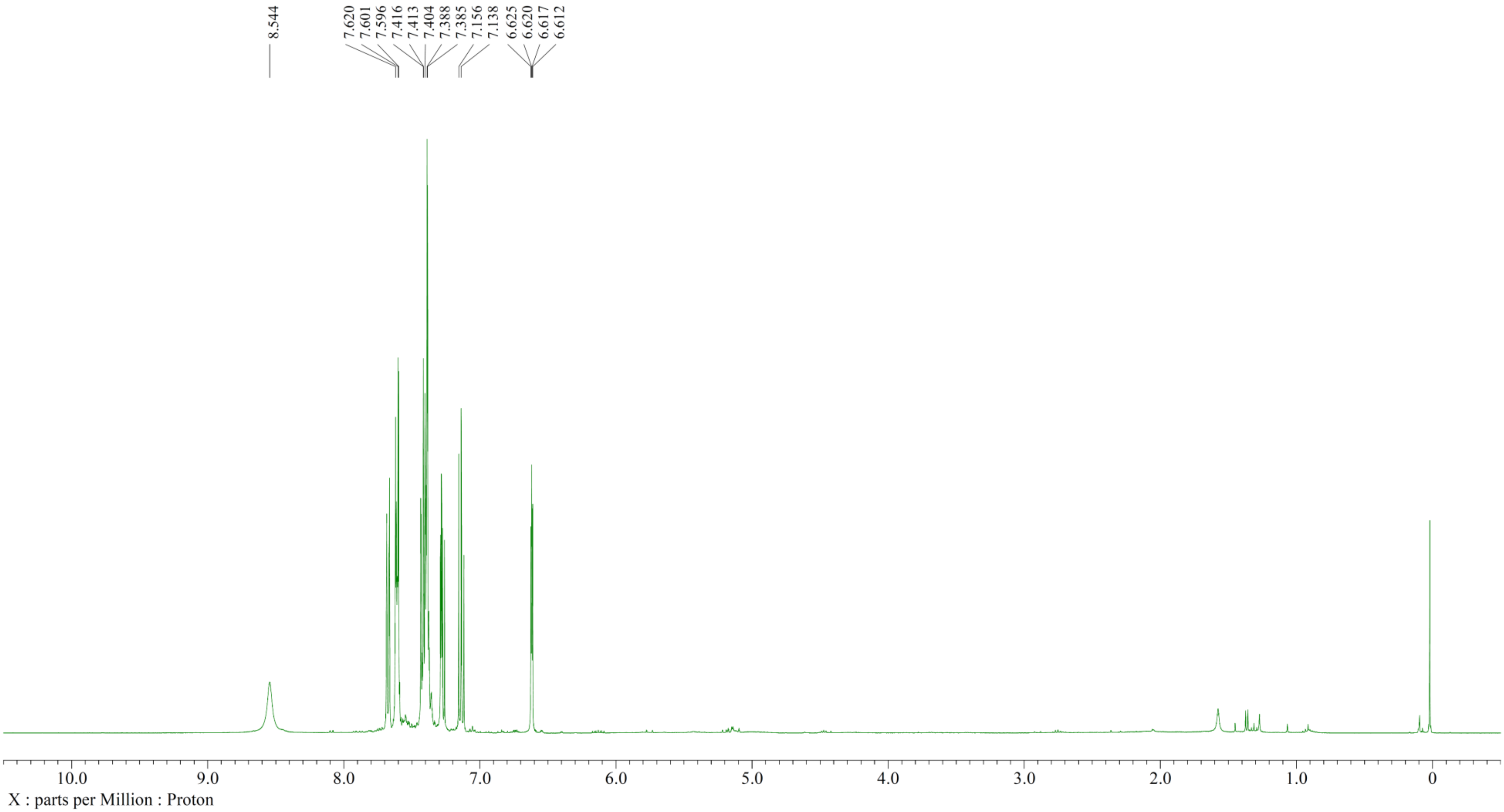

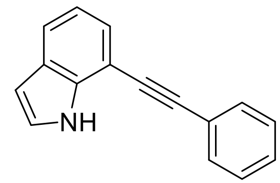

15

^{13}C NMR (100 MHz, $CDCl_3$)

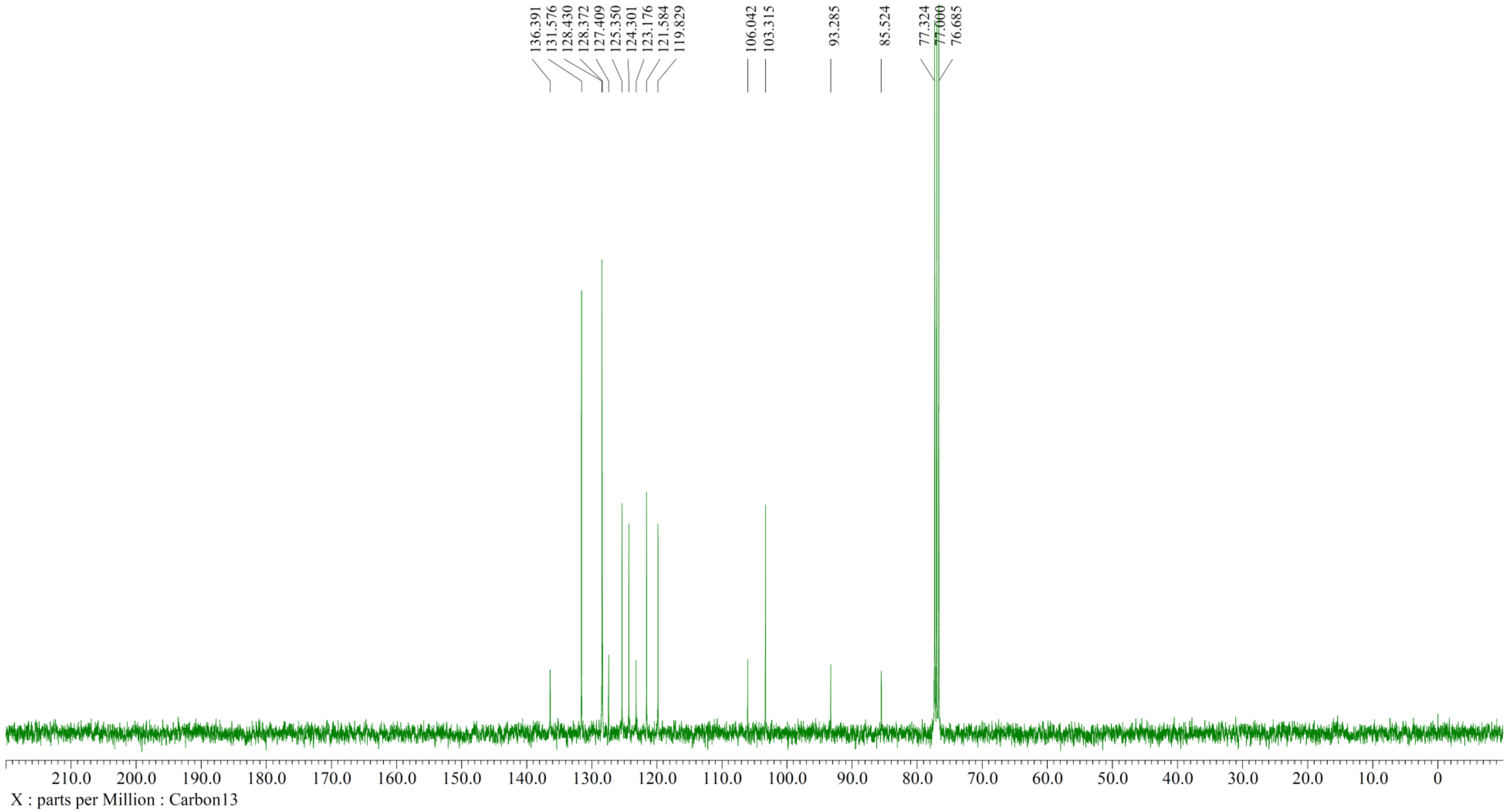

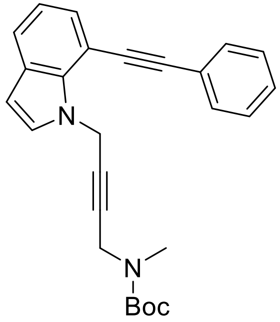

16

^{1}H NMR (400 MHz, $CDCl_3$)

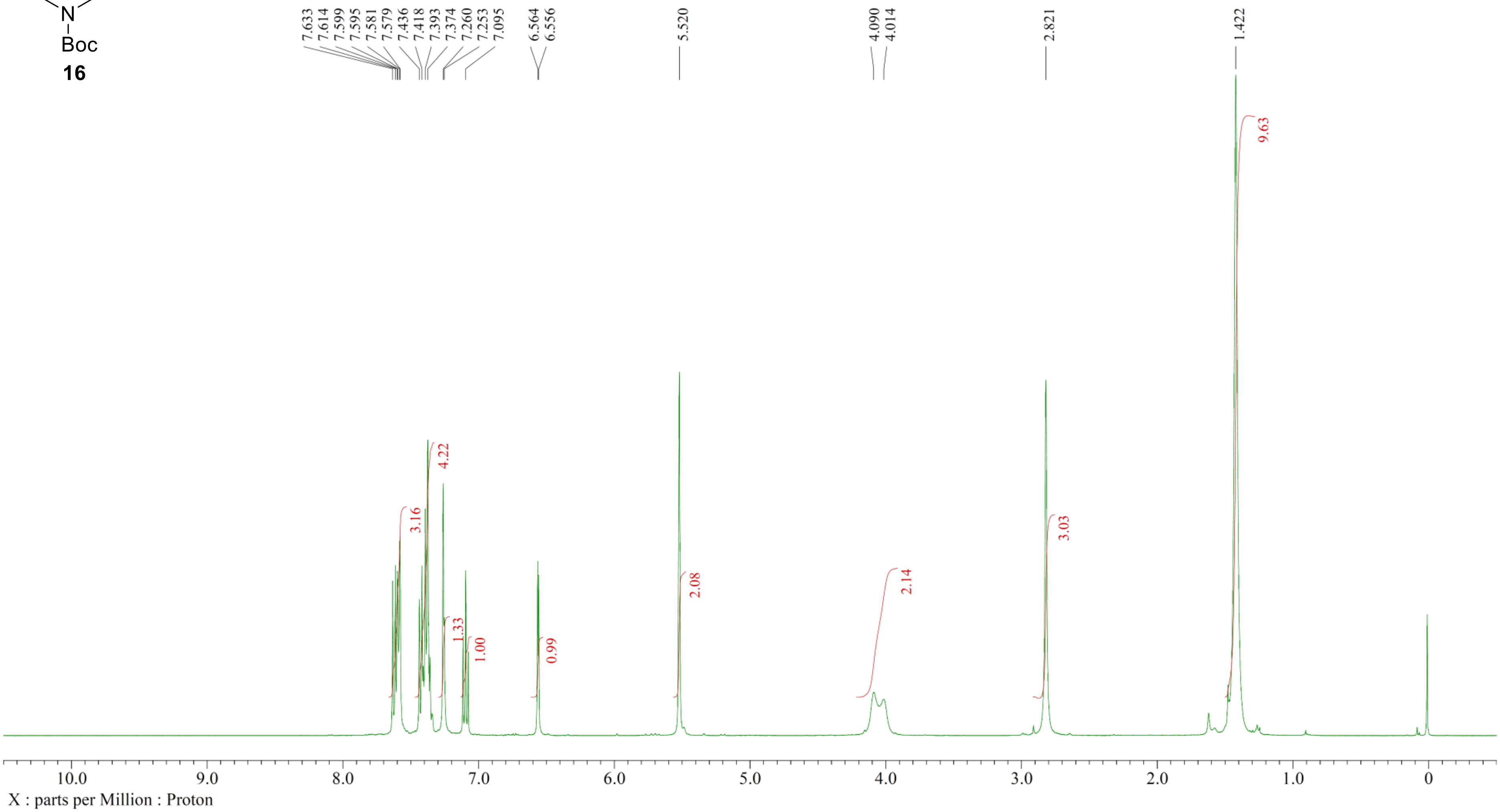

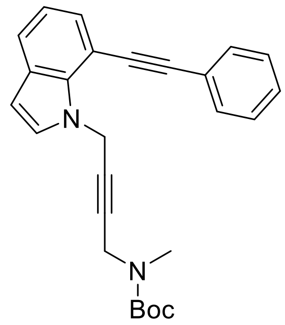

16

^{13}C NMR (100 MHz, $CDCl_3$)

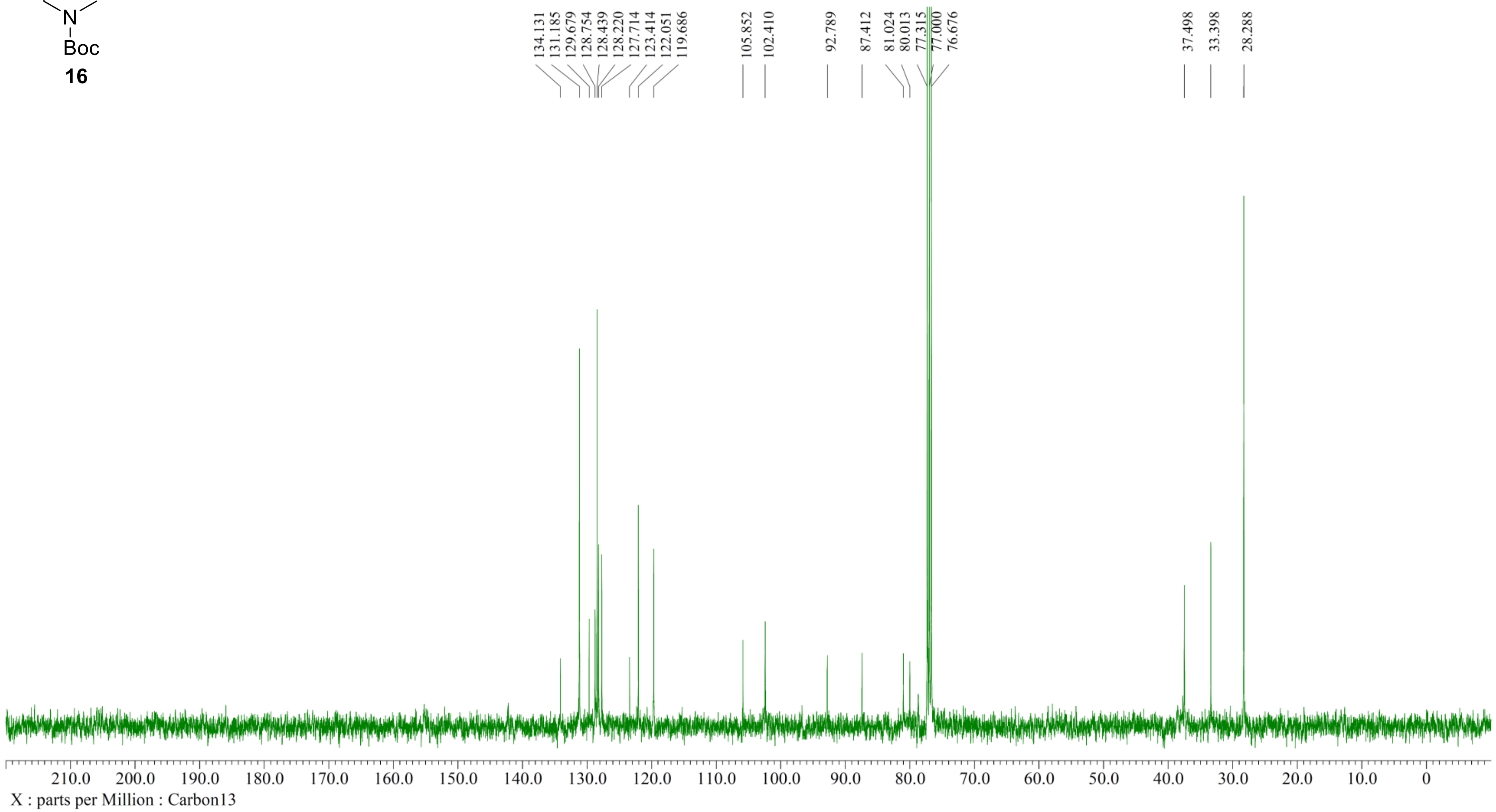

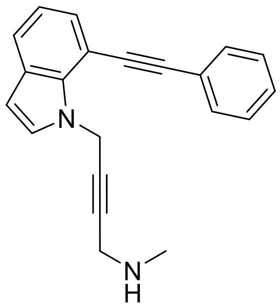

DQ-14

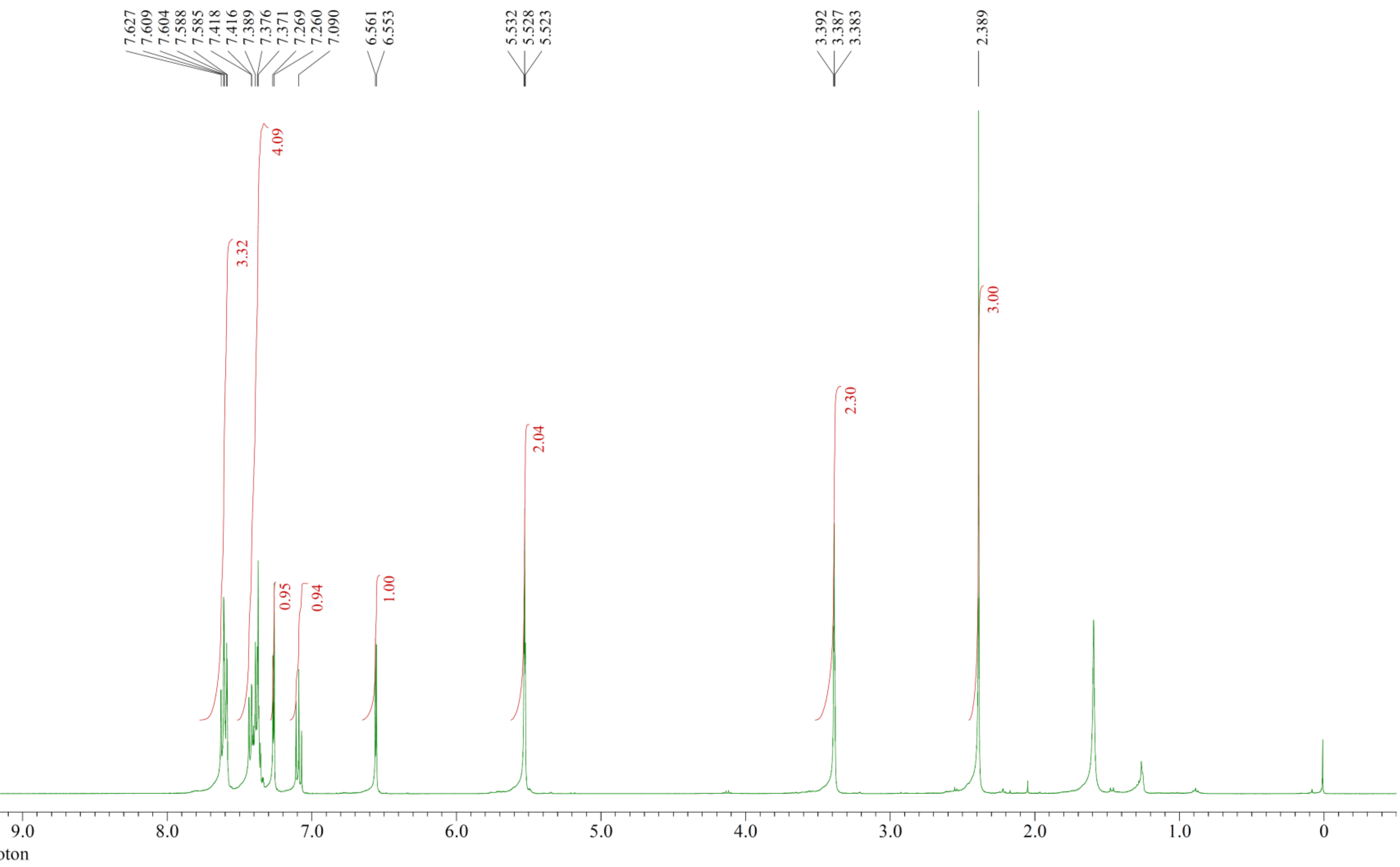

¹H NMR (400 MHz, CDCl₃)
7.627
7.609
7.604
7.588
7.585
7.418
7.416
7.389
7.376
7.371
7.269
7.260
7.090
6.561
6.553
5.532
5.528
5.523
3.392
3.387
3.383
2.389
3.32
4.09
0.95
0.94
1.00
2.04
2.30
3.00
10.0
9.0
8.0
7.0
6.0
5.0
4.0
3.0
2.0
1.0
0
X : parts per Million : Proton

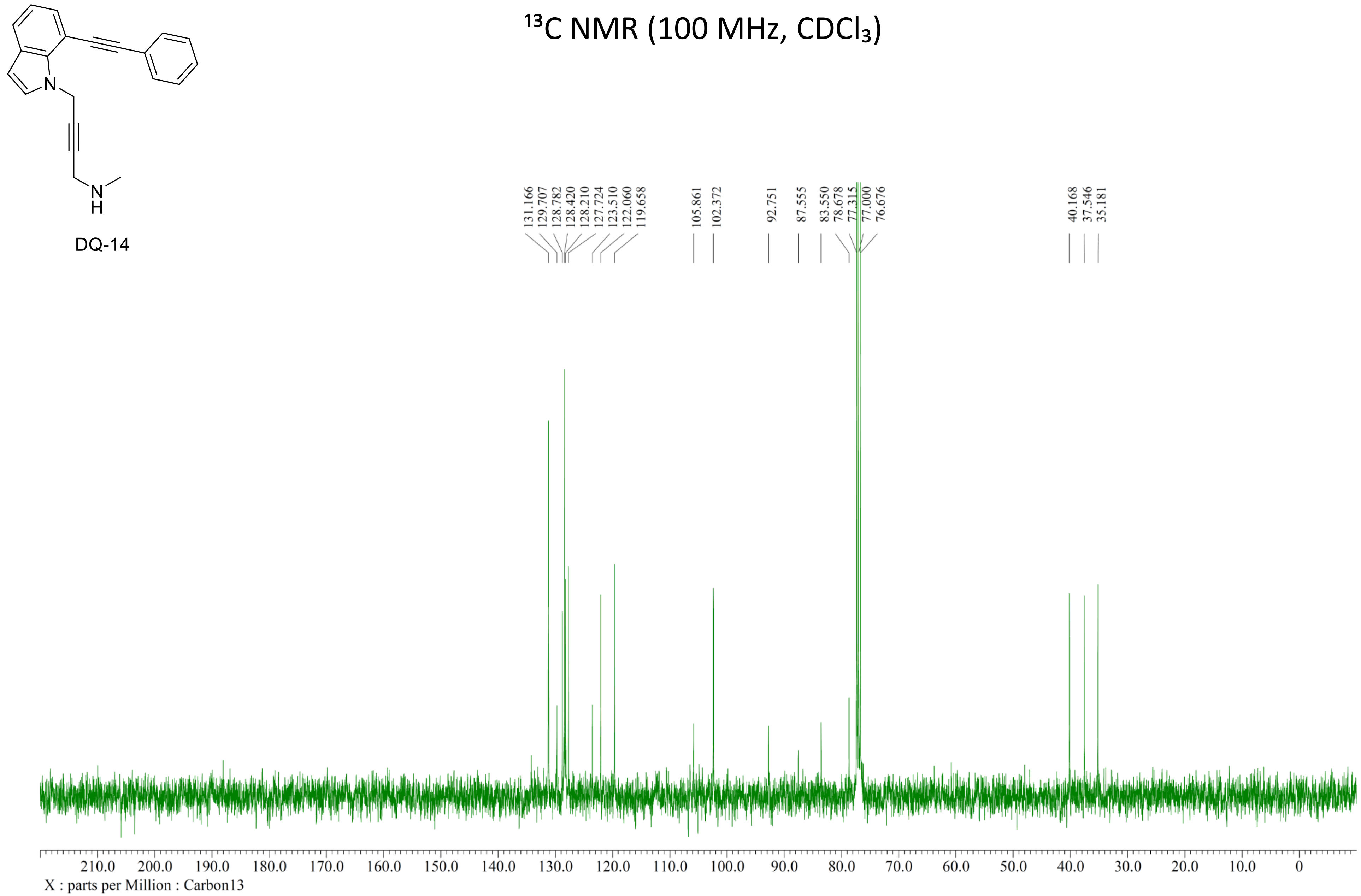
DQ-14
13C NMR (100 MHz, CDCl3)
131.166
129.707
128.782
128.420
128.210
127.724
123.510
122.060
119.658
105.861
102.372
92.751
87.555
83.550
78.678
77.315
77.000
76.676
40.168
37.546
35.181
210.0
200.0
190.0
180.0
170.0
160.0
150.0
140.0
130.0
120.0
110.0
100.0
90.0
80.0
70.0
60.0
50.0
40.0
30.0
20.0
10.0
0
X : parts per Million : Carbon13

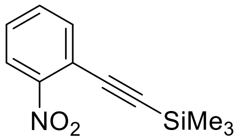

17

^1H NMR (400 MHz, $CDCl_3$)

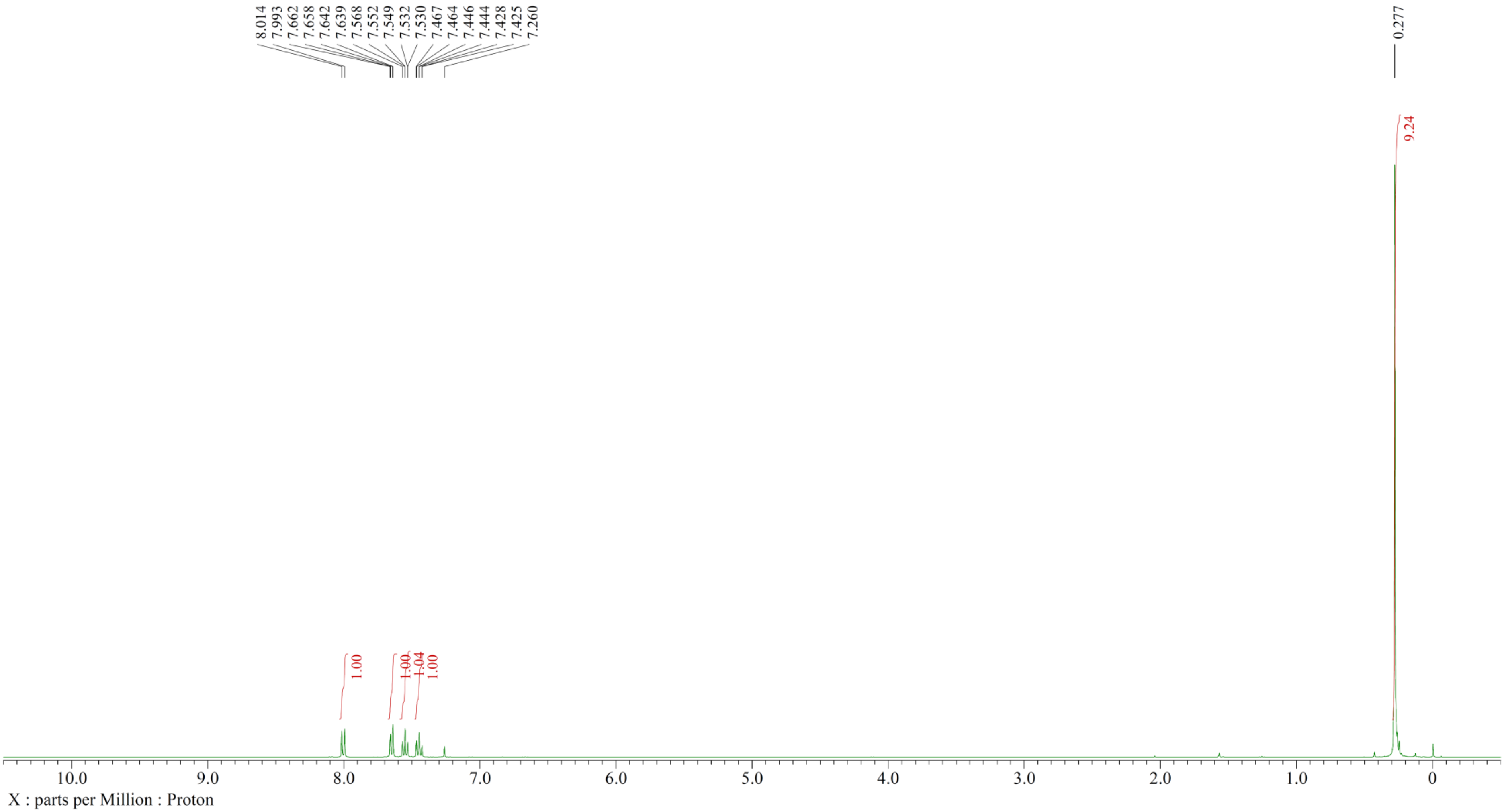

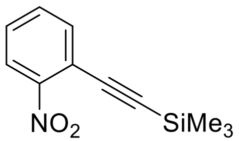

17

^{13}C NMR (100 MHz, $CDCl_3$)

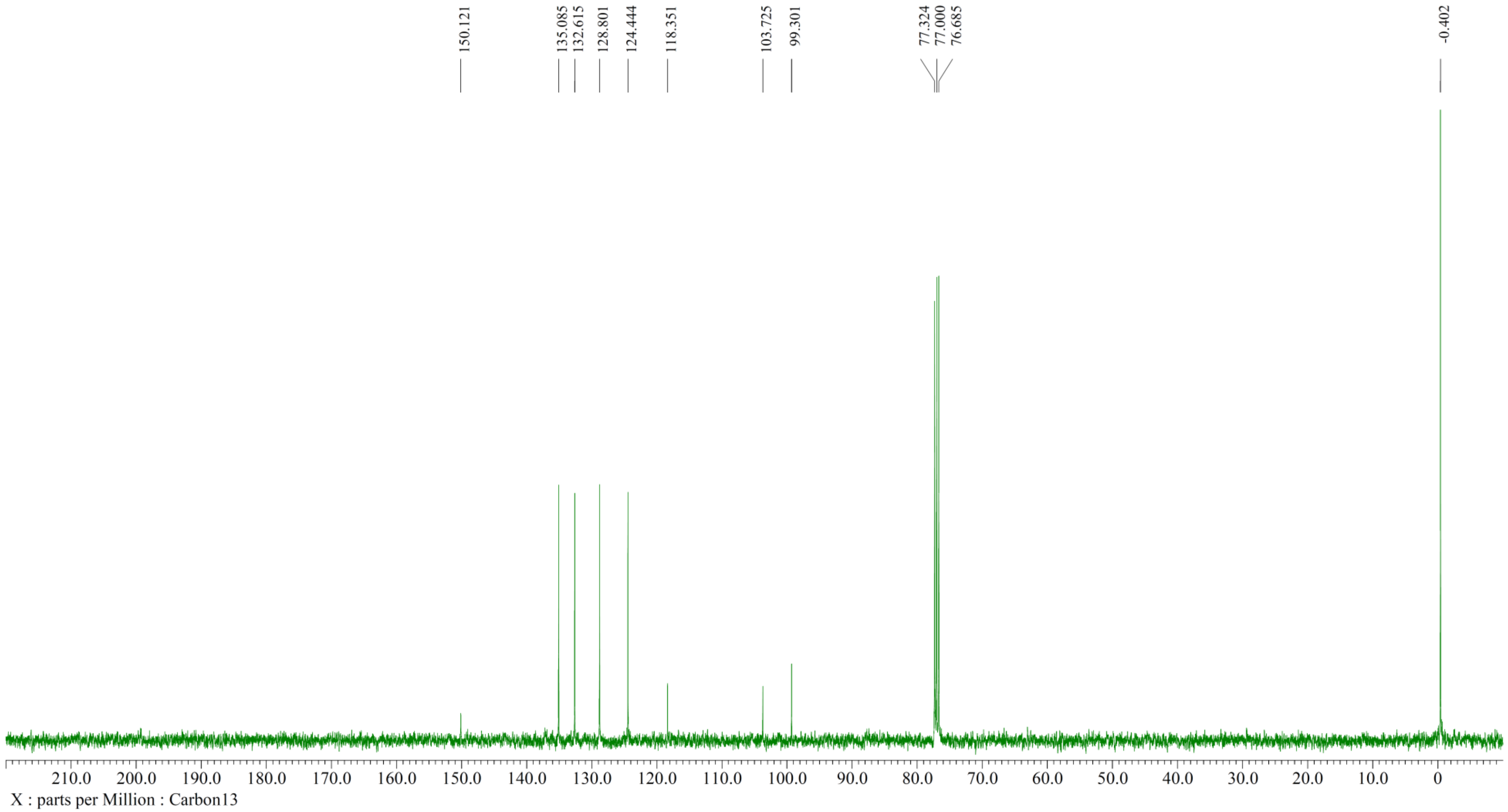

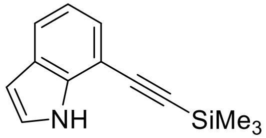

18

^1H NMR (400 MHz, $CDCl_3$)

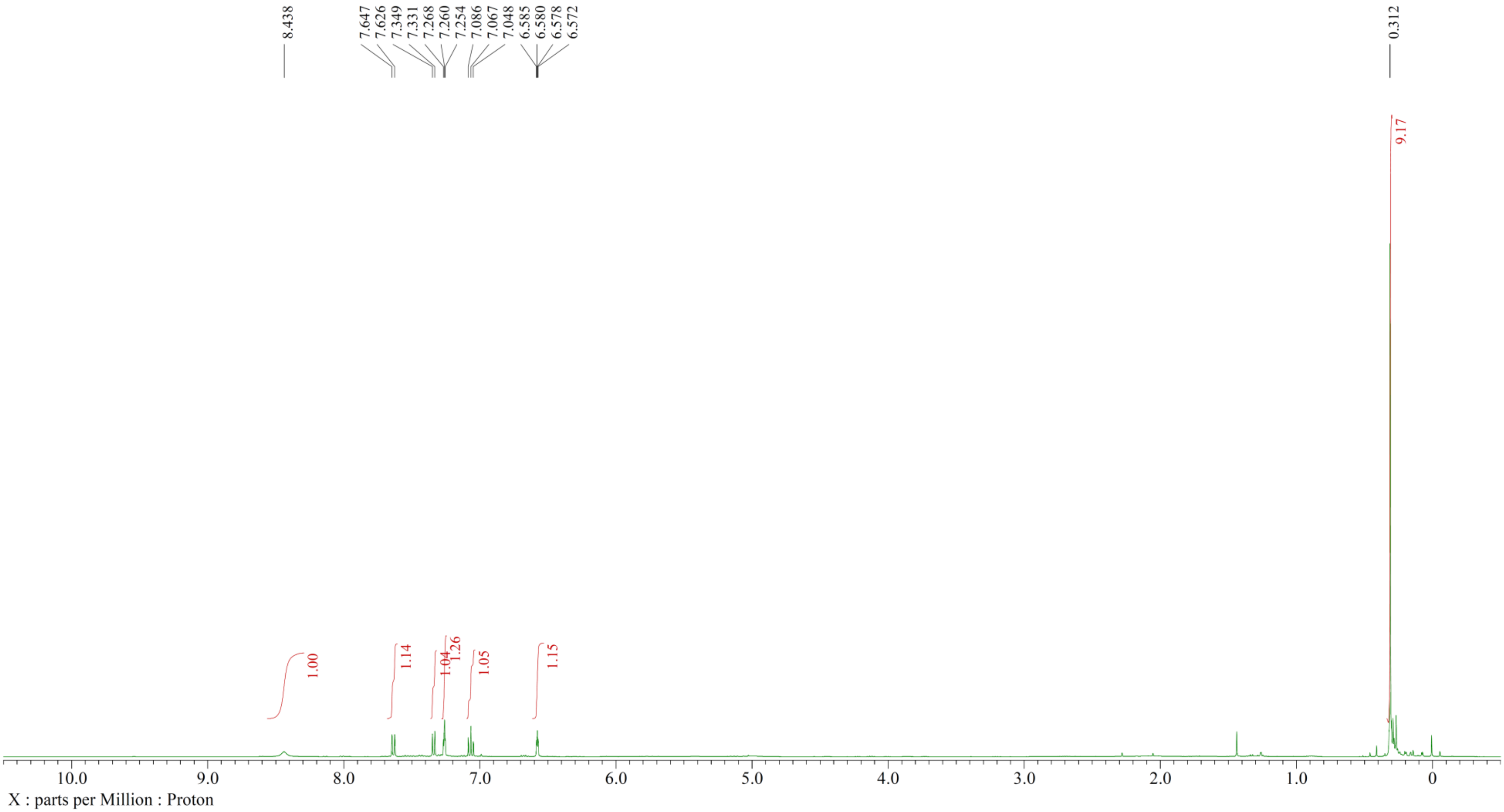

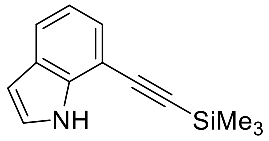

18

^{13}C NMR (100 MHz, $CDCl_3$)

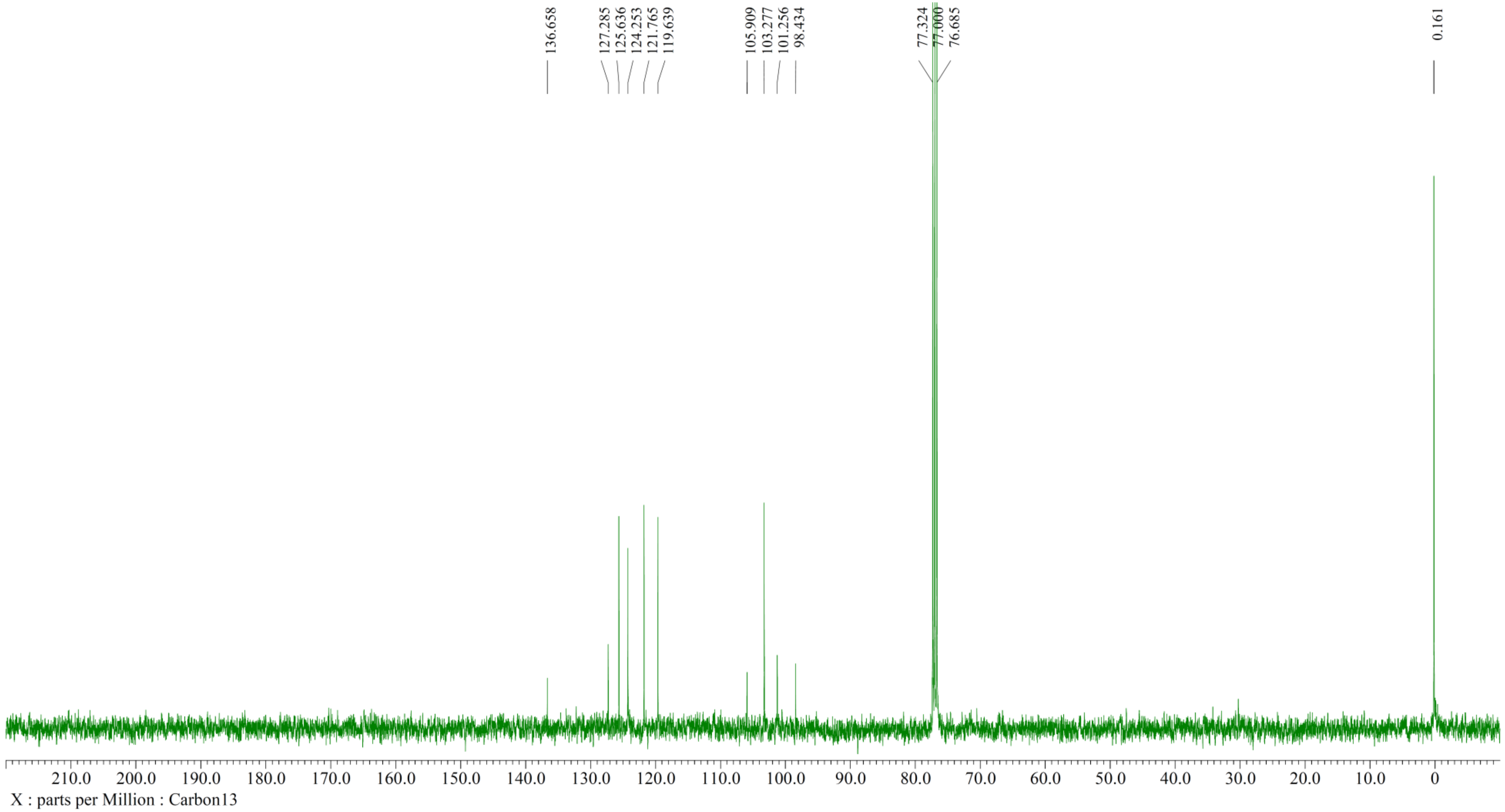

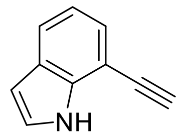

19

^{1}H NMR (400 MHz, $CDCl_3$)

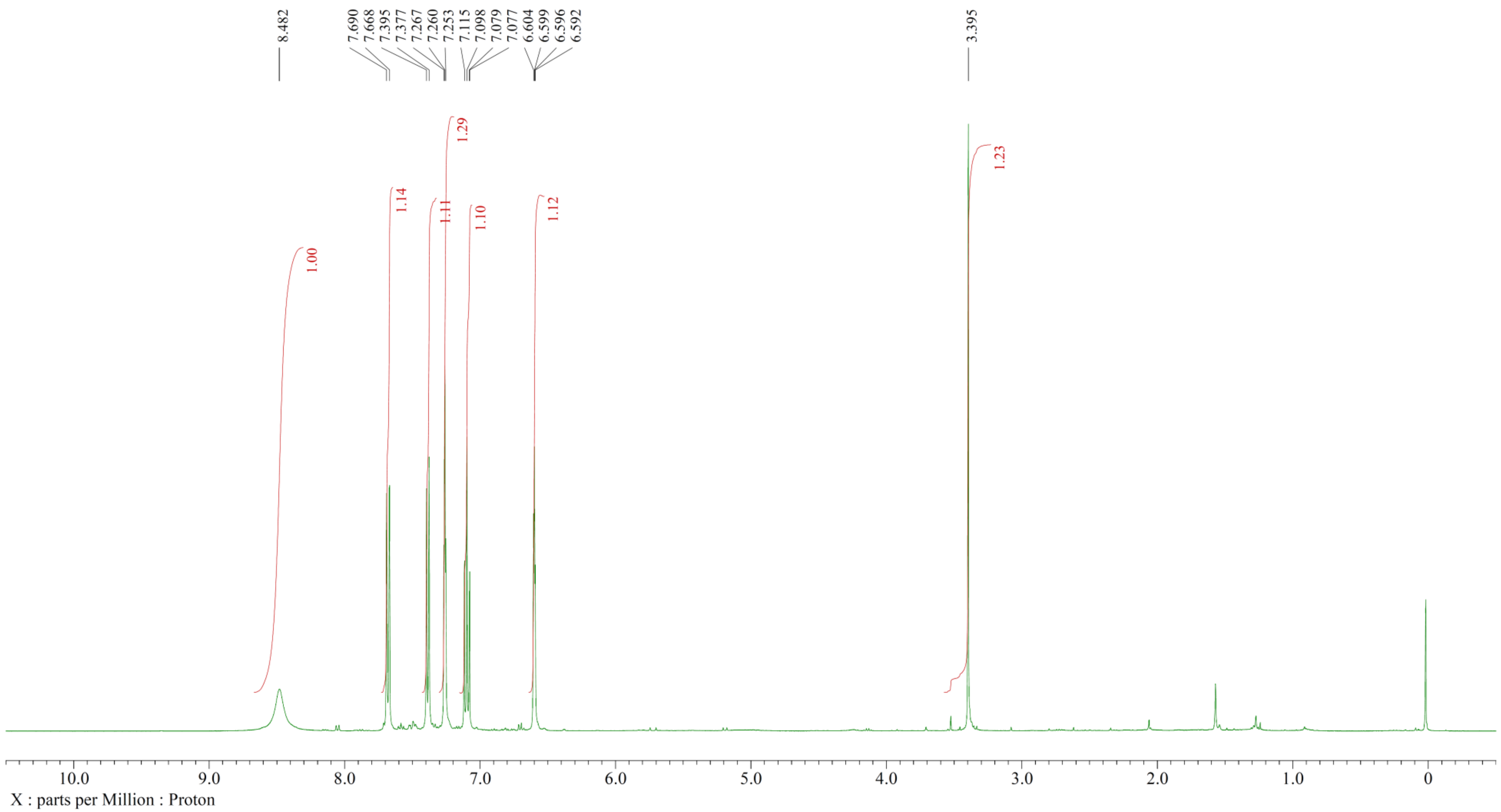

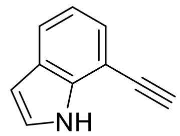

19

^{13}C NMR (100 MHz, $CDCl_3$)

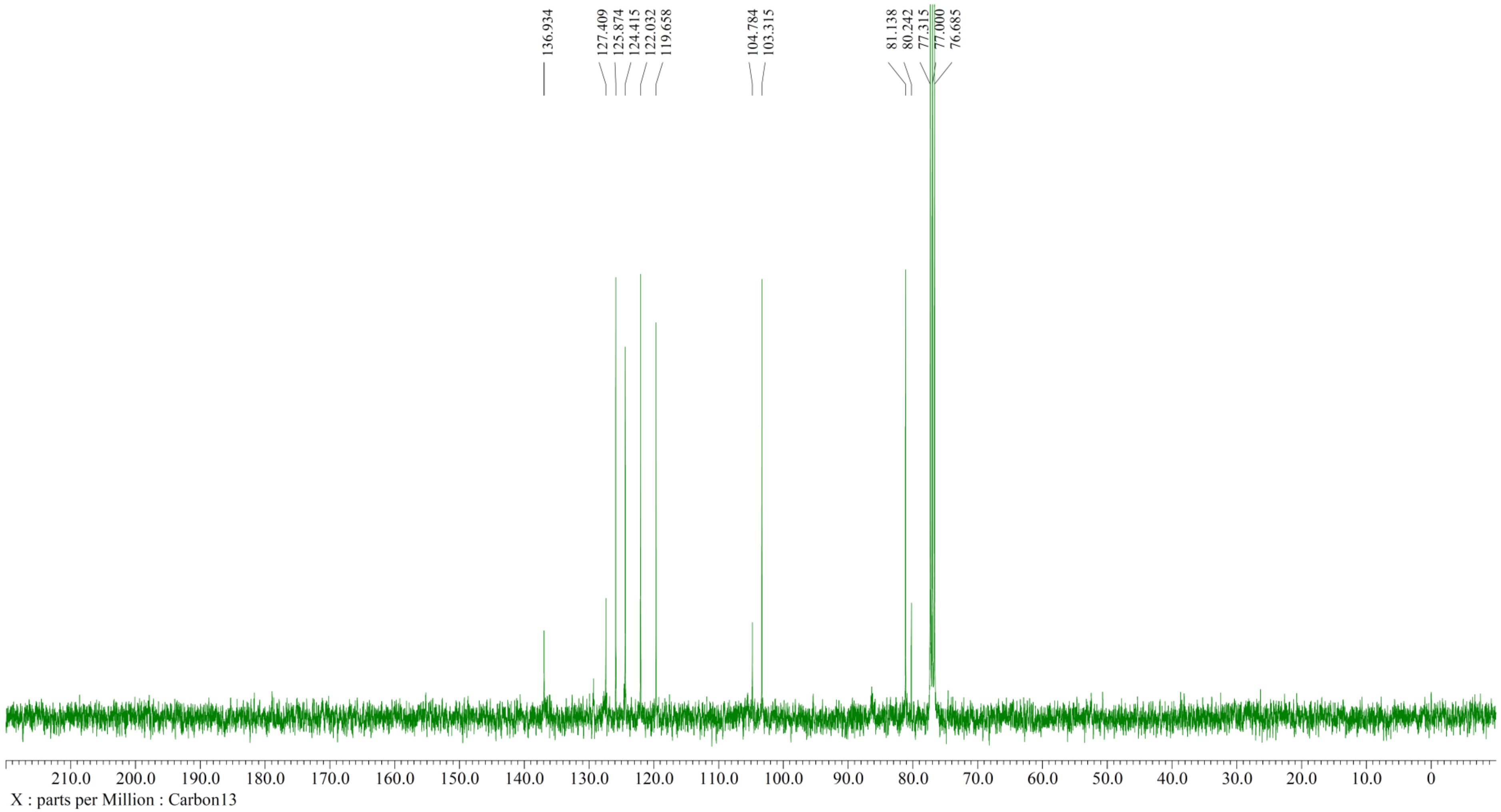

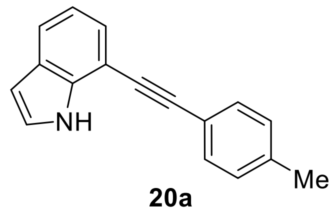

20a

^{1}H NMR (400 MHz, $CDCl_3$)

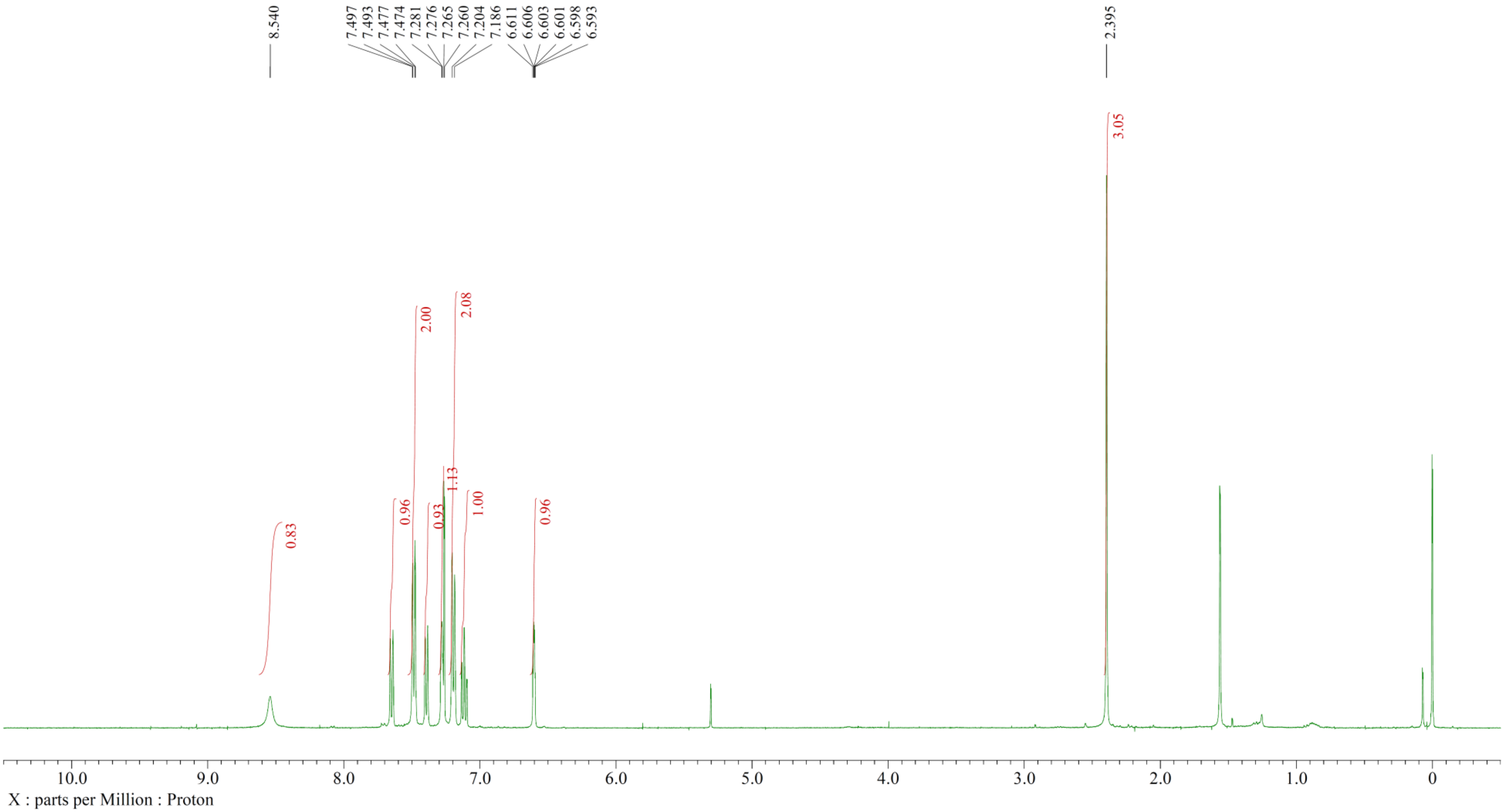

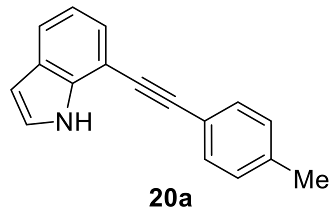

20a

^{13}C NMR (100 MHz, $CDCl_3$)

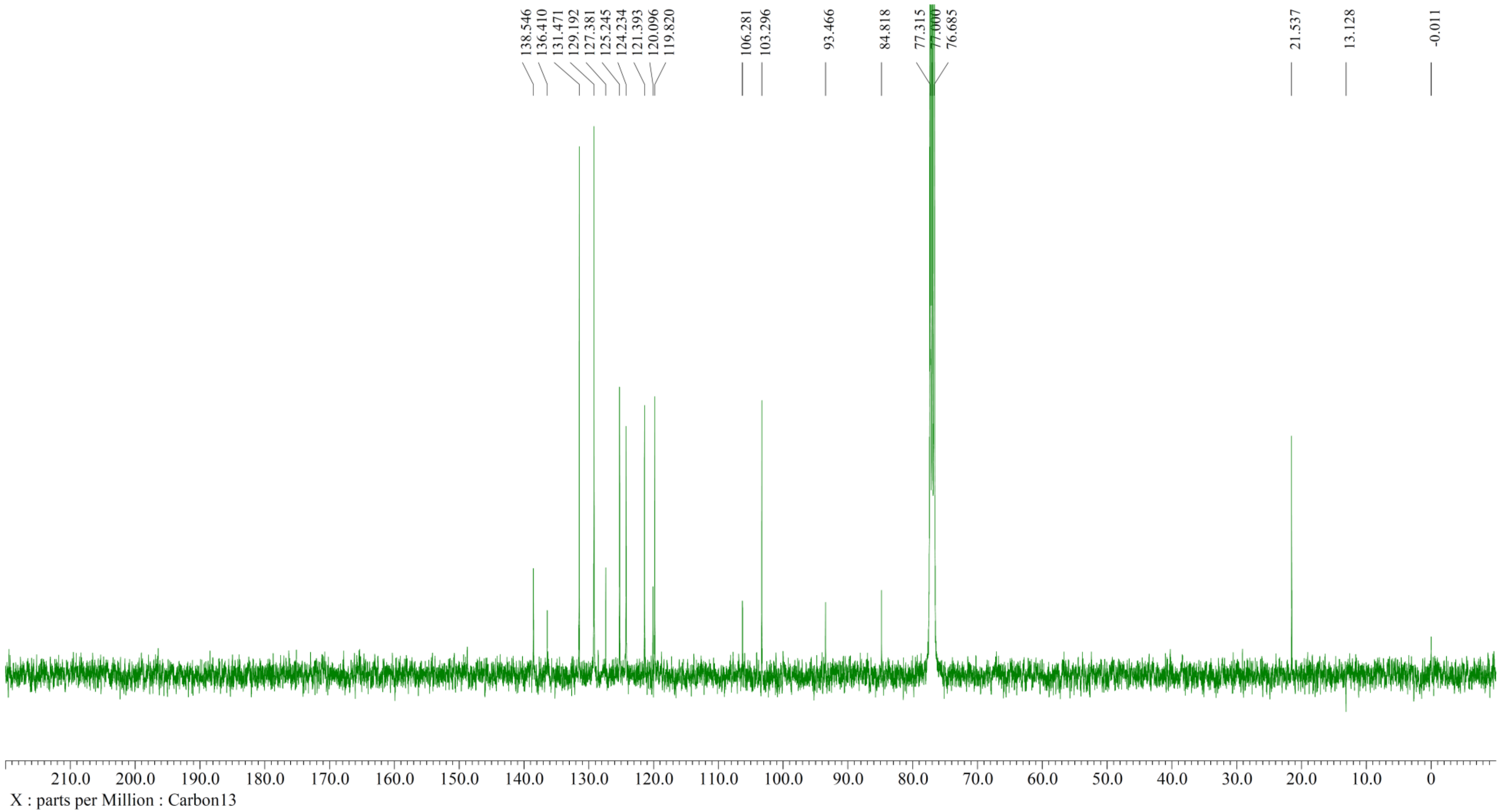

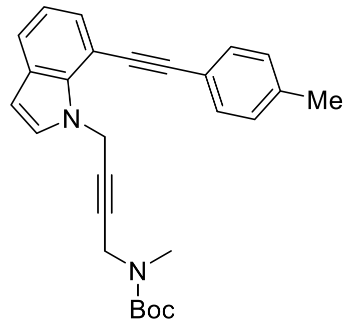

21a

^1H NMR (400 MHz, $CDCl_3$)

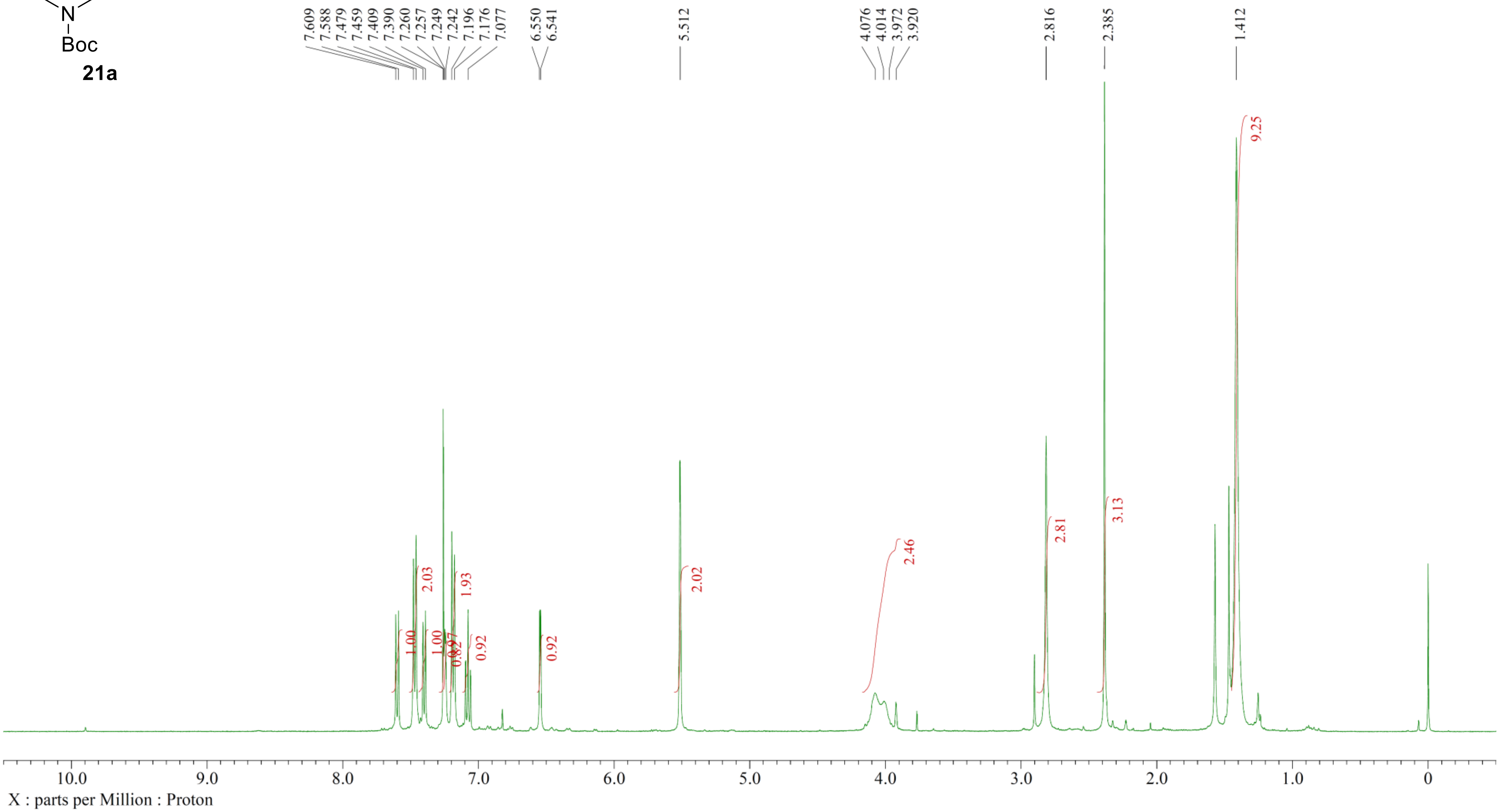

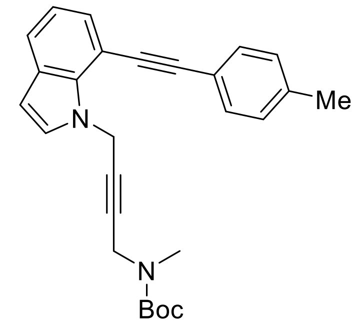

21a

^{13}C NMR (100 MHz, $CDCl_3$)

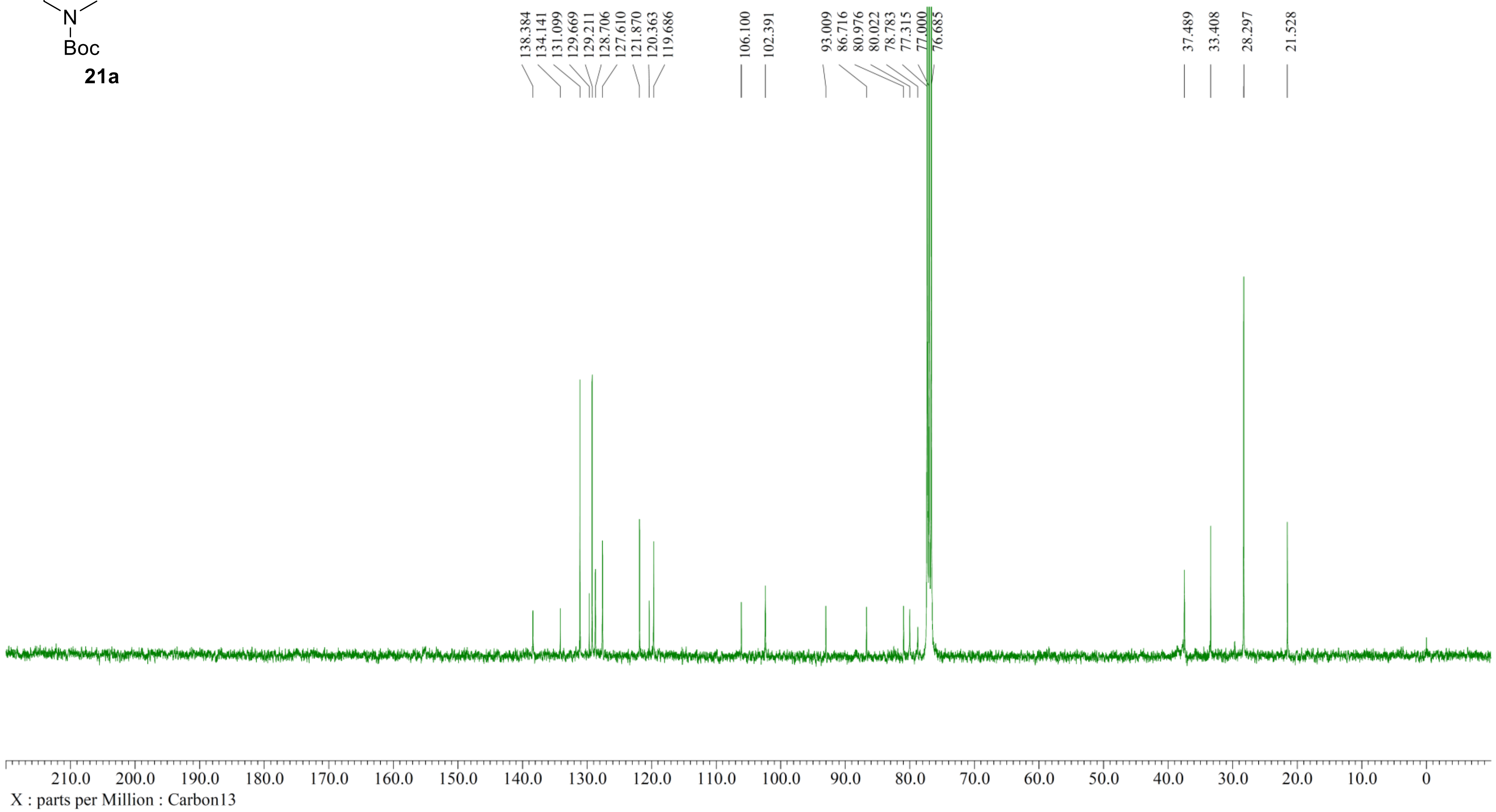

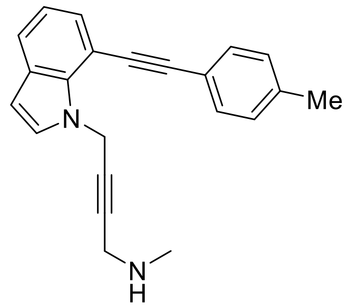

DQ-15

^{1}H NMR (400 MHz, $CDCl_3$)

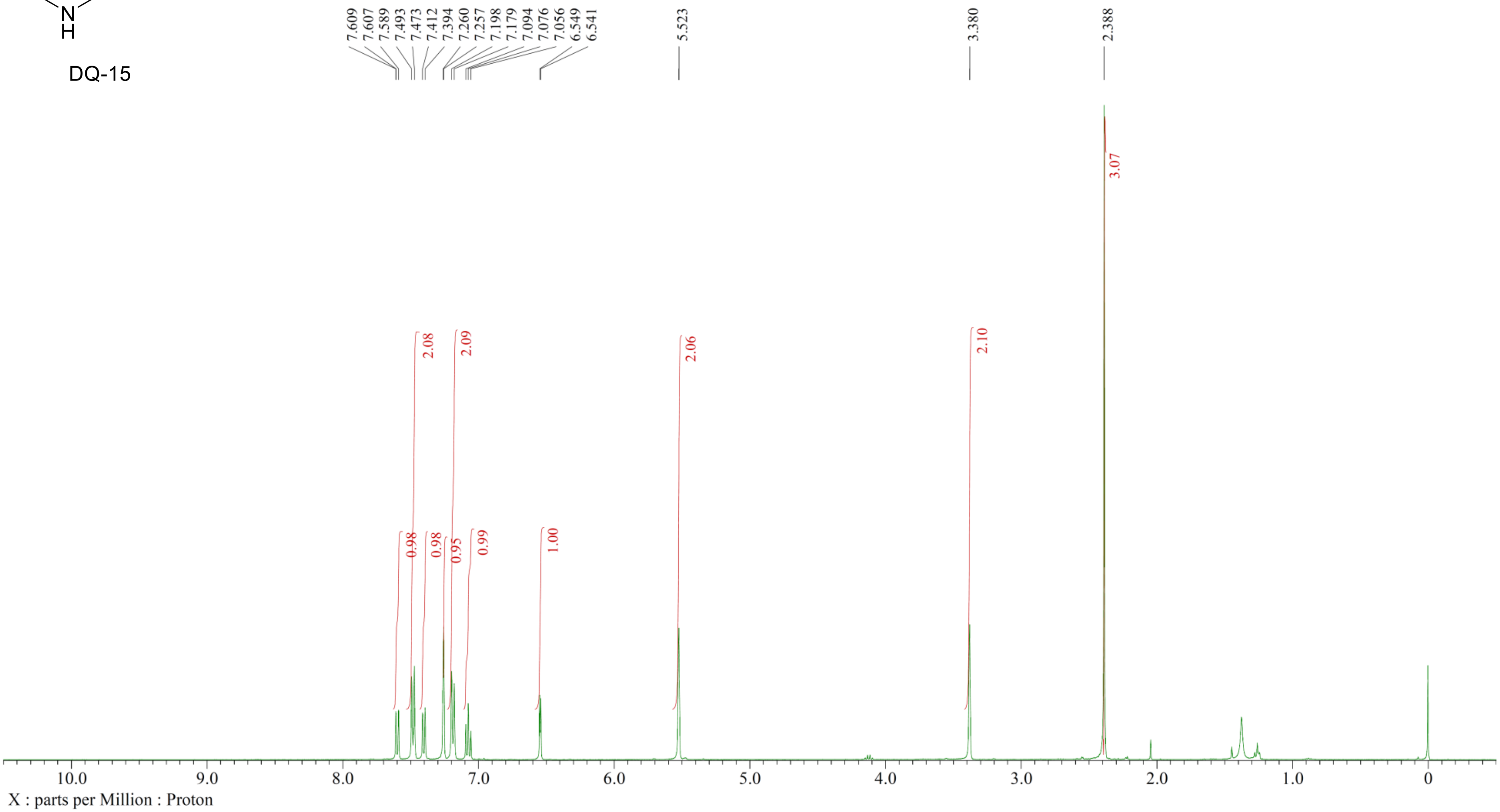

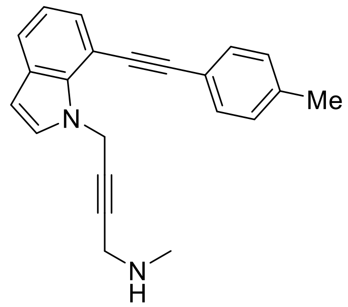

DQ-15

^{13}C NMR (100 MHz, $CDCl_3$)

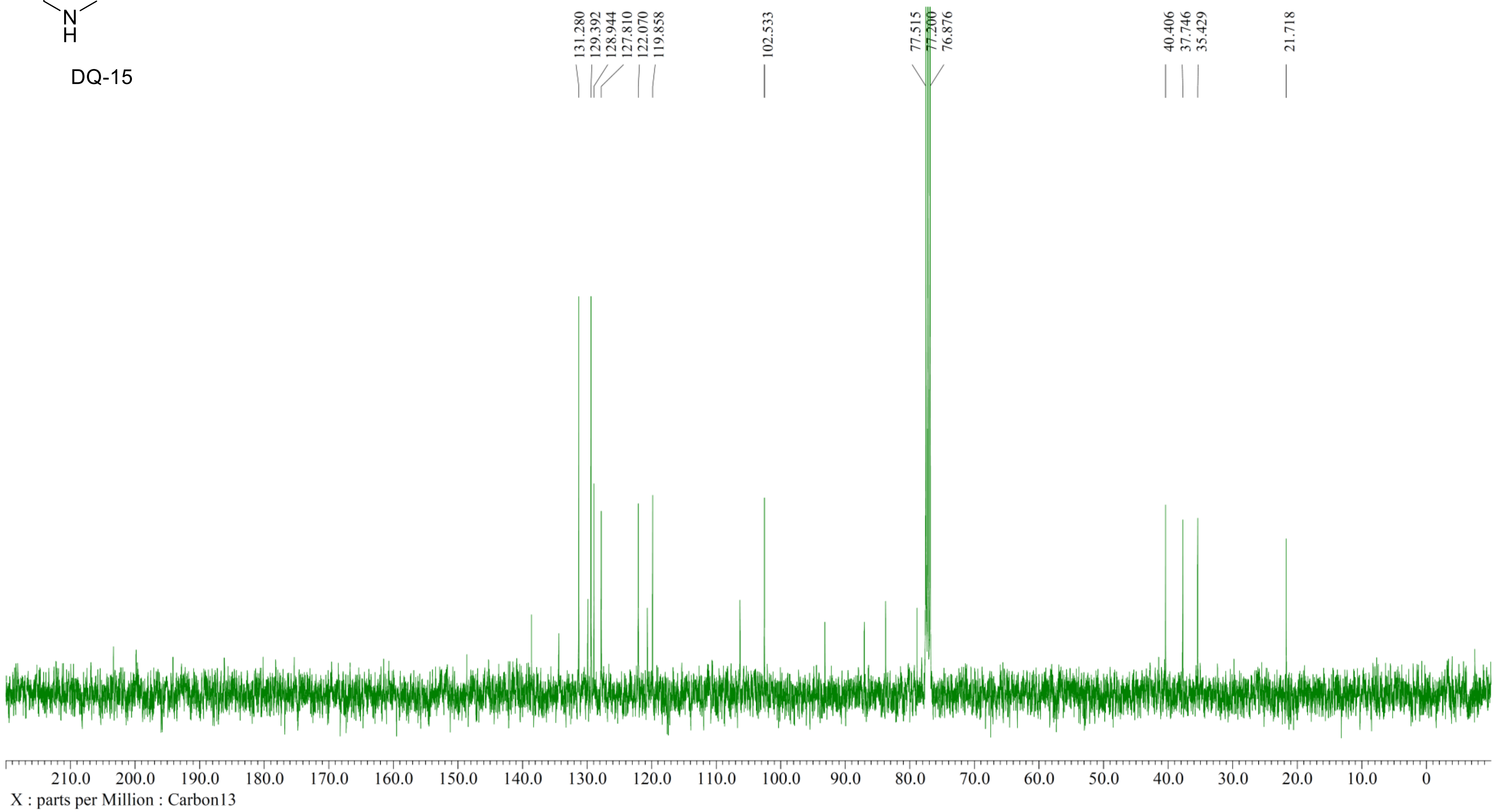

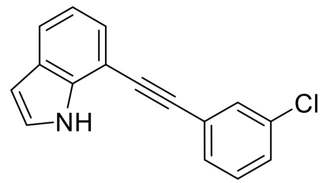

20b

^{1}H NMR (400 MHz, $CDCl_3$)

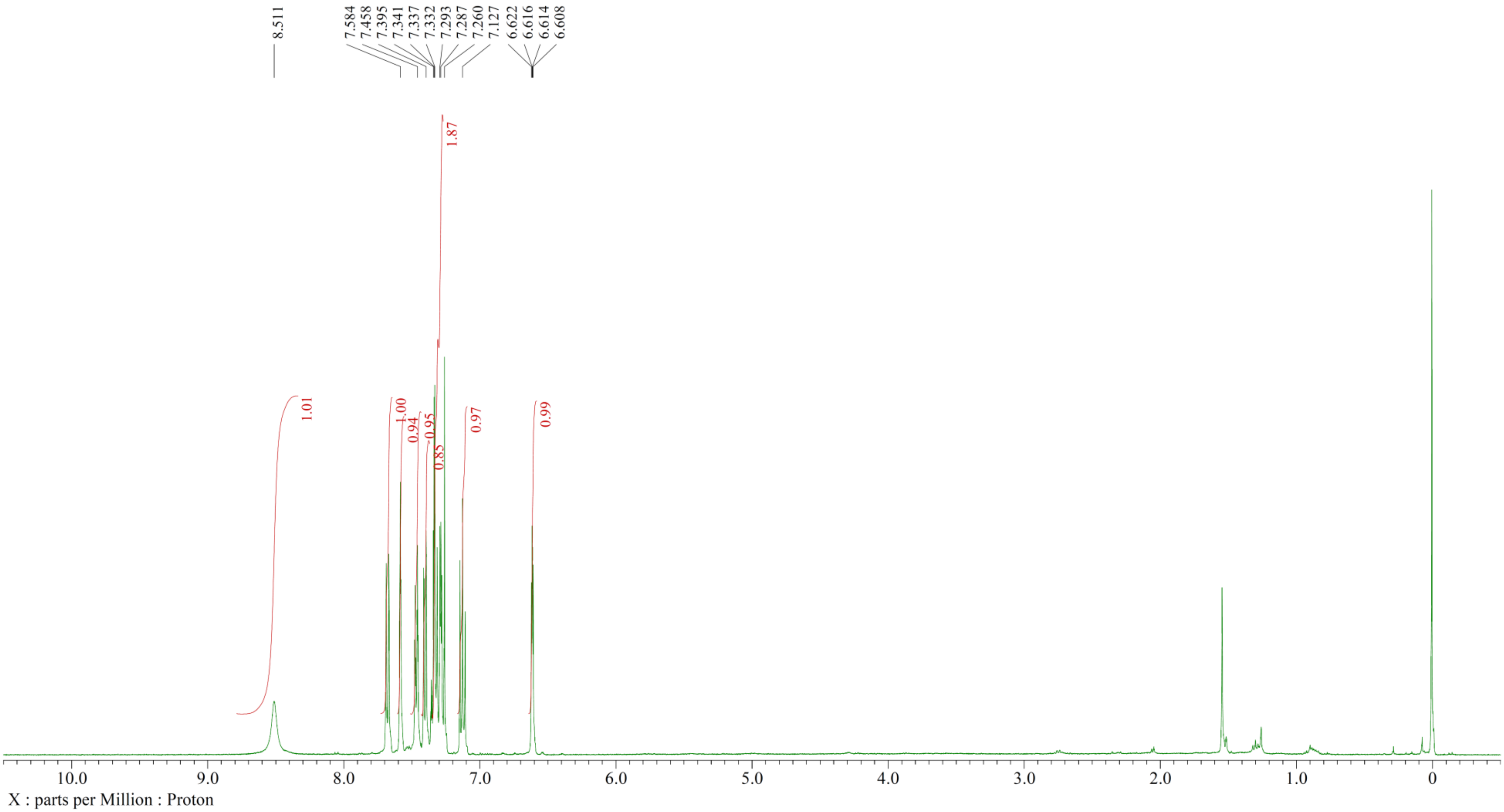

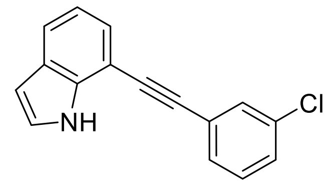

20b

^{13}C NMR (100 MHz, $CDCl_3$)

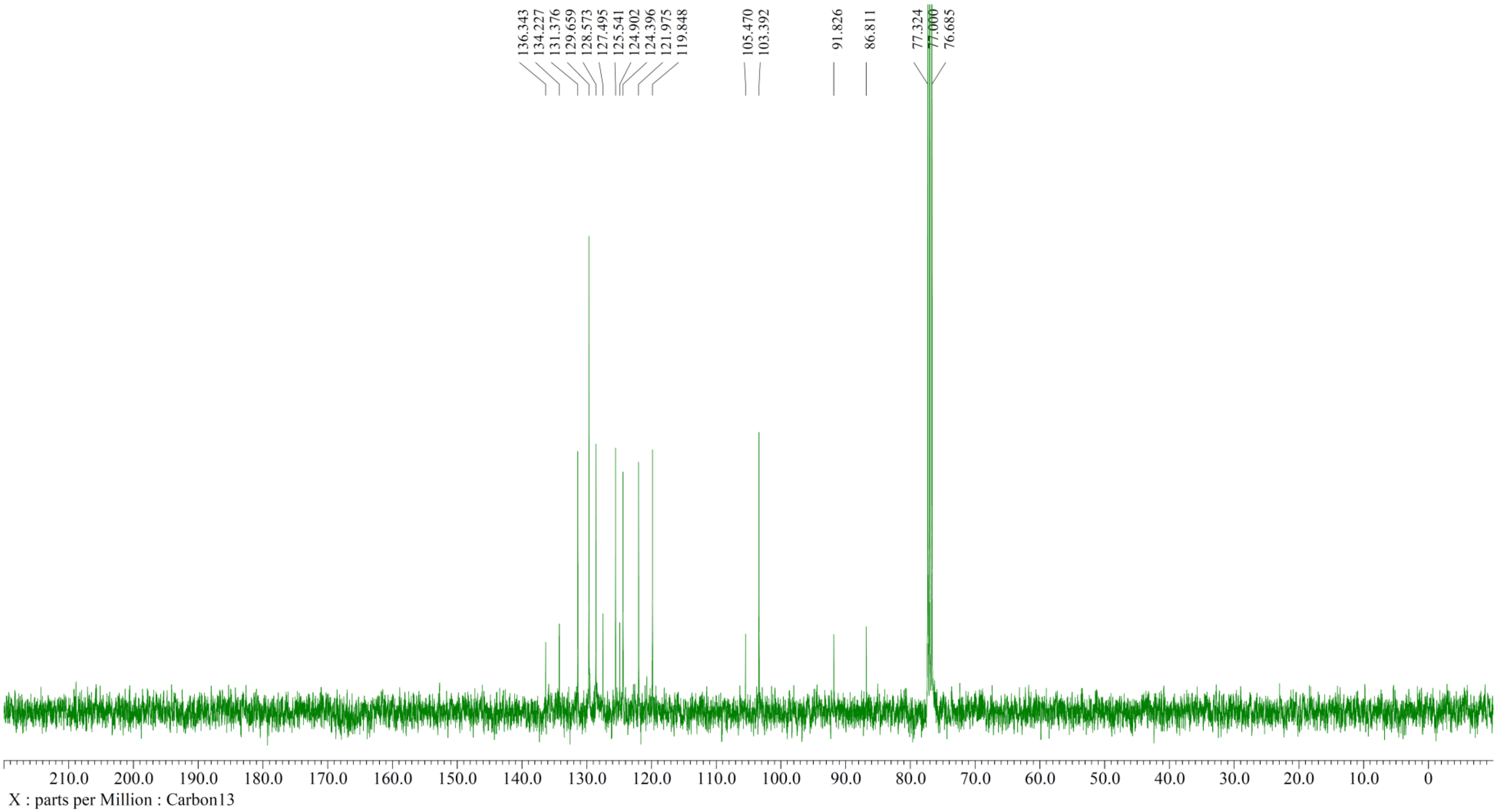

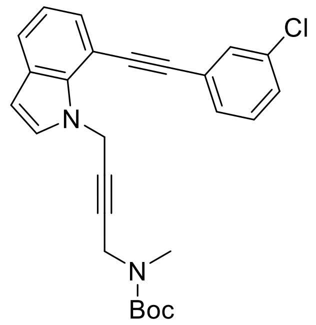

21b

^{1}H NMR (400 MHz, $CDCl_3$)

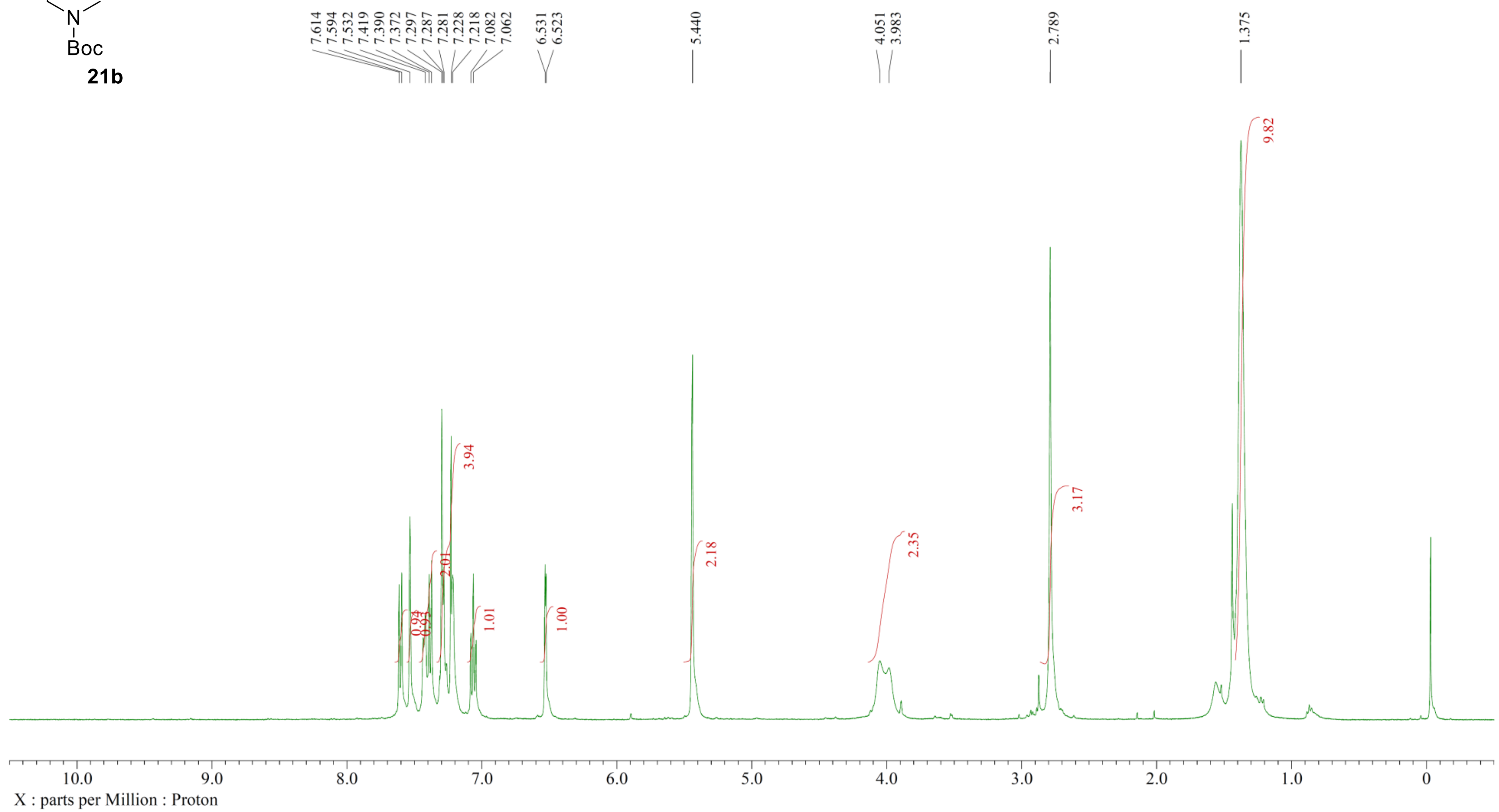

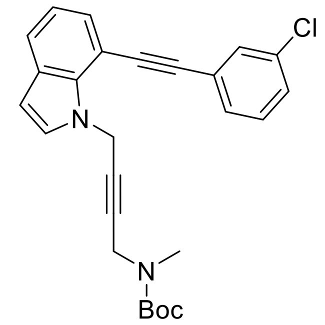

21b

^{13}C NMR (100 MHz, $CDCl_3$)

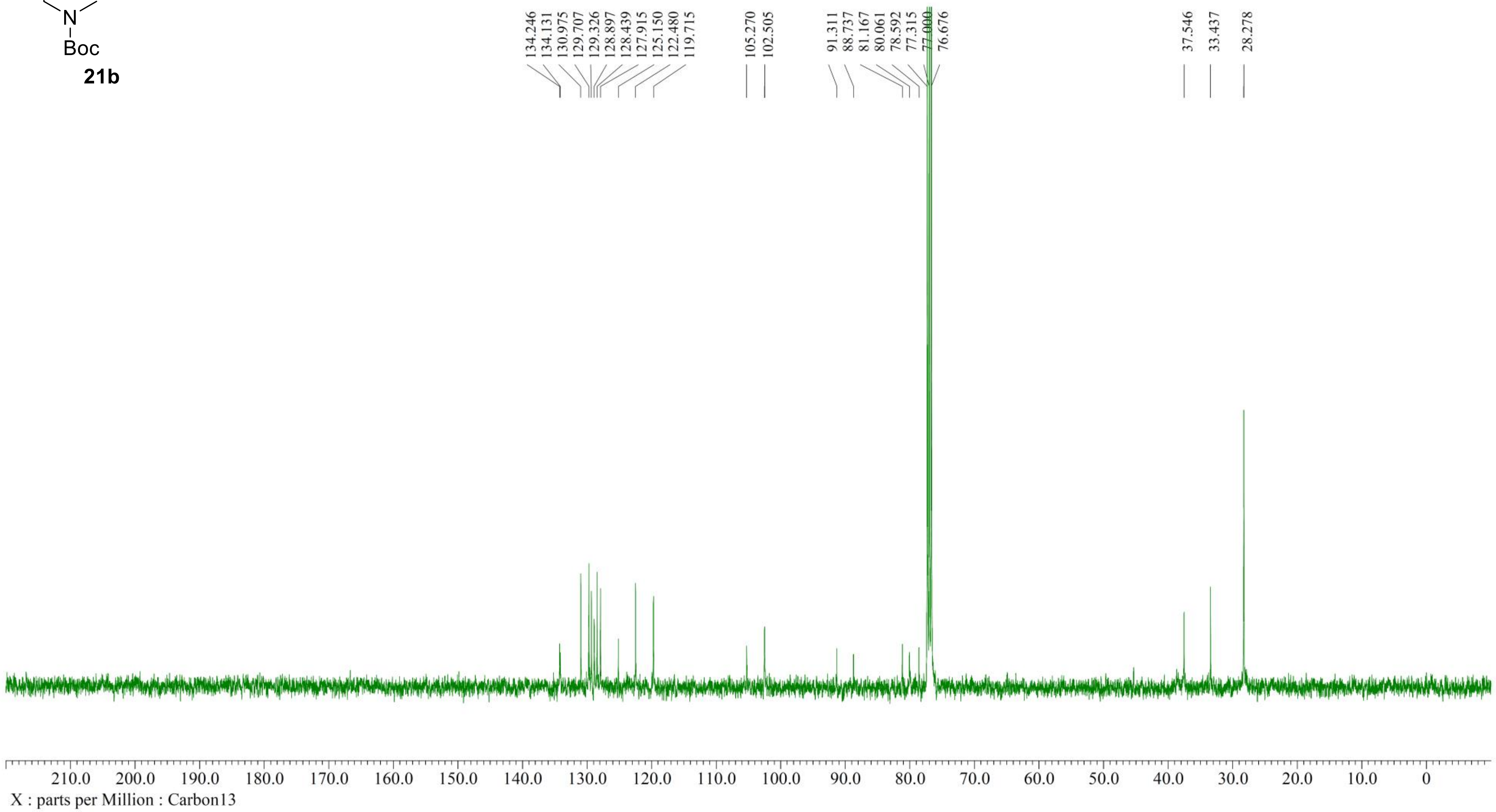

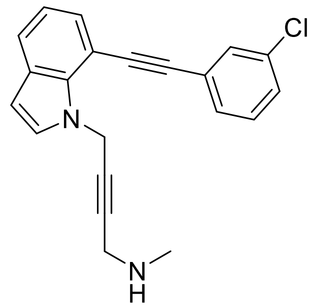

DQ-16

^{1}H NMR (400 MHz, $CDCl_3$)

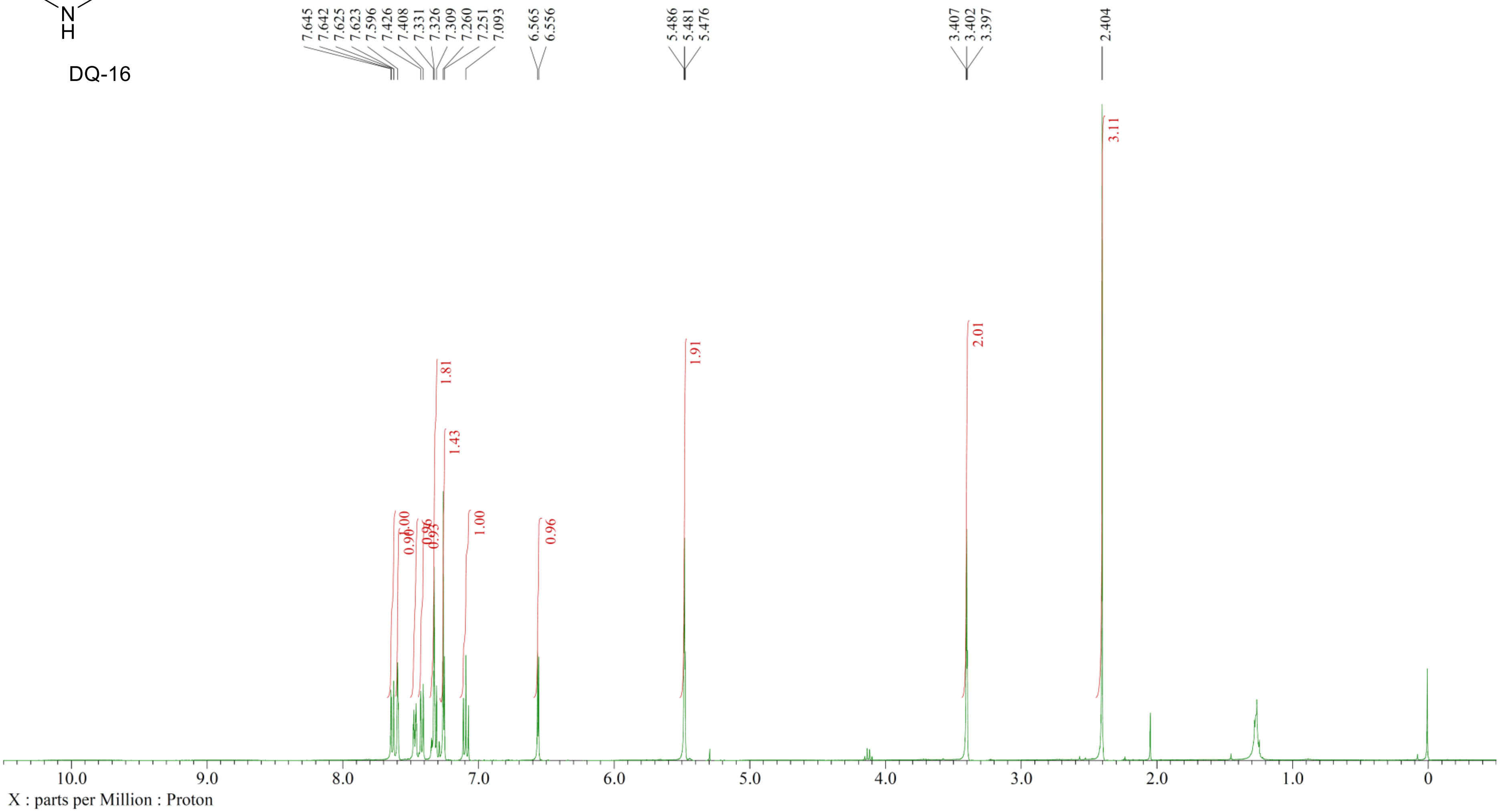

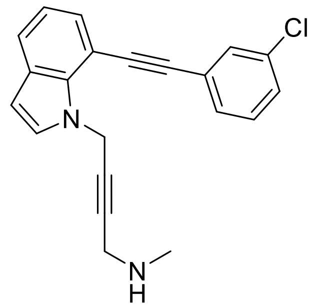

DQ-16

^{13}C NMR (100 MHz, $CDCl_3$)

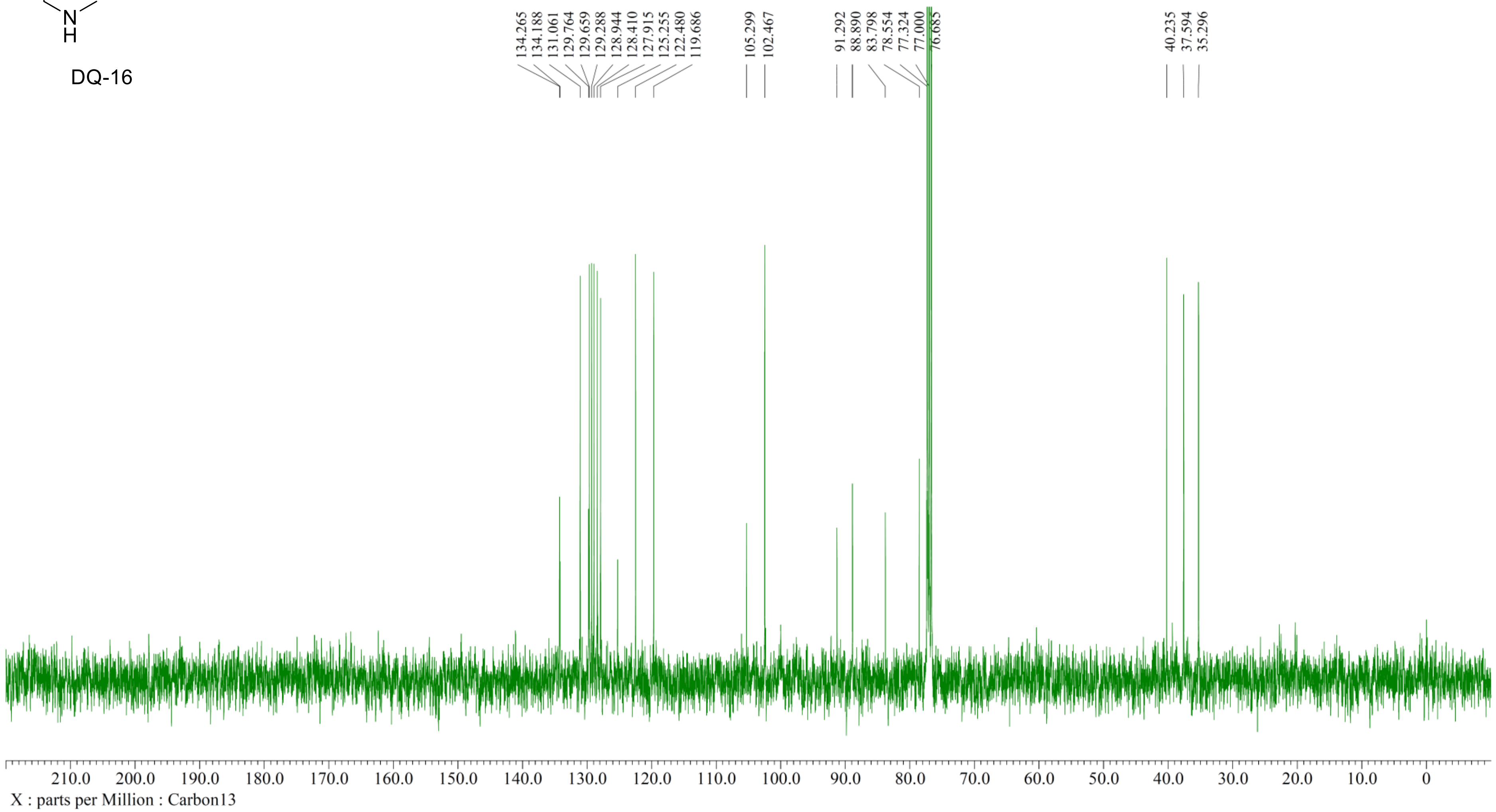

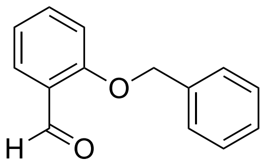

23a

^{1}H NMR (400 MHz, $CDCl_3$)

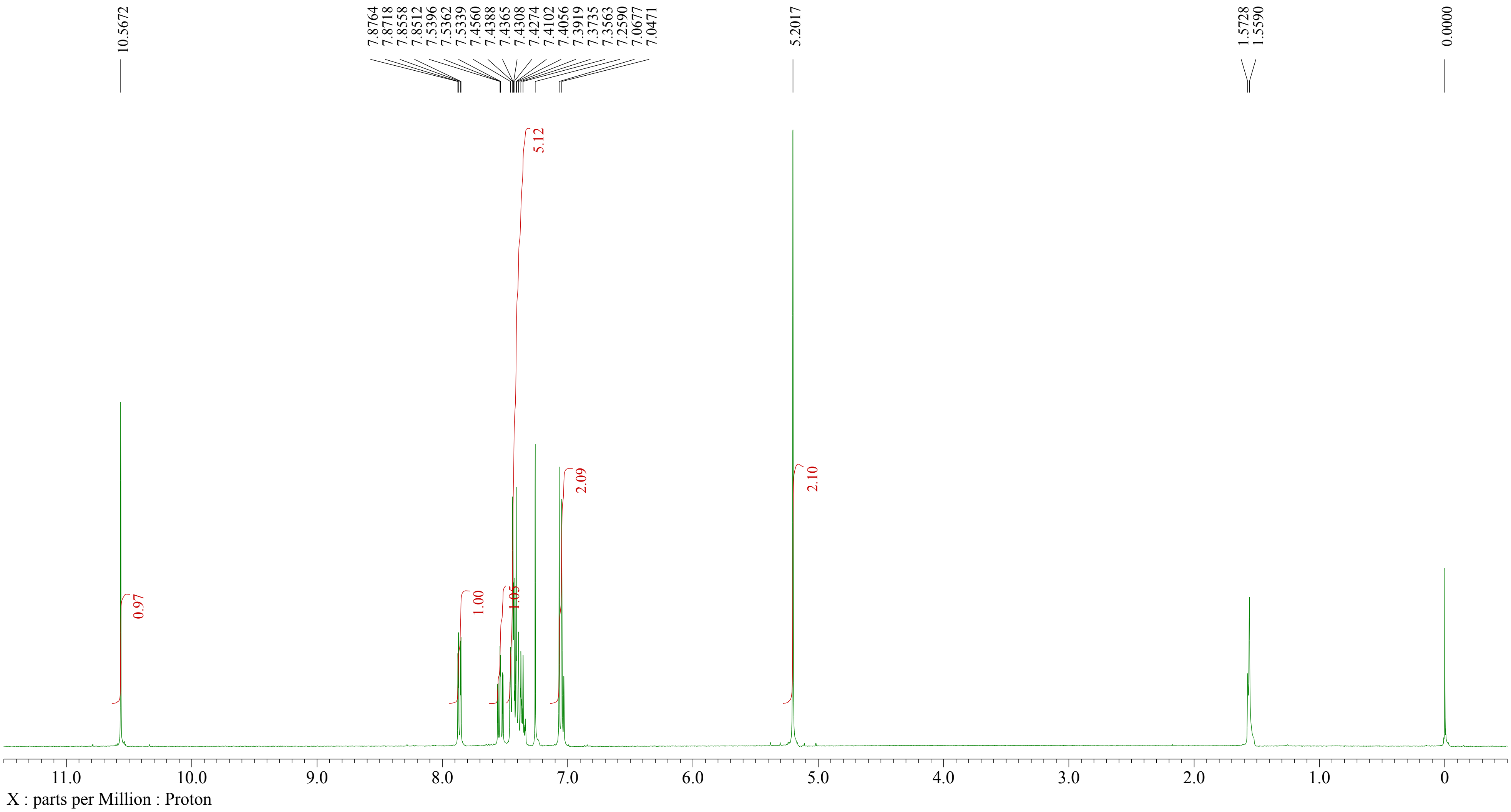

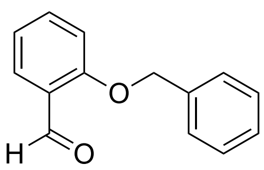

23a

^{13}C NMR (100 MHz, $CDCl_3$)

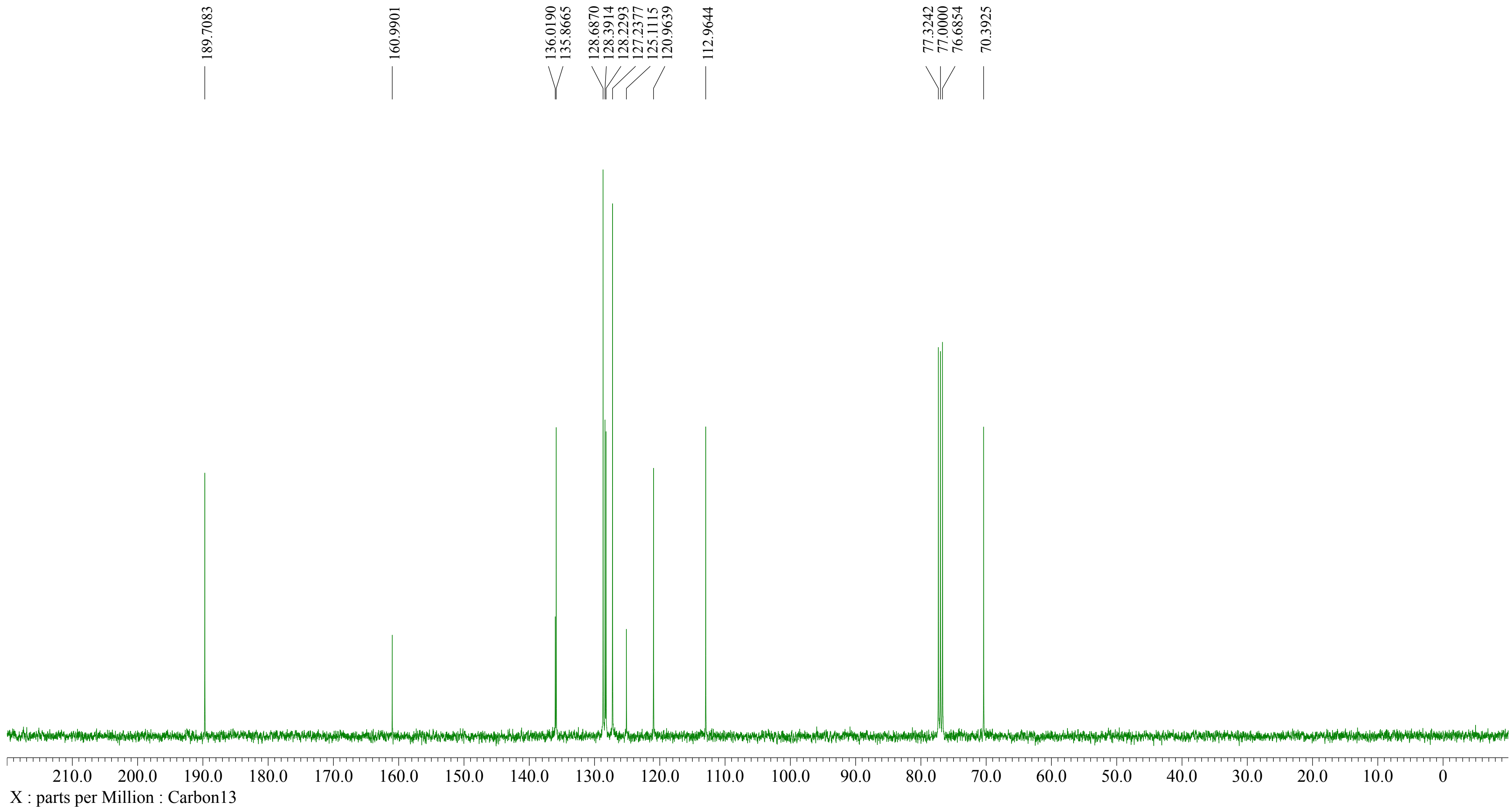

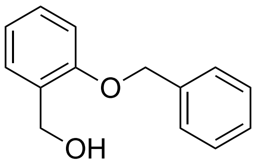

24a

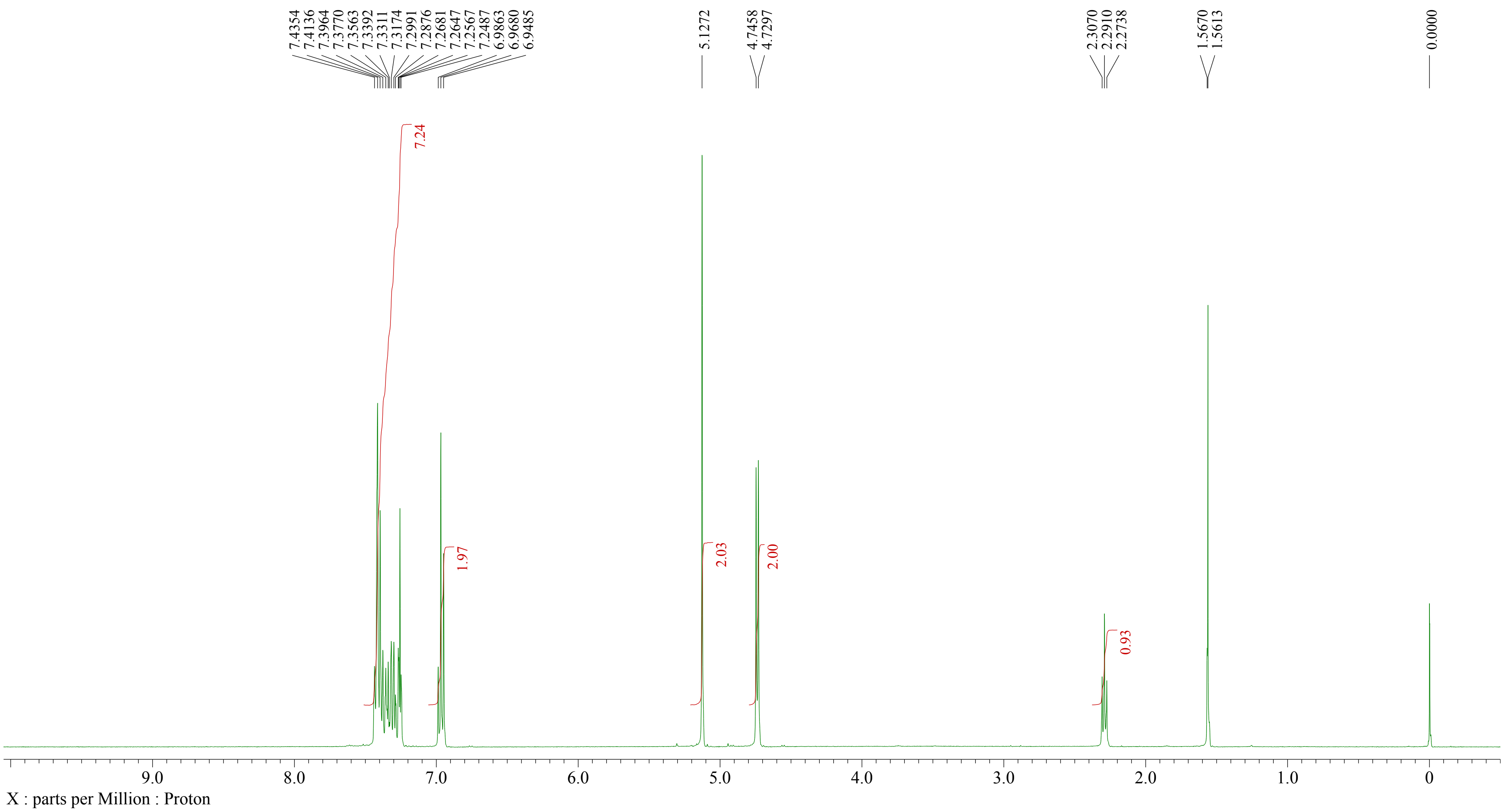

^{1}H NMR (400 MHz, $CDCl_3$)
7.4354
7.4136
7.3964
7.3770
7.3563
7.3392
7.3311
7.3174
7.2991
7.2876
7.2681
7.2647
7.2567
7.2487
6.9863
6.9680
6.9485
5.1272
4.7458
4.7297
2.3070
2.2910
2.2738
1.5670
1.5613
0.0000
7.24
1.97
2.03
2.00
0.93
9.0
8.0
7.0
6.0
5.0
4.0
3.0
2.0
1.0
0
X : parts per Million : Proton

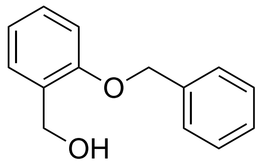

24a

^{13}C NMR (100 MHz, $CDCl_3$)

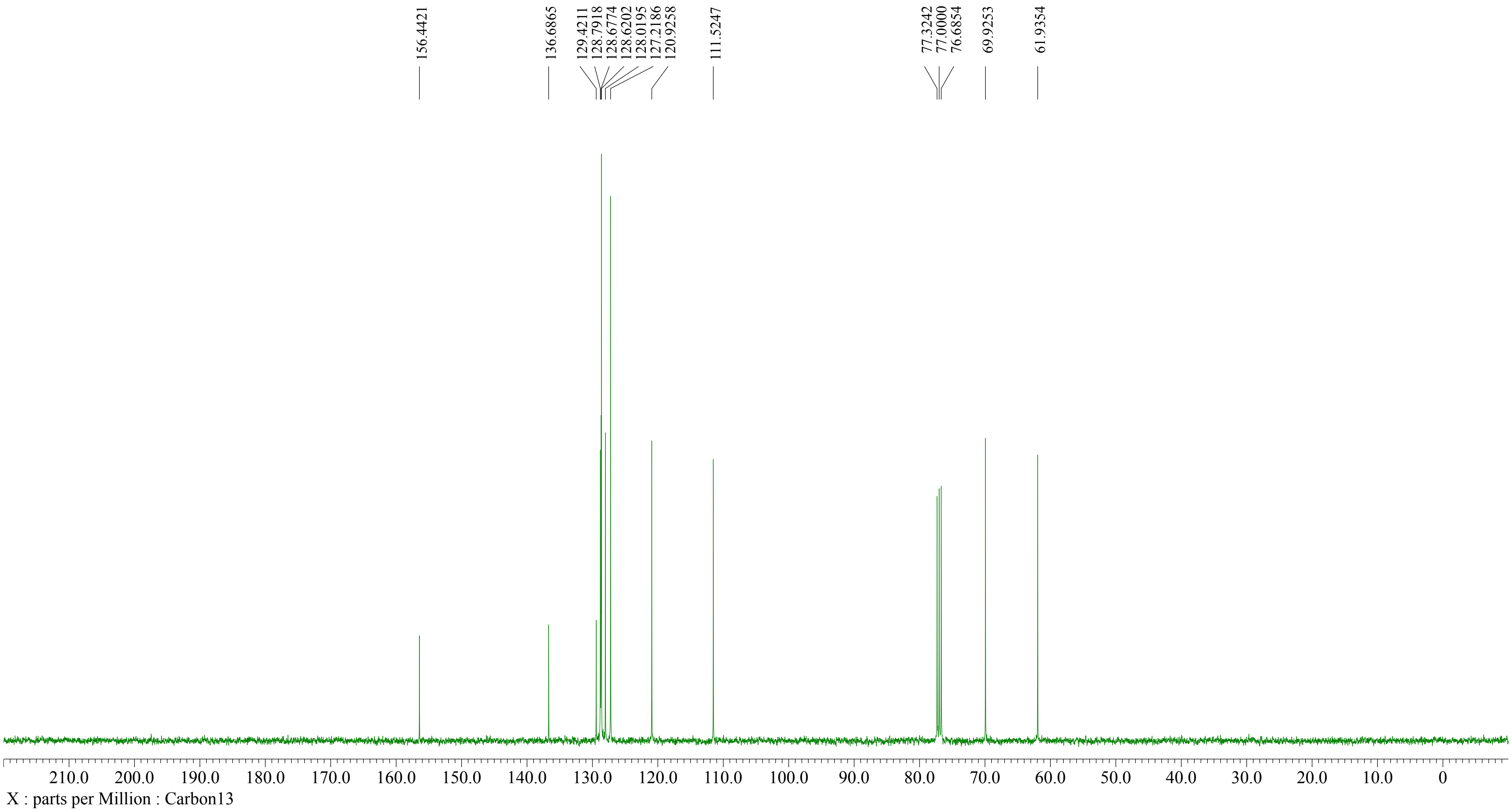

^1H NMR (400 MHz, $CDCl_3$)

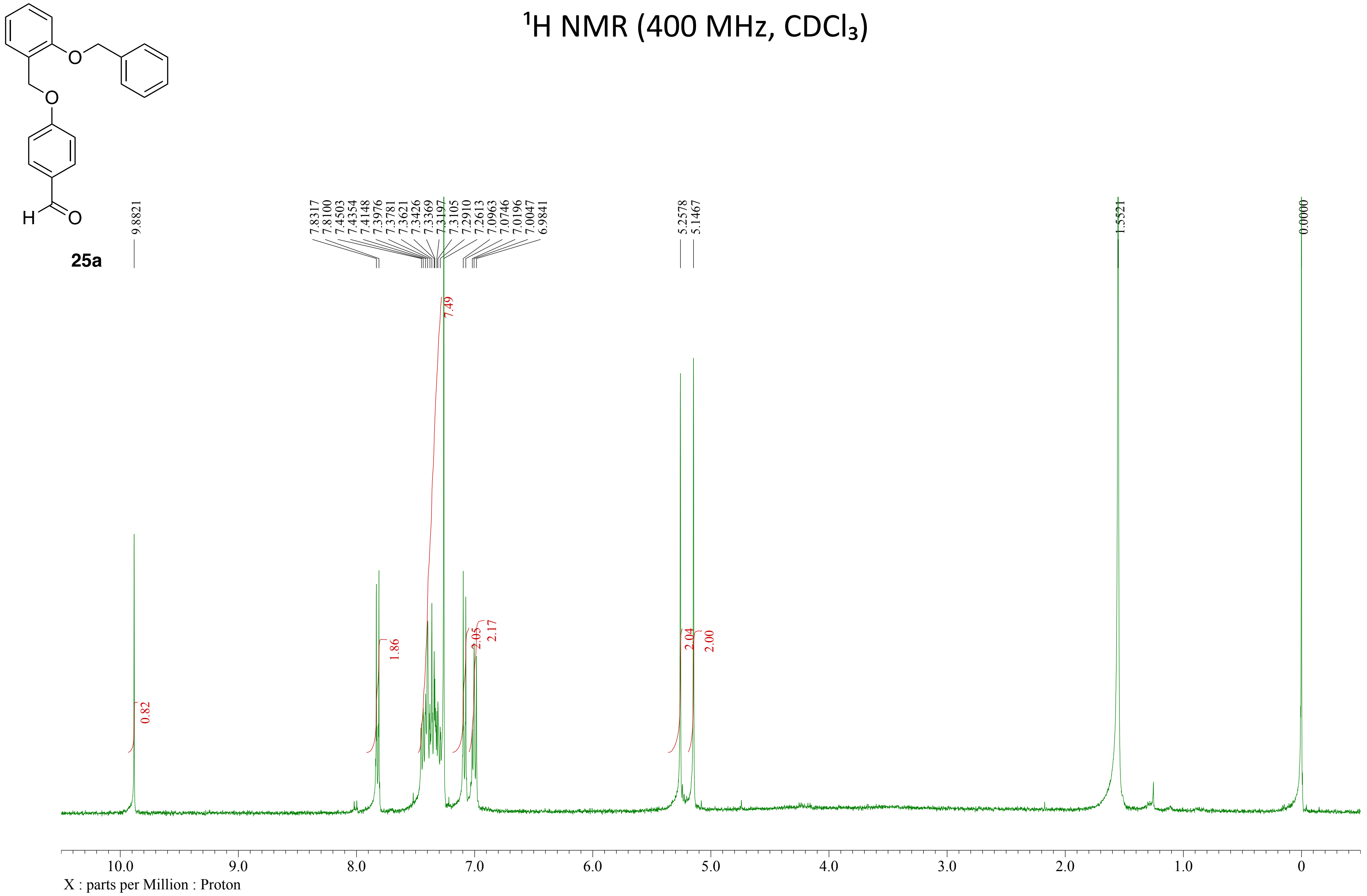

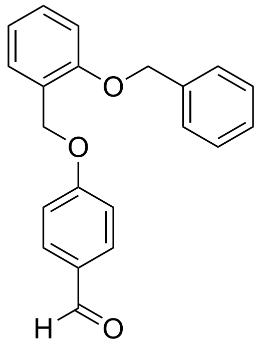

25a

^{13}C NMR (100 MHz, $CDCl_3$)

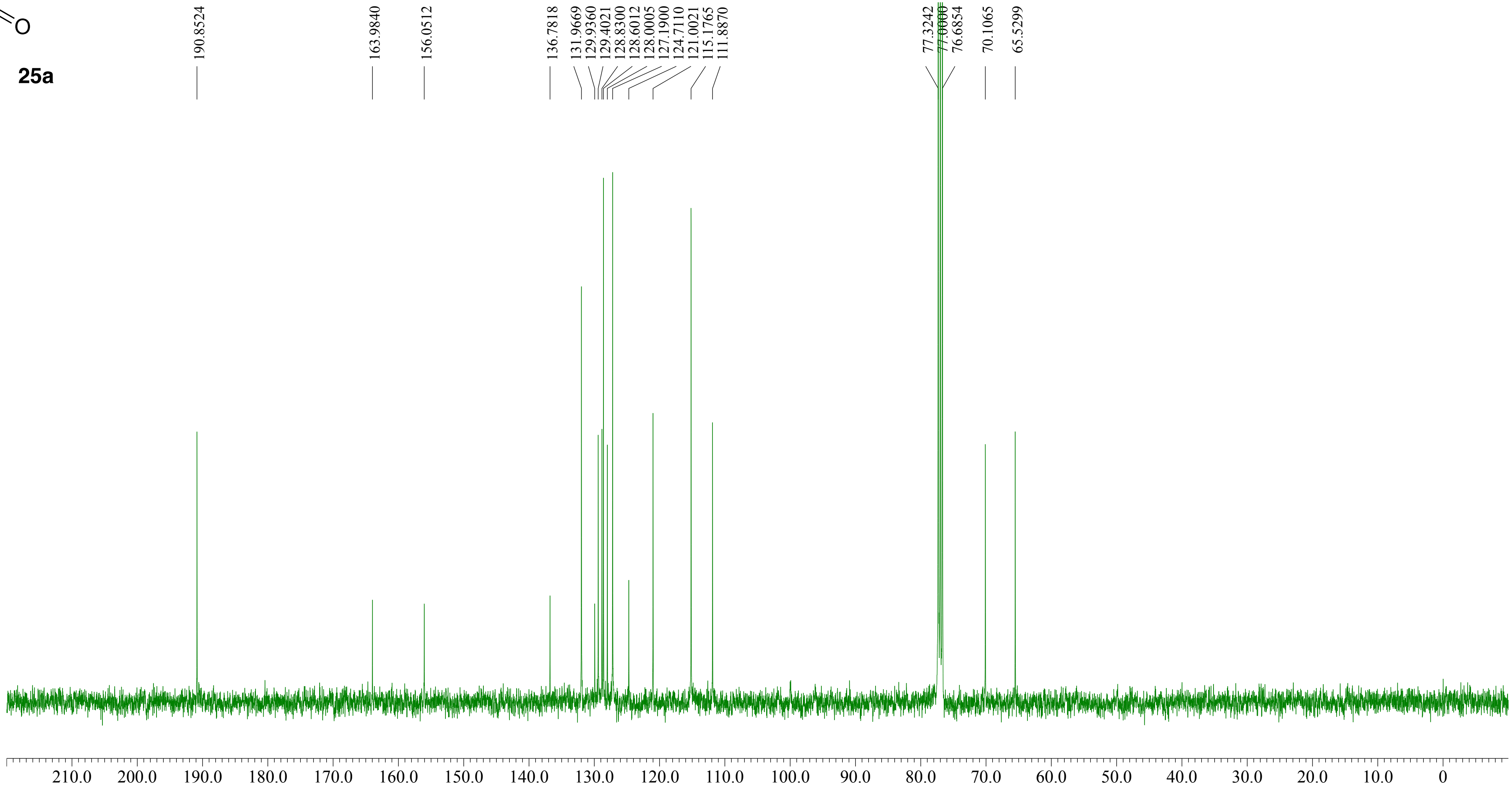

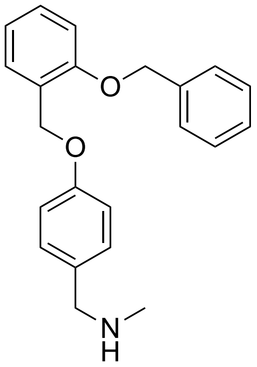

DQ-06

^{1}H NMR (400 MHz, $CDCl_3$)

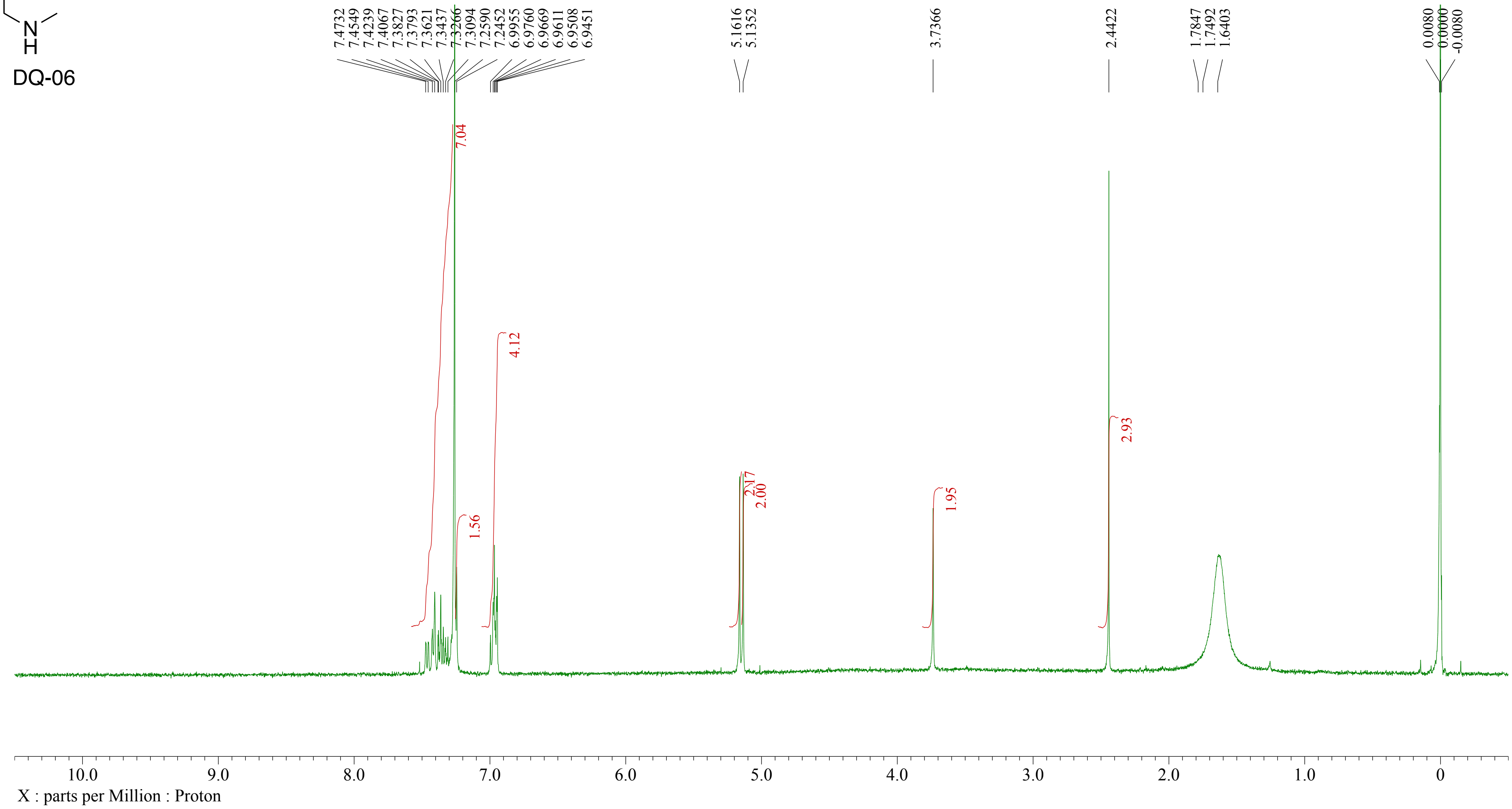

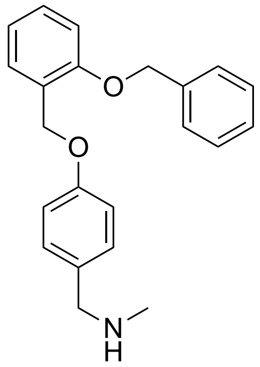

DQ-06

^{13}C NMR (100 MHz, $CDCl_3$)

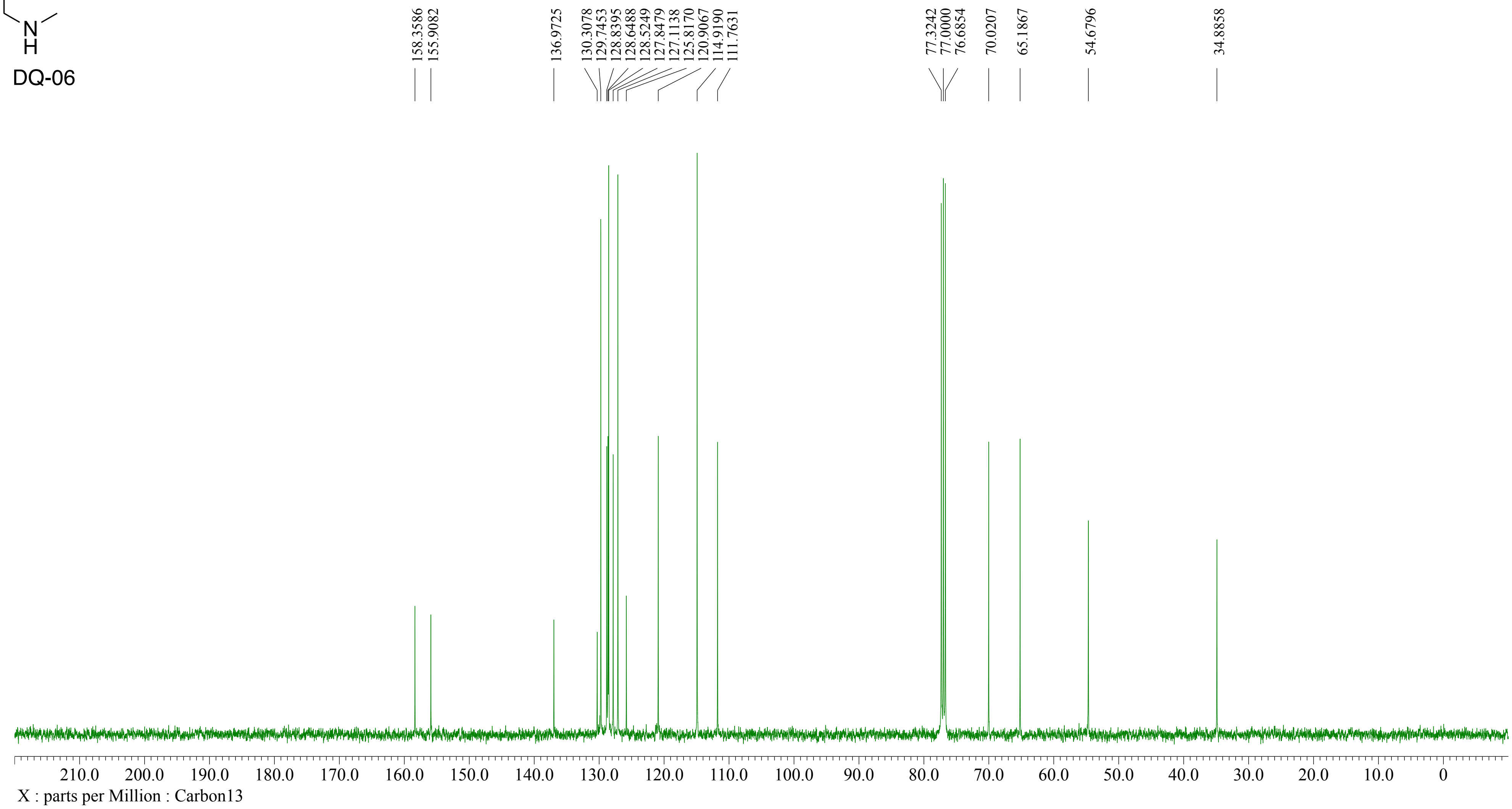

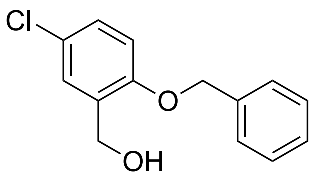

24b

^{1}H NMR (400 MHz, $CDCl_3$)

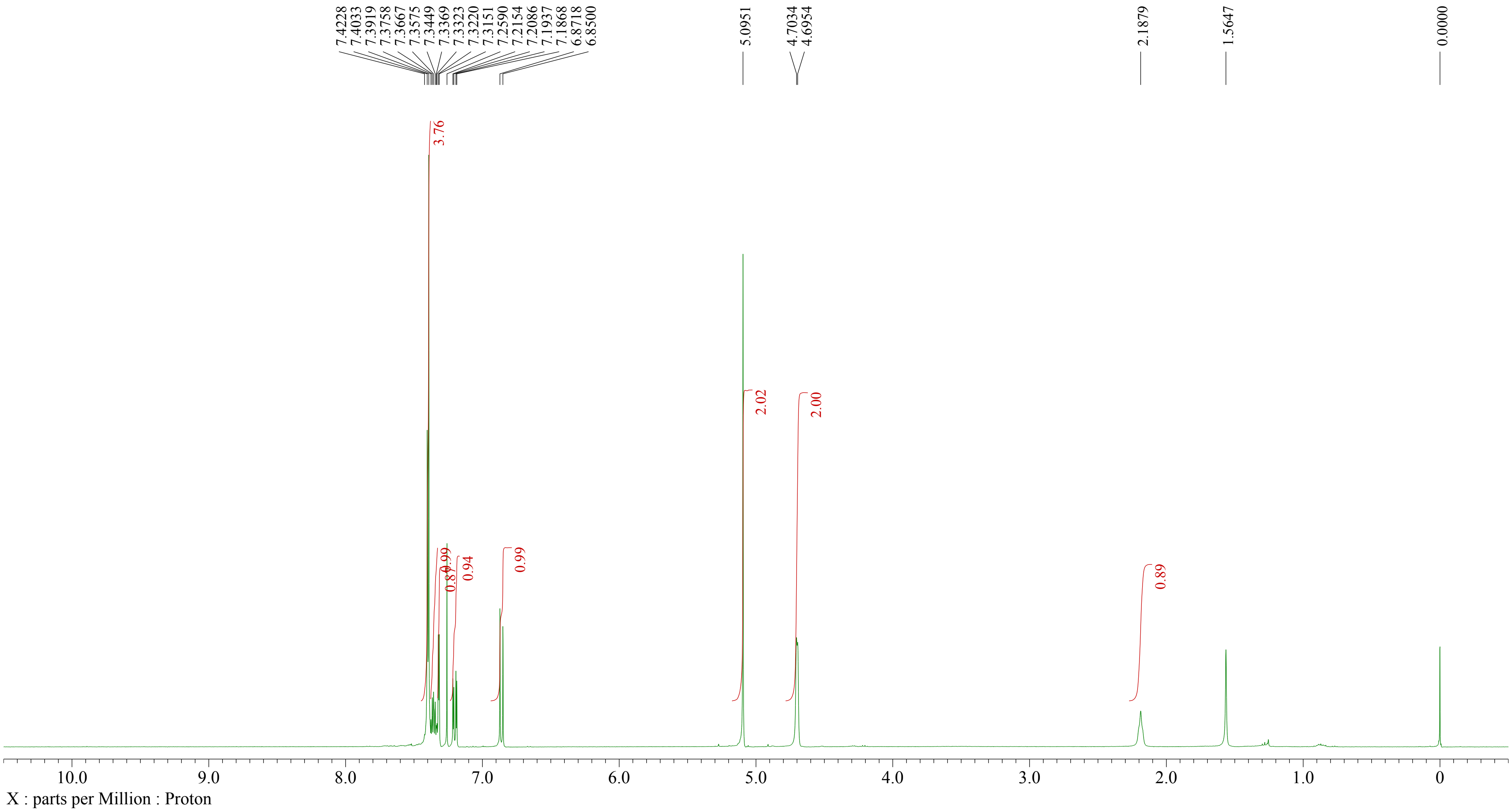

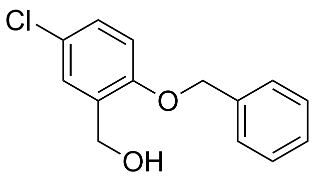

24b

^{13}C NMR (100 MHz, $CDCl_3$)

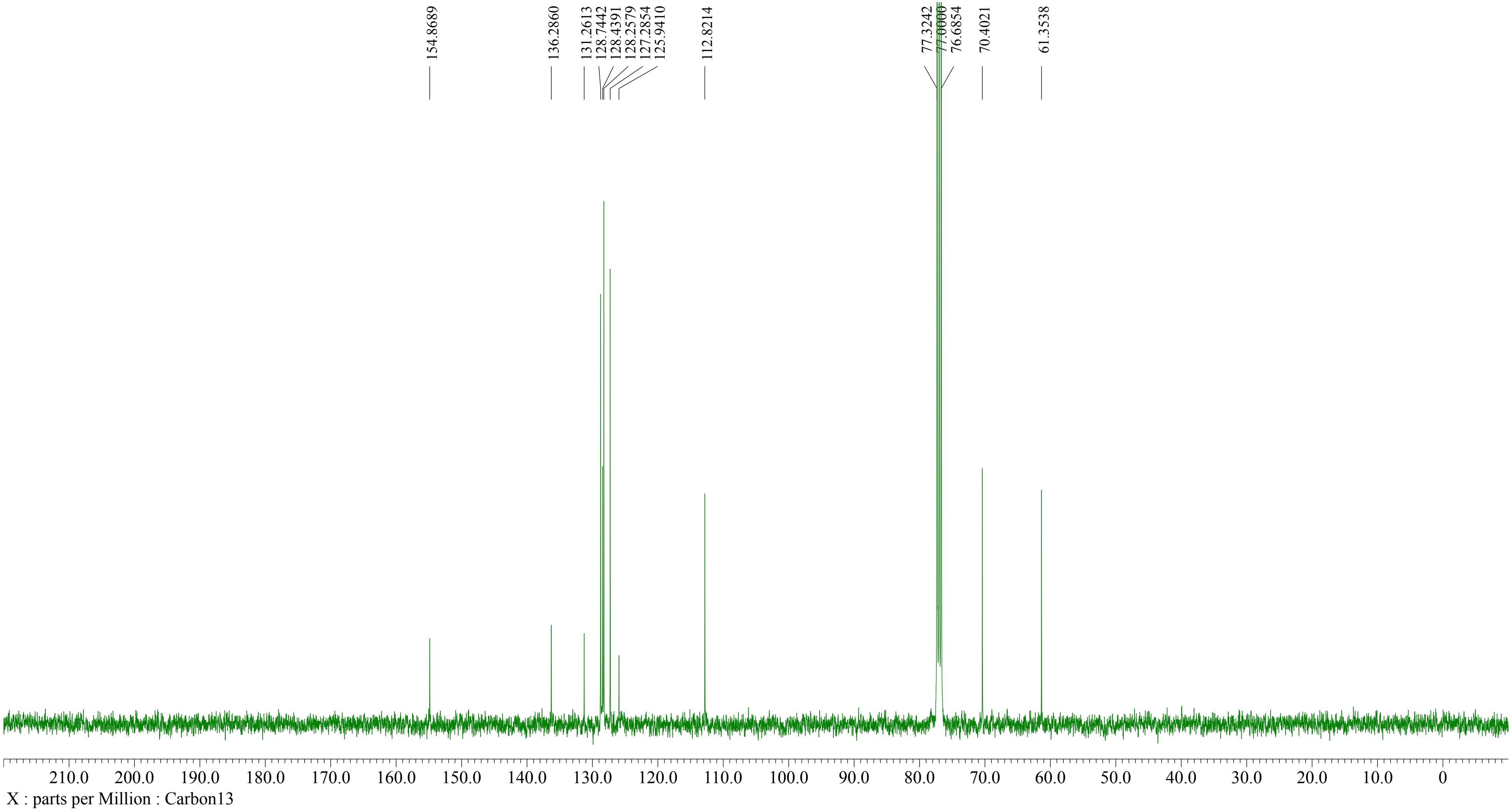

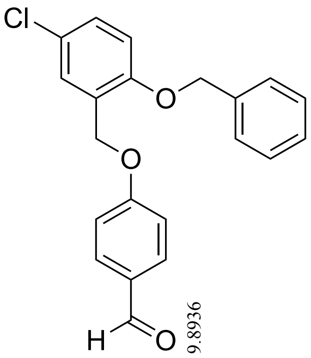

25b

^{1}H NMR (400 MHz, $CDCl_3$)

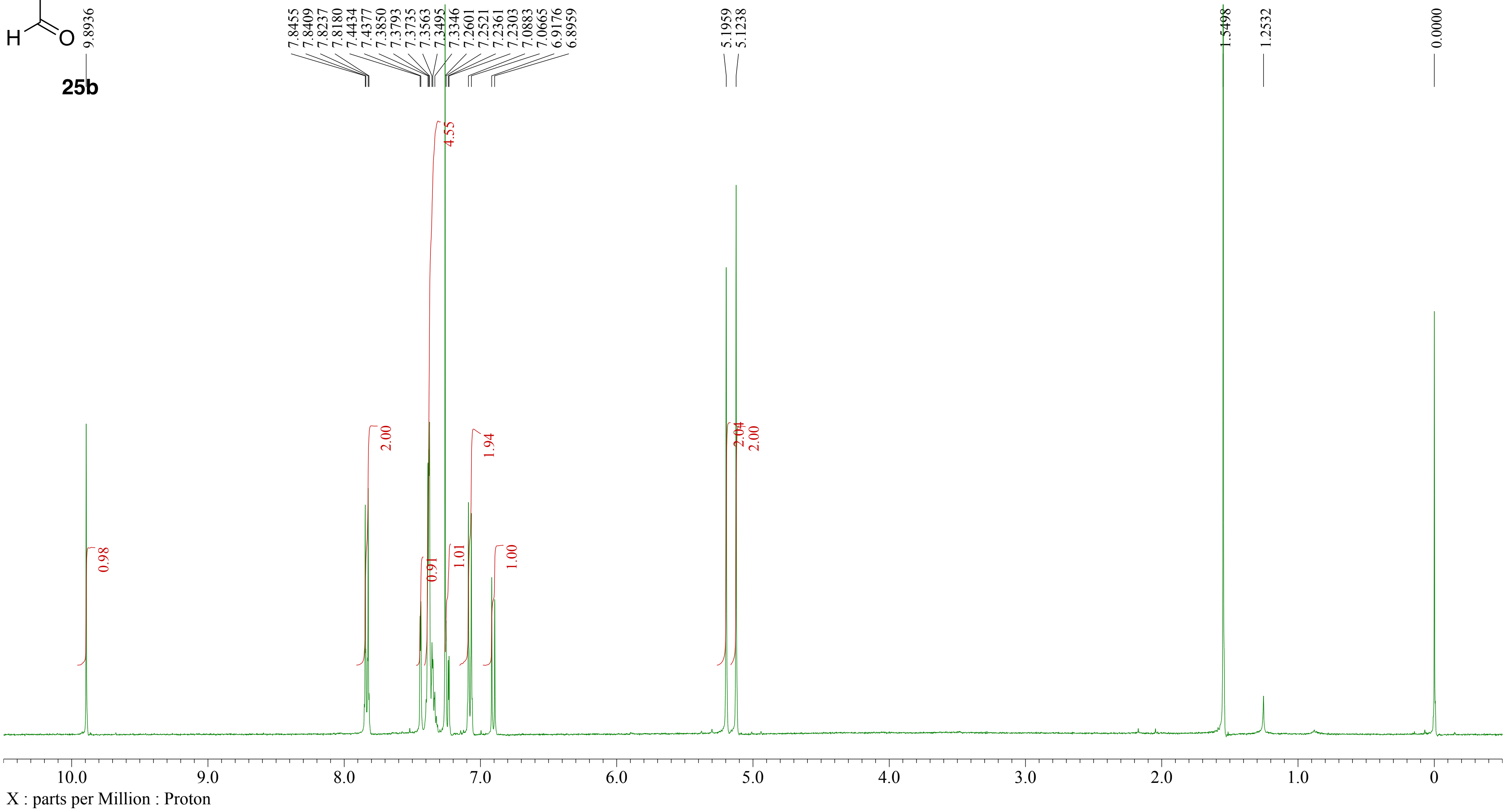

^{13}C NMR (100 MHz, $CDCl_3$)

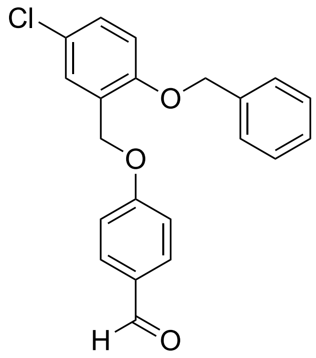

25b

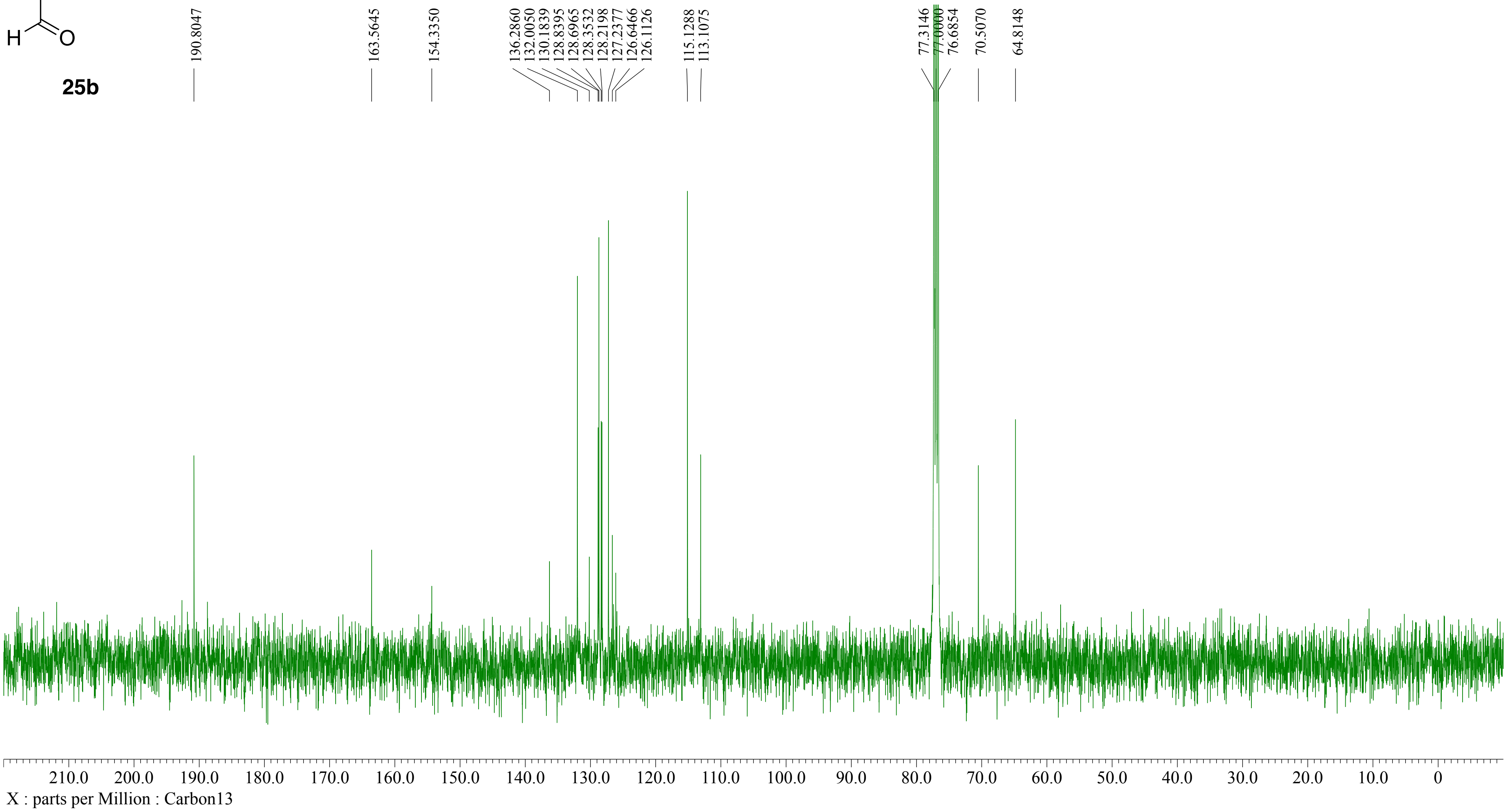

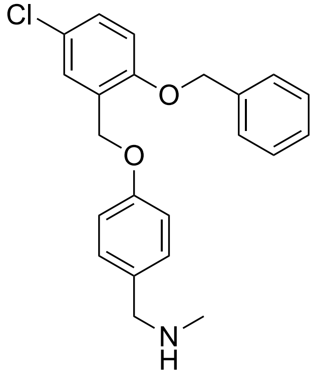

DQ-18

^{1}H NMR (400 MHz, $CDCl_3$)

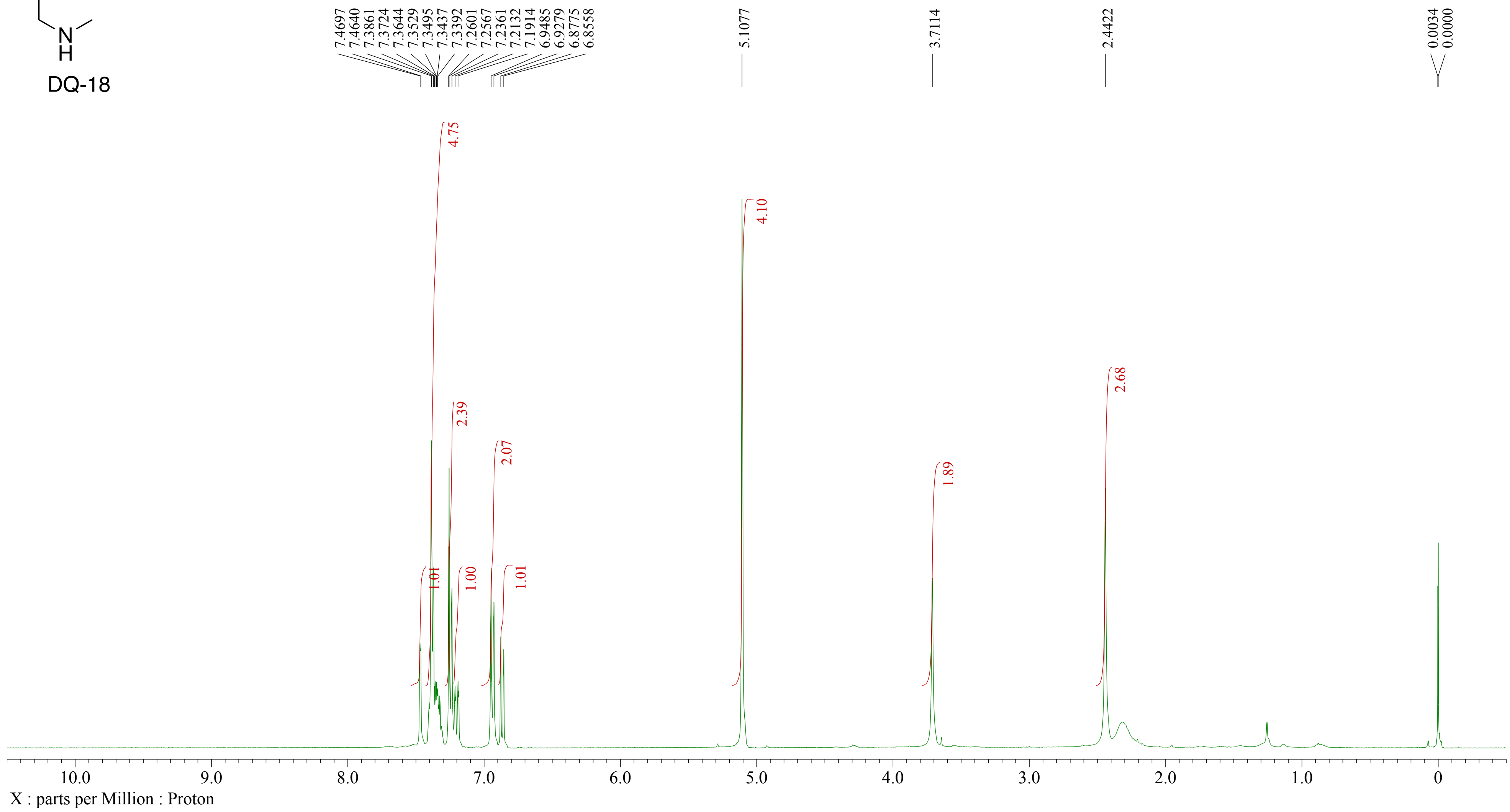

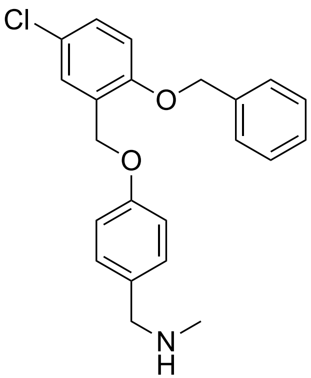

DQ-18

^{13}C NMR (100 MHz, $CDCl_3$)

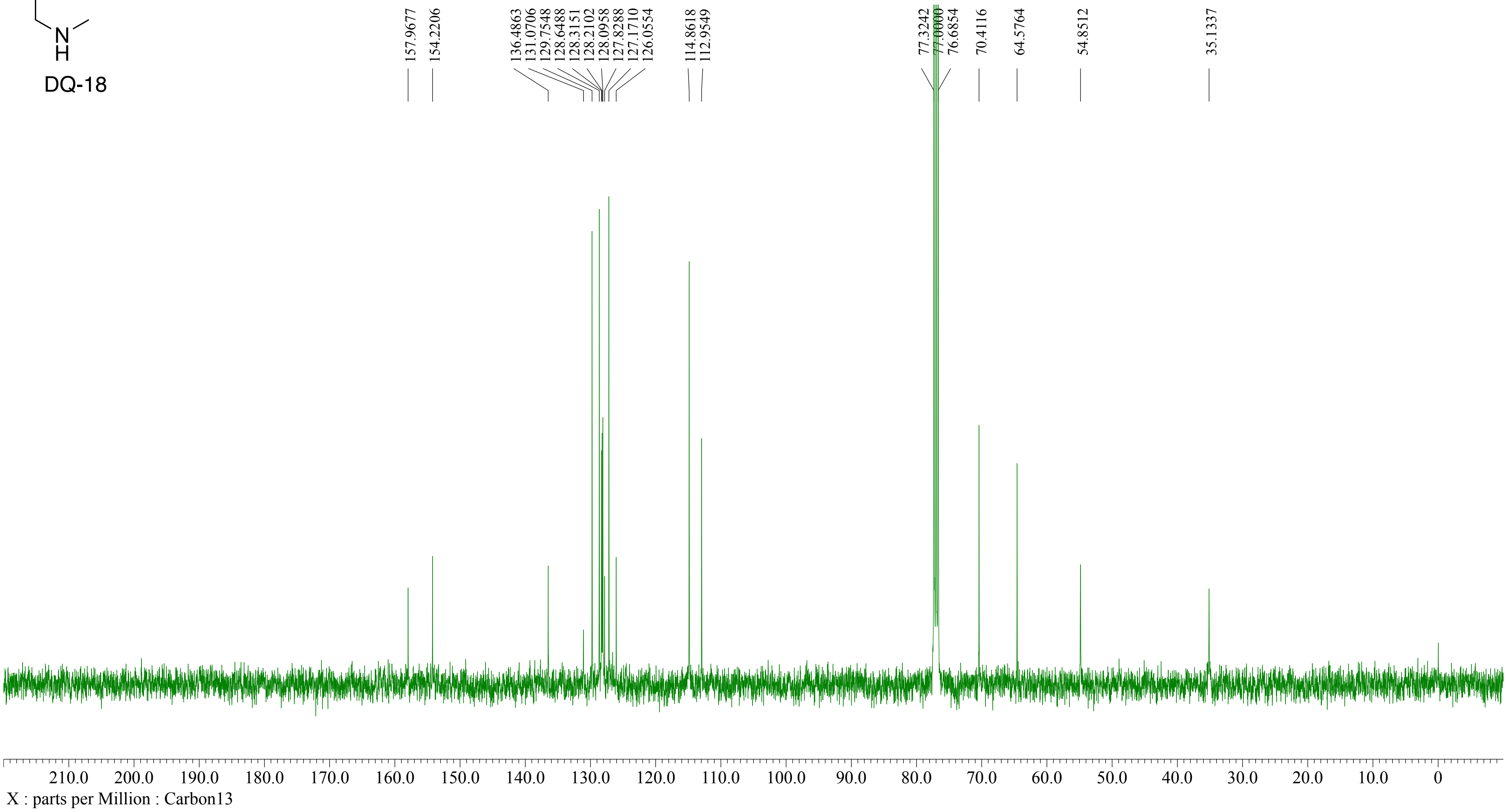

^{1}H NMR (400 MHz, $CDCl_3$)

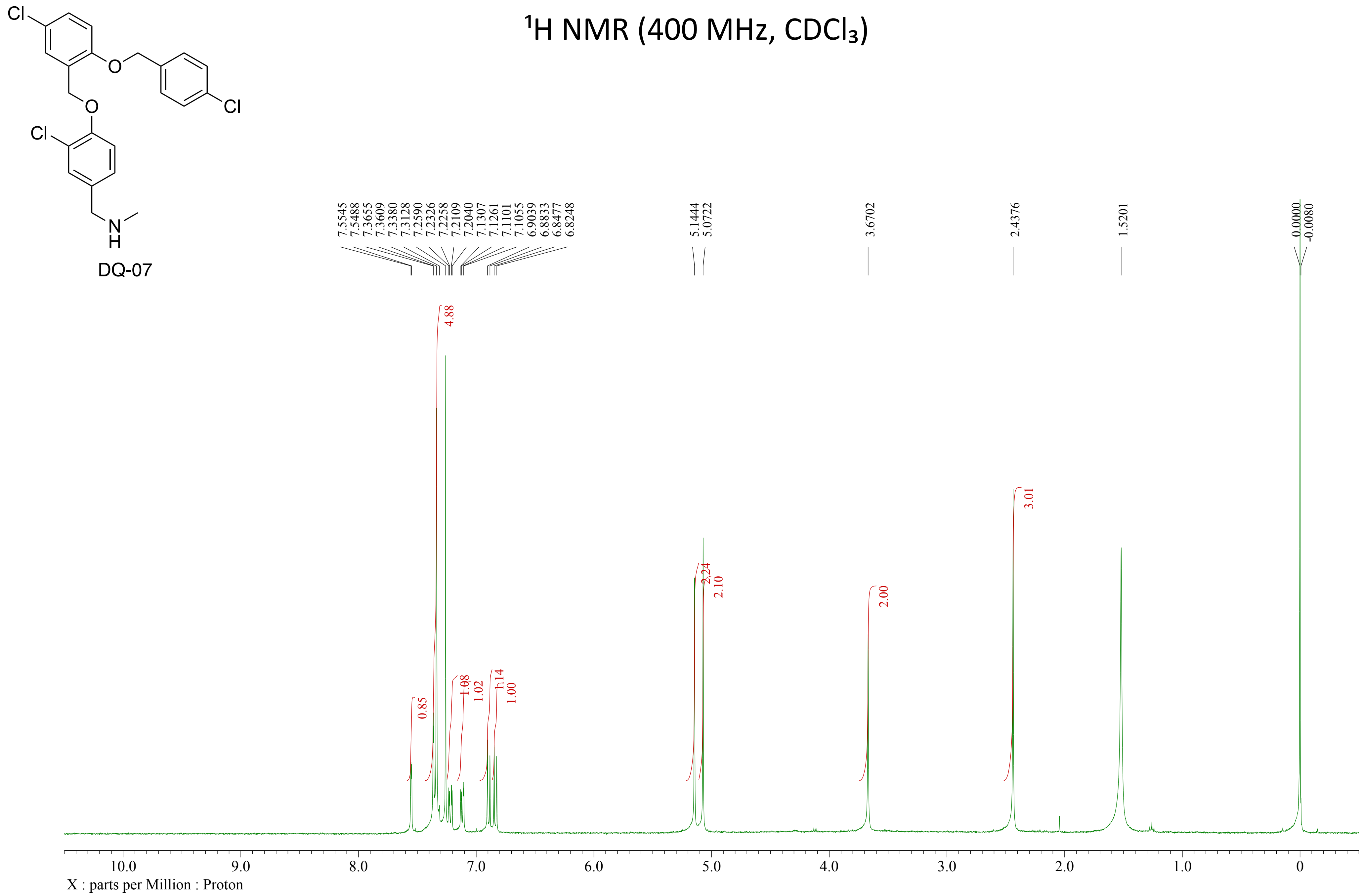

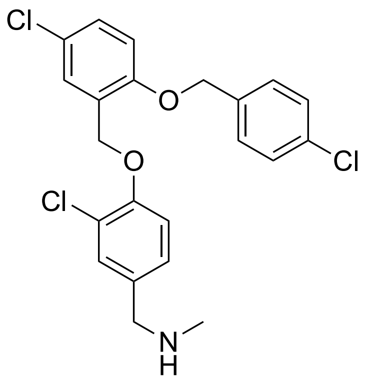

DQ-07

^{13}C NMR (100 MHz, $CDCl_3$)

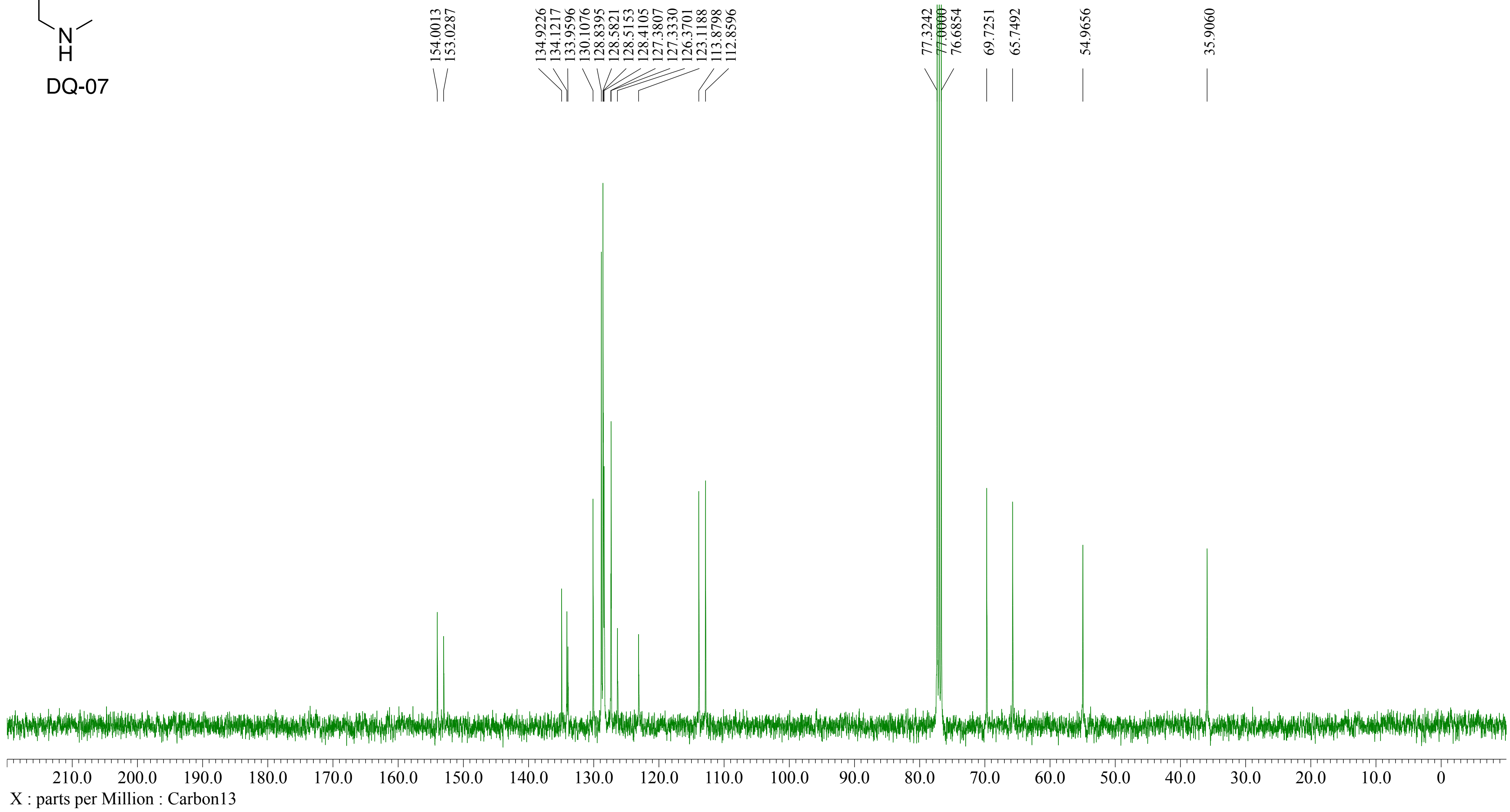

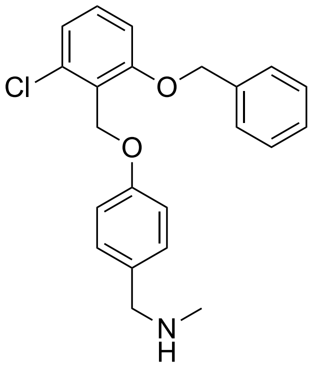

DQ-19

^{1}H NMR (400 MHz, $CDCl_3$)

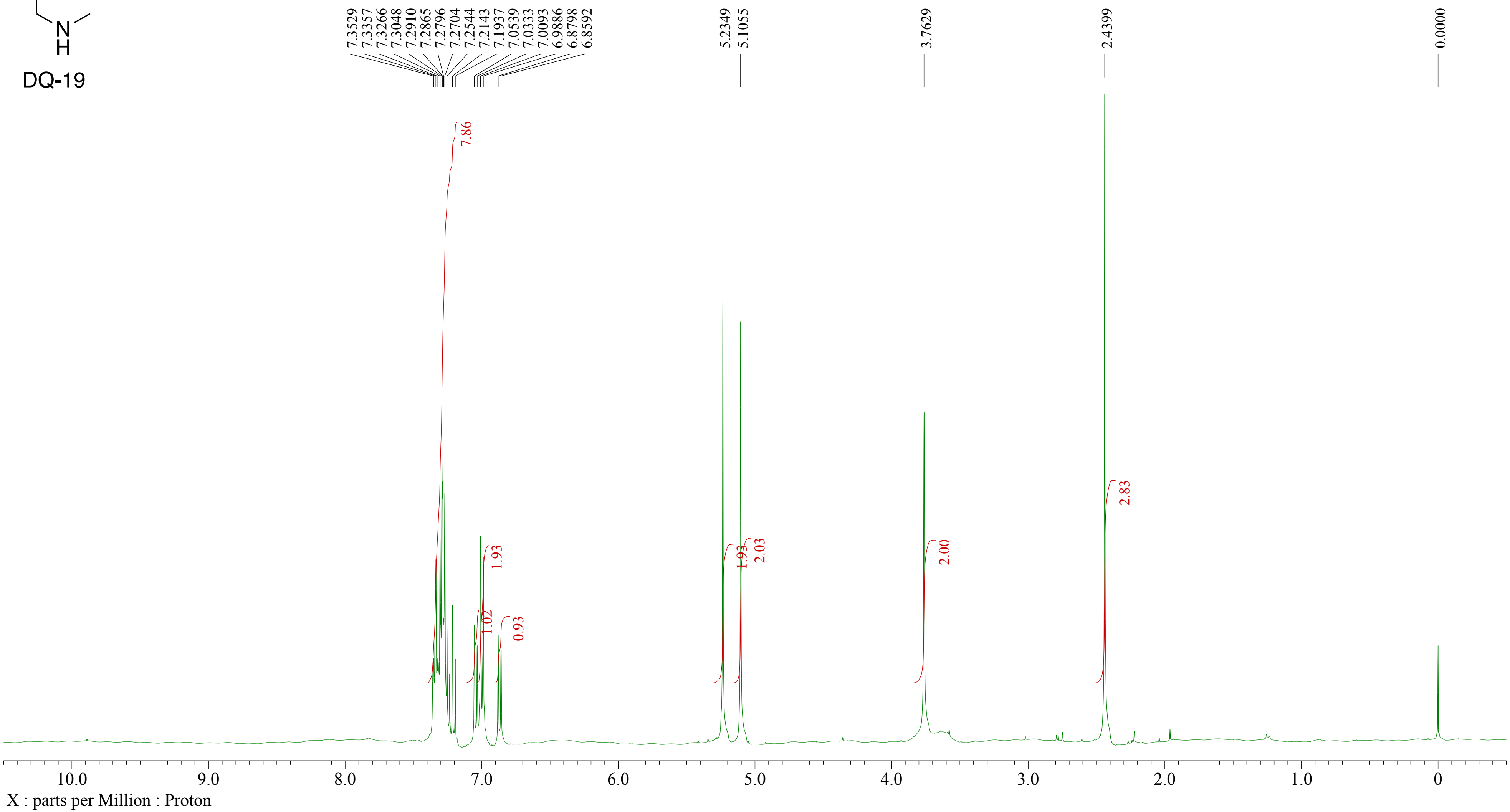

^{13}C NMR (100 MHz, $CDCl_3$)

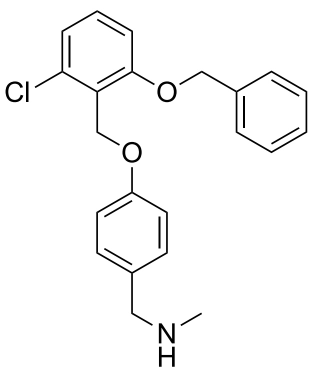

DQ-19

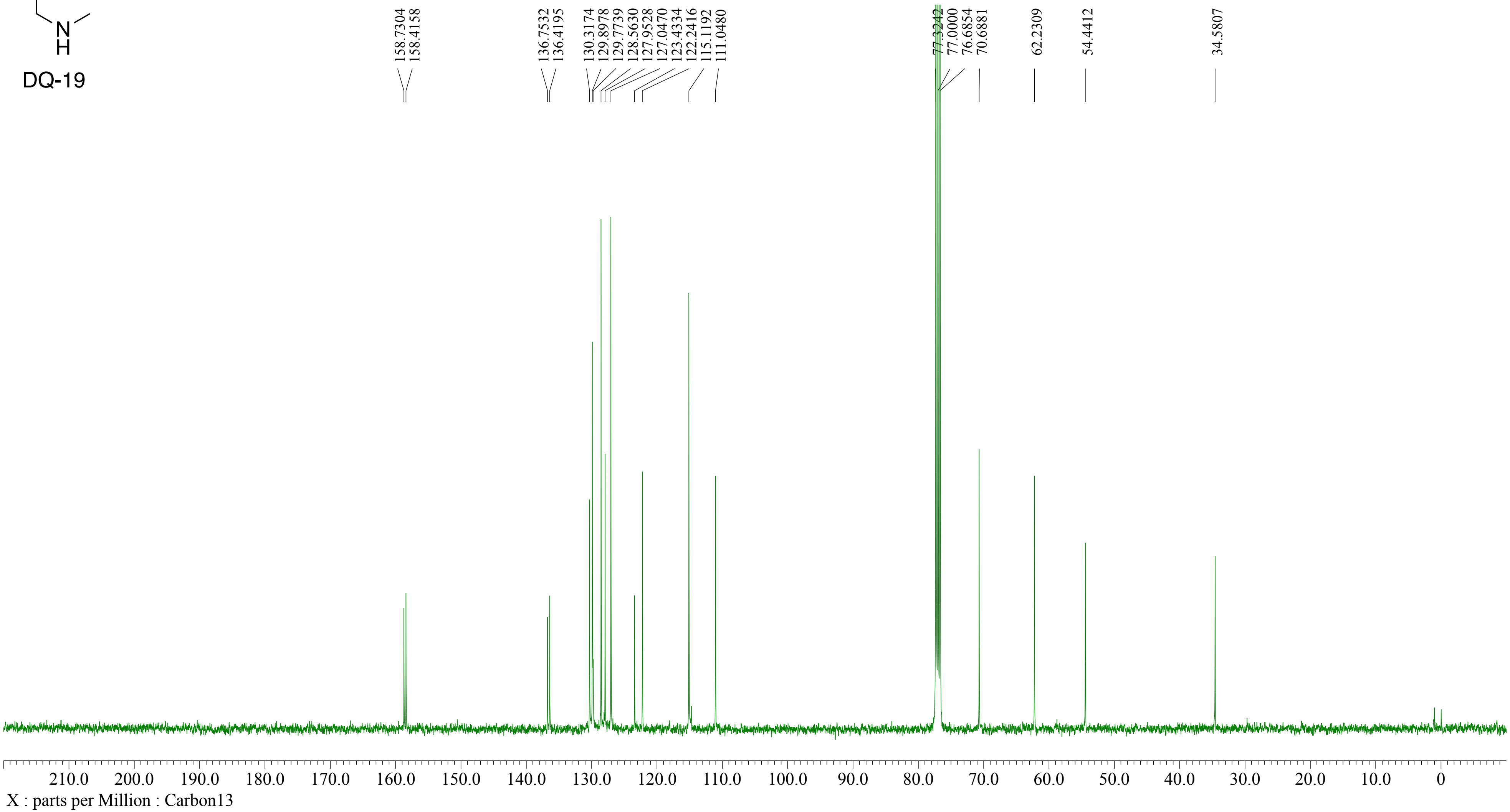

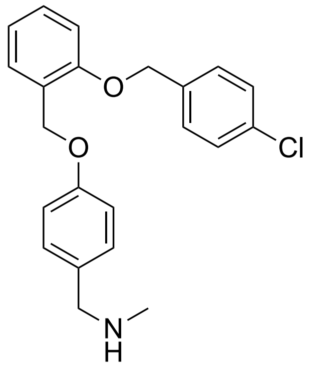

DQ-21

^1H NMR (400 MHz, $CDCl_3$)

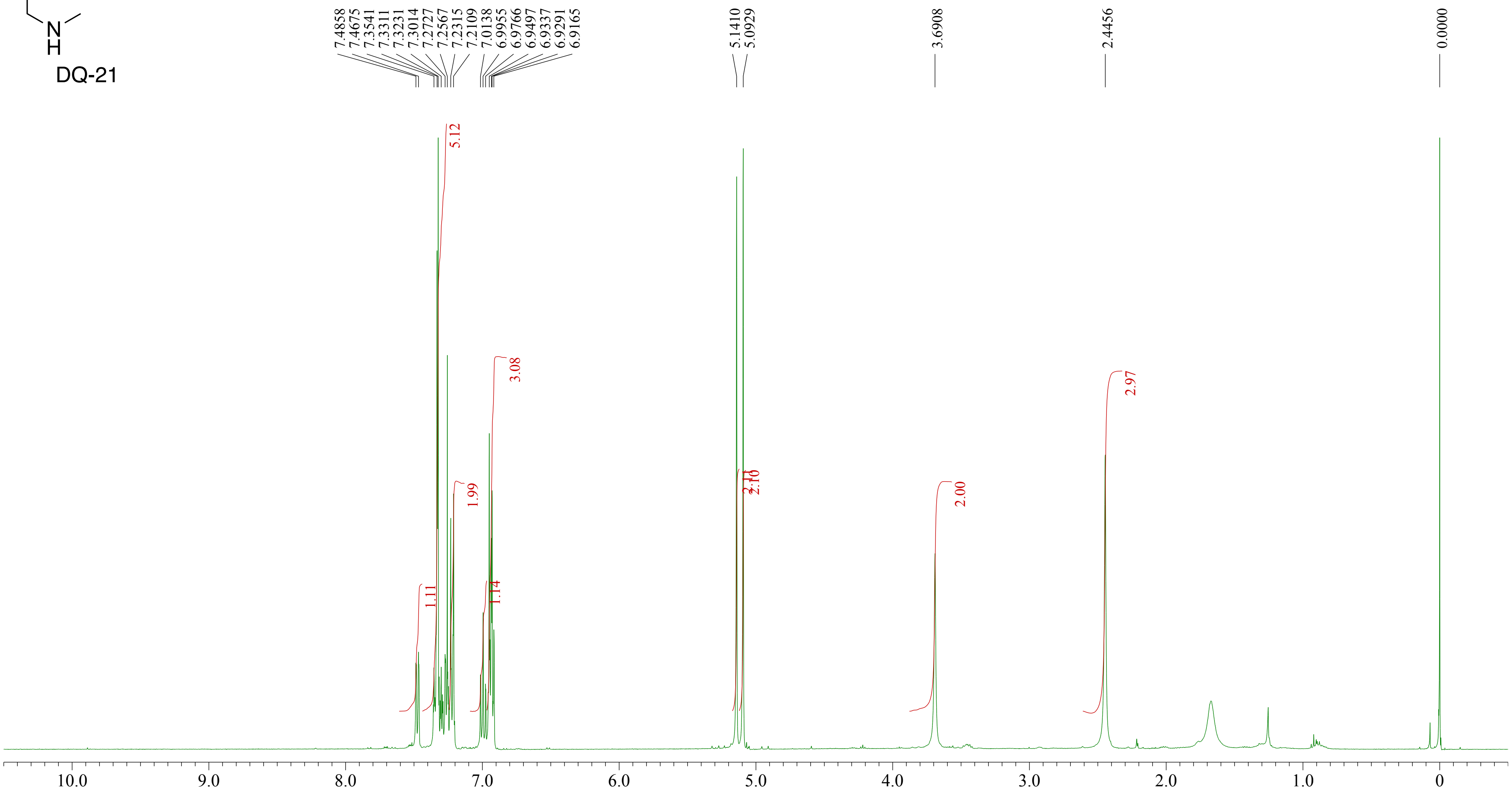

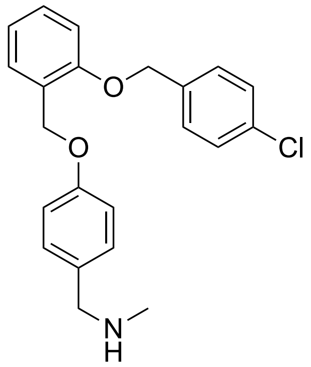

DQ-21

^{13}C NMR (100 MHz, $CDCl_3$)

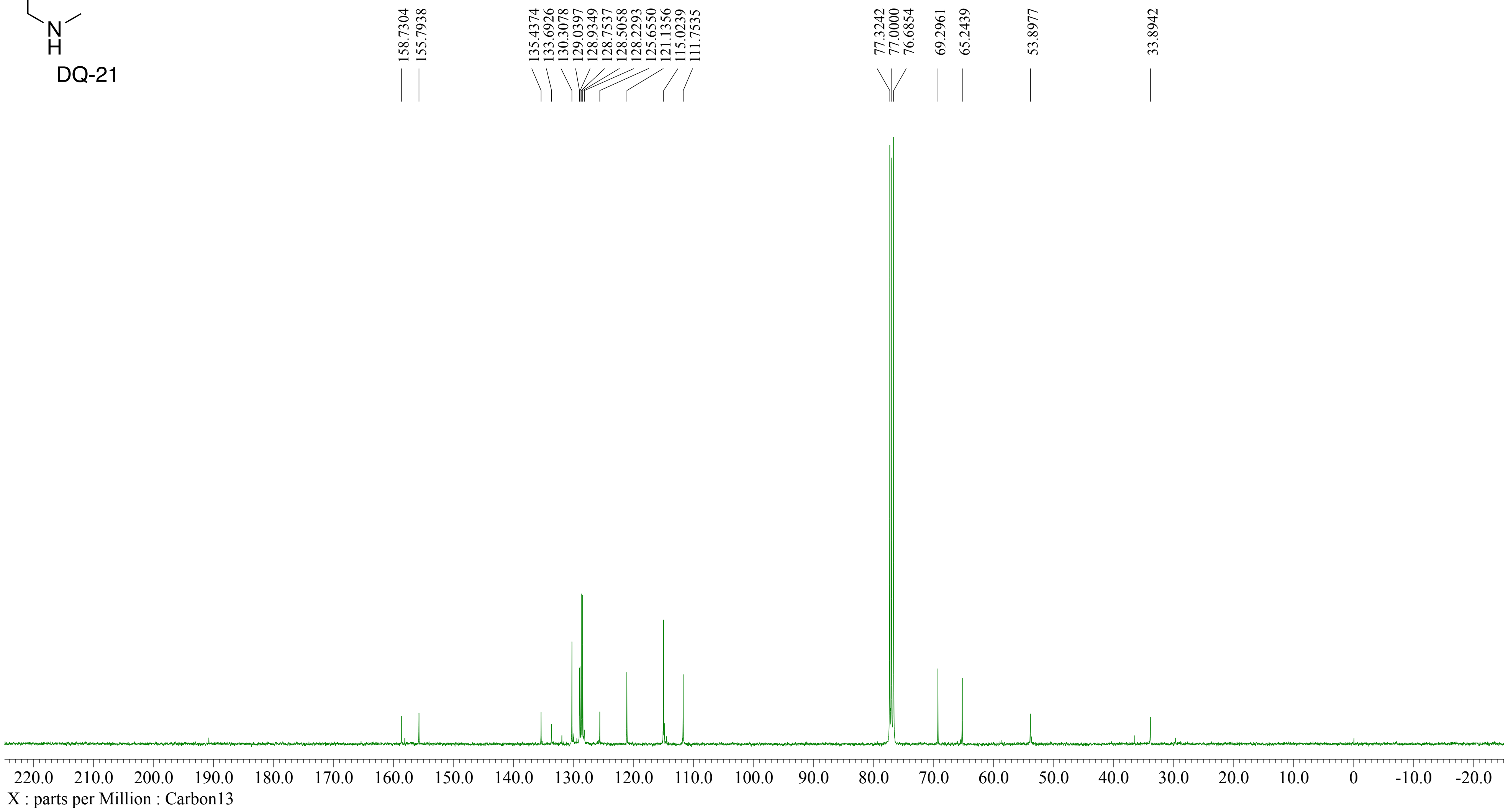

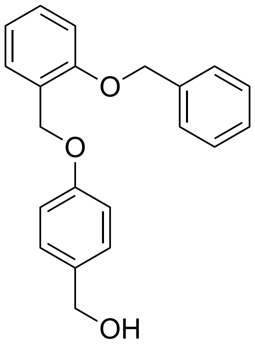

DQ-10

^{1}H NMR (400 MHz, $CDCl_3$)

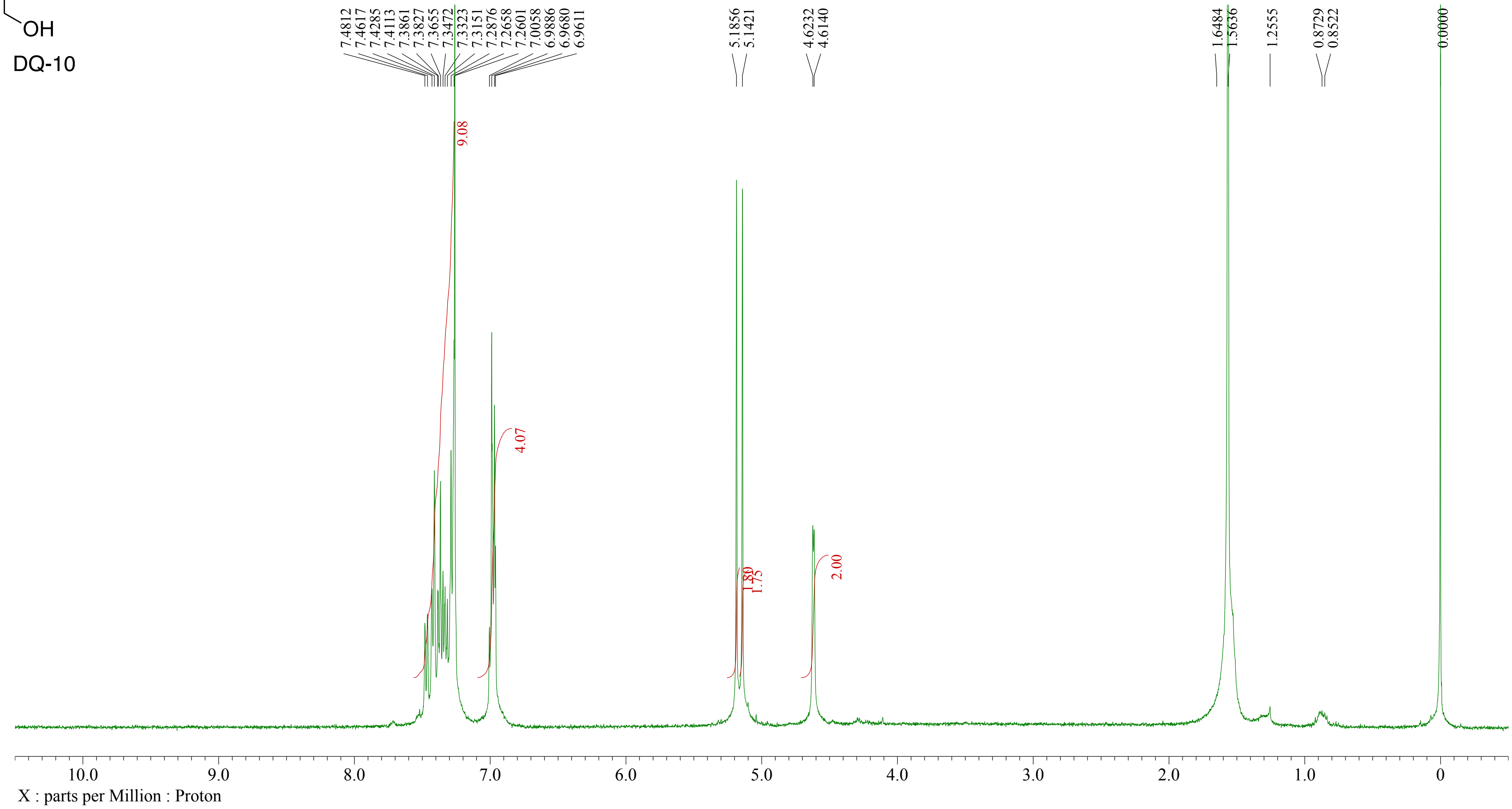

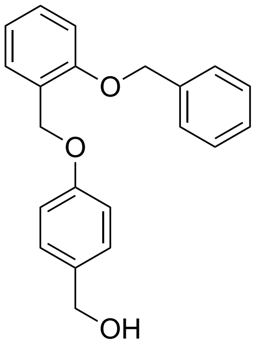

DQ-10

^{13}C NMR (100 MHz, $CDCl_3$)

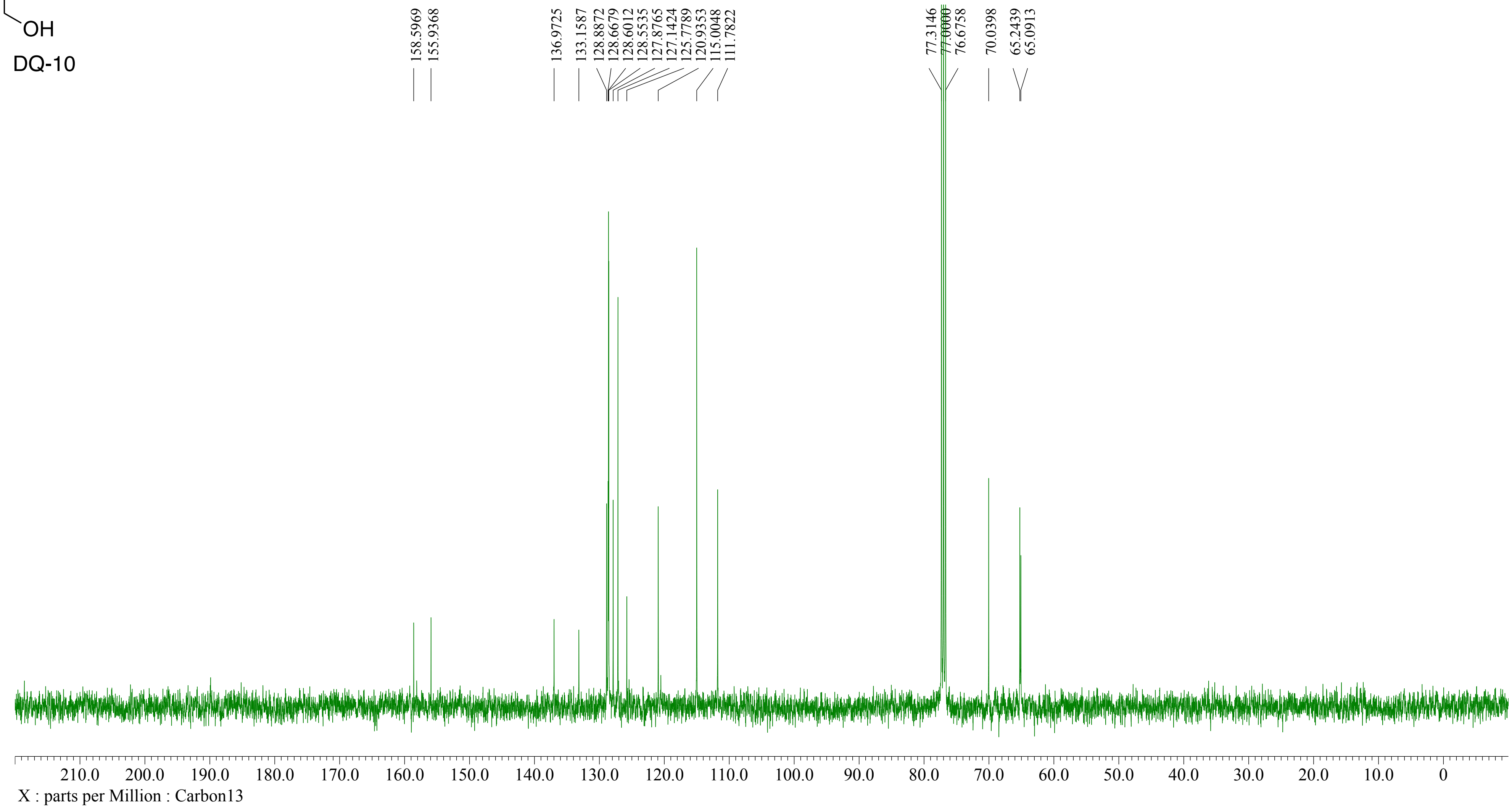

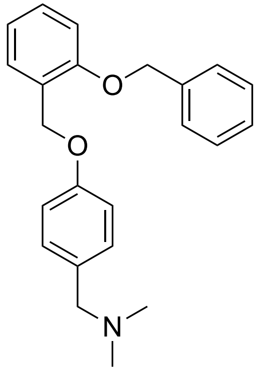

DQ-11

^1H NMR (400 MHz, $CDCl_3$)

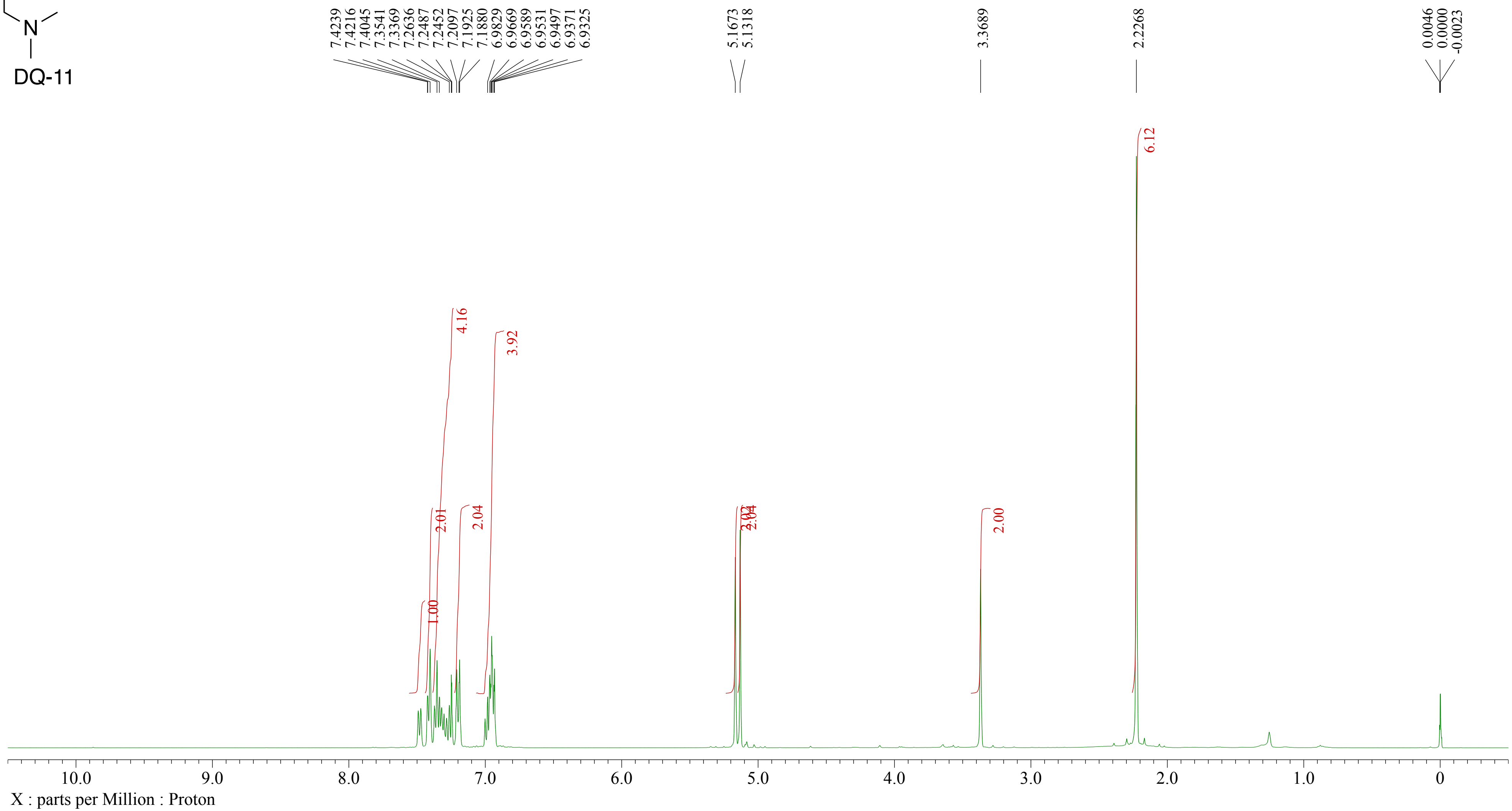

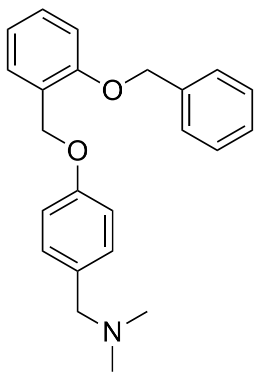

DQ-11

^{13}C NMR (100 MHz, $CDCl_3$)

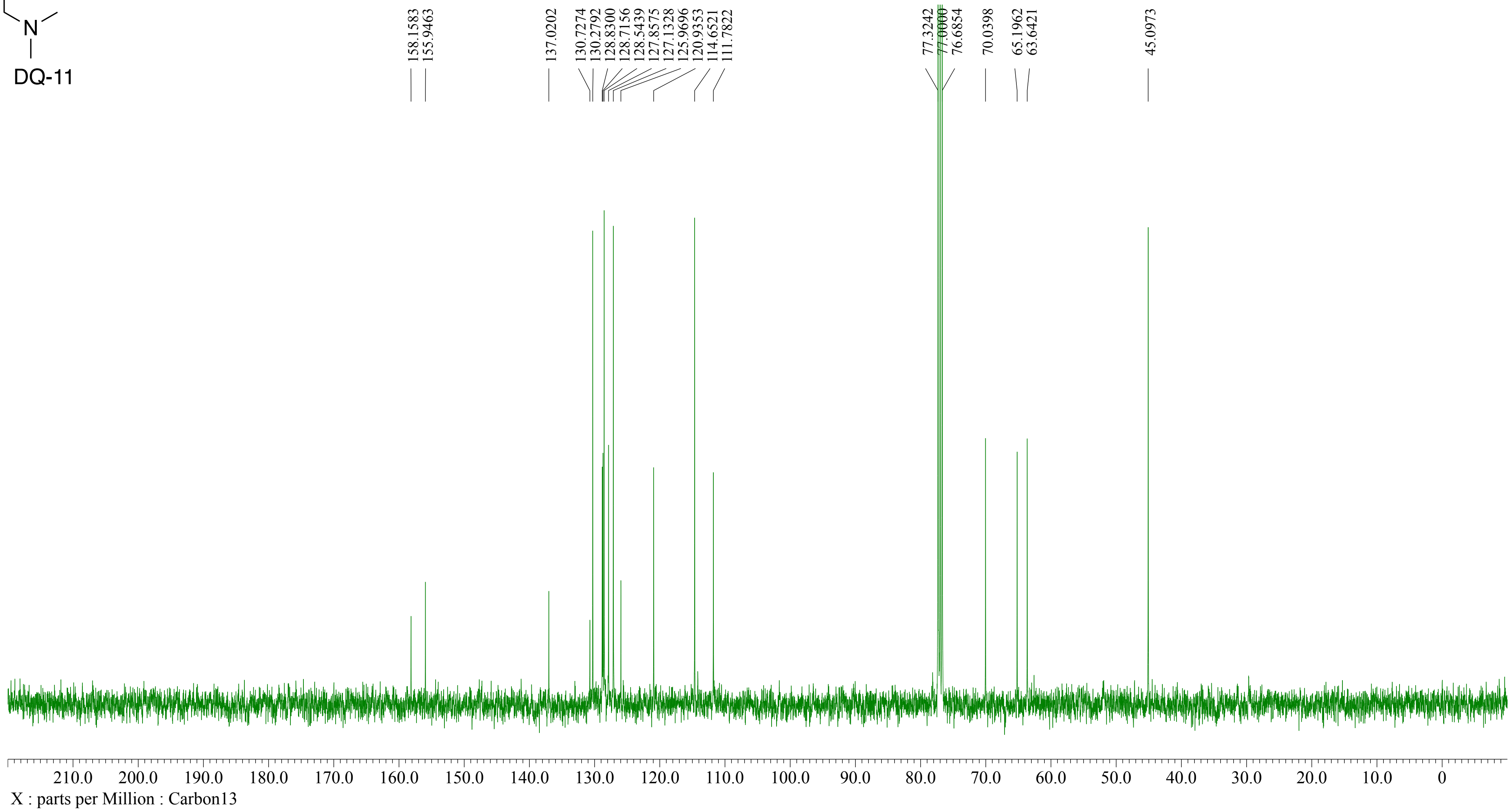

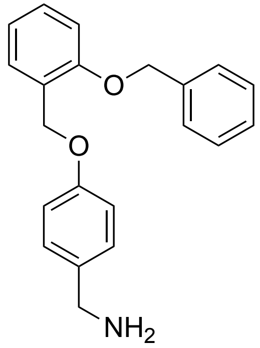

DQ-12

^{1}H NMR (400 MHz, $CDCl_3$)

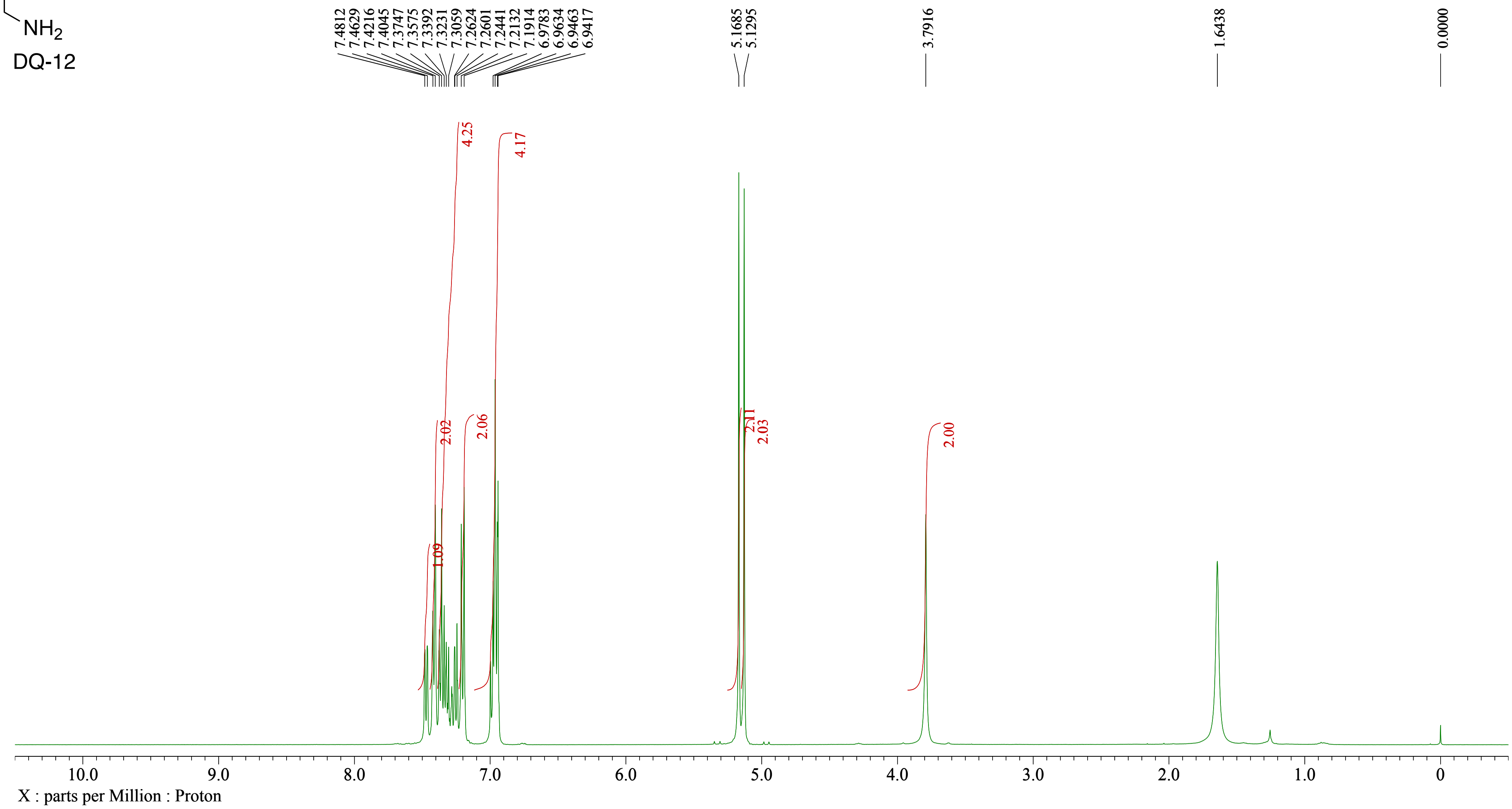

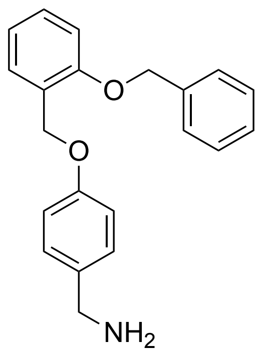

DQ-12

^{13}C NMR (100 MHz, $CDCl_3$)

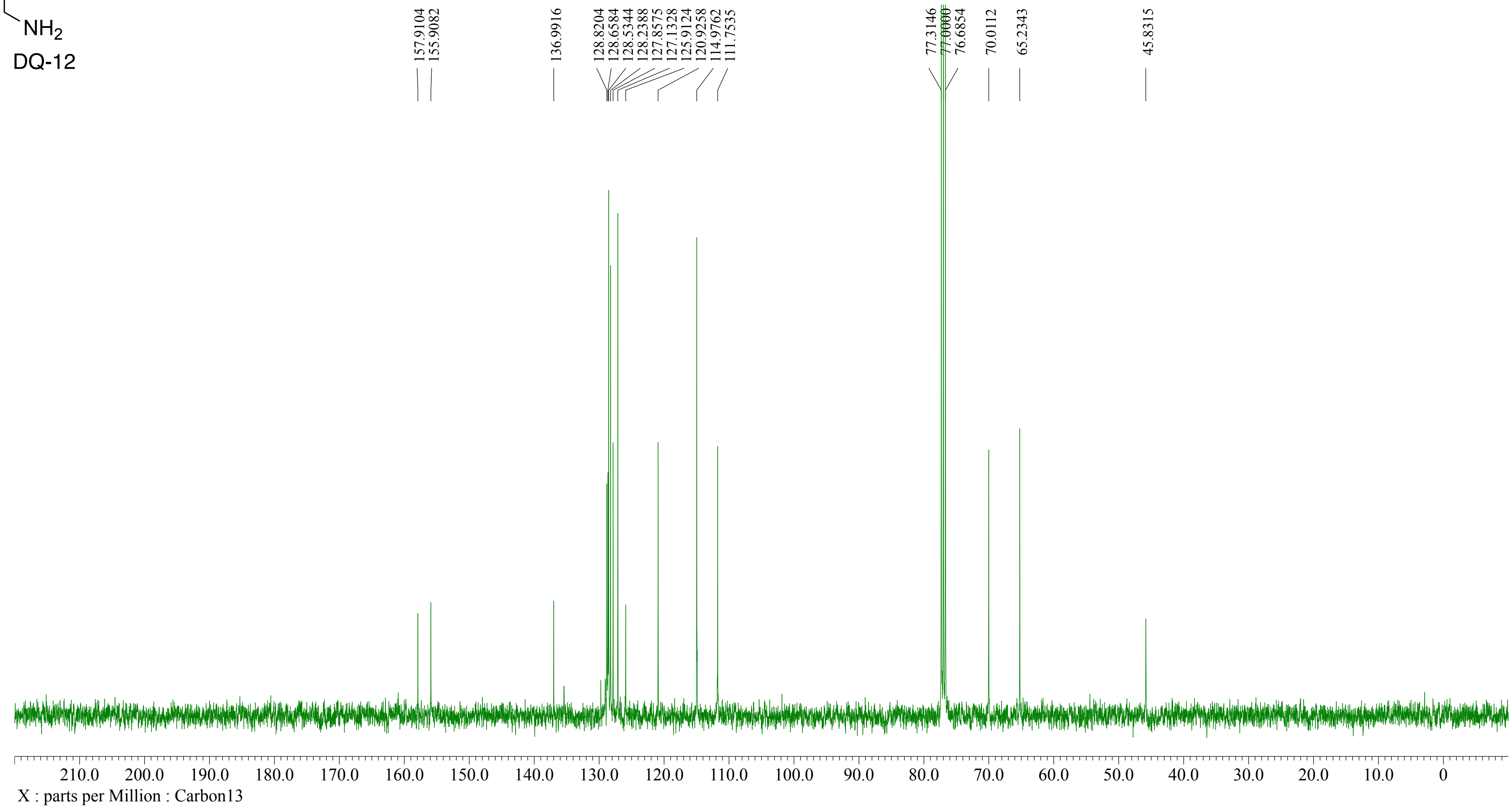

Supplement: Supplementary file 5 — Supplementary Data 2 [file 42003_2023_5334_MOESM5_ESM.pdf]
