# Supplementary material for: Deep learning driven de novo drug design based on gastric proton pump structures
Source: Commun Biol. 2023 Sep 19;6:956. doi: 10.1038/s42003-023-05334-8 (PMC10509173; doi:10.1038/s42003-023-05334-8)
Supplement: Supplementary file 9 — Supplementary Software [file 42003_2023_5334_MOESM9_ESM.zip › DQ_script/Vizard/templates/index.html]

{{ bokeh\_css }}
{{ bokeh\_js }}
MolExplorer


# Vizard

{{ plot\_div|indent(8) }}

{{ plot\_script|indent(8) }}
