# Supplementary material for: Deep learning driven de novo drug design based on gastric proton pump structures
Source: Commun Biol. 2023 Sep 19;6:956. doi: 10.1038/s42003-023-05334-8 (PMC10509173; doi:10.1038/s42003-023-05334-8)
Supplement: Supplementary file 1 — Peer Review File [file 42003_2023_5334_MOESM1_ESM.pdf]

nature portfolio

Peer Review File

Deep learning driven de novo drug design based on gastric proton pump structures

Open Access This file is licensed under a Creative Commons Attribution 4.0 International License, which permits use, sharing, adaptation, distribution and reproduction in any medium or format, as long as you give appropriate credit to the original author(s) and the source, provide a link to the Creative Commons license, and indicate if changes were made. In the cases where the authors are anonymous, such as is the case for the reports of anonymous peer reviewers, author attribution should be to 'Anonymous Referee' followed by a clear attribution to the source work. The images or other third party material in this file are included in the article's Creative Commons license, unless indicated otherwise in a credit line to the material. If material is not included in the article's Creative Commons license and your intended use is not permitted by statutory regulation or exceeds the permitted use, you will need to obtain permission directly from the copyright holder. To view a copy of this license, visit http://creativecommons.org/licenses/by/4.0/.

REVIEWERS' COMMENTS:

Reviewer #4 (Remarks to the Author):

The new discussions described in the ‘Response to Referees Letter’ are written well. It would be better if the authors could include the discussion of the estimated pKa value of DQ-designed compounds plus no pH dependence of them (presumed by the high pKa values of DQ compounds), in the Discussion section.
